# Virtual reality for neurorehabilitation: A bibliometric analysis of knowledge structure and theme trends

**DOI:** 10.3389/fpubh.2022.1042618

**Published:** 2022-11-10

**Authors:** Qi-Fan Guo, Lin He, Wei Su, Hui-Xin Tan, Lian-Yi Han, Chen-Fan Gui, Yi Chen, Han-Hong Jiang, Qiang Gao

**Affiliations:** ^1^Key Laboratory of Rehabilitation Medicine in Sichuan Province, West China Hospital, Sichuan University, Chengdu, China; ^2^Department of Rehabilitation Medicine, West China Hospital, Sichuan University, Chengdu, China; ^3^Biostatistics Group, State Key Laboratory of Genetic Engineering, Greater Bay Area Institute of Precision Medicine (Guangzhou), Fudan University, Guangzhou, China

**Keywords:** virtual reality, neurorehabilitation, bibliometric, hotspots, research trends

## Abstract

**Background:**

As an emerging technology, virtual reality (VR) has been broadly applied in the medical field, especially in neurorehabilitation. The growing application of VR therapy promotes an increasing amount of clinical studies. In this paper, we present a bibliometric analysis of the existing studies to reveal the current research hotspots and guide future research directions.

**Methods:**

Articles and reviews on the related topic were retrieved from the Science Citation Index Expanded of Web of Science Core Collection database. VOSviewer and Citespace software were applied to systematically analyze information about publications, countries, institutions, authors, journals, citations, and keywords from the included studies.

**Results:**

A total of 1,556 papers published between 1995 and 2021 were identified. The annual number of papers increased gradually over the past three decades, with a peak publication year in 2021 (*n* = 276). Countries and institutions from North America and Western European were playing leading roles in publications and total citations. Current hotspots were focused on the effectiveness of VR therapy in cognitive and upper limb motor rehabilitation. The clusters of keywords contained the four targeted neurological diseases of VR, while the burst keywords represented that the latest studies were directed toward more defined types of VR therapy and greater study design.

**Conclusions:**

Our study offers information regarding to the current hotspots and emerging trends in the VR for rehabilitation field. It could guide future research and application of VR therapy in neurorehabilitation.

## Introduction

Neurological conditions are major contributing factors to death and disability in the modern world (1, 2). It is recently estimated that neurological disorders affect more than one billion people (3), and the number will likely keep rising owing to the growth of the aging population. Most neurological patients report different degrees of impediment to motor, sensory, cognitive, and visual function, thereby resulting in severe functional limitations in performing activities of daily living, participation, and social interaction (4–6). According to the expanding research evidence, rehabilitation is the most effective way to reduce disability and a key link to neurological disease management (7, 8). Conventional neurorehabilitation with high-intensity, repetitive, and task-specific practice has confirmed a curative effect on improving the performance of neurological patients (8, 9). However, these rehabilitation techniques can be costly and inconvenient due to professional and resource-intensive requirements (10, 11). Therefore, a new effective treatment, different in perspective from existing treatments, is urgently required.

As health applications of new technologies develop, virtual reality (VR) is likely to become more widely used in the clinical rehabilitation setting. VR is a unique form of rehabilitation technique established by Morton Heiling in 1962 and has been evolving over the past 60 years (12–14). VR technology is defined as a system that allows users to interact with images and sounds in the virtual environment, which can stimulate response and provide real-time feedback concerning their performance (15). This technology can be combined with computers, mobile device screens, and head-mounted displays to better interact with users (16). Over the past decade, VR has gradually become a valuable tool for assessment and intervention in clinical rehabilitation due to the continuous research and reduction in the cost of virtual technology (17). The potential therapeutic mechanisms include task-oriented repetition, strategic feedback, and embodied simulation, while the environmental enrichment effect of VR therapy has also been documented in previous research (18–20).

As a non-invasive therapeutic approach, VR therapy has attracted plenty of studies on neurological disorders management (21, 22). Common examples of VR on neurorehabilitation are as follows: (1) VR-based treadmill training for regaining gross motor function and balance (23, 24), (2) manipulating virtual objects exercises for fine motor skill acquisition (25, 26), and (3) performing various activities using individual game platforms for improvement in quality of life (23, 27, 28). The VR-based experimental approaches can simulate a real-life safe setting, and it has been considered an essential part of effective neurorehabilitation with demonstrated improved motivation (29). In addition, under the current status of epidemic outbreak and the increasing rehabilitation requirements of patients with neurological diseases, it is time to shift the traditional face-to-face rehabilitation therapy to telemedicine *via* VR and other digital tools (30). The growing application of VR therapy has turned it into a meaningful research field. However, these papers tend to be complex and unstructured, while the therapeutic mechanisms of VR also remain unclear. Moreover, no studies performed a cutting-edge overview of VR on neurorehabilitation through bibliometric analysis to this point.

To fill the gap in the quantitative analysis of this research hotspot, this bibliometric study aims to obtain the global scientific outputs of VR for neurorehabilitation field from the inception to 2021. We used the Citespace and VOSviewer to comprehensively analyze publications based on the Web of Science Core Collection (WoSCC) database. This article will help clinicians and researchers to comprehend the hotspots and emerging trends in this field, to guide clinical practice and future research development.

## Materials and methods

### Data sources and search strategy

**Figure 1:**
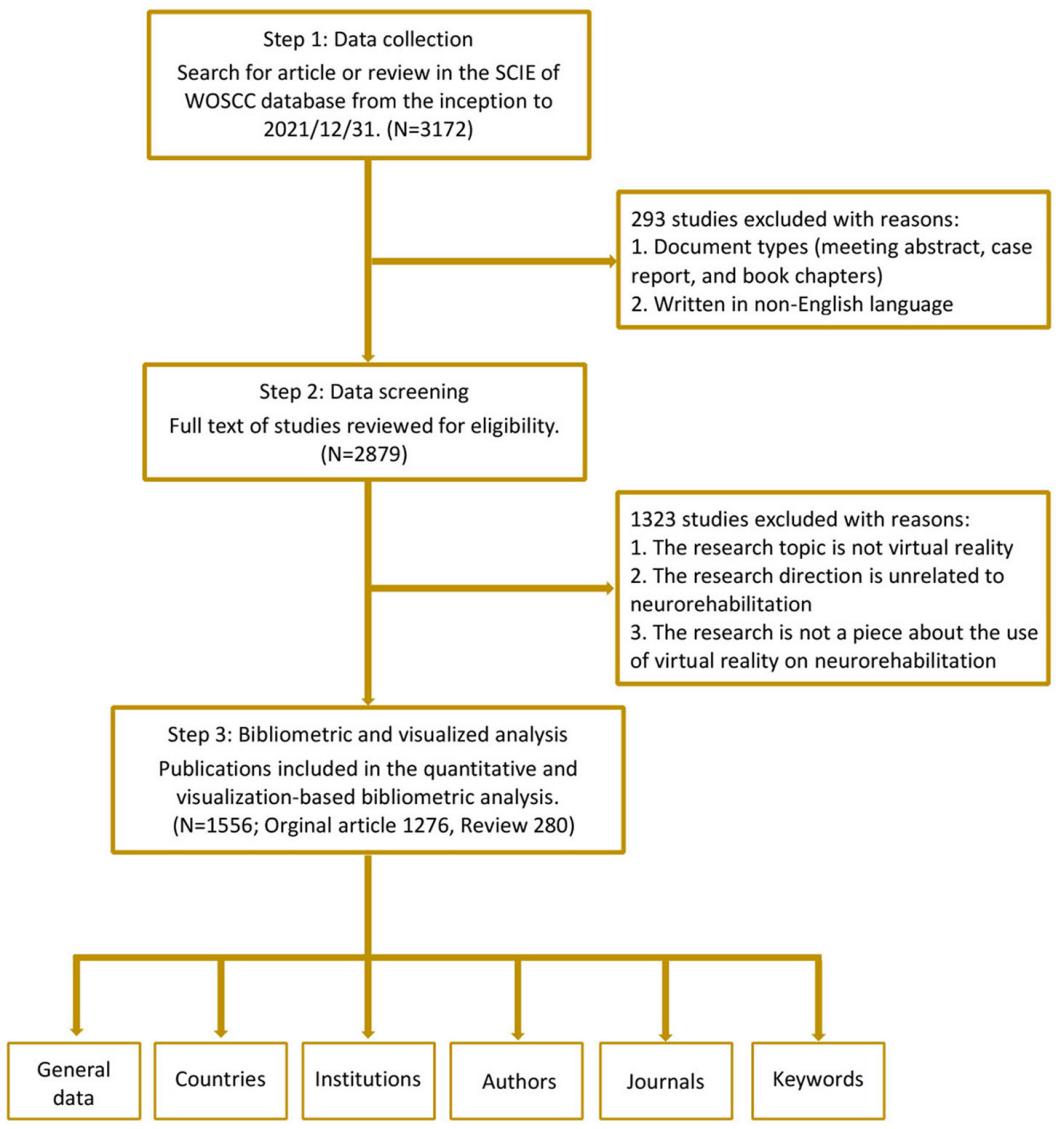
Flow chart of the bibliometric search and analysis process.

Publications with related themes from the inception to 2021 were searched from the Science Citation Index Expanded (SCIE) of the WoSCC database on 15th August 2022. Two authors (Q-FG and LH) independently performed the retrieval, and the search strategy was presented in Supplementary material 1. We only selected articles or reviews in English, and all included documents were required to undergo peer review. All bibliometric data were imported into Endnote X9, and then two researchers (Q-FG and LH) screened the titles, abstracts, and full texts of the included papers to identify the available studies independently based on the pre-decided exclusion criteria. Exclusion criteria were as follows: (1) The intervention modality is not VR; (2) Targeted conditions are unrelated to neurorehabilitation; (3) The theme of the paper is uncorrelated to the implementation of VR therapy on neurorehabilitation. Finally, it yielded a total of 1,556 documents. The flowchart outlining the bibliometric search and analysis process is demonstrated in Figure 1.

### Data extraction and analysis

After the screening and check *via* Endnote, the available documents were manually selected from WOS database. Then, plain texts containing information of these documents downloaded from WOS database were analyzed by VOSviewer and Citespace for further analysis (Supplementary material 2). VOSviewer and Microsoft Excel were used to capture basic information and perform the co-occurrence or co-citation map across different countries, institutions, authors, and journals. The node size in the VOSviewer co-occurrence graph reflects the number of published articles, and connecting lines between the nodes indicate cooperation strength. The color of the circles represents the cluster to which the node belongs. In addition, Citespace was applied to analyze the co-occurrence network of keywords, cluster network, and burst keywords analysis, indicating cutting-edge knowledge and research trends. GraphPad Prism 8 was used to describe the amount of publications among various countries and their cooperation strength.

## Results

### Publication outputs and growth trend

**Figure 2:**
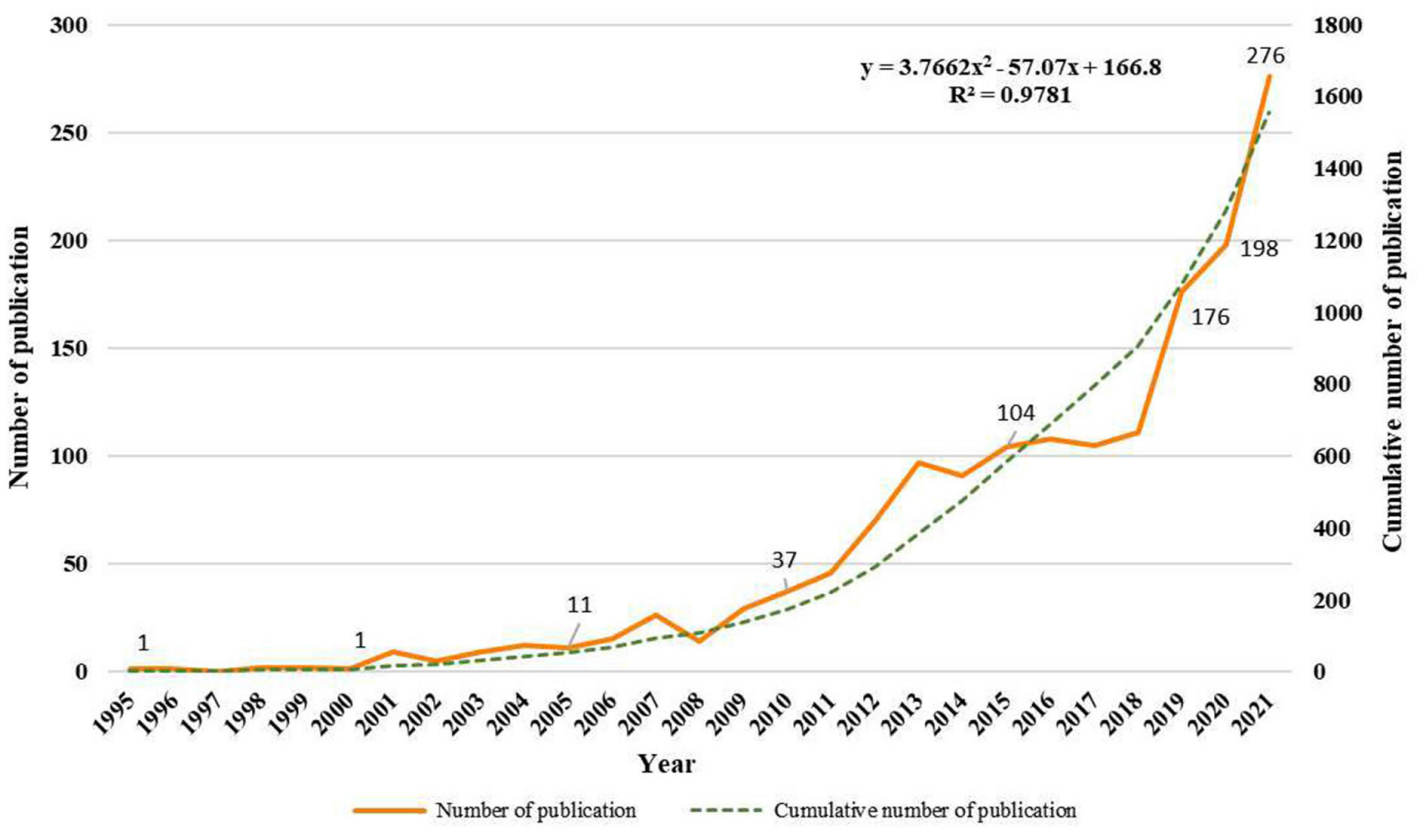
Trend of publication outputs from 1995 to 2021 on VR for neurorehabilitation topic.

The initial retrieval of the WoSCC database identified 3,172 publications. After excluding other document types, limited English language, and unrelated research topics, 1,556 papers were finally enrolled in the analysis, including 1,276 articles [207 randomized controlled trial (RCT) and 1,069 non-RCT] and 280 reviews. These papers were published from 1995 to 2021. The first publication about VR application for neurorehabilitation was published in 1995 by Pugnetti et al. (31), which provided an extensive review of the use of immersive VR therapy in patients with cognitive impairment. Figure 2 describes the annual numbers and bibliometric trends in 1,556 publications. The timing of publication could be divided into three phases: the infancy phase (1995–2008), the slow-growth phase (2009–2017), and the rapid-growth phase (2018–2021). In the infancy phase, the annual publication remained under 15 papers besides 2007. Over the slow-growth phase, a slow and steady upward tendency was exhibited from 29 papers in 2009 to 105 papers in 2017. In the rapid-growth phase, the amount of papers yielded a huge growth, with more than 100 publications each year. Nearly half of the publications were released in the final stage, with a peak publication year in 2021 (*n* = 276). Meanwhile, the linear regression analysis showed that the publications were positively correlated with the publication year (*R*^2^ = 0.9781, *p* < 0.001). Research in this area is expected to continue to grow in the future.

### Active countries

**Figure 3:**
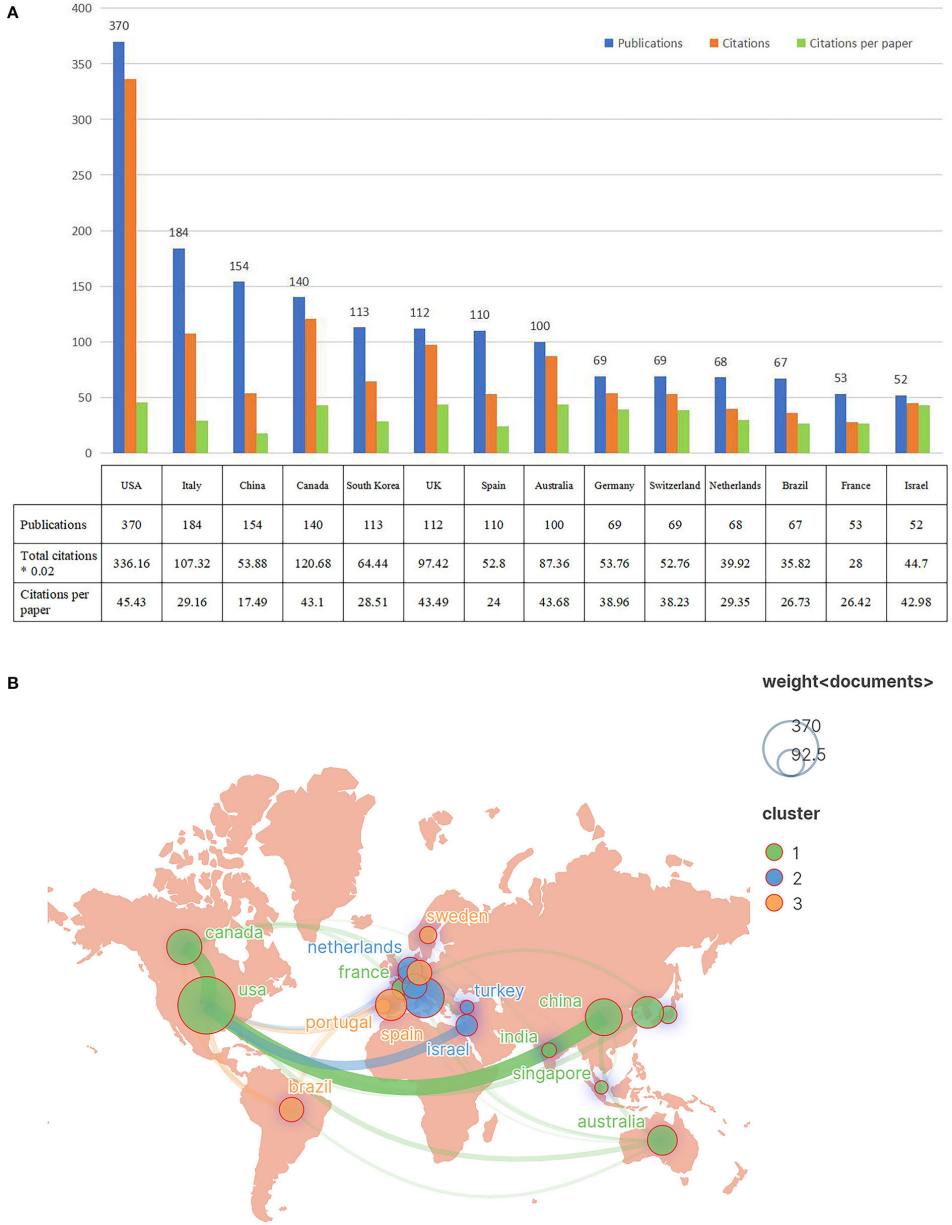
The top 14 prolific countries and international collaboration network on VR for neurorehabilitation research. **(A)** The number of publications, total citations, and citations per paper in the top 14 countries. **(B)** The co-operative network visualization map of countries.

A total of 68 countries participated in the publications on VR for neurorehabilitation topics. The top 14 countries with 50 or more papers are displayed in Figure 3A. The five most active nations were the United States (370, 23.8%), Italy (184, 11.8%), China (154, 9.9%), Canada (140, 9.0%), and South Korea (113, 7.3%). Amongst the 14 countries with the highest output of publications, only China and Brazil were developing countries, while others were developed countries. Research from the first-ranked United States was cited 16,808 times, followed by Canada (6,034), and Italy (5,366). In terms of citations per paper, the United States also took first place (45.43), followed by Australia (43.68) and the United Kingdom (43.49). Geographical data of included papers were extracted by VOSviewer and imported into Scimago Graphica to generate the cooperative network among countries in Figure 3B. Obviously, there were three main country clusters, including North America, Western European, and Eastern Asia. The strongest cooperation was between the United States and China.

### Institution distributions

**Figure 4:**
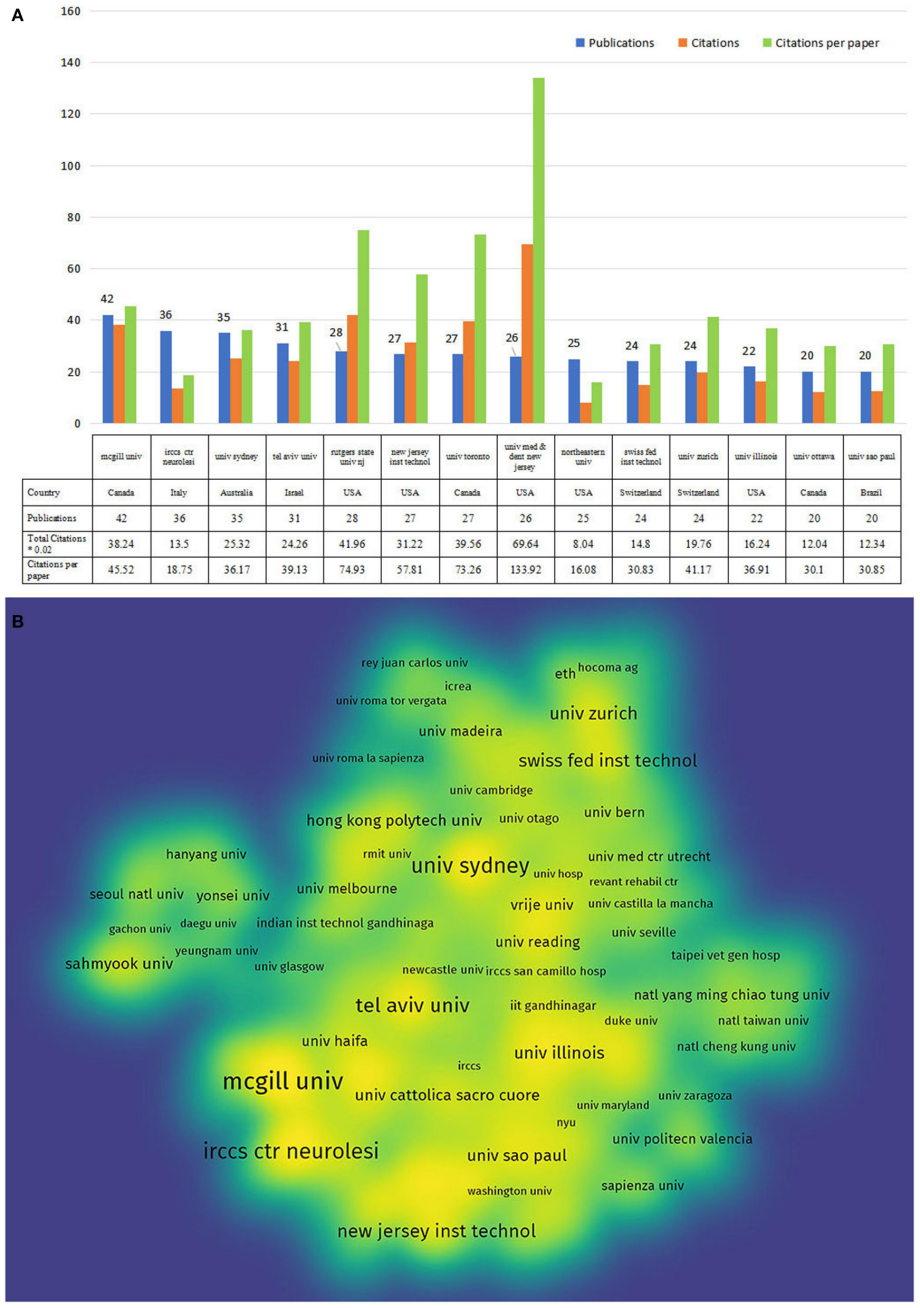
The top 14 active institutions and the inter-institutional collaboration network on VR for neurorehabilitation research. **(A)** The number of publications, total citations, and citations per paper in the top 14 institutions. **(B)** The density visualization map of institutions.

In accordance with the author address, 1,994 institutions contributed to these 1,556 publications. The 14 most productive institutions publishing more than 20 papers are listed in Figure 4A. The most prolific institution was McGill University (Canada, 42 papers), followed by IRCCS Centro Neurolesi Bonino Pulejo (Italy, 36 papers) and the University of Sydney (Australia, 35 papers). The papers by the University of Medicine and Dentistry of New Jersey-Robert Wood Johnson Medical School had the most citations (3,482 times) and citations per paper (113.92). Figure 4B shows the cooperative network among the top institutions engaging in VR for neurorehabilitation research. The top organizations showed extensive relationships with others, while the yellow color block indicated the density of cooperation between institutions. There were close cooperation between different institutions, especially in McGill University, Tel Aviv University, and The University of Sydney.

### Author analysis

**Figure 5:**
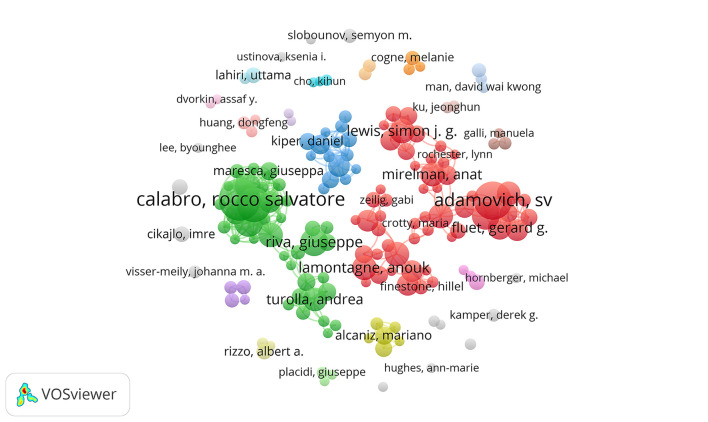
Network map of active authors contributed to VR for neurorehabilitation research.

A total of 5,885 authors engaged in the documents on VR in neurorehabilitation field. Table 1 shows the top 10 most active authors and their related information. The top three authors with the highest output were Calabro RS (35 papers), Adamovich SV (31 papers), and De Luca R (27 papers). Five of the top 10 authors were from IRCCS Ctr Neurolesi Bonino Pulejon, while others were scattered in research units. In terms of total citations and citations per paper, Deutsch JE from the University of Medicine and Dentistry of New Jersey ranked first (2,243 citations, 131.94 citations/paper), as shown in Table 1. Additionally, H-index could accurately reflect the academic achievement of the author. Levin MF ranked first (32) on H-index and held the greatest influence in this field. In Figure 5, an overlay visualization map of author co-occurrence analysis was generated by VOSviewer. The graph forms two main clusters centered on Calabro RS and Adamovich SV. There were discrete co-operations between them, but authors who worked together held a robust partnership.

**Table 1:** The top 10 active authors who published literature on VR for neurorehabilitation.

**Rank**	**Author**	**Institution**	**Country**	**Publications**	**Citations**	**Citations per paper**	**H-index**
1	Calabro, Rocco Salvatore	IRCCS Ctr Neurolesi Bonino Pulejon	Italy	35	676	19.31	30
2	Adamovich, SV	New Jersey Institute of Technology	USA	31	1,643	53.00	26
3	De luca, Rosaria	IRCCS Ctr Neurolesi Bonino Pulejo	Italy	27	571	21.15	21
4	Naro, Antonino	IRCCS Ctr Neurolesi Bonino Pulejo	Italy	21	398	18.95	24
5	Merians, AS	Rutgers, The State University of New Jersey	USA	20	1,353	67.65	24
6	Riva, Giuseppe	IRCCS Institution Auxologico Italiano	Italy	20	394	19.70	14
7	Maggio, Maria Grazia	IRCCS Ctr Neurolesi Bonino Pulejo	Italy	18	305	16.94	14
8	Bramanti, Placido	IRCCS Ctr Neurolesi Bonino Pulejo	Italy	17	437	25.71	30
9	Deutsch, Judith E.	University of Medicine and Dentistry of New Jersey	USA	17	2,243	131.94	31
10	Levin, Mindy F.	McGill University	Canada	17	1,391	81.82	49

### Journal characteristic

**Figure 6:**
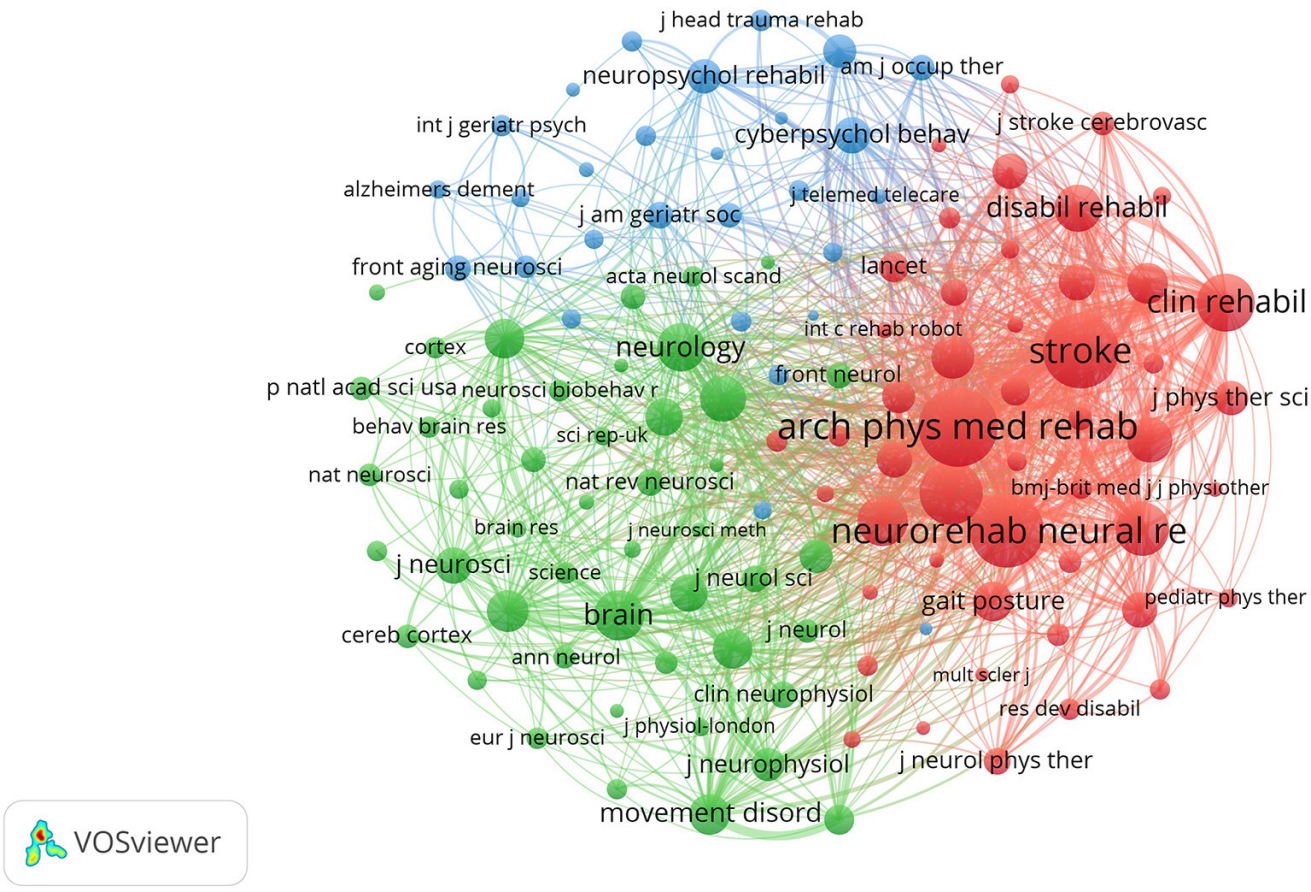
Co-citation network map of journals.

The included publications were published in 416 academic journals. As per Bradford's law, core journals were identified as journals publishing more than one-third of all related papers, indicating 17 core journals and 399 non-core journals in this research field. The top 10 most productive journals accounted for 24.29 % (378 papers) of all research, as shown in Table 2. Journal of neuroengineering and rehabilitation published the most papers (102 papers), followed by Frontiers in neurology (39 papers) and IEEE transactions on neural systems and rehabilitation engineering (36 papers). Regarding the impact factor (IF) of journals, only one of the top 10 journals held an IF >5.000 (Journal of neuroengineering and rehabilitation, 5.208). Seven journals had an IF varying from 2.000 to 5.000, one had an IF <2.000, and one was not assigned an IF to the present. VOSviewer was used to yield the co-citation map of journals, including 124 journals with at least 100 citations. The top three co-cited journals were Archives of physical medicine and rehabilitation (4.060), Stroke (10.170), and Neurorehabilitation and neural repair (4.895), which were representative and professional journals in this field (Figure 6).

**Table 2:** The top 10 most productive journals in the VR for neurorehabilitation field.

**Rank**	**Journal**	**Publications**	**Citations**	**Citations per paper**	**IF**	**JCR**	**OA**
1	Journal of Neuroengineering and Rehabilitation	102	4,150	40.69	5.208	Q2	Yes
2	Frontiers in Neurology	39	583	14.95	4.086	Q2	Yes
3	IEEE Transactions on Neural Systems and Rehabilitation Engineering	36	1,555	43.19	4.528	Q1	No
4	Neurorehabilitation	35	780	22.29	1.986	Q3	No
5	Archives of Physical Medicine and Rehabilitation	31	1,904	61.42	4.060	Q1	No
6	Disability and Rehabilitation	31	847	27.32	2.439	Q2	No
7	Journal of Physical Therapy Science	27	685	25.37	/	/	No
8	Neurorehabilitation and Neural Repair	26	1,323	50.88	4.895	Q1	No
9	Topics in Stroke Rehabilitation	26	1,037	39.88	2.177	Q3	No
10	Plos One	25	902	36.08	3.752	Q2	Yes

### Analysis of keywords

**Figure 7:**
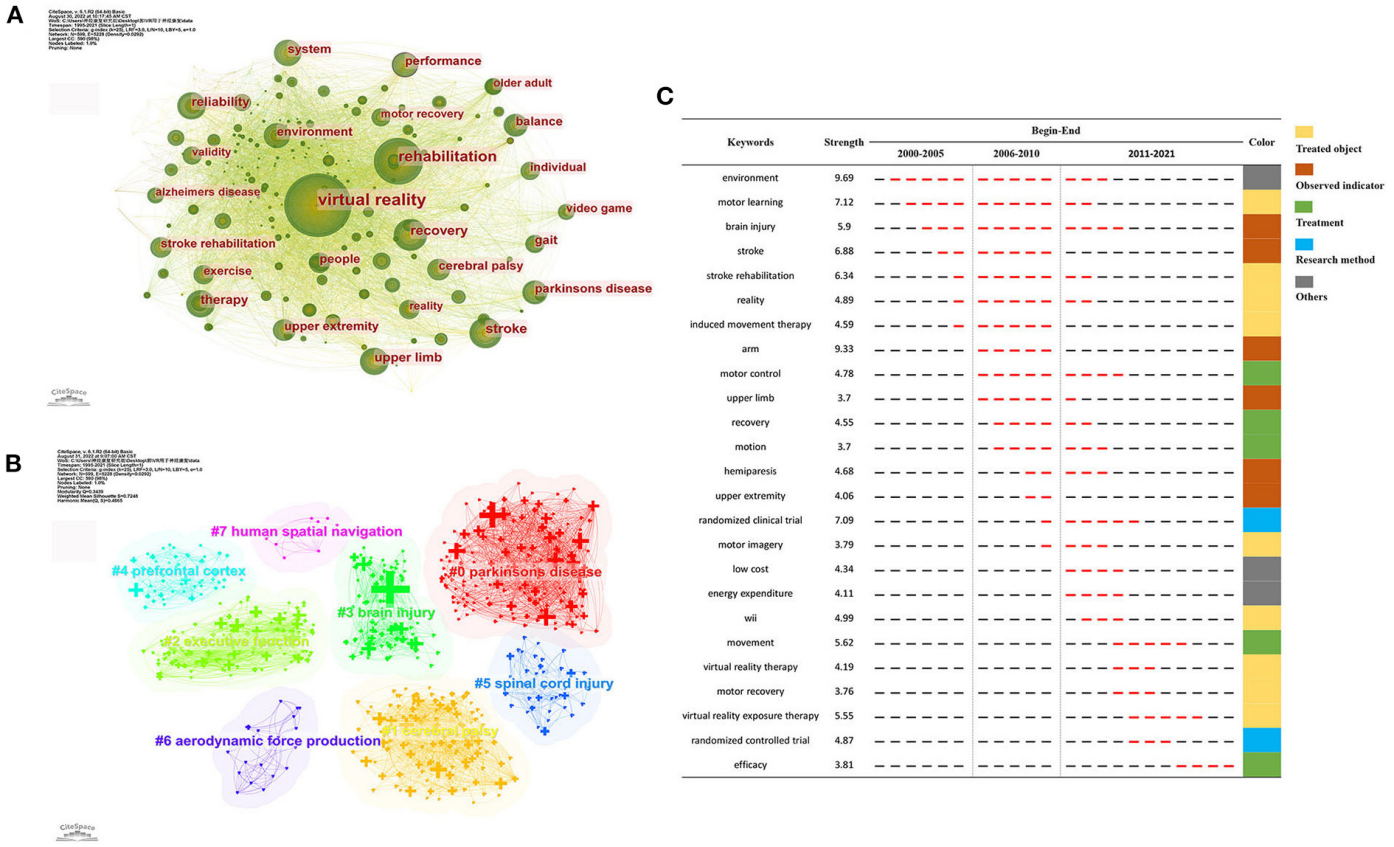
Analysis of keywords related to publications on VR for neurorehabilitation field. **(A)** The keyword co-occurrence network map. **(B)** The keyword cluster map. **(C)** The top 25 keywords with the strongest citation bursts.

Keywords are the core summary of a paper. These high-frequency or burst keywords could reflect the current themes and predict future research frontiers. As shown in Figure 7A, the top three keywords with the highest occurrence were virtual reality, rehabilitation, and stroke. The overall keywords could be divided into eight clusters depending on their type (Figure 7B). Clusters #0, #1, #3, and #5 mainly described the target patients of VR therapy, including Parkinson's disease, cerebral palsy, brain injury, and spinal cord injury, which were also the major populations in neurorehabilitation. Clusters #2 and #7 mainly focused on the various function in which VR therapy might serve a role, with other clusters representing the game design of VR and stimulated brain area.

In addition, Citespace was used to generate the top 25 keywords with the strongest burst, and the results are indicated in Figure 7C. Based on the burst time, keywords were divided into three periods: from 2000 to 2005, from 2006 to 2010, and from 2011 to 2021. Among these keywords, environment and arm held the highest burst strength. Wii, virtual reality exposure therapy, motor recovery, and randomized controlled trial were presented as the most recent burst keywords, suggesting the research directions in the near future.

## Discussion

### Overview of the results

In this bibliometric study, 1,556 papers focusing on VR in neurorehabilitation were included and visualized by VOSviewer and Citespace to display the research hotspots and trends of this field. The publications on the related topic might reveal variations in research activity and productivity, which could be classified into three phases. Before 2009, the number of papers remained largely unchanged. VR technology has not been widely applied in the medical field due to the high cost and immature technologies. With the reduced costs and the availability of high-quality technologies in the 2010s, the number of papers steadily increased, and it attracted the increasing attention of medical staff and researchers. Since 2020, the annual publication has grown dramatically to over 200 publications in 2021. This is undoubtedly due to the explosion of COVID-19 facilitating the development of telerehabilitation techniques, such as VR therapy (33, 34). It is foreseeable that this research area will maintain its popularity in the near future.

Regarding the nation of researchers, over one-third of countries worldwide engaged in publications on VR for neurorehabilitation topics. Without surprise, the USA, Italy, Canada, UK, and Spain took the dominant places in this field that were also the driving forces in other telemedicine research domains (35, 36). This is probably owing to the large national gross domestic product (GDP) which can provide sufficient support for clinical research, and the higher incidence and prevalence of neurological disease in these Western European and North American countries (4). In addition, only two emerging countries, namely China and Brazil, have displayed their presence in the past 5 years. The financial constraint and inadequate attention of such developing countries hinder their sustained investment in the application of novel intelligent technologies to healthcare management. However, with the ongoing innovation of intelligent technologies such as artificial intelligence and the advent of the 5G era, the availability of more portable, individualized, and affordable VR devices appears increasingly realistic in the healthcare area, which will definitely facilitate the general research and application of VR technology across the world.

In terms of the researchers and their institutions, the most active and highlighted institutions listed in the Figure 4A were nearly well-established universities from developed countries with rich academic resources, which could also be observed in the co-occurrence network map of the author. As shown in Table 1, half of the top 10 investigators were affiliated with IRCCS Ctr Neurolesi Bonino Pulejon, Italy, and all held lower citations than those from North American countries. A possible explanation may be that Italian fellows published papers mostly on the post-2015 period with limited cited length. Besides the above indicators, core journals with high publications could offer fundamental insights into a certain domain. The number of papers published in the top 10 journals was less than one-third of the overall papers. These findings implied that publications on VR for neurorehabilitation were broadly distributed across multiple journals. In addition, these active journals did not have a high IF, with only one journal reaching a score of 5. Therefore, the level and quality of studies in VR for neurorehabilitation need to be improved, and it requires more international cooperation between authors to perform high-quality clinical research.

### Hotspots analysis

The author clusters could assist in indicating the past, present, and future hotspots in a certain field. As indicated in Figure 5, the authors engaging in the publication of 1,556 papers could be divided into two groups, with Calabro RS and Adamovich SV as the primary ongoing force, respectively.

The team of Calabro RS is focused on investigating the benefits of the VR approach to enhancing cognitive function in patients with neurological disorders, especially stroke patients (37–40). According to previous research, cognitive impairment is common among patients with neurological diseases, and the incidence of cognitive impairment in stroke patients was 78% (41). Virtual technology has confirmed therapeutic efficacy in cognitive rehabilitation in that VR can simulate real-life scenarios in a safe valid setting for patients to reproduce their real cognitive performance (42). Davis et al. (43) reported that significant improvement in the cognitive ability of patients with dementia was observed after receiving a VR exercise program, with its safety also demonstrated.

The Adamovich SV research group is currently interested in the effectiveness of VR applications coupled with rehabilitation robots in enhancing upper extremity motor function in patients with neurological disorders (11, 44, 45). Severe gross and fine motor dysfunction is generally present in patients suffering from neurological diseases. The slow and partial recovery in upper limb function is the greatest challenge, and improving upper limb dysfunction is still a major research priority in this field (46). The 2008 EBRSR guideline (47) recommended VR technology as an effective method to improve patients' motor function during stroke recovery, clearly indicating that computer-assisted sensorimotor training could effectively reduce the functional limitations of the upper extremity. This may be because enough sensory feedback, the essential component of motor learning, can cause increased activity of the corticospinal system and produce better motor function as a result (48). The therapeutic effect of VR can be combined with rehabilitation robots and other computer-assisted technology to yield better recovery for patients (33, 49).

### Keywords and trend analysis

The cluster analysis of co-occurrence and burst keywords could reveal the frontier topics and emerging trends in a given field. Based on the clustering analysis, it could be concluded that the major targeted illnesses of VR therapy were still aimed at four major diseases, namely Parkinson's disease, cerebral palsy, brain injury, and spinal cord injury. Moreover, apart from the above-mentioned upper limb motor and cognitive functions, more studies have been conducted to investigate the benefits of VR on human spatial navigation and executive function, and the potential activated brain area of VR therapy might locate in the prefrontal cortex.

As observed in Figure 7C, the theme of the included studies underwent three variation stages (Phase I, 2000–2005; Phase II, 2006–2010; Phase III, 2011–2021). The literature published in 2005 and earlier paid more attention to routine rehabilitation for neurological disorders (stroke, brain injury), with less content directed toward VR. Since 2006, researchers around the globe have started to show more interest in adapting VR to enhance upper limb motor function (arm, upper limb, hemiparesis, motor) in patients with neurological disorders. Following the breakthrough of VR technology in the 2010s, several types of VR therapy (Nintendo Wii, Xbox Kinect) and VR-related proper nouns (virtual reality exposure therapy) became available with a more standardized research design (RCT). Over the last 3 years, studies have mainly verified the efficacy of VR therapy on patients with neurological disorders *via* RCTs, representing the state-of-the-art trend in VR for neurorehabilitation.

### Summary of VR intervention

The consensus that VR can restore or improve function levels in neurorehabilitation has reached in recent decades. Still, some obstacles remain to the successful application of VR therapy: (1) Various technical limitations regarding the software and hardware capabilities are left to be tackled to meet clinical requirements and individualization. (2) Patients who had suffered adverse reactions (e.g., motion sickness, feeling disoriented, and eye strain) were monitored in previous VR-related trials (32, 50, 51), which are expected to explore in future research. (3) The gap in population diversity, such as the older adults' acceptance of VR technology, needs more research. (4) Ethical and political factors should also be considered in the subsequent system design. Moreover, the epidemic outbreak can be an impetus for driving the adoption of VR in telemedicine to comply with public health rules and interrupt the spread of COVID-19. Virtual technology has been considered helpful in improving the efficiency of healthcare workers, reducing the risk of outbreaks, and enabling patients with neurological disorders to access suitable therapies with efficacy monitoring, which are likely to be the future application scenario (34, 52–54). The findings of our paper may provide some guidance and inspire researchers to perform more studies and clinical applications in this field.

### Strength and limitations

This is the first review to summarize the current publications and development trends of VR for neurorehabilitation from the perspective of bibliometrics. To evaluate as systemically and comprehensively as possible, this study collected 1,556 related papers published over the last three decades from the WoSCC database. Furthermore, we utilized the popular bibliometric tools, Citespace and VOSviewer, to quantitative analyze VR therapy in neurorehabilitation field in conjunction with the specific data in countries, institutions, authors, journals, citations, and keywords.

However, the limitations of our work need to be acknowledged. First, this study was limited to the Web of Science database, and the retrieved results of other databases (e.g., Pubmed, Scopus, and Google scholar) might be inconsistent with the findings of the present paper. Second, only studies in English were eligible for the bibliometric analysis, which may cause publication bias. Third, our search strategy in this article was formulated based on Topic (TS) and not strict enough, which may lead to the inclusion of some less relevant papers and caused some bias to the final results. Future researchers are recommended to adopt Title (TI) or Author Keywords (AK) to design a more accurate search formula to retrieve the results in this research domain. Fourth, the current work aims to reflect the overall landscape of this research field. Specific curative effects and applied modes of VR therapy on neurorehabilitation need to be refined in more well-designed trials or systematic reviews with a focused scope, to facilitate the development of clinical guidelines.

## Conclusion

This bibliometric study provides a deeper insight into research on VR therapy for neurorehabilitation. In the past three decades, the number of studies in this field has exhibited an upward trend, with North America and Western European occupying a leading role in publications and total citations. Most journals hold a low IF, which deserves more future attention. The author co-occurrence analysis identified two predominant clusters centering on cognitive rehabilitation by Calabro RS, and upper limb motor rehabilitation by Adamovich SV, respectively. The most recent research trends cover more defined types of VR therapy and greater study design. These findings may assist future researchers in better comprehending the current hotspots and future development trends in this field.

## Data availability statement

The original contributions presented in the study are included in the article/Supplementary materials, further inquiries can be directed to the corresponding author.

## Author contributions

Q-FG, LH, WS, H-XT, and L-YH acquired, analyzed the data, and drafted the manuscript. Q-FG, LH, C-FG, YC, H-HJ, and QG designed the research, acquired the article information, and revised the manuscript. All authors contributed to the article and approved the submitted version.

## Conflict of interest

The authors declare that the research was conducted in the absence of any commercial or financial relationships that could be construed as a potential conflict of interest.

## Publisher's note

All claims expressed in this article are solely those of the authors and do not necessarily represent those of their affiliated organizations, or those of the publisher, the editors and the reviewers. Any product that may be evaluated in this article, or claim that may be made by its manufacturer, is not guaranteed or endorsed by the publisher.
