# Supplementary material for: Virtual reality for neurorehabilitation: A bibliometric analysis of knowledge structure and theme trends
Source: Front Public Health. 2022 Nov 10;10:1042618. doi: 10.3389/fpubh.2022.1042618 (PMC9684719; doi:10.3389/fpubh.2022.1042618)
Supplement: Supplementary file 1 [file Table_1.DOCX]

Search Strategy:

1.Database: Web of Science core collection (to 31th December 2021).

#1 TS=((“Virtual reality”) OR (VR)) (32063)

#2 TS=(hemipleg* or hemipar* or paresis* or paretic* or stroke* or paralysis* or “cerebrovascular disorder*” or “cerebrovascular accident*” or “cerebral pals*” or paraplegi* or paraparesis or qadriplegi* or quadriparesi* or tetraplegi* or tetraplagi* or tetraparesis or “central cord injury syndrome” or “myelopathy” or “peripheral nerve inj*” or “nerve regeneration” or “peripheral nerves” or dement* or neurorehabilitation or parkinson*) (855266)

#3 TS=((brain* or cerebr* or cerebell* or intracran* or intracerebral or head or crani* or skull* or hemispher* or intercran* or “spinal cord” or spine or spinal) and (ischemi* or infarct* or thrombo* or emboli* or occlus* or fractur* or wound* or trauma* or injur* or damag* or lesion* or contusion* or laceration* or oedema* or edema* or swell* or contusion* or concus* or haematoma* or hematoma* or haemorrhag* or hemorrhag* or bleed*)) (819403)

#4 #2 OR #3 (1468277)

#5 #1 AND #4 (3172)
