# Supplementary material for: Virtual reality for neurorehabilitation: A bibliometric analysis of knowledge structure and theme trends
Source: Front Public Health. 2022 Nov 10;10:1042618. doi: 10.3389/fpubh.2022.1042618 (PMC9684719; doi:10.3389/fpubh.2022.1042618)
Supplement: Supplementary file 2 [file Table_2.DOCX]

FN Clarivate Analytics Web of Science

VR 1.0

PT J

AU Abbruzzese, G

Marchese, R

Avanzino, L

Pelosin, E

AF Abbruzzese, Giovanni

Marchese, Roberta

Avanzino, Laura

Pelosin, Elisa

TI Rehabilitation for Parkinson's disease: Current outlook and future

challenges

SO PARKINSONISM & RELATED DISORDERS

LA English

DT Article; Proceedings Paper

CT 21st World Congress on Parkinson's Disease and Relative Disorders

CY DEC 06-09, 2015

CL Milan, ITALY

DE Parkinson's disease; Rehabilitation; Physical exercise; Plasticity

ID VIRTUAL-REALITY; EXERCISE; MOTOR; THERAPY; NEUROPLASTICITY; RISK

AB Rehabilitation is considered as an adjuvant to pharmacological and surgical treatments for Parkinson's disease (PD) to maximize functional ability and minimize secondary complications. Originally, approaches were based on empirical experience, but growing evidence suggests that exercise-dependent plasticity constitutes the main mechanism underlying the effects of physiotherapy. Exercise increases synaptic strength and influences neurotransmission, thus potentiating functional circuitry in PD. In addition, exercise is a pivotal element of motor learning. PD patients retain a sufficient capacity of motor learning, though learning rates and performance are reduced in comparison to normal controls. Recent meta-analyses demonstrated that rehabilitation could induce short-lasting, but clinically important benefits, particularly for gait and balance. However, the interventions are largely heterogeneous (stretching, muscle strengthening, balance, postural exercises, occupational therapy, cueing, treadmill training), and there is still no consensus about the optimal approach. Innovative techniques have been recently proposed: virtual reality and exergaming, motor imagery and action observation, robot-assisted physiotherapy and non-conventional therapies (e.g.: dance, martial arts). The rehabilitative program for PD should be "goal-based" (targeted to practicing and learning specific activities in the core areas), but a number of practice variables (intensity, specificity, complexity) need to be identified and the program should tailored to the individual patients' characteristics. (C) 2015 Elsevier Ltd. All rights reserved.

C1 [Abbruzzese, Giovanni; Marchese, Roberta; Pelosin, Elisa] Univ Genoa, Ctr Parkinsons Dis, Dept Neurosci Rehabil Ophthalmol Genet & Maternal, I-16132 Genoa, Italy.

[Abbruzzese, Giovanni; Marchese, Roberta; Pelosin, Elisa] IRCCS S Martino Hosp IST, Unit Rehabil & Funct Recovery, Genoa, Italy.

[Avanzino, Laura] Univ Genoa, Dept Expt Med, Sect Human Physiol, I-16132 Genoa, Italy.

[Avanzino, Laura] Univ Genoa, Ctr Polifunz Sci Motorie, I-16132 Genoa, Italy.

RP Abbruzzese, G (通讯作者)，Univ Genoa, DINOGMI, Largo Daneo 3, I-16132 Genoa, Italy.

EM giabbr@unige.it

RI Avanzino, Laura/K-6623-2016; Marchese, Roberta/AAB-9484-2019; Pelosin,

Elisa/AAA-9637-2019

OI Marchese, Roberta/0000-0003-2321-088X; Pelosin,

Elisa/0000-0002-9880-2241; AVANZINO, LAURA/0000-0001-6286-1509

CR Abbruzzese G, 2014, FRONT HUM NEUROSCI, V8, DOI 10.3389/fnhum.2014.00961

Ahlskog JE, 2011, NEUROLOGY, V77, P288, DOI 10.1212/WNL.0b013e318225ab66

Barry G, 2014, J NEUROENG REHABIL, V11, DOI [10.1186/1743-0003-11-60, 10.1186/1743-0003-11-33]

Beall EB, 2013, BRAIN CONNECT, V3, P190, DOI 10.1089/brain.2012.0104

Corcos DM, 2013, MOVEMENT DISORD, V28, P1230, DOI 10.1002/mds.25380

Deane KHO, 2002, MOVEMENT DISORD, V17, P984, DOI 10.1002/mds.10197

ERICKSON DJ, 1956, JAMA-J AM MED ASSOC, V162, P1041, DOI 10.1001/jama.1956.02970280021008

Fisher BE, 2013, NEUROREPORT, V24, P509, DOI 10.1097/WNR.0b013e328361dc13

Frazzitta G, 2010, MOVEMENT DISORD, V25, P1762, DOI 10.1002/mds.23316

Hirsch MA, 2009, EUR J PHYS REHAB MED, V45, P215

King L.A., 2008, MOVEMENT DISORD, V28, P1587

Li FZ, 2012, NEW ENGL J MED, V366, P511, DOI 10.1056/NEJMoa1107911

Marchese R, 2000, MOVEMENT DISORD, V15, P879, DOI 10.1002/1531-8257(200009)15:5<879::AID-MDS1018>3.0.CO;2-9

Marinelli L, 2009, PARKINSONISM RELAT D, V15, P6, DOI 10.1016/j.parkreldis.2008.02.012

Mehrholz J, 2010, COCHRANE DB SYST REV, V1

Mirelman A, 2013, MOVEMENT DISORD, V28, P1597, DOI 10.1002/mds.25670

Mirelman A, 2013, BMC NEUROL, V13, DOI 10.1186/1471-2377-13-15

Moisello C, 2015, BRAIN STIMUL, V8, P224, DOI 10.1016/j.brs.2014.11.005

Monteiro RS, 2015, MED HYPOTHESES, V85, P537, DOI 10.1016/j.mehy.2015.07.011

Monticone M, 2015, MOVEMENT DISORD, V30, P1050, DOI 10.1002/mds.26256

Nieuwboer A, 2007, J NEUROL NEUROSUR PS, V78, P134, DOI 10.1136/jnnp.200X.097923

Nieuwboer A, 2009, PARKINSONISM RELAT D, V15, pS53, DOI 10.1016/S1353-8020(09)70781-3

Petzinger GM, 2013, LANCET NEUROL, V12, P716, DOI 10.1016/S1474-4422(13)70123-6

Picelli A, 2014, J NEUROENG REHABIL, V11, DOI 10.1186/1743-0003-11-28

Reis J, 2011, CURR OPIN NEUROL, V24, P590, DOI 10.1097/WCO.0b013e32834c3db0

Sehm B, 2014, NEUROBIOL AGING, V35, P232, DOI 10.1016/j.neurobiolaging.2013.06.021

Tomlinson C. L, 2012, COCHRANE DB SYST REV, V8

Tomlinson CL, 2014, COCHRANE DB SYST REV, DOI 10.1002/14651858.CD002815.pub2

Winward C, 2012, MOVEMENT DISORD, V27, P143, DOI 10.1002/mds.23966

Yang F, 2015, BRAIN, V138, P269, DOI 10.1093/brain/awu323

NR 30

TC 163

Z9 179

U1 11

U2 153

PU ELSEVIER SCI LTD

PI OXFORD

PA THE BOULEVARD, LANGFORD LANE, KIDLINGTON, OXFORD OX5 1GB, OXON, ENGLAND

SN 1353-8020

EI 1873-5126

J9 PARKINSONISM RELAT D

JI Parkinsonism Relat. Disord.

PD JAN

PY 2016

VL 22

SU 1

BP S60

EP S64

DI 10.1016/j.parkreldis.2015.09.005

PG 5

WC Clinical Neurology

WE Science Citation Index Expanded (SCI-EXPANDED); Conference Proceedings Citation Index - Science (CPCI-S)

SC Neurosciences & Neurology

GA CZ0GA

UT WOS:000366781900014

PM 26360239

DA 2022-06-21

ER

PT J

AU Abd El-Kafy, EM

Alshehri, MA

El-Fiky, AAR

Guermazi, MA

AF Abd El-Kafy, Ehab Mohamed

Alshehri, Mansour Abdullah

El-Fiky, Amir Abdel-Raouf

Guermazi, Mohamad Abdelhamid

TI The Effect of Virtual Reality-Based Therapy on Improving Upper Limb

Functions in Individuals With Stroke: A Randomized Control Trial

SO FRONTIERS IN AGING NEUROSCIENCE

LA English

DT Article

DE virtual reality; exergames; physiotherapy; upper limb (UL); stroke

ID MOTOR FUNCTION-TEST; UPPER EXTREMITY FUNCTION; CEREBRAL-PALSY;

REHABILITATION; RECOVERY; ENVIRONMENT; RELIABILITY; CHILDREN; CARE; TASK

AB Background: Stroke is a common cause of motor disability. The recovery of upper limb after stroke is poor, with few stroke survivors regaining some functional use of the affected upper limb. This is further complicated by the fact that the prolonged rehabilitation is accompanied by multiple challenges in using and identifying meaningful and motivated treatment tasks that may be adapted and graded to facilitate the rehabilitation program. Virtual reality-based therapy is one of the most innovative approaches in rehabilitation technology and virtual reality systems can provide enhanced feedback to promote motor learning in individuals with neurological or musculoskeletal diseases.Purpose: This study investigated the effect of virtual reality-based therapy on improving upper limb functions in individuals with chronic stroke.Methods: Forty Saudi individuals with chronic stroke (6-24 months following stroke incidence) and degree of spasticity ranged between 1, 1 + and 2 according to Modified Ashworth Scale were included in this study. Participants were randomly assigned into two groups, experimental and control, with the experimental group undertaking a conventional 1-h functional training program, followed by another hour of virtual reality-based therapy using Armeo Spring equipment and the control group received 2 h of a conventional functional training program. The treatment program was conducted three times per week for three successive months. The change in the scores of Action Research Arm Test (ARAT), Wolf Motor Function Test (WMFT), WMFT-Time (time required to complete the test) and Hand Grip Strength (HGS) were recorded at baseline and after completion of the treatment. Parametric (paired and unpaired t-tests) non-parametric (Wilcoxon and Mann-Whitney tests) statistical tests were used to identify the differences within and between groups (experimental group and control group) and evaluation times (pre- and immediately post-treatment).Results: Both groups showed significant differences (all, P < 0.05) in all measured variables after 3 months of the treatment. Individuals with stoke in the experimental group had a better improvement in ARAT (P < 0.01), WMFT (P < 0.01) and WMFT-Time (P < 0.01) scores after completion of the treatment compared to the control group. No significant difference in HGS scores was detected between groups after completion of the treatment (P = 0.252).Conclusion: The use of combined treatment of virtual reality-based therapy and conventional functional training program is more effective for improving upper limb functions in individuals with chronic stroke than the use of the conventional program alone.

C1 [Abd El-Kafy, Ehab Mohamed; Alshehri, Mansour Abdullah; El-Fiky, Amir Abdel-Raouf] Umm Al Qura Univ, Fac Appl Med Sci, Dept Physiotherapy, Mecca, Saudi Arabia.

[Alshehri, Mansour Abdullah] Univ Queensland, Sch Hlth & Rehabil Sci, Brisbane, Qld, Australia.

[Guermazi, Mohamad Abdelhamid] King Abdul Aziz Hosp, Dept Rheumatol, Jeddah, Saudi Arabia.

RP Abd El-Kafy, EM; Alshehri, MA (通讯作者)，Umm Al Qura Univ, Fac Appl Med Sci, Dept Physiotherapy, Mecca, Saudi Arabia.; Alshehri, MA (通讯作者)，Univ Queensland, Sch Hlth & Rehabil Sci, Brisbane, Qld, Australia.

EM emkafy@uqu.edu.sa; mamshehri@uqu.edu.sa

RI Kafy, Ehab Mohamed Abd El/C-2973-2019; Alshehri, Mansour

Abdullah/S-4862-2016

OI Alshehri, Mansour Abdullah/0000-0002-0294-9856; Abd El Kafy,

Ehab/0000-0003-3632-9548

FU National Science, Technology and Innovation Plan (MAARIFAH)

[14-MED998-10]; King Abdulaziz City for Science and Technology; Kingdom

of Saudi Arabia

FX This study was funded (award number: 14-MED998-10) by the National Plan

for Science, Technology and Innovation (MAARIFAH), King Abdulaziz City

for Science and Technology, the Kingdom of Saudi Arabia.

CR Ada L., 2005, SCI BASED REHABILITA, P87, DOI [10.1016/B978-0-7506-5564-4.50008-5, DOI 10.1016/B978-0-7506-5564-4.50008-5]

Al Khathaami AM, 2011, INT J STROKE, V6, P75, DOI 10.1111/j.1747-4949.2010.00542.x

Alahmari K, 2016, MEDITERR J SOC SCI, V7, P228, DOI [10.5901/mjss.2016.v7n1s1p228, DOI 10.5901/MJSS.2016.V7N1S1P228]

Basteris A, 2014, J NEUROENG REHABIL, V11, DOI 10.1186/1743-0003-11-111

Beer RF, 2007, MUSCLE NERVE, V36, P242, DOI 10.1002/mus.20817

BOHANNON RW, 1987, PHYS THER, V67, P206, DOI 10.1093/ptj/67.2.206

BOTTE MJ, 1988, CLIN ORTHOP RELAT R, P7

Brown JK, 2000, CONGENITAL HEMIPLEGIA, P113

Carpinella I, 2014, J NEUROENG REHABIL, V11, DOI 10.1186/1743-0003-11-67

Chin LF, 2020, TOP STROKE REHABIL, V27, P224, DOI 10.1080/10749357.2019.1690796

Crosbie JH, 2007, DISABIL REHABIL, V29, P1139, DOI 10.1080/09638280600960909

Dandekar S.P., 2019, INT J RES MED SCI, V7, P3224, DOI [10.18203/2320-6012.ijrms20193425, DOI 10.18203/2320-6012.IJRMS20193425]

Dobkin BH, 2004, LANCET NEUROL, V3, P528, DOI 10.1016/S1474-4422(04)00851-8

Duff SV, 2003, DEV MED CHILD NEUROL, V45, P746, DOI 10.1017/S00121622030012397

Feys HM, 1998, STROKE, V29, P785, DOI 10.1161/01.STR.29.4.785

Gordon AM, 2003, DEV MED CHILD NEUROL, V45, P240, DOI 10.1017/S0012162203000471

Guy S., 2004, INTERDISCIPLINARY TE, P23, DOI [10.3109/9780203640364-3, DOI 10.3109/9780203640364-3]

Hodics TM, 2012, ARCH PHYS MED REHAB, V93, P1963, DOI 10.1016/j.apmr.2012.05.002

Holden MK, 2007, IEEE T NEUR SYS REH, V15, P36, DOI 10.1109/TNSRE.2007.891388

Hung YC, 2004, DEV MED CHILD NEUROL, V46, P746, DOI 10.1017/S0012162204001288

Kim WS, 2020, J CLIN MED, V9, DOI 10.3390/jcm9103369

Kim WS, 2018, MEDICINE, V97, DOI 10.1097/MD.0000000000011173

Kwakkel G, 2004, STROKE, V35, P2529, DOI 10.1161/01.STR.0000143153.76460.7d

Kwakkel G, 2008, NEUROREHAB NEURAL RE, V22, P111, DOI 10.1177/1545968307305457

Langhorne P, 2011, LANCET, V377, P1693, DOI 10.1016/S0140-6736(11)60325-5

Langhorne P, 2009, LANCET NEUROL, V8, P741, DOI 10.1016/S1474-4422(09)70150-4

Levac DE, 2019, J NEUROENG REHABIL, V16, DOI 10.1186/s12984-019-0587-8

LYLE RC, 1981, INT J REHABIL RES, V4, P483, DOI 10.1097/00004356-198112000-00001

Mayo NE, 1999, DISABIL REHABIL, V21, P258

Merians AS, 2006, NEUROREHAB NEURAL RE, V20, P252, DOI 10.1177/1545968306286914

Merians AS, 2002, PHYS THER, V82, P898, DOI 10.1093/ptj/82.9.898

Mihelj M, 2012, PRESENCE-TELEOP VIRT, V21, P1, DOI 10.1162/PRES_a_00078

Morris DM, 2001, ARCH PHYS MED REHAB, V82, P750, DOI 10.1053/apmr.2001.23183

Nijland R, 2010, J REHABIL MED, V42, P694, DOI 10.2340/16501977-0560

Nomikos Polykarpos Angelos, 2018, J Phys Ther Sci, V30, P1271, DOI 10.1589/jpts.30.1271

Pangman VC, 2000, APPL NURS RES, V13, P209, DOI 10.1053/apnr.2000.9231

Piron L, 2005, PRESENCE-TELEOP VIRT, V14, P732, DOI 10.1162/105474605775196580

Qureshi S., 2008, SAUDI STROKE ASS PRO, DOI [10.1111/j.1747-4949.2008.00207.x, DOI 10.1111/J.1747-4949.2008.00207.X]

Sabut SK, 2010, DISABIL REHABIL, V32, P1594, DOI 10.3109/09638281003599596

Sacco RL, 2013, STROKE, V44, P2064, DOI 10.1161/STR.0b013e318296aeca

Sakzewski L, 2009, PEDIATRICS, V123, pE1111, DOI 10.1542/peds.2008-3335

Sisto Sue Ann, 2002, Top Stroke Rehabil, V8, P11

Skold A, 2004, AM J OCCUP THER, V58, P416, DOI 10.5014/ajot.58.4.416

Sloan R L, 1992, Int J Rehabil Res, V15, P158, DOI 10.1097/00004356-199206000-00009

Song CS, 2012, J PHYS THER SCI, V24, P1355, DOI 10.1589/jpts.24.1355

Spence N, 2020, J MUSCULOSKEL NEURON, V20, P480

Steenbergen B, 2004, DEV MED CHILD NEUROL, V46, P325, DOI 10.1017/S0012162204000532

Taveggia G, 2016, EUR J PHYS REHAB MED, V52, P767

Timmermans AAA, 2009, J NEUROENG REHABIL, V6, DOI 10.1186/1743-0003-6-1

Walker M., 2000, BRIT J OCCUP THER, V63, P367, DOI [10.1177/030802260006300803, DOI 10.1177/030802260006300803]

WIKLUND LM, 1991, DEV MED CHILD NEUROL, V33, P512

Wolf SL, 2001, STROKE, V32, P1635, DOI 10.1161/01.STR.32.7.1635

NR 52

TC 1

Z9 1

U1 14

U2 17

PU FRONTIERS MEDIA SA

PI LAUSANNE

PA AVENUE DU TRIBUNAL FEDERAL 34, LAUSANNE, CH-1015, SWITZERLAND

SN 1663-4365

J9 FRONT AGING NEUROSCI

JI Front. Aging Neurosci.

PD NOV 2

PY 2021

VL 13

AR 731343

DI 10.3389/fnagi.2021.731343

PG 8

WC Geriatrics & Gerontology; Neurosciences

WE Science Citation Index Expanded (SCI-EXPANDED); Social Science Citation Index (SSCI)

SC Geriatrics & Gerontology; Neurosciences & Neurology

GA WZ1BX

UT WOS:000719709400001

PM 34795574

OA gold, Green Published

DA 2022-06-21

ER

PT J

AU Abou, L

Malala, VD

Yarnot, R

Alluri, A

Rice, LA

AF Abou, Libak

Malala, Vonjiniaina Domohina

Yarnot, Rebecca

Alluri, Aditya

Rice, Laura A.

TI Effects of Virtual Reality Therapy on Gait and Balance Among Individuals

With Spinal Cord Injury: A Systematic Review and Meta-analysis

SO NEUROREHABILITATION AND NEURAL REPAIR

LA English

DT Review

DE spinal cord injuries; virtual reality; gait; postural balance;

systematic review

ID STANDING BALANCE; REHABILITATION; PRIORITIES; RECOVERY; ABILITY; PEOPLE;

STROKE

AB Background and Purpose. The use of virtual reality (VR) therapy among individuals with spinal cord injury (SCI) is a relatively new rehabilitation approach replicating real-life scenarios. The aim of this study was to evaluate the effectiveness of VR therapy for improving gait and balance in individuals with SCI. Methods. Databases of PubMed, Web of Science, Scopus, SportDiscuss, and CINHAL were searched from inception until September 2019. Two independent reviewers screened articles for inclusion, extracted data, and evaluated methodological quality of the trials. Results. Ten trials, including 3 randomized clinical trials (RCTs) and 7 pre-post trials, with a total of 149 participants were analyzed. Meta-analysis of RCTs demonstrated significant improvement in sitting balance (standardized mean difference [SMD] = 1.65; 95% CI 1.21-2.09; P < .01) after VR therapy with conventional rehabilitation compared with only conventional rehabilitation. Also, pre-post studies using VR therapy without a control group showed improvement in standing balance (Berg Balance Scale, MD = 4.22; 95% CI 1.78-6.66; P < .01 and Activities-specific Balance Confidence scale, MD = 8.53; 95% CI 2.52-14.53; P = .01) and a trend for improvement in gait (SMD = 0.34; 95% CI 0.02-0.66; P = .04). Conclusion. This study demonstrated the beneficial effects of VR therapy to enhance sitting and standing balance and showed a trend of gait improvement in individuals with SCI. This conclusion is based on mainly preliminary data and therefore, more RCTs are needed to confirm the effects of the use of VR in individuals with SCI.

C1 [Abou, Libak; Yarnot, Rebecca; Rice, Laura A.] Univ Illinois, Dept Kinesiol & Community Hlth, Urbana, IL USA.

[Malala, Vonjiniaina Domohina] Univ Paris Sud, UFR STAPS, Bures Sur Yvette, Ile De France, France.

[Alluri, Aditya] Univ Illinois, Dept Mol & Cellular Biol, Urbana, IL USA.

RP Rice, LA (通讯作者)，Univ Illinois, Coll Appl Hlth Sci, Dept Kinesiol & Community Hlth, 219 Freer Hall,906 South Goodwin Ave, Urbana, IL 61801 USA.

EM ricela@illinois.edu

RI Abou, Libak/AAH-4763-2020

OI Abou, Libak/0000-0001-6404-7623

CR Abou L, 2019, SPINAL CORD, V57, P1001, DOI 10.1038/s41393-019-0346-5

An CM, 2018, J SPINAL CORD MED, V41, P223, DOI 10.1080/10790268.2017.1369217

Anderson KD, 2004, J NEUROTRAUM, V21, P1371, DOI 10.1089/neu.2004.21.1371

[Anonymous], 2014, REV MAN REVMAN COMP

CHEN L, 2016, BIOMED RES INT, V2016, DOI DOI 10.1155/2016/7309272

Cuijpers P, 2017, EPIDEMIOL PSYCH SCI, V26, P364, DOI 10.1017/S2045796016000809

D'Addio G, 2014, IEEE INT SYM MED MEA, P555

de Rooij IJM, 2016, PHYS THER, V96, P1905, DOI 10.2522/ptj.20160054

Dunsky A, 2017, BIOMED RES INT, V2017, DOI 10.1155/2017/6987017

Fizzotti G, 2015, STUD HEALTH TECHNOL, V210, P479, DOI 10.3233/978-1-61499-512-8-479

Higgins JPT, 2002, STAT MED, V21, P1539, DOI 10.1002/sim.1186

Higgins JPT., 2011, COCHRANE HDB SYSTEMA

Casuso-Holgado MJ, 2018, CLIN REHABIL, V32, P1220, DOI 10.1177/0269215518768084

Keshner Emily A, 2004, J Neuroeng Rehabil, V1, P8, DOI 10.1186/1743-0003-1-8

Khurana M, 2017, TOP SPINAL CORD INJ, V23, P263, DOI 10.1310/sci16-00003

de Araujo AVL, 2019, BIOMED RES INT, V2019, DOI 10.1155/2019/7106951

Levin MF, 2015, PHYS THER, V95, P415, DOI 10.2522/ptj.20130579

Li Z, 2016, CLIN REHABIL, V30, P432, DOI 10.1177/0269215515593611

Lo C, 2016, J NEUROTRAUM, V33, P1958, DOI 10.1089/neu.2016.4423

Moher D, 2009, PLOS MED, V6, DOI [DOI 10.1136/BMJ.B2535, 10.1371/journal.pmed.1000097]

Moore JL, 2018, J NEUROL PHYS THER, V42, P174, DOI 10.1097/NPT.0000000000000229

National Institute of Health, STUD QUAL ASS TOOLS

Nijendijk JHB, 2014, SPINAL CORD, V52, P258, DOI 10.1038/sc.2013.180

Porras DC, 2018, NEUROLOGY, V90, P1017, DOI 10.1212/WNL.0000000000005603

Quinzanos-Fresnedo J, 2018, J SPINAL CORD MED, DOI 10.1080/10790268.2018.1518124

Richard-Denis A, 2020, J SPINAL CORD MED, V43, P241, DOI 10.1080/10790268.2018.1517138

Santos P, 2019, NEUROREHABILITATION, V44, P569, DOI 10.3233/NRE-192700

Sayenko DG, 2010, SPINAL CORD, V48, P886, DOI 10.1038/sc.2010.41

Scivoletto G, 2014, FRONT HUM NEUROSCI, V8, DOI 10.3389/fnhum.2014.00141

Tak S, 2015, MED SCI TECHNOL, V56, P53, DOI [10.12659/MST.894514, DOI 10.12659/MST.894514]

Truchon C, 2017, J NEUROTRAUM, V34, P2901, DOI 10.1089/neu.2016.4932

van Dijsseldonk RB, 2018, FRONT NEUROL, V9, DOI 10.3389/fneur.2018.00963

Villiger M, 2017, FRONT NEUROL, V8, DOI 10.3389/fneur.2017.00635

Villiger M, 2013, NEUROREHAB NEURAL RE, V27, P675, DOI 10.1177/1545968313490999

Wall T, 2015, J SPINAL CORD MED, V38, P777, DOI 10.1179/2045772314Y.0000000296

Wang B, 2019, CLIN REHABIL, V33, P1130, DOI 10.1177/0269215519843174

Yang JF, 2014, NEUROREHAB NEURAL RE, V28, P314, DOI 10.1177/1545968313508473

Yeo Elizabeth, 2019, Innov Clin Neurosci, V16, P13

NR 38

TC 8

Z9 8

U1 2

U2 18

PU SAGE PUBLICATIONS INC

PI THOUSAND OAKS

PA 2455 TELLER RD, THOUSAND OAKS, CA 91320 USA

SN 1545-9683

EI 1552-6844

J9 NEUROREHAB NEURAL RE

JI Neurorehabil. Neural Repair

PD MAY

PY 2020

VL 34

IS 5

BP 375

EP 388

AR 1545968320913515

DI 10.1177/1545968320913515

EA APR 2020

PG 14

WC Clinical Neurology; Rehabilitation

WE Science Citation Index Expanded (SCI-EXPANDED)

SC Neurosciences & Neurology; Rehabilitation

GA LM6AH

UT WOS:000527683200001

PM 32270736

OA Bronze

DA 2022-06-21

ER

PT J

AU Aburub, AS

Lamontagne, A

AF Aburub, Ala' S.

Lamontagne, Anouk

TI Altered steering strategies for goal-directed locomotion in stroke

SO JOURNAL OF NEUROENGINEERING AND REHABILITATION

LA English

DT Article

DE Heading; Hemiparesis; Optic flow; Virtual reality; Visuomotor; Walking

ID OPTIC-FLOW; WALKING; MOTION; NEGLECT; PERCEPTION; BEHAVIOR; FALLS; HEAD

AB Background: Individuals who have sustained a stroke can manifest altered locomotor steering behaviors when exposed to optic flows expanding from different locations. Whether these alterations persist in the presence of a visible goal and whether they can be explained by the presence of a perceptuo-motor disorder remain unknown. The purpose of this study was to compare stroke participants and healthy participants on their ability to control heading while exposed to changing optic flows and target locations.

Methods: Ten participants with stroke (55.6 +/- 9.3 yrs) and ten healthy controls (57.0 +/- 11.5 yrs) participated in a mouse-driven steering task (perceptuo-motor task) while seated and in a walking steering task. In the seated steering task, participants were instructed to head or 'walk' toward a target in the virtual environment by using a mouse while wearing a helmet-mounted display (HMD). In the walking task, participants performed a similar steering task in the same virtual environment while walking overground at their comfortable speed. For both experiments, the target and/or the focus of expansion (FOE) of the optic flow shifted to the side (+/-20 degrees) or remained centered. The main outcome measure was net heading errors (NHE). Secondary outcomes included mediolateral displacement, horizontal head orientation, and onsets of heading and head reorientation.

Results: In the walking steering task, the presence of FOE shifts modulated the extent and timing of mediolateral displacement and head rotation changes, as well as NHE magnitudes. Participants overshot and undershot their net heading, respectively, in response to ipsilateral and contralateral FOE and target shifts. Stroke participants made larger NHEs, especially when the FOE was shifted towards the non-paretic side. In the seated steering task, similar NHEs were observed between stroke and healthy participants.

Conclusions: The findings highlight the fine coordination between rotational and translational steering mechanisms in presence of targets and FOE shifts. The altered performance of stroke participants in walking but not in the seated steering task suggests that an altered perceptuo-motor processing of optic flow is not a main contributing factor and that other stroke-related sensorimotor deficits are involved.

C1 [Aburub, Ala' S.] McGill Univ, Sch Phys & Occupat Therapy, Montreal, PQ, Canada.

[Lamontagne, Anouk] Ctr Interdisciplinary Res Rehabil Greater Montrea, Jewish Rehabil Hosp Res Site, Montreal, PQ, Canada.

RP Lamontagne, A (通讯作者)，Ctr Interdisciplinary Res Rehabil Greater Montrea, Jewish Rehabil Hosp Res Site, Montreal, PQ, Canada.

EM Anouk.lamontagne@mcgill.ca

RI Aburub, Ala'/AAH-8173-2020

OI Aburub, Ala' S./0000-0003-3573-7304; Lamontagne,

Anouk/0000-0003-2033-9063

FU Canadian Institutes of Health Research (CIHR) [MOP-77548]; Canada

Foundation for Innovation; Foundation of the Jewish Rehabilitation

Hospital

FX The authors would like to thank all participants, as well as Andrei

Garcia Popov, Christian Beaudoin and Valeri Goussev for their technical

assistance. This study was funded by the Canadian Institutes of Health

Research (CIHR, MOP-77548), the Canada Foundation for Innovation and the

Foundation of the Jewish Rehabilitation Hospital.

CR Berard J, 2012, CLIN NEUROPHYSIOL, V123, P1422, DOI 10.1016/j.clinph.2011.11.081

Berti A, 2002, NEUROPSYCHOLOGY, V16, P390, DOI 10.1037//0894-4105.16.3.390

Billino J, 2009, NEUROPSYCHOLOGIA, V47, P2133, DOI 10.1016/j.neuropsychologia.2009.04.005

Dean CM, 2001, CLIN REHABIL, V15, P415, DOI 10.1191/026921501678310216

Garcia-Popov A, 2011, ZUR INT C VIRT REH

GIBSON JJ, 1994, PSYCHOL REV, V101, P318, DOI 10.1037/0033-295X.101.2.318

Grasso R, 1996, NEUROREPORT, V7, P1170, DOI 10.1097/00001756-199604260-00015

Hollands MA, 2002, EXP BRAIN RES, V143, P221, DOI 10.1007/s00221-001-0983-7

Huitema RB, 2006, GAIT POSTURE, V23, P200, DOI 10.1016/j.gaitpost.2005.02.003

Kim J, 1999, VISION RES, V39, P3175, DOI 10.1016/S0042-6989(99)00024-3

Lamontagne A, 2010, NEUROREHAB NEURAL RE, V24, P457, DOI 10.1177/1545968309355985

Lamontagne A, 2009, NEUROREHAB NEURAL RE, V23, P256, DOI 10.1177/1545968308324549

Langhorne P, 2000, STROKE, V31, P1223, DOI 10.1161/01.STR.31.6.1223

LIPSITZ LA, 1991, J GERONTOL, V46, pM114, DOI 10.1093/geronj/46.4.M114

NYBERG L, 1995, STROKE, V26, P838, DOI 10.1161/01.STR.26.5.838

Patla AE, 1999, EXP BRAIN RES, V129, P629, DOI 10.1007/s002210050932

Reed-Jones R, 2009, EXP BRAIN RES, V197, P357, DOI 10.1007/s00221-009-1923-1

ROBERTSON IH, 1994, NEUROPSYCHOLOGIA, V32, P1495, DOI 10.1016/0028-3932(94)90121-X

Rushton SK, 1998, CURR BIOL, V8, P1191, DOI 10.1016/S0960-9822(07)00492-7

Sarre G, 2008, NEUROSCI LETT, V436, P96, DOI 10.1016/j.neulet.2008.02.049

Turano KA, 2005, VISION RES, V45, P3117, DOI 10.1016/j.visres.2005.06.017

Vaina LM, 2010, J NEUROPSYCHOL, V4, P121, DOI 10.1348/174866409X471760

VONSCHROEDER HP, 1995, J REHABIL RES DEV, V32, P25

Warren WH, 2001, NAT NEUROSCI, V4, P213, DOI 10.1038/84054

NR 24

TC 5

Z9 5

U1 0

U2 6

PU BMC

PI LONDON

PA CAMPUS, 4 CRINAN ST, LONDON N1 9XW, ENGLAND

EI 1743-0003

J9 J NEUROENG REHABIL

JI J. NeuroEng. Rehabil.

PD JUL 22

PY 2013

VL 10

AR 80

DI 10.1186/1743-0003-10-80

PG 11

WC Engineering, Biomedical; Neurosciences; Rehabilitation

WE Science Citation Index Expanded (SCI-EXPANDED)

SC Engineering; Neurosciences & Neurology; Rehabilitation

GA 194WK

UT WOS:000322663200001

PM 23875969

OA Green Published, gold

DA 2022-06-21

ER

PT J

AU Acar, G

Altun, GP

Yurdalan, S

Polat, MG

AF Acar, Gonul

Altun, Gamze Polen

Yurdalan, SaadetUfuk

Polat, Mine Gulden

TI Efficacy of neurodevelopmental treatment combined with the Nintendo (R)

Wii in patients with cerebral palsy

SO JOURNAL OF PHYSICAL THERAPY SCIENCE

LA English

DT Article

DE Cerebral palsy; Virtual reality; Nintendo (R) Wii

ID FUNCTIONAL INDEPENDENCE MEASURE; UPPER EXTREMITY FUNCTION;

VIRTUAL-REALITY; MANUAL ABILITY; HAND FUNCTION; LOW-COST; CHILDREN;

PROGRAM; THERAPY; REHABILITATION

AB [Purpose] The aim of this study was to investigate the efficiency of Nintendo (R) Wii games in addition to neurodevelopmental treatment in patients with cerebral palsy. [Subjects and Methods] Thirty hemiparetic cerebral palsy patients (16 females, 14 males; mean age, 6-15 years) were included in the study and divided into two groups: a neurodevelopmental treatment+Nintendo Wii group (group 1, n=15) and a neurodevelopmental treatment group (group 2, n=15). Both groups received treatment in 45-minute sessions 2 days/week for six weeks. Use of the upper extremities, speed, disability and functional independence were evaluated using the Quality of Upper Extremity Skills Test, Jebsen Taylor Hand Function Test, ABILHAND-Kids test, and Pediatric Functional Independence Measure (self-care) before and after treatment. [Results] There were statistically significant improvements in all parameters for group 1 and group 2 (except quality of function) after six weeks of treatment. Intergroup analysis showed that group 1 was superior to group 2 in mean change differences in the Jebsen Taylor Hand Function Test. [Conclusion] Our results showed that neurodevelopmental treatment is effective for improving hand functions in hemiplegic cerebral palsy. To provide a enjoyable, motivational, safe, and effective rehabilitation program, the Nintendo (R) Wii may be used in addition to neurodevelopmental treatment.

C1 [Acar, Gonul; Yurdalan, SaadetUfuk; Polat, Mine Gulden] Marmara Univ, Fac Hlth Sci, Dept Physiotherapy & Rehabil, E-5 Yanyol Uzeri, TR-34865 Istanbul, Turkey.

[Altun, Gamze Polen] TEMAS Pediat Therapy & Special Educ Ctr, Istanbul, Turkey.

RP Acar, G (通讯作者)，Marmara Univ, Fac Hlth Sci, Dept Physiotherapy & Rehabil, E-5 Yanyol Uzeri, TR-34865 Istanbul, Turkey.

EM gonulacar34@gmail.com

RI Polat, Mine Gulden/X-4812-2019

OI Polat, Mine Gulden/0000-0002-9705-9740; , Gulden/0000-0003-1485-5117

CR Abdel Rahman S., 2011, MIDDLE E J SCI RES, V7, P63

AlSaif AA, 2015, J PHYS THER SCI, V27, P2001, DOI 10.1589/jpts.27.2001

Arner M, 2008, J HAND SURG-AM, V33A, P1337, DOI 10.1016/j.jhsa.2008.02.032

Arnould C, 2004, NEUROLOGY, V63, P1045, DOI 10.1212/01.WNL.0000138423.77640.37

Basu AP, 2015, FRONT NEUROL, V5, DOI 10.3389/fneur.2014.00281

Bobath B, 1984, CLIN DEV MED, V90, P6

Burdea GC, 2011, IEEE ENG MED BIO, P1835, DOI 10.1109/IEMBS.2011.6090522

Cans C, 2000, DEV MED CHILD NEUROL, V42, P816, DOI 10.1111/j.1469-8749.2000.tb00695.x

Chen YP, 2007, PHYS THER, V87, P1441, DOI 10.2522/ptj.20060062

Chiu HC, 2014, CLIN REHABIL, V28, P1015, DOI 10.1177/0269215514533709

Dematteo Carol, 1993, Physical and Occupational Therapy in Pediatrics, V13, P1, DOI 10.1300/J006v13n02_01

Deutsch JE, 2008, PHYS THER, V88, P1196, DOI 10.2522/ptj.20080062

Eliasson AC, 2006, DEV MED CHILD NEUROL, V48, P549, DOI 10.1017/S0012162206001162

Exner CE., 2006, HAND FUNCTION CHILD, V2nd ed, P239

Farr W, 2013, ARCH DIS CHILD, V98, pA97

Himmelmann K, 2013, HAND CLINIC, V111, P163, DOI 10.1016/B978-0-444-52891-9.00015-4

Jannink MJA, 2008, CYBERPSYCHOL BEHAV, V11, P27, DOI 10.1089/cpb.2007.0014

JEBSEN R H, 1969, Archives of Physical Medicine and Rehabilitation, V50, P311

Kerem Gunel M, VIRTUAL REALITY REHA

Law M, 1997, DEV MED CHILD NEUROL, V39, P664

Lee KH, 2015, J PHYS THER SCI, V27, P1637, DOI 10.1589/jpts.27.1637

Lotze M, 2003, BRAIN, V126, P866, DOI 10.1093/brain/awg079

Luna-Oliva L, 2013, NEUROREHABILITATION, V33, P513, DOI 10.3233/NRE-131001

MAYSTON MJ, 1992, MED SPORT SCI, V36, P1

MSALL ME, 1994, CLIN PEDIATR, V33, P421, DOI 10.1177/000992289403300708

Ottenbacher KJ, 1996, DEV MED CHILD NEUROL, V38, P907

Reid D, 2006, THER RECREAT J, V40, P255

REID DT, 2002, TECHNOL DISABIL, V14, P53

Shin JW, 2015, J PHYS THER SCI, V27, P2151, DOI 10.1589/jpts.27.2151

Shumway-Cook A., 2001, MOTOR CONTROL THEORY, V2nd ed.

Tarakci D, 2013, J PHYS THER SCI, V25, P1123, DOI 10.1589/jpts.25.1123

TAYLOR N, 1973, ARCH PHYS MED REHAB, V54, P129

Tsorlakis N, 2004, DEV MED CHILD NEUROL, V46, P740, DOI 10.1017/S0012162204001276

Winkels DGM, 2013, DEV NEUROREHABIL, V16, P44, DOI 10.3109/17518423.2012.713401

You SH, 2005, DEV MED CHILD NEUROL, V47, P628, DOI 10.1017/S0012162205001234

Zoccolillo L, 2015, EUR J PHYS REHABIL M

NR 36

TC 19

Z9 20

U1 6

U2 28

PU SOC PHYSICAL THERAPY SCIENCE

PI TOKYO

PA C/O PUBLICATION CENTER, 1-24-12 SUGAMO, TOSHIMA-KU, TOKYO, 170-0002,

JAPAN

SN 0915-5287

EI 2187-5626

J9 J PHYS THER SCI

JI J. Phys. Ther. Sci.

PD MAR

PY 2016

VL 28

IS 3

BP 774

EP 780

DI 10.1589/jpts.28.774

PG 7

WC Rehabilitation

WE Science Citation Index Expanded (SCI-EXPANDED)

SC Rehabilitation

GA DP9ZB

UT WOS:000378855600012

PM 27134357

OA Green Submitted, Green Published, gold

DA 2022-06-21

ER

PT J

AU Achanccaray, D

Izumi, SI

Hayashibe, M

AF Achanccaray, David

Izumi, Shin-Ichi

Hayashibe, Mitsuhiro

TI Visual-Electrotactile Stimulation Feedback to Improve Immersive

Brain-Computer Interface Based on Hand Motor Imagery

SO COMPUTATIONAL INTELLIGENCE AND NEUROSCIENCE

LA English

DT Article

ID VIRTUAL-REALITY; CORTICAL ACTIVITY; SPATIAL FILTERS; BCI; STROKE;

CLASSIFICATION; DESYNCHRONIZATION; COMMUNICATION; PERFORMANCE; SYSTEM

AB In the aging society, the number of people suffering from vascular disorders is rapidly increasing and has become a social problem. The death rate due to stroke, which is the second leading cause of global mortality, has increased by 40% in the last two decades. Stroke can also cause paralysis. Of late, brain-computer interfaces (BCIs) have been garnering attention in the rehabilitation field as assistive technology. A BCI for the motor rehabilitation of patients with paralysis promotes neural plasticity, when subjects perform motor imagery (MI). Feedback, such as visual and proprioceptive, influences brain rhythm modulation to contribute to MI learning and motor function restoration. Also, virtual reality (VR) can provide powerful graphical options to enhance feedback visualization. This work aimed to improve immersive VR-BCI based on hand MI, using visual-electrotactile stimulation feedback instead of visual feedback. The MI tasks include grasping, flexion/extension, and their random combination. Moreover, the subjects answered a system perception questionnaire after the experiments. The proposed system was evaluated with twenty ablebodied subjects. Visual-electrotactile feedback improved the mean classification accuracy for the grasping (93.00% +/- 3.50%) and flexion/extension (95.00% +/- 5.27%) MI tasks. Additionally, the subjects achieved an acceptable mean classification accuracy (maximum of 86.5% +/- 5.80%) for the random MI task, which required more concentration. The proprioceptive feedback maintained lower mean power spectral density in all channels and higher attention levels than those of visual feedback during the test trials for the grasping and flexion/extension MI tasks. Also, this feedback generated greater relative power in the mu-b an d for the premotor cortex, which indicated better MI preparation. Thus, electrotactile stimulation along with visual feedback enhanced the immersive VR-BCI classification accuracy by 5.5% and 4.5% for the grasping and flexion/extension MI tasks, respectively, retained the subject's attention, and eased MI better than visual feedback alone.

C1 [Achanccaray, David; Hayashibe, Mitsuhiro] Tohoku Univ, Dept Robot, Neuro Robot Lab, Sendai, Miyagi 9808579, Japan.

[Izumi, Shin-Ichi] Tohoku Univ, Dept Phys Med & Rehabil, Grad Sch Med, Sendai, Miyagi 9808575, Japan.

[Izumi, Shin-Ichi] Tohoku Univ, Grad Sch Biomed Engn, Sendai, Miyagi 9808574, Japan.

RP Achanccaray, D (通讯作者)，Tohoku Univ, Dept Robot, Neuro Robot Lab, Sendai, Miyagi 9808579, Japan.

EM david.ad@dc.tohoku.ac.jp

RI Hayashibe, Mitsuhiro/B-8170-2009

OI Hayashibe, Mitsuhiro/0000-0001-6179-5706; Achanccaray,

David/0000-0002-7309-7988; Izumi, Shin-Ichi/0000-0001-9035-2808

FU FONDECYT from CONCYTEC, Peru [112-2017]; Japan Society for the Promotion

of Science [18H01399]

FX This work was supported in part by FONDECYT from CONCYTEC, Peru, under

Contract 112-2017, and in part by the Japan Society for the Promotion of

Science Grant-in-Aid for Scientific Research (B) under Project 18H01399.

CR Aldea R, 2013, 2013 INTERNATIONAL SYMPOSIUM ON SIGNALS, CIRCUITS AND SYSTEMS (ISSCS)

Alimardani M., 2018, EVOLVING BCI THERAPY, VVolume 2, P64, DOI 10.5772/intechopen.78695

Alimardani M, 2016, SCI REP-UK, V6, DOI 10.1038/srep33514

Angulo-Sherman IN, 2014, INT CONF ELECTR COMM, P14, DOI 10.1109/CONIELECOMP.2014.6808561

Belkacem AN, 2020, FRONT NEUROSCI-SWITZ, V14, DOI 10.3389/fnins.2020.00692

Benjamin EJ, 2018, CIRCULATION, V137, pE67, DOI 10.1161/CIR.0000000000000558

Bhattacharyya Saugat, 2019, IEEE Transactions on Robotics and Bionics, V1, P247, DOI 10.1109/TMRB.2019.2949854

Bishop C.M., 2006, PATTERN RECOGN

Blankertz B, 2008, IEEE SIGNAL PROC MAG, V25, P41, DOI 10.1109/MSP.2008.4408441

Bockbrader MA, 2018, PM&R, V10, pS233, DOI 10.1016/j.pmrj.2018.05.028

Chang CC, 2011, ACM T INTEL SYST TEC, V2, DOI 10.1145/1961189.1961199

Chaudhary U, 2015, ANN PHYS REHABIL MED, V58, P9, DOI 10.1016/j.rehab.2014.11.002

Choi I, 2016, IEEE SYS MAN CYBERN, P2324, DOI 10.1109/SMC.2016.7844585

Devore J.L., 2015, PROBABILITY STAT ENG, Vninth

Engel AK, 2010, CURR OPIN NEUROBIOL, V20, P156, DOI 10.1016/j.conb.2010.02.015

Faria-Fortini I, 2011, J HAND THER, V24, P257, DOI 10.1016/j.jht.2011.01.002

Foong R, 2020, IEEE T BIO-MED ENG, V67, P786, DOI 10.1109/TBME.2019.2921198

Frey J., 2014, P PHYCS INT C PHYS C

Ganzer PD, 2020, CELL, V181, P763, DOI 10.1016/j.cell.2020.03.054

Garry MI, 2004, J NEUROPHYSIOL, V91, P1570, DOI 10.1152/jn.00595.2003

Gomez-Pilar J, 2016, MED BIOL ENG COMPUT, V54, P1655, DOI 10.1007/s11517-016-1454-4

Sburlea AI, 2015, J NEURAL ENG, V12, DOI 10.1088/1741-2560/12/3/036007

Isakovic Milica, 2016, Eur J Transl Myol, V26, P6069

Jeannerod M, 1999, CURR OPIN NEUROBIOL, V9, P735, DOI 10.1016/S0959-4388(99)00038-0

Jeunet C, 2016, J NEURAL ENG, V13, DOI 10.1088/1741-2560/13/3/036024

Jirayucharoensak S, 2019, CLIN INTERV AGING, V14, P347, DOI 10.2147/CIA.S189047

Johansson RS, 2009, NAT REV NEUROSCI, V10, P345, DOI 10.1038/nrn2621

Kalcher J, 1996, MED BIOL ENG COMPUT, V34, P382, DOI 10.1007/BF02520010

Kim J, 2014, J PHYS THER SCI, V26, P215, DOI 10.1589/jpts.26.215

Kishore S, 2014, PRESENCE-TELEOP VIRT, V23, P242, DOI 10.1162/PRES_a_00192

Lazzaretti A.E., P 2019 9 INT IEEE EM, P526

Lee TS, 2013, PLOS ONE, V8, DOI 10.1371/journal.pone.0079419

Leeuwis N, 2020, IEEE SYS MAN CYBERN, P1518, DOI 10.1109/SMC42975.2020.9283259

Li AS, 2015, PLOS ONE, V10, DOI 10.1371/journal.pone.0116429

Li K., 2018, ELECTROTACTILE FEEDB

Li KR, 2019, IEEE T AUTOM SCI ENG, V16, P1556, DOI 10.1109/TASE.2018.2882465

Lotte F., 2016, BRAIN COMPUTER INTER

Ma XL, 2020, IEEE T NEUR SYS REH, V28, P297, DOI 10.1109/TNSRE.2019.2953121

McFarland DJ, 2003, BIOL PSYCHOL, V63, P237, DOI 10.1016/S0301-0511(03)00073-5

Miller KJ, 2010, P NATL ACAD SCI USA, V107, P4430, DOI 10.1073/pnas.0913697107

Moldoveanu A, 2019, IEEE ACCESS, V7, P8151, DOI 10.1109/ACCESS.2018.2886271

Muller-Gerking J, 1999, CLIN NEUROPHYSIOL, V110, P787, DOI 10.1016/S1388-2457(98)00038-8

Mulvey MR, 2012, NEUROMODULATION, V15, P42, DOI 10.1111/j.1525-1403.2011.00408.x

Nam CS, 2018, BRAIN-COMPUTER INTERFACES HANDBOOK: TECHNOLOGICAL AND THEORETICAL ADVANCES, P1

Neuper C, 1999, J CLIN NEUROPHYSIOL, V16, P373, DOI 10.1097/00004691-199907000-00010

Ono T, 2013, CLIN NEUROPHYSIOL, V124, P1779, DOI 10.1016/j.clinph.2013.03.006

Ono Y, 2018, NEUROPSYCHOLOGIA, V114, P134, DOI 10.1016/j.neuropsychologia.2018.04.016

Onose G, 2012, SPINAL CORD, V50, P599, DOI 10.1038/sc.2012.14

Pacheco K, 2017, P ANN INT IEEE EMBS, P1014, DOI 10.1109/EMBC.2017.8036998

PFURTSCHELLER G, 1989, ELECTROEN CLIN NEURO, V72, P250, DOI 10.1016/0013-4694(89)90250-2

Pfurtscheller G, 2001, P IEEE, V89, P1123, DOI 10.1109/5.939829

Pfurtscheller G, 1999, CLIN NEUROPHYSIOL, V110, P1842, DOI 10.1016/S1388-2457(99)00141-8

Pineda JA, 2003, IEEE T NEUR SYS REH, V11, P181, DOI 10.1109/TNSRE.2003.814445

Plow EB, 2010, CORTEX, V46, P310, DOI 10.1016/j.cortex.2009.02.024

Popovic DB, 2014, J ELECTROMYOGR KINES, V24, P795, DOI 10.1016/j.jelekin.2014.09.008

Ren SX, 2020, IEEE T NEUR SYS REH, V28, P1846, DOI 10.1109/TNSRE.2020.3001990

Reynolds C, 2015, CLIN NEUROPHYSIOL, V126, P1360, DOI 10.1016/j.clinph.2014.10.007

Romero-Laiseca MA, 2020, IEEE T NEUR SYS REH, V28, P988, DOI 10.1109/TNSRE.2020.2974056

Ron-Angevin R, 2009, NEUROSCI LETT, V449, P123, DOI 10.1016/j.neulet.2008.10.099

SANES JN, 1995, SCIENCE, V268, P1775, DOI 10.1126/science.7792606

Skola F, 2019, FRONT HUM NEUROSCI, V13, DOI 10.3389/fnhum.2019.00329

Tabernig CB, 2018, J REHABIL ASSIST TER, V5, DOI 10.1177/2055668318789280

Takeuchi N, 2012, NEURAL PLAST, V2012, DOI 10.1155/2012/823285

Tani M, 2018, NEUROSCI RES, V133, P7, DOI 10.1016/j.neures.2017.10.002

Tavakolan M, 2017, PLOS ONE, V12, DOI 10.1371/journal.pone.0174161

Tipper SP, 2010, Q J EXP PSYCHOL, V63, P2081, DOI 10.1080/17470211003624002

Trincado-Alonso F, 2018, J MED BIOL ENG, V38, P790, DOI 10.1007/s40846-017-0343-0

Vourvopoulos A, 2016, J NEUROENG REHABIL, V13, DOI 10.1186/s12984-016-0173-2

Wang ZP, 2019, J NEURAL ENG, V16, DOI 10.1088/1741-2552/ab377d

WHO, 2018, GLOB HLTH EST 2016 D

Wilson JA, 2012, J NEURAL ENG, V9, DOI 10.1088/1741-2560/9/4/045007

Wolpaw JR, 2002, CLIN NEUROPHYSIOL, V113, P767, DOI 10.1016/S1388-2457(02)00057-3

Yi W., P 2018 40 ANN INT C, P2527

NR 73

TC 1

Z9 1

U1 10

U2 19

PU HINDAWI LTD

PI LONDON

PA ADAM HOUSE, 3RD FLR, 1 FITZROY SQ, LONDON, W1T 5HF, ENGLAND

SN 1687-5265

EI 1687-5273

J9 COMPUT INTEL NEUROSC

JI Comput. Intell. Neurosci.

PD FEB 25

PY 2021

VL 2021

AR 8832686

DI 10.1155/2021/8832686

PG 13

WC Mathematical & Computational Biology; Neurosciences

WE Science Citation Index Expanded (SCI-EXPANDED)

SC Mathematical & Computational Biology; Neurosciences & Neurology

GA QU3FB

UT WOS:000627166900001

OA gold

DA 2022-06-21

ER

PT J

AU Adamovich, SV

August, K

Merians, A

Tunik, E

AF Adamovich, S. V.

August, K.

Merians, A.

Tunik, E.

TI A virtual reality-based system integrated with fmri to study neural

mechanisms of action observation-execution: A proof of concept study

SO RESTORATIVE NEUROLOGY AND NEUROSCIENCE

LA English

DT Article

DE Virtual environment; VR; motor control; imitation; hand

ID CORTICAL REORGANIZATION; FUNCTIONAL-ANATOMY; EXPOSURE THERAPY; MENTAL

PRACTICE; MOTOR IMAGERY; RECOVERY; REHABILITATION; ACTIVATION; MIRROR;

STROKE

AB Purpose: Emerging evidence shows that interactive virtual environments (VEs) may be a promising tool for studying sensorimotor processes and for rehabilitation. However, the potential of VEs to recruit action observation-execution neural networks is largely unknown. For the first time, a functional MRI-compatible virtual reality system (VR) has been developed to provide a window into studying brain-behavior interactions. This system is capable of measuring the complex span of hand-finger movements and simultaneously streaming this kinematic data to control the motion of representations of human hands in virtual reality.

Methods: In a blocked fMRI design, thirteen healthy subjects observed, with the intent to imitate (OTI), finger sequences performed by the virtual hand avatar seen in 1st person perspective and animated by pre-recorded kinematic data. Following this, subjects imitated the observed sequence while viewing the virtual hand avatar animated by their own movement in real-time. These blocks were interleaved with rest periods during which subjects viewed static virtual hand avatars and control trials in which the avatars were replaced with moving non-anthropomorphic objects.

Results: We show three main findings. First, both observation with intent to imitate and imitation with real-time virtual avatar feedback, were associated with activation in a distributed frontoparietal network typically recruited for observation and execution of real-world actions. Second, we noted a time-variant increase in activation in the left insular cortex for observation with intent to imitate actions performed by the virtual avatar. Third, imitation with virtual avatar feedback (relative to the control condition) was associated with a localized recruitment of the angular gyrus, precuneus, and extrastriate body area, regions which are (along with insular cortex) associated with the sense of agency.

Conclusions: Our data suggest that the virtual hand avatars may have served as disembodied training tools in the observation condition and as embodied "extensions" of the subject's own body (pseudo-tools) in the imitation. These data advance our understanding of the brain-behavior interactions when performing actions in VE and have implications in the development of observation-and imitation-based VR rehabilitation paradigms.

C1 [Adamovich, S. V.; Merians, A.; Tunik, E.] Univ Med & Dent New Jersey, Dept Rehabil & Movement Sci, Newark, NJ 07103 USA.

[Adamovich, S. V.; August, K.] New Jersey Inst Technol, Dept Biomed Engn, Newark, NJ 07102 USA.

[Tunik, E.] NYU, Dept Phys Therapy, New York, NY USA.

RP Tunik, E (通讯作者)，Univ Med & Dent New Jersey, Dept Rehabil & Movement Sci, 65 Bergen St,Room 714, Newark, NJ 07103 USA.

EM tunikeu@umdnj.edu

RI Tunik, Eugene/O-8327-2015

OI Tunik, Eugene/0000-0002-7497-1251

FU New York University; Steinhardt School of Culture, Education, and Human

Development; University of Medicine and Dentistry of New Jersey;

Department of Rehabilitation and Movement Science; NIH [HD 42161];

National Institute on Disability and Rehabilitation Research RERC

[H133E050011]; EUNICE KENNEDY SHRIVER NATIONAL INSTITUTE OF CHILD HEALTH

& HUMAN DEVELOPMENT [R01HD058301] Funding Source: NIH RePORTER; EUNICE

KENNEDY SHRIVER NATIONAL INSTITUTE OF CHILD HEALTH &HUMAN DEVELOPMENT

[R03HD042161] Funding Source: NIH RePORTER

FX Special thanks to On-Yee Lo, Jeffrey Lewis, and Qinyin Qiu for expert

technical assistance. This work was supported by institutional funds

provided by New York University, Steinhardt School of Culture,

Education, and Human Development ( ET), University of Medicine and

Dentistry of New Jersey, Department of Rehabilitation and Movement

Science ( ET), and by the NIH grant HD 42161 ( SA) and National

Institute on Disability and Rehabilitation Research RERC Grant #

H133E050011 ( SA).

CR *5 DIM TECHN, 5DT DAT GLOV 16 MRI

ADAMOVICH S, IEEE T NEUR IN PRESS

Adamovich S, 2008, IEEE ENG MED BIO, P3475, DOI 10.1109/IEMBS.2008.4649954

Adamovich SV, 2005, PRESENCE-TELEOP VIRT, V14, P161, DOI 10.1162/1054746053966996

Altschuter EL, 2005, PERCEPTION, V34, P1153, DOI 10.1068/p3409bn

BLATT GJ, 1990, J COMP NEUROL, V299, P421, DOI 10.1002/cne.902990404

BLUM B, 1985, BEHAV BRAIN RES, V18, P167, DOI 10.1016/0166-4328(85)90072-5

Buccino G, 2001, EUR J NEUROSCI, V13, P400, DOI 10.1046/j.1460-9568.2001.01385.x

Buccino G, 2006, COGN BEHAV NEUROL, V19, P55, DOI 10.1097/00146965-200603000-00007

Butler AJ, 2006, ARCH PHYS MED REHAB, V87, pS2, DOI 10.1016/j.apmr.2006.08.326

Cavanna AE, 2006, BRAIN, V129, P564, DOI 10.1093/brain/awl004

Celnik P, 2006, NEUROIMAGE, V29, P677, DOI 10.1016/j.neuroimage.2005.07.039

Celnik P, 2008, STROKE, V39, P1814, DOI 10.1161/STROKEAHA.107.508184

Corradi-Dell'Acqua C, 2008, NEUROIMAGE, V40, P1902, DOI 10.1016/j.neuroimage.2007.12.062

David N, 2007, NEUROIMAGE, V36, P1004, DOI 10.1016/j.neuroimage.2007.03.030

Decety J, 1997, BRAIN, V120, P1763, DOI 10.1093/brain/120.10.1763

Diedrichsen J, 2005, J NEUROSCI, V25, P9919, DOI 10.1523/JNEUROSCI.1874-05.2005

Downing PE, 2007, J NEUROSCI, V27, P226, DOI 10.1523/JNEUROSCI.3619-06.2007

Downing PE, 2001, SCIENCE, V293, P2470, DOI 10.1126/science.1063414

Ehrsson HH, 2001, J NEUROPHYSIOL, V85, P2613, DOI 10.1152/jn.2001.85.6.2613

Farrer C, 2003, NEUROIMAGE, V18, P324, DOI 10.1016/S1053-8119(02)00041-1

Farrer C, 2002, NEUROIMAGE, V15, P596, DOI 10.1006/nimg.2001.1009

FARRER C, 2007, CEREB CORTEX

Gaggioli A, 2006, NEUROREHAB NEURAL RE, V20, P503, DOI 10.1177/1545968306290224

Gallese V, 2004, TRENDS COGN SCI, V8, P396, DOI 10.1016/j.tics.2004.07.002

Grafton ST, 1996, CEREB CORTEX, V6, P226, DOI 10.1093/cercor/6.2.226

Gregoriou GG, 2006, J COMP NEUROL, V496, P422, DOI 10.1002/cne.20933

Grezes J, 2001, NEUROIMAGE, V13, P775, DOI 10.1006/nimg.2000.0740

Hanakawa T, 2003, J NEUROPHYSIOL, V89, P989, DOI 10.1152/jn.00132.2002

Holden MK, 2007, IEEE T NEUR SYS REH, V15, P36, DOI 10.1109/TNSRE.2007.891388

Holden MK, 2005, CYBERPSYCHOL BEHAV, V8, P187, DOI 10.1089/cpb.2005.8.187

Kilner JM, 2004, NEUROREPORT, V15, P637, DOI 10.1097/00001756-200403220-00013

MACKAY WA, 1992, J NEUROPHYSIOL, V67, P1335, DOI 10.1152/jn.1992.67.5.1335

McCloy R, 2001, BRIT MED J, V323, P912, DOI 10.1136/bmj.323.7318.912

Merians AS, 2006, NEUROREHAB NEURAL RE, V20, P252, DOI 10.1177/1545968306286914

MERIANS AS, VIRTUAL REA IN PRESS

MIRELMAN A, 2008, STROKE 1106

OLDFIELD RC, 1971, NEUROPSYCHOLOGIA, V9, P97, DOI 10.1016/0028-3932(71)90067-4

Peelen MV, 2007, NAT REV NEUROSCI, V8, P636, DOI 10.1038/nrn2195

Peelen MV, 2005, HUM BRAIN MAPP, V25, P402, DOI 10.1002/hbm.20116

Pellijeff A, 2006, NEUROPSYCHOLOGIA, V44, P2685, DOI 10.1016/j.neuropsychologia.2006.01.009

Perani D, 2001, NEUROIMAGE, V14, P749, DOI 10.1006/nimg.2001.0872

Pomeroy VM, 2005, NEUROREHAB NEURAL RE, V19, P4, DOI 10.1177/1545968304274351

Powers MB, 2008, J ANXIETY DISORD, V22, P561, DOI 10.1016/j.janxdis.2007.04.006

Riva G, 2006, BEHAV RES METHODS, V38, P237, DOI 10.3758/BF03192775

Rizzo AA, 2008, STUD HEALTH TECHNOL, V132, P420

Rizzolatti G, 2001, NEURON, V31, P889, DOI 10.1016/S0896-6273(01)00423-8

Rozzi S, 2006, CEREB CORTEX, V16, P1389, DOI 10.1093/cercor/bhj076

Rushworth MFS, 2006, CEREB CORTEX, V16, P1418, DOI 10.1093/cercor/bhj079

Spiridon M, 2006, HUM BRAIN MAPP, V27, P77, DOI 10.1002/hbm.20169

Suchan B, 2008, BEHAV BRAIN RES, V188, P78, DOI 10.1016/j.bbr.2007.10.021

Tai YF, 2004, CURR BIOL, V14, P117, DOI 10.1016/j.cub.2004.01.005

Tunik E, 2007, J NEUROPHYSIOL, V97, P2107, DOI 10.1152/jn.00405.2006

Urgesi C, 2004, CURR BIOL, V14, P2130, DOI 10.1016/j.cub.2004.11.031

Vaillancourt DE, 2007, NEUROIMAGE, V36, P793, DOI 10.1016/j.neuroimage.2007.03.002

Vingerhoets G, 2002, NEUROIMAGE, V17, P1623, DOI 10.1006/nimg.2002.1290

Yokochi H, 2003, SOMATOSENS MOT RES, V20, P115, DOI 10.1080/0899022031000105145

You SH, 2005, DEV MED CHILD NEUROL, V47, P628, DOI 10.1017/S0012162205001234

You SH, 2005, STROKE, V36, P1166, DOI 10.1161/01.STR.0000162715.43417.91

VIRTUAL REALITY PERI

NR 60

TC 51

Z9 52

U1 0

U2 32

PU IOS PRESS

PI AMSTERDAM

PA NIEUWE HEMWEG 6B, 1013 BG AMSTERDAM, NETHERLANDS

SN 0922-6028

EI 1878-3627

J9 RESTOR NEUROL NEUROS

JI Restor. Neurol. Neurosci.

PY 2009

VL 27

IS 3

BP 209

EP 223

DI 10.3233/RNN-2009-0471

PG 15

WC Neurosciences

WE Science Citation Index Expanded (SCI-EXPANDED)

SC Neurosciences & Neurology

GA 467SU

UT WOS:000267763500006

PM 19531876

OA Green Accepted

DA 2022-06-21

ER

PT J

AU Adamovich, SV

Fluet, GG

Merians, AS

Mathai, A

Qiu, QY

AF Adamovich, Sergei V.

Fluet, Gerard G.

Merians, Alma S.

Mathai, Abraham

Qiu, Qinyin

TI Incorporating Haptic Effects Into Three-Dimensional Virtual Environments

to Train the Hemiparetic Upper Extremity

SO IEEE TRANSACTIONS ON NEURAL SYSTEMS AND REHABILITATION ENGINEERING

LA English

DT Article

DE Cerebrovascular accident; neuroplasticity; robotics; upper extremity;

virtual reality

ID CHRONIC STROKE; PARKINSONS-DISEASE; ROBOTIC ASSISTANCE; MOVEMENT

PATTERNS; SUBACUTE STROKE; MOTOR RECOVERY; REHABILITATION;

NEUROREHABILITATION; POSTSTROKE; THERAPY

AB Current neuroscience has identified several constructs to increase the effectiveness of upper extremity rehabilitation. One is the use of progressive, skill acquisition-oriented training. Another approach emphasizes the use of bilateral activities. Building on these principles, this paper describes the design and feasibility testing of a robotic/virtual environment system designed to train the arm of persons who have had strokes. The system provides a variety of assistance modes, scalable workspaces and hand-robot interfaces allowing persons with strokes to train multiple joints in three dimensions. The simulations utilize assistance algorithms that adjust task difficulty both online and offline in relation to subject performance. Several distinctive haptic effects have been incorporated into the simulations. An adaptive master-slave relationship between the unimpaired and impaired arm encourages active movement of the subject's hemiparetic arm during a bimanual task. Adaptive anti-gravity support and damping stabilize the arm during virtual reaching and placement tasks. An adaptive virtual spring provides assistance to complete the movement if the subject is unable to complete the task in time. Finally, haptically rendered virtual objects help to shape the movement trajectory during a virtual placement task. A proof of concept study demonstrated this system to be safe, feasible and worthy of further study.

C1 [Adamovich, Sergei V.; Mathai, Abraham; Qiu, Qinyin] New Jersey Inst Technol, Newark, NJ 07102 USA.

[Fluet, Gerard G.; Merians, Alma S.] Univ Med & Dent New Jersey, Newark, NJ 07107 USA.

RP Adamovich, SV (通讯作者)，New Jersey Inst Technol, Newark, NJ 07102 USA.

EM sergei.adamovich@njit.edu; fluetge@umdnj.edu; merians@umdnj.edu;

am65@njit.edu; qq4@njit.edu

RI LUO, JINGJING/AAZ-6697-2021

OI LUO, JINGJING/0000-0002-3222-1276

FU National Institutes of Health [HD 58301, 42161]; National Institute on

Disability and Rehabilitation Research RERC [H133E050011]; EUNICE

KENNEDY SHRIVER NATIONAL INSTITUTE OF CHILD HEALTH & HUMAN DEVELOPMENT

[R01HD058301] Funding Source: NIH RePORTER; EUNICE KENNEDY SHRIVER

NATIONAL INSTITUTE OF CHILD HEALTH &HUMAN DEVELOPMENT [R03HD042161]

Funding Source: NIH RePORTER

FX This work was supported in part by the National Institutes of Health

under Grant HD 58301 & 42161 and in part by the National Institute on

Disability and Rehabilitation Research RERC under Grant H133E050011.

CR ADAMOVICH S, 2008, P 28 EMBC ANN INT C, P3475

Adamovich SV, 2005, PRESENCE-TELEOP VIRT, V14, P161, DOI 10.1162/1054746053966996

Adamovich SV, 2001, NEUROSCIENCE, V104, P1027, DOI 10.1016/S0306-4522(01)00099-9

Amirabdollahian F, 2002, 2002 IEEE INTERNATIONAL CONFERENCE ON ROBOTICS AND AUTOMATION, VOLS I-IV, PROCEEDINGS, P3380, DOI 10.1109/ROBOT.2002.1014233

BOHANNON RW, 1987, PHYS THER, V67, P206, DOI 10.1093/ptj/67.2.206

Cauraugh JH, 2005, PROG NEUROBIOL, V75, P309, DOI 10.1016/j.pneurobio.2005.04.001

DUNCAN PW, 1992, STROKE, V23, P1084, DOI 10.1161/01.STR.23.8.1084

Ellis MD, 2008, NEUROREHAB NEURAL RE, V22, P321, DOI 10.1177/1545968307313509

Fasoli SE, 2003, ARCH PHYS MED REHAB, V84, P477, DOI 10.1053/apmr.2003.50110

GOWLAND C, 1992, PHYS THER, V72, P624, DOI 10.1093/ptj/72.9.624

Harwin W, 2001, ASSIST TECHN RES SER, V10, P36

Hogan N, 2006, J REHABIL RES DEV, V43, P605, DOI 10.1682/JRRD.2005.06.0103

Holden MK, 2005, CYBERPSYCHOL BEHAV, V8, P187, DOI 10.1089/cpb.2005.8.187

Hornby TG, 2008, STROKE, V39, P1786, DOI 10.1161/STROKEAHA.107.504779

Houtsma JA, 2006, P I MECH ENG H, V220, P715, DOI 10.1243/09544119H06104

Krebs Hermano I, 2004, J Neuroeng Rehabil, V1, P5, DOI 10.1186/1743-0003-1-5

Krebs HI, 2008, NEUROREHABILITATION, V23, P81

Krebs HI, 2007, IEEE T NEUR SYS REH, V15, P327, DOI 10.1109/TNSRE.2007.903899

Krebs HI, 2003, AUTON ROBOT, V15, P7, DOI 10.1023/A:1024494031121

Lum PS, 2006, J REHABIL RES DEV, V43, P631, DOI 10.1682/JRRD.2005.02.0044

MAJASK M, 1996, TOP STROKE REHABIL, V3, P27

Merians AS, 2006, NEUROREHAB NEURAL RE, V20, P252, DOI 10.1177/1545968306286914

Merians AS, 2002, PHYS THER, V82, P898, DOI 10.1093/ptj/82.9.898

Nef T, 2007, MED BIOL ENG COMPUT, V45, P887, DOI 10.1007/s11517-007-0226-6

Patterson Scott D., 2004, Current Proteomics, V1, P3, DOI 10.2174/1570164043488306

Patton JL, 2006, EXP BRAIN RES, V168, P368, DOI 10.1007/s00221-005-0097-8

Patton JL, 2004, IEEE T BIO-MED ENG, V51, P636, DOI 10.1109/TBME.2003.821035

Plautz EJ, 2000, NEUROBIOL LEARN MEM, V74, P27, DOI 10.1006/nlme.1999.3934

Reinkensmeyer DJ, 2004, ANNU REV BIOMED ENG, V6, P497, DOI 10.1146/annurev.bioeng.6.040803.140223

Rohrer B, 2002, J NEUROSCI, V22, P8297

Rosati G, 2007, IEEE T NEUR SYS REH, V15, P560, DOI 10.1109/TNSRE.2007.908560

SANCHEZ RJ, 2005, P 2005 IEEE 9 INT C

Stewart KC, 2006, J NEUROL SCI, V244, P89, DOI 10.1016/j.jns.2006.01.005

Stinear JW, 2004, J CLIN NEUROPHYSIOL, V21, P124, DOI 10.1097/00004691-200403000-00008

Sugar TG, 2007, IEEE T NEUR SYS REH, V15, P336, DOI 10.1109/TNSRE.2007.903903

Todorov E, 2004, NAT NEUROSCI, V7, P907, DOI 10.1038/nn1309

Tresilian JR, 1997, BRAIN, V120, P2093, DOI 10.1093/brain/120.11.2093

Van der Linde R.Q., 2002, P EUR MADR SPAIN 10, P1

Waller SM, 2004, ARCH PHYS MED REHAB, V85, P1076, DOI 10.1016/j.apmr.2003.10.020

Whitall J, 2000, STROKE, V31, P2390, DOI 10.1161/01.STR.31.10.2390

Wisneski KJ, 2007, J NEUROENG REHABIL, V4, DOI 10.1186/1743-0003-4-7

Wolbrecht ET, 2008, IEEE T NEUR SYS REH, V16, P286, DOI 10.1109/TNSRE.2008.918389

Wolf SL, 2005, NEUROREHAB NEURAL RE, V19, P194, DOI 10.1177/1545968305276663

NR 43

TC 41

Z9 43

U1 1

U2 16

PU IEEE-INST ELECTRICAL ELECTRONICS ENGINEERS INC

PI PISCATAWAY

PA 445 HOES LANE, PISCATAWAY, NJ 08855-4141 USA

SN 1534-4320

EI 1558-0210

J9 IEEE T NEUR SYS REH

JI IEEE Trans. Neural Syst. Rehabil. Eng.

PD OCT

PY 2009

VL 17

IS 5

BP 512

EP 520

DI 10.1109/TNSRE.2009.2028830

PG 9

WC Engineering, Biomedical; Rehabilitation

WE Science Citation Index Expanded (SCI-EXPANDED)

SC Engineering; Rehabilitation

GA 515OV

UT WOS:000271482600013

PM 19666345

OA Green Accepted

DA 2022-06-21

ER

PT J

AU Adamovich, SV

Fluet, GG

Mathai, A

Qiu, QY

Lewis, J

Merians, AS

AF Adamovich, Sergei V.

Fluet, Gerard G.

Mathai, Abraham

Qiu, Qinyin

Lewis, Jeffrey

Merians, Alma S.

TI Design of a complex virtual reality simulation to train finger motion

for persons with hemiparesis: a proof of concept study

SO JOURNAL OF NEUROENGINEERING AND REHABILITATION

LA English

DT Article

ID STROKE; REHABILITATION; ENVIRONMENT; PLASTICITY; POSTSTROKE; MOVEMENT;

SYSTEM

AB Background: Current neuroscience has identified rehabilitation approaches with the potential to stimulate adaptive changes in the brains of persons with hemiparesis. These approaches include, intensive task-oriented training, bimanual activities and balancing proximal and distal upper extremity interventions to reduce competition between these segments for neural territory.

Methods: This paper describes the design and feasibility testing of a robotic/virtual environment system designed to train the hand and arm of persons with hemiparesis. The system employs a simulated piano that presents visual, auditory and tactile feedback comparable to an actual piano. Arm tracking allows patients to train both the arm and hand as a coordinated unit, emphasizing the integration of both transport and manipulation phases. The piano trainer includes songs and scales that can be performed with one or both hands. Adaptable haptic assistance is available for more involved subjects. An algorithm adjusts task difficulty in proportion to subject performance. A proof of concept study was performed on four subjects with upper extremity hemiparesis secondary to chronic stroke to establish: a) the safety and feasibility of this system and b) the concurrent validity of robotically measured kinematic and performance measures to behavioral measures of upper extremity function.

Results: None of the subjects experienced adverse events or responses during or after training. As a group, the subjects improved in both performance time and key press accuracy. Three of the four subjects demonstrated improvements in fractionation, the ability to move each finger individually. Two subjects improved their aggregate time on the Jebsen Test of Hand Function and three of the four subjects improved in Wolf Motor Function Test aggregate time.

Conclusion: The system designed in this paper has proven to be safe and feasible for the training of hand function for persons with hemiparesis. It features a flexible design that allows for the use and further study of adjustments in point of view, bilateral and unimanual treatment modes, adaptive training algorithms and haptically rendered collisions in the context of rehabilitation of the hemiparetic hand.

C1 [Adamovich, Sergei V.; Fluet, Gerard G.; Lewis, Jeffrey; Merians, Alma S.] Univ Med & Dent New Jersey, Dept Rehabil & Movement Sci, Newark, NJ 07103 USA.

[Adamovich, Sergei V.; Mathai, Abraham; Qiu, Qinyin; Lewis, Jeffrey] New Jersey Inst Technol, Dept Biomed Engn, Newark, NJ 07102 USA.

RP Fluet, GG (通讯作者)，Univ Med & Dent New Jersey, Dept Rehabil & Movement Sci, Newark, NJ 07103 USA.

EM sergei.adamovich@njit.edu; fluet179@comcast.net; ams6@njit.edu;

qq4@njit.edu; jelewis09@gsb.columbia.edu; merians@umdnj.edu

FU NIH [HD 42161]; National Institute on Disability and Rehabilitation

Research RERC [H133E050011]; EUNICE KENNEDY SHRIVER NATIONAL INSTITUTE

OF CHILD HEALTH & HUMAN DEVELOPMENT [R01HD058301] Funding Source: NIH

RePORTER; EUNICE KENNEDY SHRIVER NATIONAL INSTITUTE OF CHILD HEALTH

&HUMAN DEVELOPMENT [R03HD042161] Funding Source: NIH RePORTER

FX This work was supported in part by NIH grant HD 42161 and by the

National Institute on Disability and Rehabilitation Research RERC (Grant

# H133E050011).

CR Adamovich SV, 2004, P ANN INT IEEE EMBS, V26, P4936

Adamovich SV, 2005, PRESENCE-TELEOP VIRT, V14, P161, DOI 10.1162/1054746053966996

BOHANNON RW, 1987, PHYS THER, V67, P206, DOI 10.1093/ptj/67.2.206

Bouzit M, 2002, IEEE-ASME T MECH, V7, P256, DOI 10.1109/TMECH.2002.1011262

DOVAT L, 2008, INT CONV REH ENG ASS

Fischer HC, 2007, TOP STROKE REHABIL, V14, P1, DOI 10.1310/tsr1401-1

GOWLAND C, 1992, PHYS THER, V72, P624, DOI 10.1093/ptj/72.9.624

GOWLAND C, 1993, STROKE, V24, P58, DOI 10.1161/01.STR.24.1.58

Hlustik P, 2004, J CLIN NEUROPHYSIOL, V21, P180

HUANG H, 2006, C P IEEE ENG MED BIO, V1, P4925

JEBSEN R H, 1969, Archives of Physical Medicine and Rehabilitation, V50, P311

KAWASAKI H, 2007, IEEE INT C ROB REH I

Kleim JA, 1998, J NEUROPHYSIOL, V80, P3321, DOI 10.1152/jn.1998.80.6.3321

Mahncke HW, 2006, PROG BRAIN RES, V157, P81, DOI 10.1016/S0079-6123(06)57006-2

Merians AS, 2006, NEUROREHAB NEURAL RE, V20, P252, DOI 10.1177/1545968306286914

Merians AS, 2002, PHYS THER, V82, P898, DOI 10.1093/ptj/82.9.898

Nudo RJ, 2003, J REHABIL MED, V35, P7, DOI 10.1080/16501960310010070

Plautz EJ, 2000, NEUROBIOL LEARN MEM, V74, P27, DOI 10.1006/nlme.1999.3934

Schneider S, 2007, J NEUROL, V254, P1339, DOI 10.1007/s00415-006-0523-2

Shing CY, 2003, ROBOTICA, V21, P211, DOI 10.1017/S0263574702004708

Wolf SL, 2005, NEUROREHAB NEURAL RE, V19, P194, DOI 10.1177/1545968305276663

ZHOU Z, 2005, 9 INT C COMP AID DES, P16

NR 22

TC 82

Z9 89

U1 4

U2 35

PU BMC

PI LONDON

PA CAMPUS, 4 CRINAN ST, LONDON N1 9XW, ENGLAND

SN 1743-0003

J9 J NEUROENG REHABIL

JI J. NeuroEng. Rehabil.

PD JUL 17

PY 2009

VL 6

AR 28

DI 10.1186/1743-0003-6-28

PG 10

WC Engineering, Biomedical; Neurosciences; Rehabilitation

WE Science Citation Index Expanded (SCI-EXPANDED)

SC Engineering; Neurosciences & Neurology; Rehabilitation

GA 490ZJ

UT WOS:000269546300001

PM 19615045

OA Green Published, gold

DA 2022-06-21

ER

PT J

AU Adamovich, SV

Merians, AS

Boian, R

Lewis, JA

Tremaine, M

Burdea, GS

Recce, M

Poizner, H

AF Adamovich, SV

Merians, AS

Boian, R

Lewis, JA

Tremaine, M

Burdea, GS

Recce, M

Poizner, H

TI A virtual reality-based exercise system for hand rehabilitation

post-stroke

SO PRESENCE-TELEOPERATORS AND VIRTUAL ENVIRONMENTS

LA English

DT Article

ID STROKE REHABILITATION

AB This paper presents preliminary results from a virtual reality (VR)-based system for hand rehabilitation that uses a CyberGlove and a Rutgers Master II-ND haptic glove. This computerized system trains finger range of motion, finger flexion speed, independence of finger motion, and finger strength using specific VR simulation exercises. A remote Web-based monitoring station was developed to allow telerehabilitation interventions. The remote therapist observes simplified versions of the patient exercises that are updated in real time. Patient data is stored transparently in an Oracle database, which is also Web accessible through a portal GUI. Thus the remote therapist or attending physician can graph exercise outcomes and thus evaluate patient outcomes at a distance. Data from the VR simulations is complemented by clinical measurements of hand function and strength. Eight chronic post-stroke subjects participated in a pilot study of the above system. In keeping with variability in both their lesion size and site and in their initial upper extremity function, each subject showed improvement on a unique combination of movement parameters in VR training. Importantly, these improvements transferred to gains on clinical tests, as well as to significant reductions in task-completion times for the prehension of real objects. These results are indicative of the potential feasibility of this exercise system for rehabilitation in patients with hand dysfunction resulting from neurological impairment.

C1 Univ Med & Dent New Jersey, Dept Dev & Rehabil Sci, Newark, NJ 07103 USA.

New Jersey Inst Technol, Dept Biomed Engn, Newark, NJ 07102 USA.

Rutgers State Univ, Sch Engn, Ctr Adv Informat Proc, Piscataway, NJ USA.

New Jersey Inst Technol, Coll Comp Sci, Newark, NJ 07102 USA.

Rutgers State Univ, Ctr Mol & Behav Neurosci, Newark, NJ 07102 USA.

RP Adamovich, SV (通讯作者)，Univ Med & Dent New Jersey, Dept Dev & Rehabil Sci, Newark, NJ 07103 USA.

EM adamovic@njit.edu

CR Boian R, 2002, STUD HEALTH TECHNOL, V85, P64

Bouzit M, 2002, IEEE-ASME T MECH, V7, P256, DOI 10.1109/TMECH.2002.1011262

Buckley K. M., 2001, P STAT SCI C TEL APP, P35

Burdea G, 2000, IEEE T REHABIL ENG, V8, P430, DOI 10.1109/86.867886

Burdea G., 2002, 1 INT WORKSH VIRT RE, P1

Burdea G.C., 2003, VIRTUAL REALITY TECH, V2nd

Burgar CG, 2000, J REHABIL RES DEV, V37, P663

DHURJATY S, 2001, P STAT SCI C TEL APP, P89

Goldstein Larry B, 2003, Adv Neurol, V92, P447

Holden M., 1999, NEUROLOGY REPORT, V23, P57

Holden M. K., 2001, USABILITY EVALUATION, P624

HOLDEN MK, 2002, NEUROLOGY REPORT, V26, P62

HOLDEN MK, 2003, P 2 INT WORKSH VIRT, P4

Jack D, 2001, IEEE T NEUR SYS REH, V9, P308, DOI 10.1109/7333.948460

JEBSEN R H, 1969, Archives of Physical Medicine and Rehabilitation, V50, P311

Krebs H I, 1998, IEEE Trans Rehabil Eng, V6, P75, DOI 10.1109/86.662623

Krebs HI, 2003, ROBOTICA, V21, P3, DOI 10.1017/S0263574702004587

Lewis J, 2003, STUD HEALTH TECHNOL, V94, P190

Merians AS, 2002, PHYS THER, V82, P898, DOI 10.1093/ptj/82.9.898

Muellbacher W, 2002, ARCH NEUROL-CHICAGO, V59, P1278, DOI 10.1001/archneur.59.8.1278

Nudo RJ, 2001, MUSCLE NERVE, V24, P1000, DOI 10.1002/mus.1104

Plautz EJ, 2000, NEUROBIOL LEARN MEM, V74, P27, DOI 10.1006/nlme.1999.3934

Popescu GV, 2002, STUD HEALTH TECHNOL, V85, P362

Reinkensmeyer DJ, 2000, J REHABIL RES DEV, V37, P653

Reinkensmeyer DJ, 2002, IEEE T NEUR SYS REH, V10, P102, DOI 10.1109/TNSRE.2002.1031978

Rosen MJ, 1999, NEUROREHABILITATION, V12, P11

Taub E, 1997, Top Stroke Rehabil, V3, P38, DOI 10.1080/10749357.1997.11754128

Volpe BT, 2000, NEUROLOGY, V54, P1938, DOI 10.1212/WNL.54.10.1938

Volpe BT, 2001, CURR OPIN NEUROL, V14, P745, DOI 10.1097/00019052-200112000-00011

NR 29

TC 66

Z9 68

U1 0

U2 29

PU MIT PRESS

PI CAMBRIDGE

PA ONE ROGERS ST, CAMBRIDGE, MA 02142-1209 USA

EI 1531-3263

J9 PRESENCE-TELEOP VIRT

JI Presence-Teleoper. Virtual Env.

PD APR

PY 2005

VL 14

IS 2

BP 161

EP 174

DI 10.1162/1054746053966996

PG 14

WC Computer Science, Cybernetics; Computer Science, Software Engineering

WE Science Citation Index Expanded (SCI-EXPANDED); Social Science Citation Index (SSCI)

SC Computer Science

GA 932FF

UT WOS:000229538900004

DA 2022-06-21

ER

PT J

AU Adams, RJ

Lichter, MD

Ellington, A

White, M

Armstead, K

Patrie, JT

Diamond, PT

AF Adams, Richard J.

Lichter, Matthew D.

Ellington, Allison

White, Marga

Armstead, Kate

Patrie, James T.

Diamond, Paul T.

TI Virtual Activities of Daily Living for Recovery of Upper Extremity Motor

Function

SO IEEE TRANSACTIONS ON NEURAL SYSTEMS AND REHABILITATION ENGINEERING

LA English

DT Article

DE Upper extremity rehabilitation; stroke therapy; virtual reality; human

computer interaction; human motion tracking; human motor performance

ID OCCUPATIONAL-THERAPY; HEART-DISEASE; UPPER-LIMB; STROKE; REALITY;

REHABILITATION; PEOPLE; UPDATE

AB A study was conducted to investigate the effectiveness of virtual activities of daily living (ADL) practice using the SaeboVR software system for the recovery of upper extremity (UE) motor function following stroke. The system employs Kinect sensor-based tracking to translate human UE motion into the anatomical pose of the arm of the patient's avatar within a virtual environment, creating a virtual presence within a simulated task space. Patients gain mastery of 12 different integrated activities while traversing a metaphorical "road to recovery" that includes thematically linked levels and therapist-selected difficulty settings. Clinical trials were conducted under the study named Virtual Occupational Therapy Application. A total of 15 chronic phase stroke survivors completed a protocol involving three sessions per week over eight weeks, during which they engaged in repetitive task practice through performance of the virtual ADLs. Results show a clinically important improvement and statistically significant difference in Fugl-Meyer UE assessment scores in the study population of chronic stroke survivors over the eight-week interventional period compared with a non-interventional control period of equivalent duration. Statistically significant and clinically important improvements are also found in the wolf motor function test scores. These results provide new evidence for the use of virtual ADL practice as a tool for UE therapy for stroke patients. Limitations of the study include non-blinded assessments and the possibility of selection and/or attrition bias.

C1 [Adams, Richard J.; Lichter, Matthew D.] Barron Associates Inc, Charlottesville, VA 22901 USA.

[Ellington, Allison] Mary Baldwin Univ, Murphy Deming Coll Hlth Sci, Staunton, VA 24401 USA.

[White, Marga; Armstead, Kate] UVA HealthSouth Rehabil Hosp, Charlottesville, VA 22908 USA.

[Patrie, James T.] Univ Virginia, Dept Publ Hlth Sci, Charlottesville, VA 22908 USA.

[Diamond, Paul T.] Univ Virginia, Dept Phys Med & Rehabil, Charlottesville, VA 22908 USA.

RP Adams, RJ (通讯作者)，Barron Associates Inc, Charlottesville, VA 22901 USA.

EM adams@barron-associates.com; lichter@barron-associates.com;

aellington@marybaldwin.edu; marga.white@healthsouth.com;

kate.armstead@healthsouth.com; jp4h@virginia.edu; ptd2m@virginia.edu

FU HealthSouth Corporation; Designing Digitally Inc.; EUNICE KENNEDY

SHRIVER NATIONAL INSTITUTE OF CHILD HEALTH & HUMAN DEVELOPMENT

[R44HD071745] Funding Source: NIH RePORTER

FX The authors would like to recognize the HealthSouth Corporation and

Designing Digitally Inc. for their support of the VOTA/SaeboVR project.

We would also like to thank Paula Adams, OTR/L and Eileen Krepkovich

(Barron Associates, Inc.) for their contributions to the program.

CR Adams RJ, 2015, IEEE T NEUR SYS REH, V23, P287, DOI 10.1109/TNSRE.2014.2360149

Allain P, 2014, J INT NEUROPSYCH SOC, V20, P468, DOI 10.1017/S1355617714000344

[Anonymous], 2017, Circulation, V136, pe196, DOI 10.1161/CIR.0000000000000530

[Anonymous], 2014, OCCUPATIONAL THERAPY, VThird

Clark Gloria Frolek, 2008, Am J Occup Ther, V62, P684

ClinicalTrials. gov, 2016, VIRTUAL OCCUPATIONAL

Dohle C, 2009, NEUROREHAB NEURAL RE, V23, P209, DOI 10.1177/1545968308324786

Ellington A, 2015, AM J OCCUP THER, V69, DOI 10.5014/ajot.2015.014373

Fugl-Meyer A. R., SCANDINAVIAN J REHAB, V7, P13

Hallett M, 2001, BRAIN RES REV, V36, P169, DOI 10.1016/S0165-0173(01)00092-3

Jung SH, 2005, ARCH PHYS MED REHAB, V86, P2218, DOI 10.1016/j.apmr.2005.04.015

Katz N, 2005, DISABIL REHABIL, V27, P1235, DOI 10.1080/09638280500076079

Kim JM, 2015, FRONT PLANT SCI, V6, DOI [10.3389/fpls.2015.00114, 10.3389/fpsyg.2015.00248]

Kiper P, 2014, BIOMED RES INT, V2014, DOI 10.1155/2014/752128

Kristensen HK, 2011, SCAND J OCCUP THER, V18, P11, DOI 10.3109/11038120903563785

Kwakkel G, 2003, STROKE, V34, P2181, DOI 10.1161/01.STR.0000087172.16305.CD

Kwon JS, 2012, NEUROREHABILITATION, V31, P379, DOI 10.3233/NRE-2012-00807

Lam YS, 2006, NEUROREHABILITATION, V21, P245

Lang CE, 2009, ARCH PHYS MED REHAB, V90, P1692, DOI 10.1016/j.apmr.2009.04.005

Laver KE, 2011, COCHRANE DB SYST REV, DOI [10.1002/14651858.CD008349.pub2, 10.1002/14651858.CD008349.pub4, 10.1002/14651858.CD008349.pub3]

LAWTON MP, 1969, GERONTOLOGIST, V9, P179, DOI 10.1093/geront/9.3_Part_1.179

Legg L, 2007, BMJ-BRIT MED J, V335, P922, DOI 10.1136/bmj.39343.466863.55

Lin KC, 2009, NEUROREHAB NEURAL RE, V23, P429, DOI 10.1177/1545968308331144

LOWENTHAL MF, 1964, LIVES DISTRESS

Lum PS, 2009, TOP STROKE REHABIL, V16, P237, DOI 10.1310/tsr1604-237

Mozaffarian D, 2016, CIRCULATION, V133, pE38, DOI 10.1161/CIR.0000000000000350

Nudo RJ, 2003, J REHABIL MED, V35, P7, DOI 10.1080/16501960310010070

Page SJ, 2012, PHYS THER, V92, P791, DOI 10.2522/ptj.20110009

Page SJ, 2009, NEUROREHAB NEURAL RE, V23, P382, DOI 10.1177/1545968308326427

Rand D, 2014, NEUROREHAB NEURAL RE, V28, P733, DOI 10.1177/1545968314521008

Riecke BE, 2015, FRONT PSYCHOL, V6, DOI 10.3389/fpsyg.2015.00713

Rowe F, 2009, STRABISMUS, V17, P24, DOI 10.1080/09273970802678537

Saposnik G, 2011, STROKE, V42, P1380, DOI 10.1161/STROKEAHA.110.605451

Shin JH, 2016, J NEUROENG REHABIL, V13, DOI 10.1186/s12984-016-0125-x

Stevens JA, 2003, ARCH PHYS MED REHAB, V84, P1090, DOI 10.1016/S0003-9993(03)00042-X

Tretriluxana J, 2013, TOP STROKE REHABIL, V20, P151, DOI 10.1310/tsr2002-151

Turolla A, 2013, J NEUROENG REHABIL, V10, DOI 10.1186/1743-0003-10-85

Veerbeek JM, 2014, PLOS ONE, V9, DOI 10.1371/journal.pone.0087987

Wittmann F, 2016, J NEUROENG REHABIL, V13, DOI 10.1186/s12984-016-0182-1

Wolf SL, 2001, STROKE, V32, P1635, DOI 10.1161/01.STR.32.7.1635

Yamaguchi T, 2012, PRESENCE-TELEOP VIRT, V21, P43, DOI 10.1162/PRES_a_00080

Zhang L, 2003, ARCH PHYS MED REHAB, V84, P1118, DOI 10.1016/S0003-9993(03)00203-X

NR 42

TC 19

Z9 22

U1 0

U2 36

PU IEEE-INST ELECTRICAL ELECTRONICS ENGINEERS INC

PI PISCATAWAY

PA 445 HOES LANE, PISCATAWAY, NJ 08855-4141 USA

SN 1534-4320

EI 1558-0210

J9 IEEE T NEUR SYS REH

JI IEEE Trans. Neural Syst. Rehabil. Eng.

PD JAN

PY 2018

VL 26

IS 1

BP 252

EP 260

DI 10.1109/TNSRE.2017.2771272

PG 9

WC Engineering, Biomedical; Rehabilitation

WE Science Citation Index Expanded (SCI-EXPANDED); Social Science Citation Index (SSCI)

SC Engineering; Rehabilitation

GA FT1ZZ

UT WOS:000422939000028

PM 29324411

DA 2022-06-21

ER

PT J

AU Adams, RJ

Lichter, MD

Krepkovich, ET

Ellington, A

White, M

Diamond, PT

AF Adams, Richard J.

Lichter, Matthew D.

Krepkovich, Eileen T.

Ellington, Allison

White, Marga

Diamond, Paul T.

TI Assessing Upper Extremity Motor Function in Practice of Virtual

Activities of Daily Living

SO IEEE TRANSACTIONS ON NEURAL SYSTEMS AND REHABILITATION ENGINEERING

LA English

DT Article

DE Human computer interaction; human motion tracking; human motor

performance; occupational therapy; patient rehabilitation; virtual

reality

ID INVERSE KINEMATICS; STROKE; REHABILITATION; RECOVERY; VARIABILITY;

RELIABILITY; SENSITIVITY; PERFORMANCE; PLASTICITY; REALITY

AB A study was conducted to investigate the criterion validity of measures of upper extremity (UE) motor function derived during practice of virtual activities of daily living (ADLs). Fourteen hemiparetic stroke patients employed a Virtual Occupational Therapy Assistant (VOTA), consisting of a high-fidelity virtual world and a Kinect T sensor, in four sessions of approximately one hour in duration. An unscented Kalman Filter-based human motion tracking algorithm estimated UE joint kinematics in real-time during performance of virtual ADL activities, enabling both animation of the user's avatar and automated generation of metrics related to speed and smoothness of motion. These metrics, aggregated over discrete sub-task elements during performance of virtual ADLs, were compared to scores from an established assessment of UE motor performance, the Wolf Motor Function Test (WMFT). Spearman's rank correlation analysis indicates a moderate correlation between VOTA-derived metrics and the time-based WMFT assessments, supporting the criterion validity of VOTA measures as a means of tracking patient progress during an UE rehabilitation program that includes practice of virtual ADLs.

C1 [Adams, Richard J.; Lichter, Matthew D.; Krepkovich, Eileen T.] Barron Associates Inc, Charlottesville, VA 22901 USA.

[Ellington, Allison; White, Marga] Univ Virginia Hlth South Rehabil Hosp, Charlottesville, VA 22908 USA.

[Diamond, Paul T.] Univ Virginia, Dept Phys Med & Rehabil, Charlottesville, VA 22908 USA.

RP Adams, RJ (通讯作者)，Barron Associates Inc, Charlottesville, VA 22901 USA.

EM adams@barron-associates.com; lichter@barron-associates.com;

krepkovich@barron-associates.com; allison.ellington@healthsouth.com;

marga.white@healthsouth.com; ptd2m@virginia.edu

RI Coish, Elizabeth/AAU-1245-2020

OI Adams, Richard/0000-0001-5000-119X

FU National Institutes of Health, Eunice Kennedy Shriver National Institute

of Child Health and Human Development [1R43HD071745-01A1]; EUNICE

KENNEDY SHRIVER NATIONAL INSTITUTE OF CHILD HEALTH & HUMAN DEVELOPMENT

[R43HD071745, R44HD071745] Funding Source: NIH RePORTER

FX This work was supported by the National Institutes of Health, Eunice

Kennedy Shriver National Institute of Child Health and Human Development

under Grant 1R43HD071745-01A1.

CR Adams R. J., 2001, HAPTICS E ELECT J HA, V2

Edwards DF, 2006, NEUROREHAB NEURAL RE, V20, P42, DOI 10.1177/1545968305283038

Fluet Gerard G, 2013, Curr Phys Med Rehabil Rep, V1, P9

Garrett P., 2006, PERSPECT HLTH INF MA, V3, P1

Go AS, 2013, HEART DIS STROKE STA

Hallett M, 2001, BRAIN RES REV, V36, P169, DOI 10.1016/S0165-0173(01)00092-3

Hogan N, 2009, J MOTOR BEHAV, V41, P529, DOI 10.3200/35-09-004-RC

Holden MK, 2005, CYBERPSYCHOL BEHAV, V8, P187, DOI 10.1089/cpb.2005.8.187

Johansson BB, 2011, ACTA NEUROL SCAND, V123, P147, DOI 10.1111/j.1600-0404.2010.01417.x

Julier S. I., 1997, P AEROSENSE P 11 INT

Kallmann M, 2008, COMPUT ANIMAT VIRT W, V19, P79, DOI 10.1002/cav.176

Kopp B, 1997, ARCH PHYS MED REHAB, V78, P615, DOI 10.1016/S0003-9993(97)90427-5

LAWTON MP, 1969, GERONTOLOGIST, V9, P9, DOI 10.1093/geront/9.1.9

Lehmann EL., 1998, NONPARAMETRICS STAT

LOWENTHAL M, 1964, LIVES IN DISTRESS

Lum PS, 2009, TOP STROKE REHABIL, V16, P237, DOI 10.1310/tsr1604-237

McLaughlin A, 2012, ERGON DES, V20, P13, DOI 10.1177/1064804611435654

Morris DM, 2001, ARCH PHYS MED REHAB, V82, P750, DOI 10.1053/apmr.2001.23183

Myerson J, 2007, J EXP ANAL BEHAV, V88, P319, DOI 10.1901/jeab.2007.88-319

Nudo RJ, 2003, J REHABIL MED, V35, P7, DOI 10.1080/16501960310010070

Obdrzalek S, 2012, P 34 INT C IEEE ENG

Rand Debbie, 2008, J Neurol Phys Ther, V32, P155, DOI 10.1097/NPT.0b013e31818ee779

Rohrer B, 2004, MOTOR CONTROL, V8, P472, DOI 10.1123/mcj.8.4.472

Rohrer B, 2002, J NEUROSCI, V22, P8297

Rosen J., 2005, P INT C ADV ROB ICAR

Saposnik G, 2011, STROKE, V42, P1380, DOI 10.1161/STROKEAHA.110.605451

Subramanian SK, 2013, NEUROREHAB NEURAL RE, V27, P13, DOI 10.1177/1545968312449695

Taylor MJD, 2011, J REHABIL RES DEV, V48, P1171, DOI 10.1682/JRRD.2010.09.0171

Tolani D, 1996, PRESENCE-TELEOP VIRT, V5, P393, DOI 10.1162/pres.1996.5.4.393

Tretriluxana J, 2013, TOP STROKE REHABIL, V20, P151, DOI 10.1310/tsr2002-151

Wan EA, 2001, ADAPT LEARN SYST SIG, P221

West R, 2002, BRAIN COGNITION, V49, P402, DOI 10.1006/brcg.2001.1507

Wolf SL, 2001, STROKE, V32, P1635, DOI 10.1161/01.STR.32.7.1635

NR 33

TC 29

Z9 31

U1 0

U2 29

PU IEEE-INST ELECTRICAL ELECTRONICS ENGINEERS INC

PI PISCATAWAY

PA 445 HOES LANE, PISCATAWAY, NJ 08855-4141 USA

SN 1534-4320

EI 1558-0210

J9 IEEE T NEUR SYS REH

JI IEEE Trans. Neural Syst. Rehabil. Eng.

PD MAR

PY 2015

VL 23

IS 2

BP 287

EP 296

DI 10.1109/TNSRE.2014.2360149

PG 10

WC Engineering, Biomedical; Rehabilitation

WE Science Citation Index Expanded (SCI-EXPANDED)

SC Engineering; Rehabilitation

GA CD8SC

UT WOS:000351365100015

PM 25265612

OA Green Accepted, hybrid

DA 2022-06-21

ER

PT J

AU Adie, K

Schofield, C

Berrow, M

Wingham, J

Humfryes, J

Pritchard, C

James, M

Allison, R

AF Adie, Katja

Schofield, Christine

Berrow, Margie

Wingham, Jennifer

Humfryes, John

Pritchard, Colin

James, Martin

Allison, Rhoda

TI Does the use of Nintendo Wii Sports(TM) improve arm function? Trial of

Wii(TM) in Stroke: a randomized controlled trial and economics analysis

SO CLINICAL REHABILITATION

LA English

DT Article

DE Stroke; rehabilitation; arm; randomized controlled trial; virtual

reality

ID UPPER EXTREMITY FUNCTION; MOVEMENT THERAPY; UPPER-LIMB; RELIABILITY;

VALIDITY; IMPACT; REHABILITATION; POSTSTROKE; RECOVERY; VERSION

AB Objective: The Trial of Wii in Stroke investigated the efficacy of using the Nintendo Wii Sports (Wii(TM)) to improve affected arm function after stroke.

Design: Multicentre, pragmatic, parallel group, randomized controlled trial.

Setting: Home-based rehabilitation.

Subjects: A total of 240 participants aged 24-90years with arm weakness following a stroke within the previous six months.

Intervention: Participants were randomly assigned to exercise daily for six weeks using the Wii(TM) or arm exercises at home.

Main measures: Primary outcome was change in the affected arm function at six weeks follow-up using the Action Research Arm Test. Secondary outcomes included occupational performance, quality of life, arm function at six months and a cost effectiveness analysis.

Results: The study was completed by 209 participants (87.1%). There was no significant difference in the primary outcome of affected arm function at six weeks follow-up (mean difference -1.7, 95% CI -3.9 to 0.5, p=0.12) and no significant difference in secondary outcomes, including occupational performance, quality of life or arm function at six months, between the two groups. No serious adverse events related to the study treatment were reported. The cost effectiveness analysis showed that the Wii(TM) was more expensive than arm exercises 1106 (SD 1656) vs. 730 pound (SD 829) (probability 0.866).

Conclusion: The trial showed that the Wii(TM) was not superior to arm exercises in home-based rehabilitation for stroke survivors with arm weakness. The Wii(TM) was well tolerated but more expensive than arm exercises.

C1 [Adie, Katja] Royal Cornwall Hosp Trust, Stroke & Eldercare, Cornwall, England.

[Schofield, Christine; Wingham, Jennifer; Pritchard, Colin] Royal Cornwall Hosp Trust, Res Dev & Innovat, Truro, England.

[Berrow, Margie] Univ Plymouth, Peninsula Clin Trials Unit, Sch Med & Dent, Plymouth, Devon, England.

[Wingham, Jennifer] Univ Exeter, Med Sch Primary Care, St Lukes Campus, Exeter, Devon, England.

[James, Martin] Univ Exeter, Royal Devon & Exeter Hosp, Sch Med, Exeter, Devon, England.

[Allison, Rhoda] Torbay & South Devon NHS Fdn Trust, Torquay, Devon, England.

RP Adie, K (通讯作者)，Royal Cornwall Hosp Trust, Cornwall TR1 3LJ, England.

EM k.adie@nhs.net

RI Wingham, Jennifer/B-1311-2017

OI Wingham, Jennifer/0000-0002-3342-1202

FU National Institute for Health Research (NIHR) [PB-PG-0110-20332];

National Institute for Health Research [PB-PG-0110-20332] Funding

Source: researchfish

FX The author(s) disclosed receipt of the following financial support for

the research, authorship, and/or publication of this article: This

article summarizes independent research funded by the National Institute

for Health Research (NIHR) under its Research for Patient Benefit (RfPB)

Programme [Grant Reference Number PB-PG-0110-20332]. The views expressed

are those of the authors and not necessarily those of the NHS, the NIHR

or the Department of Health.

CR Adie K, 2014, INT J GEN MED, V7, P475, DOI 10.2147/IJGM.S65379

BAMFORD J, 1991, LANCET, V337, P1521, DOI 10.1016/0140-6736(91)93206-O

BONITA R, 1988, STROKE, V19, P1497, DOI 10.1161/01.STR.19.12.1497

Chisholm D, 2000, BRIT J PSYCHIAT, V177, pS28, DOI 10.1192/bjp.177.39.s28

Cup EHC, 2003, CLIN REHABIL, V17, P402, DOI 10.1191/0269215503cr635oa

Curtis L., 2010, UNIT COST HLTH SOCIA

Department of Health, 2010, NAT REF COSTS

Dolan P, 1997, MED CARE, V35, P1095, DOI 10.1097/00005650-199711000-00002

Duncan PW, 1999, STROKE, V30, P2131, DOI 10.1161/01.STR.30.10.2131

Eng J, 2012, GUIDELINES MANUAL

French B, 2007, COCHRANE DB SYST REV, DOI 10.1002/14651858.CD006073.pub2

Intercollegiate Stroke Working Party, 2012, NAT CLIN GUID STROK

Kwakkel G, 2003, STROKE, V34, P2181, DOI 10.1161/01.STR.0000087172.16305.CD

Kwakkel G, 2006, DISABIL REHABIL, V28, P823, DOI 10.1080/09638280500534861

Langhorne P, 2009, LANCET NEUROL, V8, P741, DOI 10.1016/S1474-4422(09)70150-4

Lohse KR, 2014, PLOS ONE, V9, DOI 10.1371/journal.pone.0093318

McNulty PA, 2015, INT J STROKE, V10, P1253, DOI 10.1111/ijs.12594

Mouawad MR, 2011, J REHABIL MED, V43, P527, DOI 10.2340/16501977-0816

Nasreddine ZS, 2005, J AM GERIATR SOC, V53, P695, DOI 10.1111/j.1532-5415.2005.53221.x

Nichols-Larsen DS, 2005, STROKE, V36, P1480, DOI 10.1161/01.STR.0000170706.13595.4f

Paternostro-Sluga T, 2008, J REHABIL MED, V40, P665, DOI 10.2340/16501977-0235

Rabin R, 2011, EQ 5D 3L USER GUIDE

Royal college of physicians, 2013, SENT STROK NAT AUD P

Saposnik G, 2010, STROKE, V41, P1477, DOI 10.1161/STROKEAHA.110.584979

Shaughnessy M, 2006, REHABIL NURS, V31, P15, DOI 10.1002/j.2048-7940.2006.tb00005.x

Uswatte G, 2005, STROKE, V36, P2493, DOI 10.1161/01.STR.0000185928.90848.2e

Van der Lee JH, 2001, ARCH PHYS MED REHAB, V82, P14, DOI 10.1053/apmr.2001.18668

Wii Habilitation, 2012, STROK CVA WII

Wingham J, 2015, CLIN REHABIL, V29, P295, DOI 10.1177/0269215514542638

Yozbatiran N, 2008, NEUROREHAB NEURAL RE, V22, P78, DOI 10.1177/1545968307305353

NR 30

TC 50

Z9 52

U1 2

U2 37

PU SAGE PUBLICATIONS LTD

PI LONDON

PA 1 OLIVERS YARD, 55 CITY ROAD, LONDON EC1Y 1SP, ENGLAND

SN 0269-2155

EI 1477-0873

J9 CLIN REHABIL

JI Clin. Rehabil.

PD FEB

PY 2017

VL 31

IS 2

BP 173

EP 185

DI 10.1177/0269215516637893

PG 13

WC Rehabilitation

WE Science Citation Index Expanded (SCI-EXPANDED)

SC Rehabilitation

GA EL6US

UT WOS:000394758500004

PM 26975313

DA 2022-06-21

ER

PT J

AU Adlakha, S

Chhabra, D

Shukla, P

AF Adlakha, Shagun

Chhabra, Deepak

Shukla, Pratyoosh

TI Effectiveness of gamification for the rehabilitation of

neurodegenerative disorders

SO CHAOS SOLITONS & FRACTALS

LA English

DT Article

DE Gamification; Neurodegenerative (ND) disorders; Virtual reality; active

video games; exergames

ID ACTIVE VIDEO GAMES; VIRTUAL-REALITY; COGNITIVE IMPAIRMENT; UPPER-LIMB;

DEMENTIA; DESIGN; ENGAGEMENT; PEOPLE

AB The present review aims to analyze distinctive features of gamification in rehabilitation from neurodegenerative disorders. This work is an effort to decipher various gamified elements which are commonly used for the rehabilitation of patients suffering from neurodegenerative diseases including the scope of these gamified techniques towards their role with traditional methods for recovery of such disorders. Further, gamified tools and techniques used in the treatment process are studied in depth. Moreover, an overview of the inclusion of gamified techniques used in healthcare for neurodegenerative diseases towards enhancing patient engagement is reviewed and modeling of gamification effectiveness using statistical tools have also been discussed. Subsequently, it is found out that the virtual reality games, active video games and serious games are most commonly used in rehabilitation of neurodegenerative diseases. The most used gamified techniques are found to be virtual reality and mobile video games followed by serious games. Moreover, despite some limitations, it is concluded that the gamified methods prove to be a valuable addition to traditional treatments as they enhance patient engagement, socialization, feedback, adherence to the treatment process and provides better health outcomes. Finally, it adds motivation, literacy and helps in diverting patients from painful treatments with effective therapeutics. (c) 2020 Elsevier Ltd. All rights reserved.

C1 [Adlakha, Shagun] Univ Rajasthan, St Xaviers PG Coll, Dept Commerce, Jaipur, Rajasthan, India.

[Chhabra, Deepak] Maharshi Dayanand Univ, Univ Inst Engn & Technol, Dept Mech Engn, Optimizat & Biomech Lab, Rohtak, Haryana, India.

[Shukla, Pratyoosh] Maharshi Dayanand Univ, Dept Microbiol, Enzyme Technol & Prot Bioinformat Lab, Rohtak, Haryana, India.

RP Chhabra, D (通讯作者)，Maharshi Dayanand Univ, Univ Inst Engn & Technol, Dept Mech Engn, Optimizat & Biomech Lab, Rohtak, Haryana, India.; Shukla, P (通讯作者)，Maharshi Dayanand Univ, Dept Microbiol, Enzyme Technol & Prot Bioinformat Lab, Rohtak, Haryana, India.

EM deepaknit10@gmail.com; pratyoosh.shukla@gmail.com

RI Chhabra, Deepak/AAG-3845-2019; Shukla, Pratyoosh/N-8039-2013

OI Chhabra, Deepak/0000-0002-3738-0153; Shukla,

Pratyoosh/0000-0002-9307-4126

CR Al-qaysi ZT, 2018, COMPUT METH PROG BIO, V164, P221, DOI 10.1016/j.cmpb.2018.06.012

Alves S, 2019, 16TH INTERNATIONAL WEB FOR ALL CONFERENCE (WEB4ALL), DOI 10.1145/3315002.3317571

Amieva H, 2015, P 2015 WORKSH VIS AN, DOI DOI 10.1145/2836034.2836037

[Anonymous], 2016, DEV EVALUATING CREAT

Belmonte-Hernandez A, 2019, 12TH ACM INTERNATIONAL CONFERENCE ON PERVASIVE TECHNOLOGIES RELATED TO ASSISTIVE ENVIRONMENTS (PETRA 2019), P535, DOI 10.1145/3316782.3322759

Biddiss E, 2010, ARCH PEDIAT ADOL MED, V164, P664, DOI 10.1001/archpediatrics.2010.104

Bohm M, INT C HUM ASP IT AG, P526

Bondy SC, 2017, ADV NEUROTOXICOL, P131, DOI 10.1016/bs.ant.2017.07.008

Bowers M, 2017, DESIGN LOW COST ROBO

Brettschneider J, 2015, NAT REV NEUROSCI, V16, P109, DOI 10.1038/nrn3887

Burdea G, 2021, ASSIST TECHNOL, V33, P117, DOI 10.1080/10400435.2019.1593260

Choi YH, 2016, RESTOR NEUROL NEUROS, V34, P455, DOI 10.3233/RNN-150626

Cikajlo I, 2017, 2017 INTERNATIONAL CONFERENCE ON VIRTUAL REHABILITATION (ICVR)

Cole JH, 2019, MOL PSYCHIATR, V24, P266, DOI 10.1038/s41380-018-0098-1

Dockx K, 2016, COCHRANE DB SYST REV, DOI 10.1002/14651858.CD010760.pub2

Duraes F, 2018, PHARMACEUTICALS-BASE, V11, DOI 10.3390/ph11020044

Eckert M, 2017, SENSORS-BASEL, V17, DOI 10.3390/s17020354

Elkin JA, 2018, THESIS UCL

Estes LTCT, 2015, INT CONF PER COMP, P37, DOI 10.4108/icst.pervasivehealth.2015.259137

Ferraz HB, 2015, P 3 2015 WORKSH ICTS, P54, DOI [10.1145/2838944.2838958, DOI 10.1145/2838944.2838958]

France CR, 2018, CONTEMP CLIN TRIALS, V69, P83, DOI 10.1016/j.cct.2018.05.001

Gamito P, 2017, DISABIL REHABIL, V39, P385, DOI 10.3109/09638288.2014.934925

Gauthier LV, 2017, BMC NEUROL, V17, DOI 10.1186/s12883-017-0888-0

Geng JS, 2017, COCHRANE DB SYST REV, DOI 10.1002/14651858.CD008557.pub3

Gentry S, 2018, COCHRANE DB SYST REV, V2018

Glaser NJ, 2017, J FORMATIVE DES LEAR, V1, P126, DOI 10.1007/s41686-017-0011-3

Grammatikopoulou A., 2019, P 12 ACM INT C PERVA, P517, DOI [10.1145/3316782.3322756, DOI 10.1145/3316782.3322756]

Grammatikopoulou A, 2019, 12TH ACM INTERNATIONAL CONFERENCE ON PERVASIVE TECHNOLOGIES RELATED TO ASSISTIVE ENVIRONMENTS (PETRA 2019), P523, DOI 10.1145/3316782.3322757

Gravenhorst F, 2015, PERS UBIQUIT COMPUT, V19, P335, DOI 10.1007/s00779-014-0829-5

Griffiths MD, 2014, BEHAVIORAL ADDICTIONS: CRITERIA, EVIDENCE, AND TREATMENT, P119, DOI 10.1016/B978-0-12-407724-9.00006-9

Hammedi W, 2017, J SERV MANAGE, V28, P640, DOI 10.1108/JOSM-04-2016-0116

Harwood T, 2015, J SERV MARK, V29, P533, DOI 10.1108/JSM-01-2015-0045

Hock P., 2018, CATARO ROBOT TELLS C, P1841, DOI [10.1145/3205651.3208264, DOI 10.1145/3205651.3208264]

Huber S, 2019, TEI'19: PROCEEDINGS OF THE THIRTEENTH INTERNATIONAL CONFERENCE ON TANGIBLE, EMBEDDED, AND EMBODIED INTERACTION, P15, DOI 10.1145/3294109.3295632

Isernia S, 2019, FRONT NEUROL, V10, DOI 10.3389/fneur.2019.01206

Jabbari B, 2018, HEMODIAL INT, V22, P150, DOI 10.1111/hdi.12587

Jia Y, 2016, 34TH ANNUAL CHI CONFERENCE ON HUMAN FACTORS IN COMPUTING SYSTEMS, CHI 2016, P2001, DOI 10.1145/2858036.2858515

Johnson TM, 2016, J SCI MED SPORT, V19, P432, DOI 10.1016/j.jsams.2015.05.002

Jung I., 2016, P 4 WORKSH ICTS IMPR, P117, DOI [10.1145/3051488.3051511, DOI 10.1145/3051488.3051511]

Kan V, 2018, PROCEEDINGS OF THE 2018 ACM INTERNATIONAL JOINT CONFERENCE ON PERVASIVE AND UBIQUITOUS COMPUTING AND PROCEEDINGS OF THE 2018 ACM INTERNATIONAL SYMPOSIUM ON WEARABLE COMPUTERS (UBICOMP/ISWC'18 ADJUNCT), P373, DOI 10.1145/3267305.3267598

Kearney EK, 2018, THESIS

Kearney E, 2019, DISABIL REHABIL, V41, P995, DOI 10.1080/09638288.2017.1419292

Khan HA, 2018, CHI 2018: EXTENDED ABSTRACTS OF THE 2018 CHI CONFERENCE ON HUMAN FACTORS IN COMPUTING SYSTEMS, DOI 10.1145/3170427.3188523

Kihe MM, 2017, 2017 INTERNATIONAL CONFERENCE ON COMPUTER SCIENCE AND ENGINEERING (UBMK), P328, DOI 10.1109/UBMK.2017.8093401

Kim O, 2019, BMC PSYCHIATRY, V19, DOI 10.1186/s12888-019-2180-x

Kimppa KK., 2017, P 1 INT GAMIFIN C GA, V1857, P96

Koldrack P, 2015, INT CONF PER COMP, P245, DOI 10.4108/icst.pervasivehealth.2015.259288

Kuosmanen E, 2018, 17TH INTERNATIONAL CONFERENCE ON MOBILE AND UBIQUITOUS MULTIMEDIA (MUM 2018), P441, DOI 10.1145/3282894.3289737

Laver KE, 2011, COCHRANE DB SYST REV, DOI [10.1002/14651858.CD008349.pub2, 10.1002/14651858.CD008349.pub4, 10.1002/14651858.CD008349.pub3]

Libey T., 2016, THESIS

[林志诚 Lin Zhicheng], 2016, [中国康复理论与实践, Chinese Journal of Rehabilitation Theory and Practice], V22, P1059

Lithoxoidou EE, 2018, 11TH ACM INTERNATIONAL CONFERENCE ON PERVASIVE TECHNOLOGIES RELATED TO ASSISTIVE ENVIRONMENTS (PETRA 2018), P482, DOI 10.1145/3197768.3201561

Lopez JP, 2018, 11TH ACM INTERNATIONAL CONFERENCE ON PERVASIVE TECHNOLOGIES RELATED TO ASSISTIVE ENVIRONMENTS (PETRA 2018), P428, DOI 10.1145/3197768.3201553

Luckett P, 2019, ACM-BCB'19: PROCEEDINGS OF THE 10TH ACM INTERNATIONAL CONFERENCE ON BIOINFORMATICS, COMPUTATIONAL BIOLOGY AND HEALTH INFORMATICS, P190, DOI 10.1145/3307339.3342131

Maggio MG, 2019, J NATL MED ASSOC, V111, P457, DOI 10.1016/j.jnma.2019.01.003

Manera V, 2017, FRONT PSYCHOL, V8, DOI 10.3389/fpsyg.2017.01243

McColl-Kennedy JR, 2017, J SERV MANAGE, V28, P2, DOI 10.1108/JOSM-01-2016-0018

Mekki M, 2018, NEUROTHERAPEUTICS, V15, P604, DOI 10.1007/s13311-018-0642-3

Mekler ED, 2017, COMPUT HUM BEHAV, V71, P525, DOI 10.1016/j.chb.2015.08.048

Mendez MF, 2015, DISABIL REHABIL-ASSI, V10, P160, DOI 10.3109/17483107.2014.889230

Moreira MC, 2017 INT C VIRT REH, P1, DOI [10.1109/ICVR.2017.8007540, DOI 10.1109/ICVR.2017.8007540]

Mubin Omar, 2019, JMIR Rehabil Assist Technol, V6, pe12010, DOI 10.2196/12010

Muhlhaus J, 2017, LECT NOTES COMPUT SC, V10279, P76, DOI 10.1007/978-3-319-58700-4_7

O'Neil O, 2018, PM&R, V10, pS198, DOI 10.1016/j.pmrj.2018.08.375

Osvath Peter, 2017, Psychiatr Hung, V32, P437

Paletta L, 2018, 11TH ACM INTERNATIONAL CONFERENCE ON PERVASIVE TECHNOLOGIES RELATED TO ASSISTIVE ENVIRONMENTS (PETRA 2018), P237, DOI 10.1145/3197768.3197789

Perez-Marcos D, 2018, FRONT PSYCHOL, V9, DOI 10.3389/fpsyg.2018.02120

Piau A, 2019, J MED INTERNET RES, V21, DOI 10.2196/12785

Potisk KP, 2016, P 4 WORKSH ICTS IMPR, P17, DOI [10.1145/ 3051488.3051493, DOI 10.1145/3051488.3051493]

Power V, 2016, P 9 ACM INT C PERV T, DOI 10.1145/2910674.2935827

Radovick S, 2018, THE J, V1, P71

Rahim MIA, 2017, SEIZURE-EUR J EPILEP, V52, P11, DOI 10.1016/j.seizure.2017.09.008

Rastogi M, 2019, APPL GAMING NEW MEDI, P244, DOI [10.4018/978- 1- 5225-6064-7. ch015, DOI 10.4018/978-1-5225-6064-7.CH015]

Riggare S, 2019, HEALTH INFORM J, V25, P91, DOI 10.1177/1460458217704248

Robert PH, 2014, FRONT AGING NEUROSCI, V6, DOI 10.3389/fnagi.2014.00054

Samuel GS, 2017, SINGAP MED J, V58, P610, DOI 10.11622/smedj.2016111

Saposnik G, 2016, LANCET NEUROL, V15, P1019, DOI 10.1016/S1474-4422(16)30121-1

Sardi L, 2017, J BIOMED INFORM, V71, P31, DOI 10.1016/j.jbi.2017.05.011

Siriaraya P, 2017, PROC INT CONF INF TE, P109

Spyridonis F, 2017, INT CONF GAMES VIRTU, P233, DOI 10.1109/VS-GAMES.2017.8056606

Stowell Elizabeth, 2019, Proceedings of the ACM on Human-Computer Interaction, V3, DOI 10.1145/3359232

Tidoni E, 2017, IEEE T NEUR SYS REH, V25, P1622, DOI 10.1109/TNSRE.2016.2626391

Tong T, 2017, WWW'17 COMPANION: PROCEEDINGS OF THE 26TH INTERNATIONAL CONFERENCE ON WORLD WIDE WEB, P1111, DOI 10.1145/3041021.3054930

Tudor S, 2015, 8TH ACM INTERNATIONAL CONFERENCE ON PERVASIVE TECHNOLOGIES RELATED TO ASSISTIVE ENVIRONMENTS (PETRA 2015), DOI 10.1145/2769493.2769593

Tzallas AT, 2018, 11TH ACM INTERNATIONAL CONFERENCE ON PERVASIVE TECHNOLOGIES RELATED TO ASSISTIVE ENVIRONMENTS (PETRA 2018), P476, DOI 10.1145/3197768.3201560

Ungurean OC, 2018, CHI 2018: EXTENDED ABSTRACTS OF THE 2018 CHI CONFERENCE ON HUMAN FACTORS IN COMPUTING SYSTEMS, DOI 10.1145/3170427.3188619

Valladares-Rodriguez S, 2017, PEERJ, V5, DOI 10.7717/peerj.3508

van de Weijer SCF, 2019, JMIR SERIOUS GAMES, V7, DOI 10.2196/12130

van der Kolk NM, 2019, LANCET NEUROL, V18, P998, DOI 10.1016/S1474-4422(19)30285-6

Vashistha R., 2019, LEVERAGING BIOMEDICA, P77, DOI DOI 10.1016/B978-0-12-809556-0.00005-8

Vashistha R, 2018, INDIAN J MICROBIOL, V58, P252, DOI 10.1007/s12088-018-0708-2

Weerakoon DSD, 2018, PROCEDIA COMPUT SCI, V141, P413, DOI 10.1016/j.procs.2018.10.175

Wilkinson A, 2017, WWW'17 COMPANION: PROCEEDINGS OF THE 26TH INTERNATIONAL CONFERENCE ON WORLD WIDE WEB, P1103, DOI 10.1145/3041021.3054933

Wolf D, 2018, ADJUNCT PUBLICATION OF THE 31ST ANNUAL ACM SYMPOSIUM ON USER INTERFACE SOFTWARE AND TECHNOLOGY (UIST'18 ADJUNCT), P42, DOI 10.1145/3266037.3266095

Xu FF, 2016, J TRAVEL TOUR MARK, V33, P1124, DOI 10.1080/10548408.2015.1093999

Zeng N, 2017, J SPORT HEALTH SCI, V6, P33, DOI 10.1016/j.jshs.2016.12.002

Zeng ZW, 2018, PROCEEDINGS OF THE 3RD INTERNATIONAL CONFERENCE ON CROWD SCIENCE AND ENGINEERING (ICCSE 2018), DOI 10.1145/3265689.3265719

NR 97

TC 4

Z9 4

U1 2

U2 7

PU PERGAMON-ELSEVIER SCIENCE LTD

PI OXFORD

PA THE BOULEVARD, LANGFORD LANE, KIDLINGTON, OXFORD OX5 1GB, ENGLAND

SN 0960-0779

EI 1873-2887

J9 CHAOS SOLITON FRACT

JI Chaos Solitons Fractals

PD NOV

PY 2020

VL 140

AR 110192

DI 10.1016/j.chaos.2020.110192

PG 11

WC Mathematics, Interdisciplinary Applications; Physics, Multidisciplinary;

Physics, Mathematical

WE Science Citation Index Expanded (SCI-EXPANDED)

SC Mathematics; Physics

GA PB4QW

UT WOS:000596308200012

DA 2022-06-21

ER

PT J

AU Adomaviciene, A

Daunoraviciene, K

Kubilius, R

Varzaityte, L

Raistenskis, J

AF Adomaviciene, Ausra

Daunoraviciene, Kristina

Kubilius, Raimondas

Varzaityte, Lina

Raistenskis, Juozas

TI Influence of New Technologies on Post-Stroke Rehabilitation: A

Comparison of Armeo Spring to the Kinect System

SO MEDICINA-LITHUANIA

LA English

DT Article

DE stroke rehabilitation; hand motor function; cognitive recovery; Kinect;

Armeo

ID VIRTUAL-REALITY; STROKE PATIENTS

AB Background: New technologies to improve post-stroke rehabilitation outcomes are of great interest and have a positive impact on functional, motor, and cognitive recovery. Identifying the most effective rehabilitation intervention is a recognized priority for stroke research and provides an opportunity to achieve a more desirable effect. Objective: The objective is to verify the effect of new technologies on motor outcomes of the upper limbs, functional state, and cognitive functions in post-stroke rehabilitation. Methods: Forty two post-stroke patients (8.69 +/- 4.27 weeks after stroke onset) were involved in the experimental study during inpatient rehabilitation. Patients were randomly divided into two groups: conventional programs were combined with the Armeo Spring robot-assisted trainer (Armeo group; n = 17) and the Kinect-based system (Kinect group; n = 25). The duration of sessions with the new technological devices was 45 min/day (10 sessions in total). Functional recovery was compared among groups using the Functional Independence Measure (FIM), and upper limbs' motor function recovery was compared using the Fugl-Meyer Assessment Upper Extremity (FMA-UE), Modified Ashworth Scale (MAS), Hand grip strength (dynamometry), Hand Tapping test (HTT), Box and Block Test (BBT), and kinematic measures (active Range Of Motion (ROM)), while cognitive functions were assessed by the MMSE (Mini-Mental State Examination), ACE-R (Addenbrooke's Cognitive Examination-Revised), and HAD (Hospital Anxiety and Depression Scale) scores. Results: Functional independence did not show meaningful differences in scores between technologies (p > 0.05), though abilities of self-care were significantly higher after Kinect-based training (p < 0.05). The upper limbs' kinematics demonstrated higher functional recovery after robot training: decreased muscle tone, improved shoulder and elbow ROMs, hand dexterity, and grip strength (p < 0.05). Besides, virtual reality games involve more arm rotation and performing wider movements. Both new technologies caused an increase in overall global cognitive changes, but visual constructive abilities (attention, memory, visuospatial abilities, and complex commands) were statistically higher after robotic therapy. Furthermore, decreased anxiety level was observed after virtual reality therapy (p < 0.05). Conclusions: Our study displays that even a short-term, two-week training program with new technologies had a positive effect and significantly recovered post-strokes functional level in self-care, upper limb motor ability (dexterity and movements, grip strength, kinematic data), visual constructive abilities (attention, memory, visuospatial abilities, and complex commands) and decreased anxiety level.

C1 [Adomaviciene, Ausra; Raistenskis, Juozas] Vilnius Univ, Fac Med, Dept Rehabil Phys & Sports Med, Santariskiu G 2, LT-08661 Vilnius, Lithuania.

[Daunoraviciene, Kristina] Vilnius Gediminas Tech Univ, Dept Biomech Engn, J Basanaviciaus G 28, LT-03224 Vilnius, Lithuania.

[Kubilius, Raimondas; Varzaityte, Lina] Lithuanian Univ Hlth Sci, Rehabil Dept, Eiveniu G 2, LT-50161 Kaunas, Lithuania.

RP Adomaviciene, A (通讯作者)，Vilnius Univ, Fac Med, Dept Rehabil Phys & Sports Med, Santariskiu G 2, LT-08661 Vilnius, Lithuania.

EM ausra.adomaviciene@gmail.com; kristina.daunoraviciene@vgtu.lt;

raimondas.kubilius@kaunoklinikos.lt; lina.varzaityte@gmail.com;

juozas.raistenskis@santa.lt

OI Daunoraviciene, Kristina/0000-0003-0898-4860; Adomaviciene,

Ausra/0000-0002-5946-6265

CR Burton CAC, 2013, INT J STROKE, V8, P545, DOI 10.1111/j.1747-4949.2012.00906.x

Calabro RS, 2016, PM&R, V8, P971, DOI 10.1016/j.pmrj.2016.02.004

Colomer C, 2013, NEUROLOGIA, V28, P261, DOI 10.1016/j.nrl.2012.04.017

EMERY CF, 1991, CHEST, V100, P613, DOI 10.1378/chest.100.3.613

Fiedorova D, 2018, NEUROPSYCHIATRY-LOND, V8, P505

Franceschini M, 2018, PLOS ONE, V13, DOI 10.1371/journal.pone.0193235

Gamito P, 2017, DISABIL REHABIL, V39, P385, DOI 10.3109/09638288.2014.934925

Gassert R, 2018, J NEUROENG REHABIL, V15, DOI 10.1186/s12984-018-0383-x

Gijbels D, 2011, J NEUROENG REHABIL, V8, DOI 10.1186/1743-0003-8-5

Huang VS, 2009, J NEUROENG REHABIL, V6, DOI 10.1186/1743-0003-6-5

Kim YM, 2011, ANN REHABIL MED-ARM, V35, P309, DOI 10.5535/arm.2011.35.3.309

Kiper P, 2018, ARCH PHYS MED REHAB, V99, P834, DOI 10.1016/j.apmr.2018.01.023

Klamroth-Marganska V, 2014, LANCET NEUROL, V13, P159, DOI 10.1016/S1474-4422(13)70305-3

Langhorne P, 2003, J NEUROL NEUROSUR PS, V74, P18

Levin MF, 2009, NEUROREHAB NEURAL RE, V23, P313, DOI 10.1177/1545968308328727

Liao W-w, 2018, COGENT MED, V5, DOI [10.1080/2331205X.2018.1428038, DOI 10.1080/2331205X.2018.1428038]

Masiero S, 2011, J REHABIL RES DEV, V48, P355, DOI 10.1682/JRRD.2010.04.0063

Mehrholz J, 2012, COCHRANE DB SYST REV, DOI [10.1002/14651858.CD006876.pub3, 10.1002/14651858.CD006876.pub4]

OCZKOWSKI WJ, 1993, ARCH PHYS MED REHAB, V74, P1291, DOI 10.1016/0003-9993(93)90081-K

Orihuela-Espina F, 2016, J HAND THER, V29, P51, DOI 10.1016/j.jht.2015.11.006

Pangman VC, 2000, APPL NURS RES, V13, P209, DOI 10.1053/apnr.2000.9231

Pool SM, 2016, ASSIST TECHNOL, V28, P225, DOI 10.1080/10400435.2016.1167789

Rose FD, 2005, CYBERPSYCHOL BEHAV, V8, P241, DOI 10.1089/cpb.2005.8.241

Scale R., 2004, TIME, DOI [10.1124/jpet.106.103382.oped, DOI 10.1124/JPET.106.103382.OPED]

Subramanian SK, 2010, NEUROREHAB NEURAL RE, V24, P113, DOI 10.1177/1545968309349941

Webster D, 2014, J NEUROENG REHABIL, V11, DOI 10.1186/1743-0003-11-108

NR 26

TC 24

Z9 25

U1 10

U2 28

PU MDPI

PI BASEL

PA ST ALBAN-ANLAGE 66, CH-4052 BASEL, SWITZERLAND

SN 1010-660X

EI 1648-9144

J9 MEDICINA-LITHUANIA

JI Med. Lith.

PD APR

PY 2019

VL 55

IS 4

AR 98

DI 10.3390/medicina55040098

PG 12

WC Medicine, General & Internal

WE Science Citation Index Expanded (SCI-EXPANDED)

SC General & Internal Medicine

GA HX3RB

UT WOS:000467308800018

PM 30970655

OA Green Submitted, Green Published, gold

DA 2022-06-21

ER

PT J

AU Afifi, T

Collins, NL

Rand, K

Fujiwara, K

Mazur, A

Otmar, C

Dunbar, NE

Harrison, K

Logsdon, R

AF Afifi, Tamara

Collins, Nancy L.

Rand, Kyle

Fujiwara, Ken

Mazur, Allison

Otmar, Chris

Dunbar, Norah E.

Harrison, Kathryn

Logsdon, Rebecca

TI Testing the Feasibility of Virtual Reality With Older Adults With

Cognitive Impairments and Their Family Members Who Live at a Distance

SO INNOVATION IN AGING

LA English

DT Article

DE Dementia; Family relationships; Livestreaming; Networking; Virtual

reality

ID QUALITY-OF-LIFE; SOCIAL PRESENCE; DEMENTIA; PEOPLE; TECHNOLOGY

AB Background and Objectives: This study tests the feasibility of using virtual reality (VR) with older adults with mild cognitive impairment (MCI) or mild-to-moderate dementia with a family member who lives at a distance.

Research Design and Methods: 21 residents in a senior living community and a family member (who participated in the VR with the older adult from a distance) engaged in a baseline telephone call, followed by 3 weekly VR sessions.

Results: Residents and family members alike found the VR safe, extremely enjoyable, and easy to use. The VR was also acceptable and highly satisfying for residents with MCI and dementia. Human and automated coding revealed that residents were more conversationally and behaviorally engaged with their family member in the VR sessions compared to the baseline telephone call and in the VR sessions that used reminiscence therapy. The results also illustrate the importance of using multiple methods to assess engagement. Residents with dementia reported greater immersion in the VR than residents with MCI. However, the automated coding indicated that residents with MCI were more kinesically engaged while using the VR than residents with dementia.

Discussion and Implications: Combining networking and livestreaming features in a single VR platform can allow older adults in senior living communities to still travel, relive their past, and engage fully with life with their family members, despite geographical separation and physical and cognitive challenges.

C1 [Afifi, Tamara; Mazur, Allison; Otmar, Chris; Dunbar, Norah E.] Univ Calif Santa Barbara, Dept Commun, 4119 SS & MS Bldg, Santa Barbara, CA 93106 USA.

[Collins, Nancy L.] Univ Calif Santa Barbara, Dept Psychol & Brain Sci, Santa Barbara, CA 93106 USA.

[Rand, Kyle] Rendever, Boston, MA USA.

[Fujiwara, Ken] Natl Chung Cheng Univ, Dept Psychol, Taipei, Taiwan.

[Harrison, Kathryn] Blizzard Entertainment, Corp Applicat, Irvine, CA USA.

[Logsdon, Rebecca] Univ Washington, Dept Psychosocial & Community Hlth, Seattle, WA 98195 USA.

RP Afifi, T (通讯作者)，Univ Calif Santa Barbara, Dept Commun, 4119 SS & MS Bldg, Santa Barbara, CA 93106 USA.

EM tafifi@ucsb.edu

OI Mazur, Allison/0000-0001-5491-9192

FU National Institute on Aging [1R41AG063640-01A1]

FX This study was funded by the National Institute on Aging

(1R41AG063640-01A1).

CR Allore HG, 2020, J AM GERIATR SOC, V68, pS68, DOI 10.1111/jgs.16616

Angelini L., 2015, UBICOMPISWC, V15, P7, DOI [https://doi.org/10.1024/1662-9647/a000159, DOI 10.1024/1662-9647/A000159]

Appel L, 2020, PILOT FEASIBILITY ST, V6, DOI 10.1186/s40814-020-00708-9

Appel L, 2020, FRONT MED-LAUSANNE, V6, DOI 10.3389/fmed.2019.00329

Benoit M, 2015, NEUROPSYCH DIS TREAT, V11, P557, DOI 10.2147/NDT.S73179

Biocca F, 2003, PRESENCE-VIRTUAL AUG, V12, P456, DOI 10.1162/105474603322761270

Blascovich J., 2011, INFINITE REALITY AVA

Burgener SC, 2015, DEMENTIA-LONDON, V14, P609, DOI 10.1177/1471301213504202

BURGOON JK, 1984, COMMUN MONOGR, V51, P193, DOI 10.1080/03637758409390195

Cagle JG, 2012, J GERONTOL SOC WORK, V55, P682, DOI 10.1080/01634372.2012.703763

Cao Z, 2017, PROC CVPR IEEE, P1302, DOI 10.1109/CVPR.2017.143

Chapoulie E, 2014, 2014 IEEE VIRTUAL REALITY (VR), P45, DOI 10.1109/VR.2014.6802049

Cummings JJ, 2016, MEDIA PSYCHOL, V19, P272, DOI 10.1080/15213269.2015.1015740

Eriksen S, 2016, DEMENT GERIATR COGN, V42, P342, DOI 10.1159/000452404

Ferguson C, 2020, AM J HOSP PALLIAT ME, V37, P809, DOI 10.1177/1049909120901525

Grinsted A, 2004, NONLINEAR PROC GEOPH, V11, P561, DOI 10.5194/npg-11-561-2004

Guerrero LK, 2005, SOURCEBOOK OF NONVERBAL MEASURES: GOING BEYOND WORDS, P221

Harms C., 2004, P 7 ANN INT WORKSH P, P246

Jensen M. L., 2016, AIS T HUMAN COMPUT I, V8, P1, DOI DOI 10.17705/1THCI.00076

Koenig TL, 2014, QUAL SOC WORK, V13, P335, DOI 10.1177/1473325013475468

Lazar A, 2014, HEALTH EDUC BEHAV, V41, p51S, DOI 10.1177/1090198114537067

Lin CX, 2018, LECT NOTES COMPUT SC, V10927, P89, DOI 10.1007/978-3-319-92037-5_8

Maani CV, 2011, J TRAUMA, V71, pS125, DOI 10.1097/TA.0b013e31822192e2

Manera V, 2016, PLOS ONE, V11, DOI 10.1371/journal.pone.0151487

McEwen D, 2014, J REHABIL RES DEV, V51, P1069, DOI 10.1682/JRRD.2013.10.0231

Moyle W, 2018, GERONTOLOGIST, V58, P478, DOI 10.1093/geront/gnw270

Moyle W, 2011, AGING MENT HEALTH, V15, P970, DOI 10.1080/13607863.2011.583620

National Institutes of Health, 2017, WHAT IS ALZH DIS

Nowak KL, 2003, PRESENCE-TELEOP VIRT, V12, P481, DOI 10.1162/105474603322761289

Nunamaker, 2011, AIS T HUMAN COMPUTER, V3, P62, DOI [10.17705/1thci.00027, DOI 10.17705/1THCI.00027]

Optale G, 2010, NEUROREHAB NEURAL RE, V24, P348, DOI 10.1177/1545968309353328

Repetto C., 2016, OPINION, V7, P1, DOI [10.3389/ fpsyg.2016.01839, DOI 10.3389/FPSYG.2016.01839]

Sayma M, 2020, GERONTOLOGIST, V60, pE502, DOI 10.1093/geront/gnz132

Sheehan OC, 2021, GERONTOLOGIST, V61, P670, DOI 10.1093/geront/gnaa108

Siverova J, 2018, INT J MENT HEALTH NU, V27, P1430, DOI 10.1111/inm.12442

Sury L, 2013, INT PSYCHOGERIATR, V25, P867, DOI 10.1017/S1041610213000057

Thompsell A, 2002, INT J GERIATR PSYCH, V17, P804, DOI 10.1002/gps.692

Tolea MI, 2016, ALZ DIS ASSOC DIS, V30, P60, DOI 10.1097/WAD.0000000000000091

Umberson D, 2010, ANNU REV SOCIOL, V36, P139, DOI 10.1146/annurev-soc-070308-120011

Vertesi A, 2001, CAN FAM PHYSICIAN, V47, P2018

NR 40

TC 2

Z9 2

U1 7

U2 7

PU OXFORD UNIV PRESS

PI OXFORD

PA GREAT CLARENDON ST, OXFORD OX2 6DP, ENGLAND

EI 2399-5300

J9 INNOV AGING

JI Innov. Aging

PY 2021

VL 5

IS 2

AR igab014

DI 10.1093/geroni/igab014

PG 14

WC Geriatrics & Gerontology; Gerontology

WE Science Citation Index Expanded (SCI-EXPANDED); Social Science Citation Index (SSCI)

SC Geriatrics & Gerontology

GA XT8FK

UT WOS:000733816300003

PM 34632105

OA gold, Green Published

DA 2022-06-21

ER

PT J

AU Afridi, A

Rathore, FA

Nazir, SNB

AF Afridi, Ayesha

Rathore, Farooq Azam

Nazir, Shaikh Nabi Bukhsh

TI Wii Fit for Balance Training in Elderly: A Systematic Review

SO JCPSP-JOURNAL OF THE COLLEGE OF PHYSICIANS AND SURGEONS PAKISTAN

LA English

DT Review

DE Postural balance; Rehabilitation; Video games; Virtual reality;

Evidence-based medicine; Technology

ID ASSISTED LIVING RESIDENTS; QUALITY-OF-LIFE; OLDER-ADULTS; EXERCISE

INTERVENTION; STANDING BALANCE; POSTURAL CONTROL; FALL PREVENTION; VIDEO

GAMES; BOARD; RELIABILITY

AB Falls due to poor balance are common in elderly people and can cause fractures, head injury, soft tissue trauma; and may even result in death from these complications. Balance training is one of the strategies used to prevent falls among the elderly population. Wii Fit is a new technological approach of balance training in the community dwelling elderly. The primary aim of this systematic review is to study the effectiveness of Wii Fit as a balance-training tool in older adults using various balance and fall risk assessment scales as outcome measures. Using selected keywords in English only, online literature search was conducted from 2009 to 2019. The initial search resulted in 312 articles. After screening, 14 full text articles were included for the final review and qualitative analysis. There is evidence that Wii Fit plus is a useful, cost-effective, user-friendly, less time consuming, home-based approach for reducing the risk of fall and improving the balance and physical performance in older adults. There are few or no adverse events of using Wii Fit. The commonly used outcome measures were 8 foot up and go test, activities-specific balance confidence scale, Berg balance scale, falls efficacy scale, postural sway, times up and go test and static and dynamic balance assessment tool.

C1 [Afridi, Ayesha] Riphah Int Univ, Fac Rehabil & Allied Hlth Sci, Islamabad, Pakistan.

[Rathore, Farooq Azam] PNS Shifa Hosp, Dept Rehabil Med, Karachi, Pakistan.

[Nazir, Shaikh Nabi Bukhsh] Dow Univ Hlth Sci, Dept Physiotherapy, IPMR, Karachi, Pakistan.

RP Rathore, FA (通讯作者)，PNS Shifa Hosp, Dept Rehabil Med, Karachi, Pakistan.

EM farooqrathore@gmail.com

RI NAZIR, NABI BUKHSH/ABC-4902-2020; Afridi, Ayesha/AAW-9192-2020

OI NAZIR, NABI BUKHSH/0000-0002-4409-5465; Afridi,

Ayesha/0000-0002-8012-0613

CR Afridi A, 2015, PAK J NEUROL SCI, V10, P5

Afridi A, 2018, J PAK MED ASSOC, V68, P480

Aman Joshua E, 2014, Front Hum Neurosci, V8, P1075, DOI 10.3389/fnhum.2014.01075

Baldan AMS, 2014, GAIT POSTURE, V40, P1, DOI 10.1016/j.gaitpost.2013.12.028

Benzinger P, 2015, OSTEOPOROSIS INT, V26, P1341, DOI 10.1007/s00198-014-3005-x

Bonnechere B, 2016, INT J REHABIL RES, V39, P277, DOI 10.1097/MRR.0000000000000190

Chang WD, 2013, J PHYS THER SCI, V25, P1251, DOI 10.1589/jpts.25.1251

Chao YY, 2015, CLIN NURS RES, V24, P589, DOI 10.1177/1054773814562880

Chao YY, 2013, GERIATR NURS, V34, P377, DOI 10.1016/j.gerinurse.2013.05.006

Cho GH, 2014, J PHYS THER SCI, V26, P615, DOI 10.1589/jpts.26.615

Clark RA, 2018, GAIT POSTURE, V61, P40, DOI 10.1016/j.gaitpost.2017.12.022

di Ruffano LF, 2017, BMC MED RES METHODOL, V17, DOI 10.1186/s12874-016-0287-z

Ebell MH, 2004, AM FAM PHYSICIAN, V69, P548

Franco JR, 2012, TECHNOL HEALTH CARE, V20, P95, DOI 10.3233/THC-2011-0661

Fu AS, 2015, ARCH PHYS MED REHAB, V96, P2096, DOI 10.1016/j.apmr.2015.08.427

Goble DJ, 2014, J NEUROENG REHABIL, V11, DOI 10.1186/1743-0003-11-12

Gondim Ihana Thaís Guerra de Oliveira, 2017, Fisioter. mov., V30, P11, DOI 10.1590/1980-5918.030.s01.ao01

Granacher U, 2013, SPORTS MED, V43, P627, DOI 10.1007/s40279-013-0041-1

Hewston P, 2016, CAN J DIABETES, V40, P6, DOI 10.1016/j.jcjd.2015.08.005

Hoffmann TC, 2014, BMJ-BRIT MED J, V348, DOI [10.1136/bmj.g1687, 10.1055/s-0041-111066]

Jorgensen MG, 2013, J GERONTOL A-BIOL, V68, P845, DOI 10.1093/gerona/gls222

Kappen DL, 2019, INT J HUM-COMPUT INT, V35, P140, DOI 10.1080/10447318.2018.1441253

KING MB, 1995, J AM GERIATR SOC, V43, P1146, DOI 10.1111/j.1532-5415.1995.tb07017.x

Kumar A, 2014, AGE AGEING, V43, P76, DOI 10.1093/ageing/aft154

Larsen LH, 2013, GAMES HEALTH J, V2, P205, DOI 10.1089/g4h.2013.0036

Lee A, 2014, ACT ADAPT AGING, V38, P53, DOI 10.1080/01924788.2013.878874

Lee Y, 2017, J AGING PHYS ACTIV, V25, P621, DOI 10.1123/japa.2015-0271

Lin MR, 2007, J AM GERIATR SOC, V55, P499, DOI 10.1111/j.1532-5415.2007.01146.x

Maillot P, 2012, PSYCHOL AGING, V27, P589, DOI 10.1037/a0026268

Maixnerov A. E., 2017, J PHYS ED SPORT, V17, P735, DOI DOI 10.7752/JPES.2017.02111

Markovic G, 2015, ARCH GERONTOL GERIAT, V61, P117, DOI 10.1016/j.archger.2015.05.009

Medicine OCfE- B, 2009, LEVELS EVIDENCE

Merriman NA, 2015, COMPUT HUM BEHAV, V45, P192, DOI 10.1016/j.chb.2014.12.017

Moher D, 2009, BMJ-BRIT MED J, V339, DOI [10.1136/bmj.i4086, 10.1136/bmj.b2535, 10.1136/bmj.g7647, 10.1016/j.ijsu.2010.02.007]

Morone G, 2016, AGING CLIN EXP RES, V28, P1187, DOI 10.1007/s40520-016-0578-6

Ni M, 2014, ARCH PHYS MED REHAB, V95, P1620, DOI 10.1016/j.apmr.2014.04.022

Nicholson VP, 2015, J AGING PHYS ACTIV, V23, P153, DOI [10.1123/japa.2013-0148, 10.1123/JAPA.2013-0148]

Nintendo, 2020, NINT HIST

Padala Kalpana P, 2012, J Aging Res, V2012, P597573, DOI 10.1155/2012/597573

Pardasaney PK, 2013, PHYS THER, V93, P1351, DOI 10.2522/ptj.20130028

Park EC, 2015, J PHYS THER SCI, V27, P1157, DOI 10.1589/jpts.27.1157

Pluchino A, 2012, ARCH PHYS MED REHAB, V93, P1138, DOI 10.1016/j.apmr.2012.01.023

Ray C, 2012, ACT ADAPT AGING, V36, P227, DOI 10.1080/01924788.2012.696236

Rendon AA, 2012, AGE AGEING, V41, P549, DOI 10.1093/ageing/afs053

Scaglioni-Solano P, 2014, INT J REHABIL RES, V37, P138, DOI 10.1097/MRR.0000000000000046

Sienko KH, 2017, J VESTIBUL RES-EQUIL, V27, P63, DOI 10.3233/VES-170606

Singh DKA, 2013, CLIMACTERIC, V16, P141, DOI 10.3109/13697137.2012.664832

Singh DKA, 2012, MATURITAS, V73, P239, DOI 10.1016/j.maturitas.2012.07.011

Toulotte C, 2012, CLIN REHABIL, V26, P827, DOI 10.1177/0269215511434996

Tripette J, 2017, PEERJ, V5, DOI 10.7717/peerj.3600

Whyatt C, 2015, GAMES HEALTH J, V4, P423, DOI 10.1089/g4h.2015.0006

NR 51

TC 2

Z9 2

U1 6

U2 11

PU COLL PHYSICIANS & SURGEONS PAKISTAN

PI KARACHI

PA SEVENTH CENTRAL ST, DEFENCE HOUSING AUTHORITY, KARACHI, 75500, PAKISTAN

SN 1022-386X

EI 1681-7168

J9 JCPSP-J COLL PHYSICI

JI JCPSP-J. Coll. Physicians Surg.

PD MAY

PY 2021

VL 31

IS 5

BP 559

EP 566

DI 10.29271/jcpsp.2021.05.559

PG 8

WC Medicine, General & Internal

WE Science Citation Index Expanded (SCI-EXPANDED)

SC General & Internal Medicine

GA RZ4PV

UT WOS:000648580000014

PM 34027869

OA gold

DA 2022-06-21

ER

PT J

AU Afsar, SI

Mirzayev, I

Yemisci, OU

Saracgil, SNC

AF Afsar, Sevgi Ikbali

Mirzayev, Ilkin

Yemisci, Oya Umit

Saracgil, Sacide Nur Cosar

TI Virtual Reality in Upper Extremity Rehabilitation of Stroke Patients: A

Randomized Controlled Trial

SO JOURNAL OF STROKE & CEREBROVASCULAR DISEASES

LA English

DT Article

DE Hemiplegia; stroke rehabilitation; upper extremity; virtual reality

ID FUNCTIONAL INDEPENDENCE MEASURE; BLOCK TEST; RELIABILITY; GAMES;

RECOVERY; KINECT; BOX

AB Objective: Virtual reality game system is one of novel approaches, which can improve hemiplegic extremity functions of stroke patients. We aimed to evaluate the effect of the Microsoft Xbox 360 Kinect video game system on upper limb motor functions for subacute stroke patients. Methods: The study included 42 stroke patients of which 35 (19 Virtual reality group, 16 control group) completed the study. All patients received 60 minutes of conventional therapy for upper extremity, 5 times per-week for 4 weeks. Virtual reality group additionally received Xbox Kinect game system 30 minutes per-day. Patients were evaluated prior to the rehabilitation and at the end of 4 weeks. Box&Block Test, Functional independence measure self-care score, Brunnstorm stage and Fugl-Meyer upper extremity motor function scale were used as outcome measures. Results: The Brunnstrom stages and the scores on the Fugl-Meyer upper extremity, Box&Block Test and Functional independence measure improved significantly from baseline to post-treatment in both the experimental and the control groups. The Brunnstrom stage-upper extremity and Box&Block Test gain for the experimental group were significantly higher compared to the control group, while the Brunnstrom stage-hand, the Functional independence measure gain and Fugl-Meyer gain were similar between the groups. Conclusions: We found evidence that kinect-based game system in addition to conventional therapy may have supplemental benefit for stroke patients. However, for virtual reality game systems to enter the routine practice of stroke rehabilitation, randomized controlled clinical trials with longer follow-up periods and larger sample sizes are needed especially to determine an optimal duration and intensity of the treatment.

C1 [Afsar, Sevgi Ikbali; Yemisci, Oya Umit; Saracgil, Sacide Nur Cosar] Baskent Univ, Fac Med, Phys Med & Rehabil Dept, Ankara, Turkey.

[Mirzayev, Ilkin] Ilke Hosp, Phys Med & Rehabil, Yildirimbeyzait Cad 56, Istanbul, Turkey.

RP Afsar, SI (通讯作者)，Baskent Univ, Fac Med, Phys Med & Rehabil Dept Ankara, M Fevzi Cakmak Cad 5 Sok 48, TR-06490 Ankara, Turkey.

EM ikbaliafsar@hotmail.com

RI Afsar, Sevgi Ikbali/E-2103-2019; Yemisci, Oya Umit/AAJ-8820-2021; Cosar,

Sacide Nur Saracgil/AAF-1085-2021

OI Afsar, Sevgi Ikbali/0000-0002-4003-3646; Yemisci, Oya

Umit/0000-0002-0501-5127; Cosar, Sacide Nur Saracgil/0000-0001-8306-463X

CR Ahmed S, 2003, PHYS THER, V83, P617, DOI 10.1093/ptj/83.7.617

Arya KN, 2011, TOP STROKE REHABIL, V18, P599, DOI 10.1310/tsr18s01-599

Bao X, 2013, NEURAL REGEN RES, V8, P2904, DOI 10.3969/j.issn.1673-5374.2013.31.003

Bower KJ, 2015, J NEUROENG REHABIL, V12, DOI 10.1186/s12984-015-0057-x

Carey JR, 2005, EXERC SPORT SCI REV, V33, P24

DESROSIERS J, 1994, ARCH PHYS MED REHAB, V75, P751

FOLSTEIN MF, 1975, J PSYCHIAT RES, V12, P189, DOI 10.1016/0022-3956(75)90026-6

FUGLMEYER AR, 1975, SCAND J REHABIL MED, V7, P13

Holden MK, 2005, CYBERPSYCHOL BEHAV, V8, P187, DOI 10.1089/cpb.2005.8.187

Kucukdeveci AA, 2001, CLIN REHABIL, V15, P311, DOI 10.1191/026921501676877265

Lange B, 2011, IEEE ENG MED BIO, P1831, DOI 10.1109/IEMBS.2011.6090521

Laver KE, 2011, COCHRANE DB SYST REV, DOI [10.1002/14651858.CD008349.pub2, 10.1002/14651858.CD008349.pub4, 10.1002/14651858.CD008349.pub3]

Lee G, 2013, J PHYS THER SCI, V25, P595, DOI 10.1589/jpts.25.595

MATHIOWETZ V, 1985, AM J OCCUP THER, V39, P386, DOI 10.5014/ajot.39.6.386

Matijevic V, 2013, ACTA CLIN CROAT, V52, P453

Murray CJL, 2012, LANCET, V380, P2197, DOI 10.1016/S0140-6736(12)61689-4

Ottenbacher KJ, 1996, ARCH PHYS MED REHAB, V77, P1226, DOI 10.1016/S0003-9993(96)90184-7

Page SJ, 2012, ARCH PHYS MED REHAB, V93, P2373, DOI 10.1016/j.apmr.2012.06.017

Pascual-Leone A, 2005, ANNU REV NEUROSCI, V28, P377, DOI 10.1146/annurev.neuro.27.070203.144216

SANFORD J, 1993, PHYS THER, V73, P447, DOI 10.1093/ptj/73.7.447

Sawner KA, 1992, NEUROPHYSIOLOGICAL A, P41

Sin H, 2013, AM J PHYS MED REHAB, V92, P871, DOI 10.1097/PHM.0b013e3182a38e40

Song GB, 2015, J PHYS THER SCI, V27, P2057, DOI 10.1589/jpts.27.2057

Takeuchi N, 2012, NEURAL PLAST, V2012, DOI 10.1155/2012/359728

NR 24

TC 37

Z9 38

U1 7

U2 40

PU ELSEVIER SCIENCE BV

PI AMSTERDAM

PA PO BOX 211, 1000 AE AMSTERDAM, NETHERLANDS

SN 1052-3057

EI 1532-8511

J9 J STROKE CEREBROVASC

JI J. Stroke Cerebrovasc. Dis.

PD DEC

PY 2018

VL 27

IS 12

BP 3473

EP 3478

DI 10.1016/j.jstrokecerebrovasdis.2018.08.007

PG 6

WC Neurosciences; Peripheral Vascular Disease

WE Science Citation Index Expanded (SCI-EXPANDED)

SC Neurosciences & Neurology; Cardiovascular System & Cardiology

GA HA8VA

UT WOS:000450571100012

PM 30193810

DA 2022-06-21

ER

PT J

AU Afzal, MR

Byun, HY

Oh, MK

Yoon, J

AF Afzal, Muhammad Raheel

Byun, Ha-Young

Oh, Min-Kyun

Yoon, Jungwon

TI Effects of kinesthetic haptic feedback on standing stability of young

healthy subjects and stroke patients

SO JOURNAL OF NEUROENGINEERING AND REHABILITATION

LA English

DT Article

DE Light touch; Kinesthetic haptic feedback; Stroke patients; Smartphone;

Postural stability; Balance training

ID LIGHT TOUCH; VISUAL FEEDBACK; POSTURAL STABILIZATION; BALANCE; GAIT;

REHABILITATION; CONTACT; STANCE; SWAY

AB Background: Haptic control is a useful therapeutic option in rehabilitation featuring virtual reality interaction. As with visual and vibrotactile biofeedback, kinesthetic haptic feedback may assist in postural control, and can achieve balance control. Kinesthetic haptic feedback in terms of body sway can be delivered via a commercially available haptic device and can enhance the balance stability of both young healthy subjects and stroke patients.

Method: Our system features a waist-attached smartphone, software running on a computer (PC), and a dedicated Phantom Omni (R) device. Young healthy participants performed balance tasks after assumption of each of four distinct postures for 30 s (one foot on the ground; the Tandem Romberg stance; one foot on foam; and the Tandem Romberg stance on foam) with eyes closed. Patient eyes were not closed and assumption of the Romberg stance (only) was tested during a balance task 25 s in duration. An Android application running continuously on the smartphone sent mediolateral (ML) and anteroposterior (AP) tilt angles to a PC, which generated kinesthetic haptic feedback via Phantom Omni (R). A total of 16 subjects, 8 of whom were young healthy and 8 of whom had suffered stroke, participated in the study.

Results: Post-experiment data analysis was performed using MATLAB (R). Mean Velocity Displacement (MVD), Planar Deviation (PD), Mediolateral Trajectory (MLT) and Anteroposterior Trajectory (APT) parameters were analyzed to measure reduction in body sway. Our kinesthetic haptic feedback system was effective to reduce postural sway in young healthy subjects regardless of posture and the condition of the substrate (the ground) and to improve MVD and PD in stroke patients who assumed the Romberg stance. Analysis of Variance (ANOVA) revealed that kinesthetic haptic feedback significantly reduced body sway in both categories of subjects.

Conclusion: Kinesthetic haptic feedback can be implemented using a commercial haptic device and a smartphone. Intuitive balance cues were created using the handle of a haptic device, rendering the approach very simple yet efficient in practice. This novel form of biofeedback will be a useful rehabilitation tool improving the balance of stroke patients.

C1 [Afzal, Muhammad Raheel; Yoon, Jungwon] Gyeongsang Natl Univ, Sch Mech & Aerosp Engn, Jinju, South Korea.

[Afzal, Muhammad Raheel; Yoon, Jungwon] Gyeongsang Natl Univ, ReCAPT, Jinan, South Korea.

[Byun, Ha-Young; Oh, Min-Kyun] Gyeongsang Natl Univ Hosp, Dept Rehabil Med, Jinju, South Korea.

RP Yoon, J (通讯作者)，Gyeongsang Natl Univ, Sch Mech & Aerosp Engn, Jinju, South Korea.

EM jwyoon@gnu.ac.kr

RI Afzal, Muhammad Raheel/H-3485-2019

OI Afzal, Muhammad Raheel/0000-0003-3572-5709; Yoon,

Jungwon/0000-0003-1350-5334

FU National Research Foundation Korea (NRF) [2012R1A2A2A01047344,

2014R1A2A1A11053989, 2012-0009524]; technology development project for

advancement of NIPA SW convergence technology & Dual Use Technology

Program of Civil and Military

FX This work was supported by National Research Foundation Korea (NRF)

(2012R1A2A2A01047344 &2014R1A2A1A11053989 & 2012-0009524) and supported

by technology development project for advancement of NIPA SW convergence

technology & Dual Use Technology Program of Civil and Military. Min-Kyun

Oh (solioh21@hanmail.net) contributed equally to this work as a

corresponding author.

CR Albertsen IM, 2010, HUM MOVEMENT SCI, V29, P999, DOI 10.1016/j.humov.2010.07.013

Allum JHJ, 1998, GAIT POSTURE, V8, P214, DOI 10.1016/S0966-6362(98)00027-7

Bayouk JF, 2006, INT J REHABIL RES, V29, P51, DOI 10.1097/01.mrr.0000192100.67425.84

Boonsinsukh R, 2009, ARCH PHYS MED REHAB, V90, P919, DOI 10.1016/j.apmr.2008.12.022

BRANDSTATER ME, 1983, ARCH PHYS MED REHAB, V64, P583

Brown R, 2009, POLYM TEST, V28, P1, DOI 10.1016/j.polymertesting.2008.12.001

Brunnstrom S, 1966, Phys Ther, V46, P357

Cheng PT, 2004, CLIN REHABIL, V18, P747, DOI 10.1191/0269215504cr778oa

Dault MC, 2003, HUM MOVEMENT SCI, V22, P221, DOI 10.1016/S0167-9457(03)00034-4

Ersal T, 2013, J NEUROENG REHABIL, V10, DOI [10.1186/1743-0003-10-14, 10.1186/1743-0003-10-93]

Franco C, 2013, IEEE T BIO-MED ENG, V60, P211, DOI 10.1109/TBME.2012.2222640

Fung J, 2003, SOC NEUROSCI, V29, P12

Fung J, 2011, IEEE ENG MED BIO, P6753, DOI 10.1109/IEMBS.2011.6091666

Geomagic Industrial Products, 2015, PHANT OMN

Gurfinkel V.S., 1991, BRAIN SPACE

Hassan BS, ANN RHEUM DIS

HESSE S, 1995, STROKE, V26, P976, DOI 10.1161/01.STR.26.6.976

HESSE S, 1994, ARCH PHYS MED REHAB, V75, P1087, DOI 10.1016/0003-9993(94)90083-3

Holden M, 1994, J Vestib Res, V4, P285

Husemann B, STROKE

JEKA JJ, 1994, EXP BRAIN RES, V100, P495, DOI 10.1007/BF02738408

JEKA JJ, 1995, EXP BRAIN RES, V103, P267

Jeka JJ, 1997, PHYS THER, V77, P476, DOI 10.1093/ptj/77.5.476

Juen J., 2013, P INT C BIOINF COMP, P897, DOI DOI 10.1145/2506583.2512362

Kouzaki M, 2008, EXP BRAIN RES, V188, P153, DOI 10.1007/s00221-008-1426-5

Kwakkel G, 1996, AGE AGEING, V25, P479, DOI 10.1093/ageing/25.6.479

Lamb SE, 2003, STROKE, V34, P494, DOI 10.1161/01.STR.0000053444.00582.B7

Lee BC, 2012, J NEUROENG REHABIL, V9, DOI 10.1186/1743-0003-9-10

Lemoyne R, 2008, J MECH MED BIOL, V8, P137, DOI 10.1142/S0219519408002656

LOEWEN SC, 1990, STROKE, V21, P78, DOI 10.1161/01.STR.21.1.78

Lofgren B, 1998, DISABIL REHABIL, V20, P55

LORISH TR, 1994, ARCH PHYS MED REHAB, V75, pS47

Maatar Dhouha, 2013, International Journal of Image, Graphics and Signal Processing, V5, P33, DOI 10.5815/ijigsp.2013.06.05

Maatar D, 2012, INT J IMAGE GRAPH SI, V4, P21, DOI [10.5815/ijigsp.2012.05.03, DOI 10.5815/IJIGSP.2012.05.03]

Maatar D., 2011, J BIOMED SCI ENG, V4, P543, DOI DOI 10.4236/JBISE.2011.48070

Marigold DS, 2004, NEUROREHAB NEURAL RE, V18, P222, DOI 10.1177/1545968304271171

Mataar D, 2013, INT J COMPUT TECHNOL, V7, P580

Matjacic Z, 2003, NEUROREHABILITATION, V18, P251

Mayr A, 2007, NEUROREHAB NEURAL RE, V21, P307, DOI 10.1177/1545968307300697

Patton J, 2008, TOP STROKE REHABIL, V15, P131, DOI 10.1310/tsr1502-131

Petersen H, 1996, SCAND J REHABIL MED, V28, P217

Sackley C M, 1991, Int Disabil Stud, V13, P1

Sackley CM, 1997, DISABIL REHABIL, V19, P536, DOI 10.3109/09638289709166047

Salisbury CM, 2011, IEEE T HAPTICS, V4, P134, DOI [10.1109/TOH.2011.5, 10.1109/ToH.2011.5]

Srivastava A, 2009, J NEUROL SCI, V287, P89, DOI 10.1016/j.jns.2009.08.051

Tremblay F, 2004, EXP BRAIN RES, V157, P275, DOI 10.1007/s00221-004-1830-4

Wing AM, 2011, PHILOS T R SOC B, V366, P3133, DOI 10.1098/rstb.2011.0169

NR 47

TC 32

Z9 33

U1 0

U2 40

PU BMC

PI LONDON

PA CAMPUS, 4 CRINAN ST, LONDON N1 9XW, ENGLAND

SN 1743-0003

J9 J NEUROENG REHABIL

JI J. NeuroEng. Rehabil.

PD MAR 13

PY 2015

VL 12

AR 27

DI 10.1186/s12984-015-0020-x

PG 11

WC Engineering, Biomedical; Neurosciences; Rehabilitation

WE Science Citation Index Expanded (SCI-EXPANDED)

SC Engineering; Neurosciences & Neurology; Rehabilitation

GA CD8ME

UT WOS:000351348300001

PM 25889581

OA gold, Green Published

DA 2022-06-21

ER

PT J

AU Aguilera-Rubio, A

Alguacil-Diego, IM

Mallo-Lopez, A

Cuesta-Gomez, A

AF Aguilera-Rubio, Angela

Alguacil-Diego, Isabel M.

Mallo-Lopez, Ana

Cuesta-Gomez, Alicia

TI Use of the Leap Motion Controller (R) System in the Rehabilitation of

the Upper Limb in Stroke. A Systematic Review

SO JOURNAL OF STROKE & CEREBROVASCULAR DISEASES

LA English

DT Review

DE Leap Motion Controller (R); Neurorehabilitation; Serious games; Stroke;

Upper limb; Virtual reality

ID VIRTUAL-REALITY; SUBACUTE STROKE; RECOVERY; FEASIBILITY

AB Objectives: Upper limb impairment is the most common motor impairment in stroke survivors. The use of new technologies in the field of rehabilitation aims to reduce the impact of functional problems. Our objective is to evaluate the effectiveness of using the Leap Motion Controller (R) virtual reality system in the treatment of upper limb functionality in people with stroke. Materials and Methods: PRISMA guidelines were used to carry out the systematic review. The literature search was restricted to articles written in English or Spanish published from 2012 to December 2020 in Pubmed, Web of Science, Scopus, PEDro and Science Direct. Of the 309 search results, 230 unique references were reviewed after duplicates were removed. The Downs and Black and CONSORT scales were applied to evaluate the methodological quality of the included papers and the degree of evidence and level of recommendation were determined through the Oxford Centre for Evidence-Based Medicine. Results: Six papers with a total of 144 participants were included in this review, with heterogeneity of the sample, assessment measures, protocols, number of sessions and diversity of games applied. The main results of the studies show favourable data after using the Leap Motion Controller (R) system in the improvement of upper limb functionality in people with stroke. Conclusions: There is a growing trend in the use of the Leap Motion Controller (R) device as a tool in the treatment of the upper limb in people with stroke. Nevertheless, the limitations encountered suggest the need for future research protocols with greater scientific rigor.

C1 [Aguilera-Rubio, Angela; Mallo-Lopez, Ana] Rey Juan Carlos Univ, Int PhD School, Madrid, Spain.

[Aguilera-Rubio, Angela; Mallo-Lopez, Ana] NeuroAvanza Neurol Physiotherapy Ctr, Madrid, Spain.

[Alguacil-Diego, Isabel M.; Cuesta-Gomez, Alicia] Rey Juan Carlos Univ, Fac Hlth Sci, Dept Phys Therapy Occupat Therapy Rehabil & Phys, Madrid, Spain.

RP Alguacil-Diego, IM (通讯作者)，Rey Juan Carlos Univ, Fac Hlth Sci, Ave Atenas S-N, Madrid 28922, Spain.

EM isabel.alguacil@urjc.es

RI Gomez, Alicia Cuesta/P-5083-2016

OI Gomez, Alicia Cuesta/0000-0001-9507-2717; Aguilera,

Angela/0000-0002-6394-7037; Mallo, Ana/0000-0003-2208-6768

CR Alvarez-Sabin J, 2016, NEUROLOGIA, V13, P3

Bayon M, 2014, REHABILITACION, V48, P232

Bernhardt J, 2017, INT J STROKE, V12, P444, DOI 10.1177/1747493017711816

Buma F, 2013, RESTOR NEUROL NEUROS, V31, P707, DOI 10.3233/RNN-130332

Centre for Evidence-Based Medicine, 2009, OXFORD CTR EVIDENCE

Colombo R, 2019, IEEE T NEUR SYS REH, V27, P664, DOI 10.1109/TNSRE.2019.2905076

Cramer SC, 1997, STROKE, V28, P2518, DOI 10.1161/01.STR.28.12.2518

Dabholkar A., 2015, INT J HLTH REHABIL S, V4, P95, DOI [10.5455/ijhrs.000000079, DOI 10.5455/IJHRS.000000079]

Downs SH, 1998, J EPIDEMIOL COMMUN H, V52, P377, DOI 10.1136/jech.52.6.377

Eldridge SM, 2016, BMJ-BRIT MED J, V355, DOI 10.1136/bmj.i5239

Fluet GG, 2019, GAMES HEALTH J, V8, P432, DOI 10.1089/g4h.2019.0012

Hatem SM, 2016, FRONT HUM NEUROSCI, V10, DOI 10.3389/fnhum.2016.00442

Hutton B, 2016, MED CLIN-BARCELONA, V147, P262, DOI 10.1016/j.medcli.2016.02.025

Iosa M, 2015, TOP STROKE REHABIL, V22, P306, DOI 10.1179/1074935714Z.0000000036

Laver KE, 2015, COCHRANE DB SYST REV, DOI 10.1002/14651858.CD008349.pub3

Massetti T, 2018, J CENT NERV SYST DIS, V10, DOI 10.1177/1179573518813541

Murphy TH, 2009, NAT REV NEUROSCI, V10, P861, DOI 10.1038/nrn2735

Ogun MN, 2019, ARQ NEURO-PSIQUIAT, V77, P681, DOI [10.1590/0004-282X20190129, 10.1590/0004-282x20190129]

Organizacion ~ Mundial de la Salud, 2007, TRASTORNOS NEUROLOGI

Peters DM, 2013, J NEUROL PHYS THER, V37, P105, DOI 10.1097/NPT.0b013e31829ee9bc

Reza H, 2018, PROCEDIA CIRP, V78, P127

Rodriguez R, 2012, METODOLOGIAS MODELAD

Smeragliuolo AH, 2016, J BIOMECH, V49, P1742, DOI 10.1016/j.jbiomech.2016.04.006

Stevens E, IMPACTO ICTUS EUROPA

Vanbellingen T, 2017, FRONT NEUROL, V8, DOI 10.3389/fneur.2017.00654

Vinas-Diz S, 2016, NEUROLOGIA, V31, P255, DOI 10.1016/j.nrl.2015.06.012

Wang ZR, 2017, NEURAL REGEN RES, V12, P1823, DOI 10.4103/1673-5374.219043

Webster A, 2019, ADV EXP MED BIOL, V1120, P83, DOI 10.1007/978-3-030-06070-1_7

Wei YH, 2019, WORLD J CLIN CASES, V7, P3964, DOI 10.12998/wjcc.v7.i23.3964

WHO, 2014, GLOBAL STATUS REPORT ON VIOLENCE PREVENTION 2014, P1

NR 30

TC 2

Z9 2

U1 2

U2 6

PU ELSEVIER

PI AMSTERDAM

PA RADARWEG 29, 1043 NX AMSTERDAM, NETHERLANDS

SN 1052-3057

EI 1532-8511

J9 J STROKE CEREBROVASC

JI J. Stroke Cerebrovasc. Dis.

PD JAN

PY 2022

VL 31

IS 1

AR 106174

DI 10.1016/j.jstrokecerebrovasdis.2021.106174

EA NOV 2021

PG 14

WC Neurosciences; Peripheral Vascular Disease

WE Science Citation Index Expanded (SCI-EXPANDED)

SC Neurosciences & Neurology; Cardiovascular System & Cardiology

GA XC5BV

UT WOS:000722029100005

PM 34800859

OA hybrid

DA 2022-06-21

ER

PT J

AU Ahmad, M

Narayanasamy, S

Siddiqui, MA

Ahmed, I

AF Ahmad, M.

Narayanasamy, S.

Siddiqui, M. A.

Ahmed, I.

TI GIANT PERIVASCULAR SPACES: UTILITY OF MR IN DIFFERENTIATION FROM OTHER

CYSTIC LESIONS OF THE BRAIN

SO JBR-BTR

LA English

DT Article

DE Brain; cysts - Brain; MR

ID VIRCHOW-ROBIN SPACES; APPEARANCE

AB Perivascular or Virchow-Robin spaces (VR) of the brain are fluid-filled, pial-lined spaces that accompany the cerebral vessels as they pass from subarachnoid space into the brain parenchyma. They are visualized on routine MR examinations as CSF intensity spaces and are normally < 2 mm in size. These spaces may rarely enlarge massively and can be mistaken for more ominous pathologic processes on CT scan and even on MRI, but careful examination on special sequences and follow-up examination can clear the uncertainty. We describe a case of a young male who presented with mildly progressive neurological symptoms and the imaging findings were typical of enlarged VR spaces. Patient was advised follow up and is doing well.

C1 [Ahmad, M.; Narayanasamy, S.; Siddiqui, M. A.; Ahmed, I.] Jawaharlal Nehru Med Coll, Dept Radiodiagnosis, Amu Aligarh, Uttar Pradesh, India.

RP Narayanasamy, S (通讯作者)，Jawaharlal Nehru Med Coll, Dept Radiodiagnosis, Aligarh 202002, Uttar Pradesh, India.

EM nsabarish86@gmail.com

CR Achiron A, 2002, AM J NEURORADIOL, V23, P376

ESIRI MM, 1990, J NEUROL SCI, V100, P3, DOI 10.1016/0022-510X(90)90004-7

HEIER LA, 1989, AM J NEURORADIOL, V10, P929

House P, 2004, J NEUROSURG, V100, P820, DOI 10.3171/jns.2004.100.5.0820

Komiyama M, 1998, NEUROL MED-CHIR, V38, P161, DOI 10.2176/nmc.38.161

Kwee RM, 2007, RADIOGRAPHICS, V27, P1071, DOI 10.1148/rg.274065722

OGAWA T, 1995, AM J NEURORADIOL, V16, P1238

Papayannis CE, 2003, AM J NEURORADIOL, V24, P1399

Salzman KL, 2005, AM J NEURORADIOL, V26, P298

NR 9

TC 2

Z9 3

U1 0

U2 1

PU ASSOC ROYAL SOC SCIENTIFIQUES MEDICALES BELGES

PI BRUSSELS

PA AVE W CHURCHILL 11-30, BRUSSELS, B-1180, BELGIUM

SN 1780-2393

J9 JBR-BTR

JI JBR-BTR

PD NOV-DEC

PY 2014

VL 97

IS 6

BP 364

EP 365

DI 10.5334/jbr-btr.123

PG 2

WC Radiology, Nuclear Medicine & Medical Imaging

WE Science Citation Index Expanded (SCI-EXPANDED)

SC Radiology, Nuclear Medicine & Medical Imaging

GA CD6AZ

UT WOS:000351172100008

PM 25786296

OA Green Submitted, gold, Green Published

DA 2022-06-21

ER

PT J

AU Aida, J

Chau, B

Dunn, J

AF Aida, Jared

Chau, Brian

Dunn, Justin

TI Immersive virtual reality in traumatic brain injury rehabilitation: A

literature review

SO NEUROREHABILITATION

LA English

DT Review

DE Virtual reality; traumatic brain injury; therapy; rehabilitation

ID PERFORMANCE; ENVIRONMENT; EXPOSURE

AB BACKGROUND: Traumatic brain injury (TBI) is a common cause of morbidity and mortality in the United States with its sequelae often affecting individuals long after the initial injury. Innovations in virtual reality (VR) technology may offer potential therapy options in the recovery from such injuries. However, there is currently no consensus regarding the efficacy of VR in the setting of TBI rehabilitation.

OBJECTIVE: The aim of this review is to evaluate and summarize the current literature regarding immersive VR in the rehabilitation of those with TBI.

METHODS: A comprehensive literature search was conducted utilizing PubMed, Google Scholar, and the Cochrane Review using the search terms "virtual reality," "traumatic brain injury," "brain injury," and " immersive. "

RESULTS: A total of 11 studies were evaluated. These were primarily of low-level evidence, with the exception of two randomized, controlled trials. 10 of 11 studies demonstrated improvement with VR therapy. VR was most frequently used to address gait or cognitive deficits.

CONCLUSIONS: While the current literature generally offers support for the use of VR in TBI recovery, there is a paucity of strong evidence to support its widespread use. The increasing availability of immersive VR technology offers the potential for engaging therapy in TBI rehabilitation, but its utility remains uncertain given the limited studies available at this time.

C1 [Aida, Jared] Loma Linda Univ Hlth, Dept Phys Med & Rehabil, 11406 Loma Linda Dr,Suite 516, Loma Linda, CA 92354 USA.

[Chau, Brian] Loma Linda Healthcare Syst, Dept Vet Affairs, Dept Phys Med & Rehabil, Redlands, CA USA.

[Dunn, Justin] Loma Linda Univ, Sch Med, Loma Linda, CA USA.

RP Aida, J (通讯作者)，Loma Linda Univ Hlth, Dept Phys Med & Rehabil, 11406 Loma Linda Dr,Suite 516, Loma Linda, CA 92354 USA.

EM jaida@llu.edu

FU Loma Linda University Health Department of Physical Medicine and

Rehabilitation

FX We would like to thank the Loma Linda University Health Department of

Physical Medicine and Rehabilitation for their support.

CR [Anonymous], 2015, C TRAUM BRAIN INJ US

Biffi E, 2015, IEEE ENG MED BIO, P7406, DOI 10.1109/EMBC.2015.7320103

Biffi E, 2017, METHOD INFORM MED, V56, P119, DOI 10.3414/ME16-02-0020

Bouchard S, 2017, BRIT J PSYCHIAT, V210, P276, DOI 10.1192/bjp.bp.116.184234

Christiansen C, 1998, ARCH PHYS MED REHAB, V79, P888, DOI 10.1016/S0003-9993(98)90083-1

Cox DJ, 2010, MIL MED, V175, P411, DOI 10.7205/MILMED-D-09-00081

Darter BJ, 2011, PHYS THER, V91, P1385, DOI 10.2522/ptj.20100360

Dunn J, 2017, NEUROREHABILITATION, V40, P595, DOI 10.3233/NRE-171447

Dvorkin AY, 2013, J NEUROENG REHABIL, V10, DOI 10.1186/1743-0003-10-92

Ferrer-Garcia M, 2013, J CONTEMP PSYCHOTHER, V43, P207, DOI 10.1007/s10879-013-9240-1

Gamito P., 2011, INT J DISABIL HUM DE, V10, P309, DOI [10.1515/IJDHD.2011.049, DOI 10.1515/IJDHD.2011.049]

Gottshall KR, 2015, FRONT SYST NEUROSCI, V9, DOI 10.3389/fnsys.2015.00106

Hashimoto D. A., 2017, SURG ENDOSCOPY

Highland KB, 2015, ANN REV CYBERTHERAPY, V13, P23

Kaufman KR, 2014, CLIN ORTHOP RELAT R, V472, P3076, DOI 10.1007/s11999-014-3664-0

Lo Priore C., CYBERPSYCHOLOGY BEHA, V6, P281

Mckee AC, 2009, J NEUROPATH EXP NEUR, V68, P709, DOI 10.1097/NEN.0b013e3181a9d503

Morina N, 2015, BEHAV RES THER, V74, P18, DOI 10.1016/j.brat.2015.08.010

Ortiz-Catalan M, 2014, FRONT NEUROSCI-SWITZ, V8, DOI 10.3389/fnins.2014.00024

Parsons TD, 2009, DEV NEUROREHABIL, V12, P224, DOI 10.1080/17518420902991719

Rabago CA, 2011, J NEUROL PHYS THER, V35, P185, DOI 10.1097/NPT.0b013e318235d7e6

Schultheis MT, 2001, REHABIL PSYCHOL, V46, P296, DOI 10.1037/0090-5550.46.3.296

Sessoms PH, 2015, MIL MED, V180, P143, DOI 10.7205/MILMED-D-14-00385

Winstein CJ, 2016, STROKE, V47, pE98, DOI 10.1161/STR.0000000000000098

NR 24

TC 32

Z9 36

U1 4

U2 28

PU IOS PRESS

PI AMSTERDAM

PA NIEUWE HEMWEG 6B, 1013 BG AMSTERDAM, NETHERLANDS

SN 1053-8135

EI 1878-6448

J9 NEUROREHABILITATION

JI Neurorehabilitation

PY 2018

VL 42

IS 4

BP 441

EP 448

DI 10.3233/NRE-172361

PG 8

WC Clinical Neurology; Rehabilitation

WE Science Citation Index Expanded (SCI-EXPANDED); Social Science Citation Index (SSCI)

SC Neurosciences & Neurology; Rehabilitation

GA GL1RG

UT WOS:000436883100006

PM 29660958

DA 2022-06-21

ER

PT J

AU Ul Ain, Q

Khan, S

Ilyas, S

Yaseen, A

Tariq, I

Liu, T

Wang, J

AF Ain, Qurat Ul

Khan, Sara

Ilyas, Saad

Yaseen, Amna

Tariq, Iqbal

Liu, Tian

Wang, Jue

TI Additional Effects of Xbox Kinect Training on Upper Limb Function in

Chronic Stroke Patients: A Randomized Control Trial

SO HEALTHCARE

LA English

DT Article

DE Box and Block Test; Fugl-Meyer Assessment Scale for Upper Extremity;

motor function; rehabilitation; stroke; upper extremity; virtual

reality; Xbox 360

ID UPPER EXTREMITY FUNCTION; FUGL-MEYER ASSESSMENT; VIRTUAL-REALITY; MOTOR

RECOVERY; DOSE-RESPONSE; REHABILITATION; BLIND; POSTSTROKE; SURVIVORS;

THERAPY

AB Background: Xbox Kinect-based virtual reality, being a novel approach, has therapeutic benefits in rehabilitation and its use is encouraged in stroke rehabilitation of upper extremities. Objective: Primary aim of the current study is to investigate the additional effects of Xbox Kinect training in combination with routine physiotherapy exercises based on each component of Fugl-Meyer Assessment Scale for Upper Extremity (FMA-UE). Moreover, effect of upper limb rehabilitation on cognitive functions was also assessed. Methods: This study was a parallel arm randomized control trial. Fifty-six participants were recruited and randomly allocated to either an Xbox Kinect training group (XKGT) or exercise training group (ETG). Measures of concern were recorded using FMA-UE, Box and Block Test (BBT), and Montreal Cognitive Assessment (MOCA). Evaluation was conducted at baseline and after completion of intervention at the sixth week. Results: There were significant differences from pre- to post-intervention scores of FMA-UE and BBT (p < 0.001) in both groups, whereas no difference was observed for MOCA (XKTG p value 0.417, ETG p value 0.113). At six-week follow-up there were significant differences between both groups in FMA-UE total score (p < 0.001), volitional movement within synergies (p < 0.001), wrist (p = 0.021), hand (p = 0.047), grasp (p = 0.006) and coordination/speed (p = 0.004), favoring the Xbox Kinect training group. Conclusion: To conclude, results indicate repetitive use of the hemiparetic upper extremity by Xbox Kinect-based upper limb rehabilitation training in addition to conventional therapy has a promising potential to enhance upper limb motor function for stroke patients.

C1 [Ain, Qurat Ul; Liu, Tian; Wang, Jue] Xi An Jiao Tong Univ, Sch Life Sci & Technol, Key Lab Biomed Informat Engn, Minist Educ,Inst Hlth & Rehabil Sci, Xian 710049, Peoples R China.

[Ain, Qurat Ul; Liu, Tian; Wang, Jue] Natl Engn Res Ctr Healthcare Devices, Guangzhou 510500, Peoples R China.

[Ain, Qurat Ul; Liu, Tian; Wang, Jue] Minist Civil Affairs, Key Lab Neuroinformat & Rehabil Engn, Xian 710049, Peoples R China.

[Khan, Sara] Mukkabir Coll, Physiotherapy Dept, Gujrat 50700, Pakistan.

[Ilyas, Saad] Univ Cent Punjab, Fac Informat Technol, Dept Comp Sci, Lahore 54000, Pakistan.

[Yaseen, Amna; Tariq, Iqbal] Riphah Int Univ, Fac Rehabil & Allied Hlth Sci, Riphah Coll Rehabil & Allied Hlth Sci, Islamabad 46000, Pakistan.

RP Liu, T; Wang, J (通讯作者)，Xi An Jiao Tong Univ, Sch Life Sci & Technol, Key Lab Biomed Informat Engn, Minist Educ,Inst Hlth & Rehabil Sci, Xian 710049, Peoples R China.; Liu, T; Wang, J (通讯作者)，Natl Engn Res Ctr Healthcare Devices, Guangzhou 510500, Peoples R China.; Liu, T; Wang, J (通讯作者)，Minist Civil Affairs, Key Lab Neuroinformat & Rehabil Engn, Xian 710049, Peoples R China.

EM qurat.iimc@gmail.com; sarah.warraich4@gmail.com; saadkhan1290@gmail.com;

amna.yaseen@riphah.edu.pk; iqbal1tariq@gmail.com; tianliu@xjtu.edu.cn;

juewang@mail.xjtu.edu.cn

OI Ain, Qurat Ul/0000-0001-6524-9796; Tariq, Muhammad

Iqbal/0000-0002-3049-9768

FU National Natural Science Foundation of China [U1913216, 31972907]

FX This research was funded by National Natural Science Foundation of

China: U1913216, 31972907.

CR Afsar SI, 2018, J STROKE CEREBROVASC, V27, P3473, DOI 10.1016/j.jstrokecerebrovasdis.2018.08.007

Askin A, 2018, SOMATOSENS MOT RES, V35, P25, DOI 10.1080/08990220.2018.1444599

August K., 2006, IEEE ENG MED BIOL SO, P3692, DOI [10.1109/IEMBS.2006.260144, DOI 10.1109/IEMBS.2006.260144]

Bao X, 2013, NEURAL REGEN RES, V8, P2904, DOI 10.3969/j.issn.1673-5374.2013.31.003

Chang YJ, 2011, RES DEV DISABIL, V32, P2566, DOI 10.1016/j.ridd.2011.07.002

Dennis A, 2011, STROKE, V42, P1056, DOI 10.1161/STROKEAHA.110.597880

Dominguez-Tellez P, 2020, GAMES HEALTH J, V9, P1, DOI 10.1089/g4h.2019.0043

DUNCAN PW, 1983, PHYS THER, V63, P1606, DOI 10.1093/ptj/63.10.1606

Gladstone DJ, 2002, NEUROREHAB NEURAL RE, V16, P232, DOI 10.1177/154596802401105171

Johnson L, 2018, BMJ OPEN, V8, DOI 10.1136/bmjopen-2017-018388

Karamians R, 2020, ARCH PHYS MED REHAB, V101, P885, DOI 10.1016/j.apmr.2019.10.195

Keller J, 2020, J NEUROENG REHABIL, V17, DOI 10.1186/s12984-020-00754-7

Kiper P, 2018, ARCH PHYS MED REHAB, V99, P834, DOI 10.1016/j.apmr.2018.01.023

Klamroth-Marganska V, 2014, LANCET NEUROL, V13, P159, DOI 10.1016/S1474-4422(13)70305-3

Kwon JS, 2012, NEUROREHABILITATION, V31, P379, DOI 10.3233/NRE-2012-00807

Lang Catherine E, 2007, J Neurol Phys Ther, V31, P3

Lang CE, 2016, ANN NEUROL, V80, P342, DOI 10.1002/ana.24734

Lang CE, 2013, J HAND THER, V26, P104, DOI 10.1016/j.jht.2012.06.005

Laver KE, 2017, COCHRANE DB SYST REV, DOI 10.1002/14651858.CD008349.pub4

Lee G, 2013, J PHYS THER SCI, V25, P595, DOI 10.1589/jpts.25.595

Licher S, 2019, J NEUROL NEUROSUR PS, V90, P148, DOI 10.1136/jnnp-2018-318650

Lohse KR, 2014, STROKE, V45, P2053, DOI 10.1161/STROKEAHA.114.004695

Merians AS, 2006, NEUROREHAB NEURAL RE, V20, P252, DOI 10.1177/1545968306286914

Park DS, 2017, J STROKE CEREBROVASC, V26, P2313, DOI 10.1016/j.jstrokecerebrovasdis.2017.05.019

Park J, 2015, J PHYS THER SCI, V27, P1075, DOI 10.1589/jpts.27.1075

Pascual-Leone A, 2005, ANNU REV NEUROSCI, V28, P377, DOI 10.1146/annurev.neuro.27.070203.144216

Pollock A, 2014, COCHRANE DB SYST REV, DOI 10.1002/14651858.CD010820.pub2

Rand D, 2018, PLOS ONE, V13, DOI 10.1371/journal.pone.0195043

Saposnik G, 2011, STROKE, V42, P1380, DOI 10.1161/STROKEAHA.110.605451

Schuster-Amft C, 2018, PLOS ONE, V13, DOI 10.1371/journal.pone.0204455

Simonetti D, 2017, FRONT HUM NEUROSCI, V11, DOI 10.3389/fnhum.2017.00268

Sin H, 2013, AM J PHYS MED REHAB, V92, P871, DOI 10.1097/PHM.0b013e3182a38e40

Soe M.M., 2019, OPENEPI OPEN SOURCE

Teka WW, 2017, PLOS ONE, V12, DOI 10.1371/journal.pone.0179288

Ward NS, 2019, J NEUROL NEUROSUR PS, V90, P498, DOI 10.1136/jnnp-2018-319954

Winstein CJ, 2016, JAMA-J AM MED ASSOC, V315, P571, DOI 10.1001/jama.2016.0276

Wolf TJ, 2016, AM J OCCUP THER, V70, DOI 10.5014/ajot.2016.017293

Xavier-Rocha TB, 2020, ARQ NEURO-PSIQUIAT, V78, P361, DOI [10.1590/0004-282X20200012, 10.1590/0004-282x20200012]

Xu GQ, 2016, FRONT HUM NEUROSCI, V10, DOI 10.3389/fnhum.2016.00038

You SH, 2005, STROKE, V36, P1166, DOI 10.1161/01.STR.0000162715.43417.91

NR 40

TC 2

Z9 2

U1 12

U2 16

PU MDPI

PI BASEL

PA ST ALBAN-ANLAGE 66, CH-4052 BASEL, SWITZERLAND

EI 2227-9032

J9 HEALTHCARE-BASEL

JI Healthcare

PD MAR

PY 2021

VL 9

IS 3

AR 242

DI 10.3390/healthcare9030242

PG 12

WC Health Care Sciences & Services; Health Policy & Services

WE Science Citation Index Expanded (SCI-EXPANDED); Social Science Citation Index (SSCI)

SC Health Care Sciences & Services

GA RD8KM

UT WOS:000633719500001

PM 33668355

OA Green Published, gold

DA 2022-06-21

ER

PT J

AU Dos Santos, LRA

Carregosa, AA

Masruha, MR

Dos Santos, PA

Coelho, MLD

Ferraz, DD

Ribeiro, NMD

AF Aguiar Dos Santos, Luan Rafael

Carregosa, Adriani Andrade

Masruha, Marcelo Rodrigues

Dos Santos, Pietro Araujo

Da Silveira Coelho, Marilia Lira

Ferraz, Daniel Dominguez

Da Silva Ribeiro, Nildo Manoel

TI The Use of Nintendo Wii in the Rehabilitation of Poststroke Patients: A

Systematic Review

SO JOURNAL OF STROKE & CEREBROVASCULAR DISEASES

LA English

DT Review

DE Stroke; virtual reality exposure therapy; video games; rehabilitation

ID RANDOMIZED CLINICAL-TRIAL; VIRTUAL-REALITY; STROKE REHABILITATION; MOTOR

FUNCTION; BALANCE; FEASIBILITY; QUALITY; THERAPY; FIT

AB Background: To evaluate the effectiveness of the video game console Nintendo Wii (NW) in motor function, balance, and functional independence in the treatment of poststroke patients and to identify which games are commonly used in therapy. Methods: Randomized controlled trials were researched in MEDLINE, Cochrane Library, PEDro, CAPES Periodic, BIREME, and LILACS databases, covering publications up to March 31, 2014. The assessment of methodological quality was performed using the PEDro Scale as reference. Results: The 5 studies included for analysis showed that NW can provide an improvement of motor function of the individual, but the data are unclear when it comes to the balance and functional independence. Conclusions: It was concluded that there is little evidence to ensure the effectiveness and support the inclusion of the treatment with NW in patients with sequelae caused by a stroke; however, some of the studies analyzed suggest that NW can provide improvement in motor function.

C1 [Aguiar Dos Santos, Luan Rafael; Carregosa, Adriani Andrade; Dos Santos, Pietro Araujo; Ferraz, Daniel Dominguez; Da Silva Ribeiro, Nildo Manoel] Univ Fed Bahia, Inst Hlth Sci, UFBA, BR-41710020 Salvador, BA, Brazil.

[Masruha, Marcelo Rodrigues] Univ Fed Sao Paulo, Dept Neurol Neurosurg, UNIFESP, Sao Paulo, Brazil.

[Da Silveira Coelho, Marilia Lira] Univ Prebiteriana Mackenzie, Sao Paulo, Brazil.

RP Dos Santos, LRA (通讯作者)，Univ Fed Bahia, Inst Hlth Sci, UFBA, Rua Lavinia Magalhaes 139, BR-41710020 Salvador, BA, Brazil.

EM aguiar.luanrafael@hotmail.com

RI Aguiar dos Santos, Luan/H-6752-2016; Dominguez-Ferraz,

Daniel/AAC-3687-2020

OI Aguiar dos Santos, Luan/0000-0002-1477-7422; Dominguez-Ferraz,

Daniel/0000-0003-3049-0058

CR Barcala L, 2011, PHYS THER MOV, V24, P337, DOI DOI 10.1590/S0103-51502011000200015

Barcala L, 2013, J PHYS THER SCI, V25, P1027, DOI 10.1589/jpts.25.1027

Bateni H, 2012, PHYSIOTHERAPY, V98, P211, DOI 10.1016/j.physio.2011.02.004

Brol AM, 2009, FISIOTER MOV, V22, P497

Cho KH, 2012, TOHOKU J EXP MED, V228, P69, DOI 10.1620/tjem.228.69

Faralli A, 2013, NEURAL PLAST, V2013, DOI 10.1155/2013/854597

Fernandes Marina Bessi, 2012, Fisioter. mov., V25, P333

Gil-Gomez JA, 2011, J NEUROENG REHABIL, V8, DOI 10.1186/1743-0003-8-30

Joo LY, 2010, J REHABIL MED, V42, P437, DOI 10.2340/16501977-0528

Kim EK, 2012, J PHYS THER SCI, V24, P901, DOI 10.1589/jpts.24.901

Kwakkel G., 1999, PHYSIOTHERAPY, V85, P377, DOI DOI 10.1016/S0031-9406(05)67198-2

Lin KC, 2009, NEUROREHAB NEURAL RE, V23, P429, DOI 10.1177/1545968308331144

Lohse KR, 2014, PLOS ONE, V9, DOI 10.1371/journal.pone.0093318

Maher CG, 2003, PHYS THER, V83, P713, DOI 10.1093/ptj/83.8.713

Montagna JC, 2014, INT J CLIN EXP MED, V7, P1182

Mouawad MR, 2011, J REHABIL MED, V43, P527, DOI 10.2340/16501977-0816

Piassaroli CA, 2011, REV NEUROCIENC, V634, P1

Piron L, 2008, J TELEMED TELECARE, V14, P257, DOI 10.1258/jtt.2008.080304

Saposnik G, 2010, STROKE, V41, P1477, DOI 10.1161/STROKEAHA.110.584979

Shiwa Sílvia Regina, 2011, Fisioter. mov., V24, P523

Souza LB, 2011, ACTAFISIATR, V18, P217

Takeuchi N, 2013, STROKE RES TREAT, V2013, DOI 10.1155/2013/128641

The Cochrane Collaborations, COCHRANE HDB SYSTEMA

Turolla A, 2013, J NEUROENG REHABIL, V10, DOI 10.1186/1743-0003-10-85

Verhagen AP, 1998, J CLIN EPIDEMIOL, V51, P1235, DOI 10.1016/S0895-4356(98)00131-0

World Health Organisation, 2018, HLTH TOP STROK CER A

NR 26

TC 34

Z9 34

U1 1

U2 17

PU ELSEVIER SCIENCE BV

PI AMSTERDAM

PA PO BOX 211, 1000 AE AMSTERDAM, NETHERLANDS

SN 1052-3057

EI 1532-8511

J9 J STROKE CEREBROVASC

JI J. Stroke Cerebrovasc. Dis.

PD OCT

PY 2015

VL 24

IS 10

BP 2298

EP 2305

DI 10.1016/j.jstrokecerebrovasdis.2015.06.010

PG 8

WC Neurosciences; Peripheral Vascular Disease

WE Science Citation Index Expanded (SCI-EXPANDED)

SC Neurosciences & Neurology; Cardiovascular System & Cardiology

GA CS6IG

UT WOS:000362181400025

PM 26303792

DA 2022-06-21

ER

PT J

AU Akhutina, T

Foreman, N

Krichevets, A

Matikka, L

Narhi, V

Pylaeva, N

Vahakuopus, J

AF Akhutina, T

Foreman, N

Krichevets, A

Matikka, L

Narhi, V

Pylaeva, N

Vahakuopus, J

TI Improving spatial functioning in children with cerebral palsy using

computerized and traditional game tasks

SO DISABILITY AND REHABILITATION

LA English

DT Article

ID VIRTUAL-REALITY; MENTAL ROTATION; REHABILITATION; MAZE; ENVIRONMENTS;

PERFORMANCE; INFORMATION; NAVIGATION; KNOWLEDGE; HUMANS

AB Purpose : To examine the effectiveness of combining virtual environment (VE) instruction with additional desk-top tasks, based on the Luria-Vygotsky methodology, for spatial remediation in children having complex motor disabilities restricting movement.

Method : In Experiment 1, from among children attending for residential rehabilitation, an experimental subgroup had additional spatial training using a VE and corresponding desk-top models. All children were tested at the start and end of training, using four spatial tests. In Experiment 2, larger groups of children (pair-matched for initial performance) were given the same training as in Experiment 1, but experimentals received both VE-based training and supporting tasks designed to improve executive functions and verbal regulation of spatial functioning. Assessment involved a wider range of tests than in Experiment 1.

Results : In Experiment 1, both groups showed improvement at retest, but experimentals showed greater improvement. Children beginning with the lowest level of cognitive performance failed to benefit from the additional training. In Experiment 2 the experimental group made significantly greater improvement than controls, irrespective of initial performance level.

Conclusions : VE-based spatial training is effective for children with complex disabilities, particularly when combined with training that remediates cognitive weaknesses.

C1 Middlesex Univ, Enfield EN3 4SF, Middx, England.

Moscow MV Lomonosov State Univ, Fac Psychol, Moscow, Russia.

Finnish Assoc Mental Retardat, Helsinki, Finland.

Univ Jyvaskyla, Dept Psychol, Niilo Maki Inst, SF-40351 Jyvaskyla, Finland.

RP Foreman, N (通讯作者)，Middlesex Univ, Enfield Campus,Queensway, Enfield EN3 4SF, Middx, England.

RI Akhutina, Tatiana/I-4793-2012; Pylaeva, Natalia/U-9655-2018; Krichevets,

Anatoly N/I-8359-2012

OI Narhi, Vesa/0000-0002-2619-8364

CR Akhutina TV, 1997, J INTELL DISABIL RES, V41, P144, DOI 10.1111/j.1365-2788.1997.tb00691.x

AKHUTINA TV, 2001, 8 INT C IACE JYV FIN

AKHUTINA TV, 2002, 4 AR LUR C MOSC RUSS

AKHUTINA TV, 2000, SHKOLA ZDAROVYA MOSC, V2, P21

Albert W. S., 1999, Spatial Cognition and Computation, V1, P131, DOI 10.1023/A:1010096408311

[Anonymous], 1980, INSTRUMENTAL ENRICHM

Benton A.L., 1983, CONTRIBUTIONS NEUROP

Bertenthal BI, 1984, CONTINUITIES DISCONT, P175, DOI [DOI 10.1007/978-1-4613-2725-7_8, 10.1007/978-1-4613-2725-7.8]

BOER LC, 1991, ACTA PSYCHOL, V76, P1, DOI 10.1016/0001-6918(91)90050-A

Cromby J. J., 1996, P 1 EUR C DIS VIRT R, P103

Doise W., 1984, SOCIAL DEV INTELLECT

FINNEY NR, 2001, CHILD CEREBRAL PALSY

Foreman N, 2003, J EXP PSYCHOL-APPL, V9, P67, DOI 10.1037/1076-898X.9.2.67

Foreman N, 2000, BEHAV BRAIN RES, V112, P53, DOI 10.1016/S0166-4328(00)00159-5

Foreman N., 1989, EUROPEAN J SPECIAL N, V3, P171, DOI 10.1080/0885625890040302

Foreman N., 1997, HDB SPATIAL RES PARA, V1

Kass S., 1998, J APPL DEV PSYCHOL, V15, P13

Korkman M., 1998, NEPSY A DEV NEUROPSY

KRICHEVETS AN, 1995, DISABIL REHABIL, V17, P100, DOI 10.3109/09638289509166635

Larson P, 1999, Cyberpsychol Behav, V2, P113, DOI 10.1089/cpb.1999.2.113

LEVCHENKO II, 2001, TECHNOLOGY ED UP BRI

LIBERTI G, 1984, J VISUAL IMPAIRM MAR, P121

Light P., 1992, CONTEXT COGNITION WA

LIGHT P, 1991, CHILD DEV SOCIAL CON, V2

Luria, 1996, HIGHER CORTICAL FUNC

Maguire EA, 1998, SCIENCE, V280, P921, DOI 10.1126/science.280.5365.921

McComas J, 1998, CYBER PSYCHOL BEHAV, V7, P115, DOI DOI 10.1089/CPB.1998.1.121

Moffat SD, 1998, EVOL HUM BEHAV, V19, P73, DOI 10.1016/S1090-5138(97)00104-9

Okagaki L., 1994, J APPL DEV PSYCHOL, V15, P33, DOI [10.1016/0193-3973(94)90005-1, DOI 10.1016/0193-3973(94)90005-1, 10.1016/0193-3973(94)90005-1.]

Pennington B. F., 1993, DIAGNOSING LEARNING

Pylaeva N. M., 2000, SCH HLTH, V3, P26

PYLAEVA NM, 1997, SCH ATTENTION METHOD

Raven J, 1965, GUIDE USING COLOURED

*RES I PRESCH ED, 1960, ROADS TEST

Rose D, 1999, PSYCHOLOGIST, V12, P550

Rose FD, 1999, DISABIL REHABIL, V21, P548

ROURKE B, 1991, NEUROPSYCHOLOGICAL V

SAARELA M, 1995, PUBLICATIONS MENTAL, V66

SHEPARD RN, 1971, SCIENCE, V171, P701, DOI 10.1126/science.171.3972.701

Skelton RW, 2000, J CLIN EXP NEUROPSYC, V22, P157, DOI 10.1076/1380-3395(200004)22:2;1-1;FT157

Snodgrass L., 2000, 27 INT C PSYCH STOCK

Stanton D, 2002, BEHAV BRAIN RES, V136, P61, DOI 10.1016/S0166-4328(02)00097-9

Stanton D., 1996, 1 EUR C DIS VIRT REA, P93

Tkacz S, 1998, J ENVIRON PSYCHOL, V18, P237, DOI 10.1006/jevp.1998.0094

VANDENBERG SG, 1978, PERCEPT MOTOR SKILL, V47, P599, DOI 10.2466/pms.1978.47.2.599

Vygotsky L. S., 1981, CONCEPT ACTIVITY SOV, P144

VYGOTSKY LS, 1965, NEUROPSYCHOLOGIA, V3, P361

Waller D, 2000, J EXP PSYCHOL-APPL, V6, P307, DOI 10.1037//1076-898X.6.4.307

Wilson PN, 1998, DISABIL REHABIL, V20, P113, DOI 10.3109/09638289809166069

Wilson PN, 1996, DISABIL REHABIL, V18, P633, DOI 10.3109/09638289609166328

Wilson PN, 1997, DISABIL REHABIL, V19, P213, DOI 10.3109/09638289709166530

WILSON PN, 1997, HDB SPATIAL RES PARA, V1

Witmer BG, 1996, INT J HUM-COMPUT ST, V45, P413, DOI 10.1006/ijhc.1996.0060

NR 53

TC 48

Z9 50

U1 2

U2 17

PU TAYLOR & FRANCIS LTD

PI ABINGDON

PA 4 PARK SQUARE, MILTON PARK, ABINGDON OX14 4RN, OXON, ENGLAND

SN 1464-5165

J9 DISABIL REHABIL

JI Disabil. Rehabil.

PD DEC 16

PY 2003

VL 25

IS 24

BP 1361

EP 1371

DI 10.1080/09638280310001616358

PG 11

WC Rehabilitation

WE Science Citation Index Expanded (SCI-EXPANDED); Social Science Citation Index (SSCI)

SC Rehabilitation

GA 746NP

UT WOS:000186752700004

PM 14660204

DA 2022-06-21

ER

PT J

AU Al Jerdi, S

Aleyadeh, R

Imam, Y

AF Al Jerdi, Salman

Aleyadeh, Rozaleen

Imam, Yahia

TI Management of Cognitive Impairment After Stroke

SO CURRENT TREATMENT OPTIONS IN NEUROLOGY

LA English

DT Review

DE Post-stroke cognitive impairment; Vascular dementia; Stroke dementia;

Vascular cognitive impairment

ID SUBCORTICAL VASCULAR DEMENTIA; DIRECT-CURRENT STIMULATION; HEALTH-CARE

PROFESSIONALS; MULTI-INFARCT DEMENTIA; LOW-DOSE ASPIRIN;

PHYSICAL-ACTIVITY; WHITE-MATTER; DOUBLE-BLIND; CIBIC PLUS; EFFICACY

AB Purpose of Review In this review, we present the reader with the latest diagnostic and therapeutic approaches in the management of patients with post-stroke cognitive impairment (PSCI). We discuss the most commonly utilized and validated neuropsychological assessments and neuroimaging diagnostic assessment tools. We also provide a critical appraisal of the pharmacological treatment approaches of PSCI, in addition to highlighting the latest emerging non-pharmacological and technological advances in cognitive rehabilitation techniques. Recent Findings Advanced MRI techniques and newer PET tracers are being utilized heavily in research into cognitive impairment. Non-invasive brain stimulation (NIBS), computer-based cognitive training (CBCT), and virtual reality (VR)-based rehabilitation are playing an increasing role in recent trials of cognitive rehabilitation techniques of PSCI patients. In approaching patients with PSCI, neuropsychological assessment and neuroimaging should be undertaken to aid in the diagnosis and classification of underlying pathological processes. Aggressive vascular risk factor modification, in addition to the use of cholinesterase inhibitors, is the recommended pharmacological treatment approach in these patients. This should be accompanied by encouraging exercise and prescribing conventional cognitive rehabilitation, while attempting to incorporate newer technological treatment modalities such as NIBS, CBCT, and VR. We identified a significant need for future research into treatment modalities aimed at PSCI and vascular dementia (VaD).

C1 [Al Jerdi, Salman; Aleyadeh, Rozaleen] Qatar Fdn, Weill Cornell Med Qatar, Doha 24144, Qatar.

[Imam, Yahia] Hamad Med Corp, Doha, Qatar.

RP Al Jerdi, S (通讯作者)，Qatar Fdn, Weill Cornell Med Qatar, Doha 24144, Qatar.

EM ska2002@qatar-med.cornell.edu

RI Imam, Yahia/AAA-1602-2022

OI Imam, Yahia/0000-0003-4623-733X; Al Jerdi, Salman/0000-0003-3495-0365

CR Aminov A, 2018, J NEUROENG REHABIL, V15, DOI 10.1186/s12984-018-0370-2

[Anonymous], 2020, STROKE J CEREBRAL CI, V45, P2825

Auchus AP, 2007, NEUROLOGY, V69, P448, DOI 10.1212/01.wnl.0000266625.31615.f6

Baker JM, 2010, STROKE, V41, P1229, DOI 10.1161/STROKEAHA.109.576785

Ballard C, 2008, CURR MED RES OPIN, V24, P2561, DOI 10.1185/03007990802328142

Baltaduoniene D, 2019, TRANSL NEUROSCI, V10, DOI 10.1515/tnsci-2019-0020

BESSON JAO, 1988, INT J GERIATR PSYCH, V3, P99, DOI 10.1002/gps.930030206

Black S, 2003, STROKE, V34, P2323, DOI 10.1161/01.STR.0000091396.95360.E1

Bordet R, 2017, BMC MED, V15, DOI 10.1186/s12916-017-0869-6

Bowen A, 2013, COCHRANE DB SYST REV, DOI 10.1002/14651858.CD003586.pub3

Brewer L, 2015, BMC NEUROL, V15, DOI 10.1186/s12883-015-0466-2

Brooks WM, 1997, STROKE, V28, P1940, DOI 10.1161/01.STR.28.10.1940

Chail Amit, 2018, Ind Psychiatry J, V27, P172, DOI 10.4103/ipj.ipj_88_18

Chen ZM, 1997, LANCET, V349, P1641, DOI 10.1016/S0140-6736(97)04010-5

Chollet F, 2011, LANCET NEUROL, V10, P123, DOI 10.1016/S1474-4422(10)70314-8

Chung CSY, 2013, COCHRANE DB SYST REV, DOI 10.1002/14651858.CD008391.pub2

Cicerone KD, 2005, ARCH PHYS MED REHAB, V86, P1681, DOI 10.1016/j.apmr.2005.03.024

Colcombe S, 2003, PSYCHOL SCI, V14, P125, DOI 10.1111/1467-9280.t01-1-01430

Crosbie JH, 2007, DISABIL REHABIL, V29, P1139, DOI 10.1080/09638280600960909

das Nair R, 2016, COCHRANE DATABASE SY, V2016, pCD002293

Datta A, 2011, BRAIN STIMUL, V4, P169, DOI 10.1016/j.brs.2010.11.001

Devine ME, 2003, INT J GERIATR PSYCH, V18, P425, DOI 10.1002/gps.857

Dichgans M, 2008, LANCET NEUROL, V7, P310, DOI 10.1016/S1474-4422(08)70046-2

Dichgans M, 2017, CIRC RES, V120, P573, DOI 10.1161/CIRCRESAHA.116.308426

Dionisio A, 2018, CEREBROVASC DIS, V46, P176, DOI 10.1159/000494213

Doppeimayr M., 2007, BIOFEEDBACK, V35, P126

Eng JJ, 2014, CLIN REHABIL, V28, P731, DOI 10.1177/0269215514523631

Engelhardt E, 2020, DEMENT NEUROPSYCHOL, V5, P251

Erkinjuntti T, 2002, LANCET, V359, P1283, DOI 10.1016/S0140-6736(02)08267-3

Feigin VL, 2014, LANCET, V383, P245, DOI 10.1016/S0140-6736(13)61953-4

Ferris SH, 2003, INT PSYCHOGERIATR, V15, P215, DOI 10.1017/S1041610203009220

Fisicaro F, 2019, THER ADV NEUROL DISO, V12, DOI 10.1177/1756286419878317

Frantellizzi V, 2020, J ALZHEIMERS DIS, V2020

Gorelick PB, 2011, STROKE, V42, P2672, DOI 10.1161/STR.0b013e3182299496

Groot C, 2016, AGEING RES REV, V25, P13, DOI 10.1016/j.arr.2015.11.005

Hao ZL, 2013, SAO PAULO MED J, V131, P440, DOI [10.1590/1516-3180.20131316T2, 10.1002/14651858.CD008862.pub2]

Heiss WD, 2016, BMC MED, V14, DOI 10.1186/s12916-016-0725-0

Hesse S, 2007, RESTOR NEUROL NEUROS, V25, P9

Hoffmann T, 2010, COCHRANE DB SYST REV, DOI 10.1002/14651858.CD006430.pub2

Jin BR, 2019, NEURAL REGEN RES, V14, P805, DOI 10.4103/1673-5374.249228

Joffres C, 2000, INT PSYCHOGERIATR, V12, P403, DOI 10.1017/S1041610200006505

Johnson W, 2016, B WORLD HEALTH ORGAN, V94, P634, DOI 10.2471/BLT.16.181636

Jorge RE, 2010, ARCH GEN PSYCHIAT, V67, P187, DOI 10.1001/archgenpsychiatry.2009.185

Kang JH, 2007, BMJ-BRIT MED J, V334, P987, DOI 10.1136/bmj.39166.597836.BE

Kimura M, 2000, STROKE, V31, P1482, DOI 10.1161/01.STR.31.7.1482

Koehler R, 2011, COGNITIVE REHABILITATION THERAPY FOR TRAUMATIC BRAIN INJURY: EVALUATING THE EVIDENCE, P1

Koennecke HC, 2006, NEUROLOGY, V66, P165, DOI 10.1212/01.wnl.0000194266.55694.1e

Laver KE, 2018, STROKE, V49, pE160, DOI 10.1161/STROKEAHA.117.020275

Lefaucheur JP, 2017, CLIN NEUROPHYSIOL, V128, P56, DOI 10.1016/j.clinph.2016.10.087

Lefaucheur JP, 2014, CLIN NEUROPHYSIOL, V125, P2150, DOI 10.1016/j.clinph.2014.05.021

Legg LA, 2006, COCHRANE DB SYST REV, DOI 10.1002/14651858.CD003585.pub2

Lo JW, 2019, NEUROLOGY, V93, pE2257, DOI 10.1212/WNL.0000000000008612

Loetscher T, 2013, COCHRANE DATABASE SY, V2013

Lohse KR, 2014, PLOS ONE, V9, DOI 10.1371/journal.pone.0093318

Merriman NA, COMMUNICATION

MEYER JS, 1989, J AM GERIATR SOC, V37, P549, DOI 10.1111/j.1532-5415.1989.tb05688.x

Miptah HN, 2019, INT J GERIATR PSYCH, V34, P1114

Modig S, 2011, BMC GERIATR, V11, DOI 10.1186/1471-2318-11-55

Moretti R, 2004, INT J CLIN PRACT, V58, P346, DOI 10.1111/j.1368-5031.2004.00127.x

Moretti R, 2002, J NEUROL SCI, V203, P141, DOI 10.1016/S0022-510X(02)00280-0

Nakamura Y, 2012, DEMENT GER COGN D EX, V2, P271, DOI 10.1159/000339953

Oberlin LE, 2017, STROKE, V48, P3093, DOI 10.1161/STROKEAHA.117.017319

Orgogozo JM, 2002, STROKE, V33, P1834, DOI 10.1161/01.STR.0000020094.08790.49

Owolabi MO, 2015, CARDIOVASC J AFR, V26, pS27, DOI 10.5830/CVJA-2015-038

Pan YS, 2017, CIRCULATION, V135, P21, DOI 10.1161/CIRCULATIONAHA.116.024913

Pantoni L, 2000, J NEUROL SCI, V175, P124, DOI 10.1016/S0022-510X(00)00300-2

Pantoni L, 2005, STROKE, V36, P619, DOI 10.1161/01.STR.0000155686.73908.3e

Park JH, 2013, J STROKE, V15, P49, DOI 10.5853/jos.2013.15.1.49

Pendlebury ST, 2009, LANCET NEUROL, V8, P1006, DOI 10.1016/S1474-4422(09)70236-4

Pinto CB, 2019, NEUROREHAB NEURAL RE, V33, P643, DOI 10.1177/1545968319860483

PRICE JF, 2008, BRIT MED J, V337, P554

Reisberg B, 1994, CIBIC PLUS INTERVIEW

Renton T, 2017, PLOS ONE, V12, DOI 10.1371/journal.pone.0177290

Robert P, 2010, ALZHEIMERS RES THER, V2, DOI 10.1186/alzrt48

Roman GC, 2010, STROKE, V41, P1213, DOI 10.1161/STROKEAHA.109.570077

Sandercock P, 1997, LANCET, V349, P1569

SAPPEYMARINIER D, 1992, MAGNET RESON MED, V26, P313, DOI 10.1002/mrm.1910260211

Schjetnan AGP, 2013, STROKE RES TREAT, V2013, DOI 10.1155/2013/170256

Schneider LS, 2009, ALZ DIS ASSOC DIS, V23, P260, DOI 10.1097/WAD.0b013e31819cb760

Stasienko A., 2016, POSTEPY REHABILITACJ, V30, P67

Sun JH, 2014, ANN TRANSL MED, V2, DOI 10.3978/j.issn.2305-5839.2014.08.05

Szczepanski SM, 2013, J NEUROSCI, V33, P5411, DOI 10.1523/JNEUROSCI.4089-12.2013

Venn RD, 1983, GERONTOLOGY, V29, P185

Veronese N, 2017, J AM GERIATR SOC, V65, P1763, DOI 10.1111/jgs.14883

Vines BW, 2011, FRONT PSYCHOL, V2, DOI 10.3389/fpsyg.2011.00230

Wang F, 2015, J MOL NEUROSCI, V56, P198, DOI 10.1007/s12031-014-0480-7

Wardlaw JM, 2013, LANCET NEUROL, V12, P822, DOI 10.1016/S1474-4422(13)70124-8

Wattjes MP, 2009, RADIOLOGY, V253, P174, DOI 10.1148/radiol.2531082262

Wilcock G, 2002, INT CLIN PSYCHOPHARM, V17, P297, DOI 10.1097/00004850-200211000-00005

Wilkinson D, 2003, NEUROLOGY, V61, P479, DOI 10.1212/01.WNL.0000078943.50032.FC

Winstein CJ, 2016, STROKE, V47, pE98, DOI 10.1161/STR.0000000000000098

Xu J, 2018, MORTALITY US

Yeager Catherine A, 2010, Issues Ment Health Nurs, V31, P376, DOI 10.3109/01612840903434589

Ylikoski R, 2007, LADIS STUDY

Zhang HF, 2019, TRANSL PSYCHIAT, V9, DOI 10.1038/s41398-019-0385-x

NR 95

TC 0

Z9 0

U1 7

U2 22

PU CURRENT MEDICINE GROUP

PI PHILADELPHIA

PA 400 MARKET STREET, STE 700, PHILADELPHIA, PA 19106 USA

SN 1092-8480

EI 1534-3138

J9 CURR TREAT OPTION NE

JI Curr. Treat. Options Neurol.

PD JUN 16

PY 2020

VL 22

IS 7

AR 20

DI 10.1007/s11940-020-00627-3

PG 17

WC Clinical Neurology

WE Science Citation Index Expanded (SCI-EXPANDED); Social Science Citation Index (SSCI)

SC Neurosciences & Neurology

GA LY4SB

UT WOS:000540518300001

DA 2022-06-21

ER

PT J

AU Al-Sharman, A

Ismaiel, IA

Khalil, H

El-Salem, K

AF Al-Sharman, Alham

Ismaiel, Ismail Alhaj

Khalil, Hanan

El-Salem, Khalid

TI Exploring the Relationship Between Sleep Quality, Sleep-Related

Biomarkers, and Motor Skill Acquisition Using Virtual Reality in People

With Parkinson's Disease: A Pilot Study

SO FRONTIERS IN NEUROLOGY

LA English

DT Article

DE Parkinson&apos; s disease; motor learning; sleep; rehabilitation;

virtual reality

AB Background and Objectives: Despite the fact that sleep disturbances are among the most common and disabling manifestations of Parkinson's disease (PD), no study has investigated the effect of sleep quality and sleep-related biomarkers on motor skill acquisition in people with Parkinson's disease (PwPD).

Objective: To examine the relationship between skill acquisition, sleep quality, and sleep-related biomarkers in PwPD using virtual reality (VR) system.

Methods: This is a cross sectional study conducted on 31 PwPD and 31 healthy controls. To assess skill acquisition, each participant practiced a VR game 6 times (blocks). The main outcomes from the VR game were the required time to complete the VR game and the recorded errors. Motor skill acquisition was calculated as the difference of scores between block 6 and block 2 for both outcomes. Sleep was assessed subjectively using Pittsburgh Sleep Quality Index (PSQI) and objectively using the Actisleep. To assess sleep related biomarker, plasma serotonin level was examined.

Results: PwPD and healthy controls demonstrated a practice-related improvement in performance as shown by the main effect of block for each of the VR outcome measures (p < 0.000, time required to complete VR game; p < 0.000, recorded errors). There was no interaction effect between Block X Group for both outcome measures. There were significant correlations in both groups (p < 0.05) between motor skill acquisition (as indicated by the difference of time required to complete the VR game between block 6 and block 2) and PSQI total score, wake after sleep onset, and sleep efficiency. Additionally, a significant correlation was observed in both groups between motor skill acquisition (as indicated by the difference of time required to complete the VR game between block 6 and block 2) and the plasma serotonin level (p < 0.05). These correlations in PwPD remained significant, even after adjusting for disease motor severity, cognitive status, depression, and daily dose of L-dopa.

Discussion and Conclusions: Sleep quality may influence motor skill acquisition in PwPD. Healthcare professionals are encouraged to be aware about sleep quality and sleep assessment tools. Therapies may target improving sleep quality which could result in improving motor skill acquisition.

C1 [Al-Sharman, Alham; Ismaiel, Ismail Alhaj; Khalil, Hanan] Jordan Univ Sci & Technol, Fac Appl Med Sci, Dept Rehabil Sci, Irbid, Jordan.

[El-Salem, Khalid] Jordan Univ Sci & Technol, Dept Neurosci, Fac Med, Irbid, Jordan.

RP Al-Sharman, A (通讯作者)，Jordan Univ Sci & Technol, Fac Appl Med Sci, Dept Rehabil Sci, Irbid, Jordan.

EM ejalshorman@just.edu.jo

OI Khalil, Hanan/0000-0001-7741-8892

FU Jordan University of Science and Technology [AA-20180525]; Clinical

Rehabilitation Sciences Master Program (CRS) - Erasmus + Programme of

the European Union [573758-EPP1-2016-1-JO-EPPKA2-CBHE-JP]

FX The authors would like to acknowledge Dr. Mayis Aldughmi for reviewing

the manuscript. The authors would like to acknowledge all the

participants of the study. Acknowledgment for funding support was to

Jordan University of Science and Technology (Grant No. AA-20180525),

Clinical Rehabilitation Sciences Master Program (CRS) funded project by

the Erasmus + Programme of the European Union (Project No:

573758-EPP1-2016-1-JO-EPPKA2-CBHE-JP).

CR Al-Sharman A, 2019, NEUROREHABILITATION, V45, P107, DOI 10.3233/NRE-192748

Al-Sharman A, 2014, J AM GERIATR SOC, V62, P1797, DOI 10.1111/jgs.13002

Al-Sharman A, 2013, PHYS THER, V93, P1625, DOI 10.2522/ptj.20120502

Blischke K, 2017, FRONT HUM NEUROSCI, V11, DOI 10.3389/fnhum.2017.00374

Brawn TP, 2008, LEARN MEMORY, V15, P815, DOI 10.1101/lm.1180908

BUYSSE DJ, 1989, PSYCHIAT RES, V28, P193, DOI 10.1016/0165-1781(89)90047-4

Chahine LM, 2017, SLEEP MED REV, V35, P33, DOI 10.1016/j.smrv.2016.08.001

Dan XJ, 2015, PLOS ONE, V10, DOI 10.1371/journal.pone.0134291

ELRUFAIE OEF, 1995, SOC PSYCH PSYCH EPID, V30, P26, DOI 10.1007/BF00784431

Fischer S, 2002, P NATL ACAD SCI USA, V99, P11987, DOI 10.1073/pnas.182178199

Hall J.E., 2010, GUYTON HALL TXB MED

HALLIDAY GM, 1990, BRAIN RES, V510, P104, DOI 10.1016/0006-8993(90)90733-R

Havlikova E, 2011, J NEUROL, V258, P2222, DOI 10.1007/s00415-011-6098-6

HOEHN MM, 1967, NEUROLOGY, V17, P427, DOI 10.1212/WNL.17.5.427

Hornung OP, 2005, EXP GERONTOL, V40, P279, DOI 10.1016/j.exger.2005.02.001

Hughes JM, 2018, CLIN GERONTOLOGIST, V41, P145, DOI 10.1080/07317115.2017.1408734

JACOBS BL, 1992, PHYSIOL REV, V72, P165, DOI 10.1152/physrev.1992.72.1.165

Kanekar N, 2015, J ELECTROMYOGR KINES, V25, P400, DOI 10.1016/j.jelekin.2014.11.002

Kaseda Y, 2017, NEUROL CLIN NEUROSCI, V5, P18, DOI 10.1111/ncn3.12088

Khalil H., 2016, DEV VIRTUAL REALITY

Khalil H, 2018, NEUROREHABILITATION, V43, P473, DOI 10.3233/NRE-182471

King BR, 2017, CEREB CORTEX, V27, P1588, DOI 10.1093/cercor/bhv347

Kish SJ, 2003, ADV NEUROL, V91, P39

Kloepfer C, 2009, J CLIN SLEEP MED, V5, P540

Lee GS, 2014, J BONE MINER RES, V29, P976, DOI 10.1002/jbmr.2086

Mak MKY, 2012, PARKINSONS DIS-US, V2012, DOI 10.1155/2012/901721

MALASI TH, 1991, ACTA PSYCHIAT SCAND, V84, P323, DOI 10.1111/j.1600-0447.1991.tb03153.x

Manni R, 2017, OXF TXB SLEEP DISORD, V36, P255, DOI [10.1093/med/9780199682003.003.0026, DOI 10.1093/MED/9780199682003.003.0026]

Martinez-Martin P, 2015, PARKINSONISM RELAT D, V21, P50, DOI 10.1016/j.parkreldis.2014.10.026

Menza M, 2010, MOVEMENT DISORD, V25, pS117, DOI 10.1002/mds.22788

Molloy E., 2019, 2019 OHBM ANN M ROM

Nantel J, 2012, PARKINSONISM RELAT D, V18, P285, DOI 10.1016/j.parkreldis.2011.11.005

Nieuwboer A, 2009, PARKINSONISM RELAT D, V15, pS53, DOI 10.1016/S1353-8020(09)70781-3

Obeso JA, 2010, NAT MED, V16, P653, DOI 10.1038/nm.2165

Olson M, 2019, FRONT NEUROL, V10, DOI 10.3389/fneur.2019.00062

Peach D, 2014, PHYSIOL MEAS, V35, P2359, DOI 10.1088/0967-3334/35/12/2359

Pendt LK, 2011, PLOS ONE, V6, DOI 10.1371/journal.pone.0021669

Potter-Nerger M, 2013, MOVEMENT DISORD, V28, P1609, DOI 10.1002/mds.25677

Polotis M, 2015, BEHAV BRAIN RES, V277, P136, DOI 10.1016/j.bbr.2014.07.037

Rahman TTA, 2009, GERIATR GERONTOL INT, V9, P54, DOI 10.1111/j.1447-0594.2008.00509.x

Reed DL, 2016, J CLIN SLEEP MED, V12, P263, DOI 10.5664/jcsm.5498

Roy S, 2015, NEUROPSYCHOLOGIA, V66, P55, DOI 10.1016/j.neuropsychologia.2014.11.005

Slater JA, 2015, SLEEP BIOL RHYTHMS, V13, P172, DOI 10.1111/sbr.12103

Stavitsky K, 2010, PARKINSONISM RELAT D, V16, P280, DOI 10.1016/j.parkreldis.2010.02.001

Stephan MA, 2011, BRAIN COGNITION, V75, P135, DOI 10.1016/j.bandc.2010.10.015

Suleiman KH, 2010, WESTERN J NURS RES, V32, P250, DOI 10.1177/0193945909348230

Terpening Z, 2013, J SLEEP RES, V22, P398, DOI 10.1111/jsr.12028

Uemura Y, 2009, J NEUROL SCI, V287, P36, DOI 10.1016/j.jns.2009.09.015

Valim V, 2013, REV BRAS REUMATOL, V53, P538, DOI [10.1016/j.rbre.2013.02.001, 10.1016/j.rbr.2013.02.001]

Walker MP, 2002, NEURON, V35, P205, DOI 10.1016/S0896-6273(02)00746-8

Watkins M., 2008, CORRELATION, V3rd ed

Wilson H, 2018, NEUROIMAGE-CLIN, V18, P630, DOI 10.1016/j.nicl.2018.03.001

Wulf G, 2002, PSYCHON B REV, V9, P185, DOI 10.3758/BF03196276

NR 53

TC 2

Z9 2

U1 3

U2 8

PU FRONTIERS MEDIA SA

PI LAUSANNE

PA AVENUE DU TRIBUNAL FEDERAL 34, LAUSANNE, CH-1015, SWITZERLAND

SN 1664-2295

J9 FRONT NEUROL

JI Front. Neurol.

PD MAR 2

PY 2021

VL 12

AR 582611

DI 10.3389/fneur.2021.582611

PG 10

WC Clinical Neurology; Neurosciences

WE Science Citation Index Expanded (SCI-EXPANDED); Social Science Citation Index (SSCI)

SC Neurosciences & Neurology

GA QX0BM

UT WOS:000629014500001

PM 33737900

OA gold, Green Published

DA 2022-06-21

ER

PT J

AU Al-Sharman, A

Al-Khazaaleh, HM

Khalil, H

Aburub, A

El-Salem, K

AF Al-Sharman, Alham

Al-Khazaaleh, Heba Mefleh

Khalil, Hanan

Aburub, Ala'S

El-Salem, Khalid

TI The Relationship Between Sleep Quality, Sleep-Related Biomarkers, and

Motor Skill Acquisition in People With Multiple Sclerosis: A Pilot Study

SO PHYSICAL THERAPY

LA English

DT Article

DE Motor Learning; Multiple Sclerosis; Rehabilitation; Sleep Quality;

Virtual Reality

ID SEROTONERGIC SYSTEM; BEHAVIOR DISORDER; FATIGUE; MEMORY; QUESTIONNAIRE;

CONSOLIDATION; RELIABILITY; IMPAIRMENT; PREVALENCE; PRINCIPLES

AB Objective. Neurorehabilitation that involves learning new motor skills is one of the promising clinical methods for motor recovery in people with multiple sclerosis (PwMS); therefore, factors that influence the acquisition of motor skills in PwMS need to be investigated. Sleep disturbances are common in PwMS; however, no study has investigated the effect of sleep and sleep-related biomarkers on skill acquisition in PwMS. This study aimed to examine the effect of sleep and sleep-related biomarkers on motor acquisition in PwMS.

Methods. Forty participants with MS and 40 controls were recruited in this study. To assess motor acquisition, each participant was asked to perform a novel game through a virtual reality (VR) system 5 times (blocks). The main outcome measures for each block were the required time to complete the VR game and the recorded errors. The difference in scores between Block 5 and Block 1 for both outcomes were considered to represent motor skill acquisition. Sleep was assessed by self-report using the Pittsburgh Sleep Quality Index (PSQI) and objectively using sleep monitor technology. Serotonin level was assessed using means of enzyme-linked immunosorbent assay using plasma samples.

Results. There were significant positive correlations in both groups between motor skill acquisition and PSQI score. In PwMS, significant negative correlation between motor skill acquisition and sleep efficiency and significant positive correlation between motor skill acquisition and sleep latency were also observed. Interestingly, a significant negative correlation was observed between motor skill acquisition and the plasma serotonin level in both groups. Most of these correlations remained significant after controlling for disease severity, fatigue, baseline performance, and cognitive status.

Conclusion. Sleep quality may influence motor skill acquisition in PwMS. Circulatory serotonin level might explain this relationship.

Impact. Physical therapists are encouraged to be aware of sleep quality and sleep assessment. Sleep management strategies should be considered when treating PwMS.

C1 [Al-Sharman, Alham; Al-Khazaaleh, Heba Mefleh; Khalil, Hanan] Jordan Univ Sci & Technol, Fac Appl Med Sci, Dept Rehabil Sci, Irbid, Jordan.

[Aburub, Ala'S] Isra Univ, Fac Allied Med Sci, Phys Therapy Dept, Amman, Jordan.

[El-Salem, Khalid] Jordan Univ Sci & Technol, Fac Med, Dept Neurosci, Irbid, Jordan.

RP Al-Sharman, A (通讯作者)，Jordan Univ Sci & Technol, Fac Appl Med Sci, Dept Rehabil Sci, Irbid, Jordan.

EM ejalshorman@just.edu.jo

FU Jordan University of Science and Technology [AA/31/2019]

FX This study was funded by Jordan University of Science and Technology

(grant no. AA/31/2019).

CR Al-Sharman A, 2021, FRONT NEUROL, V12, DOI 10.3389/fneur.2021.582611

Al-Sharman A, 2019, NEUROREHABILITATION, V45, P107, DOI 10.3233/NRE-192748

Al-Sharman A, 2019, PHYSIOTHER RES INT, V24, DOI 10.1002/pri.1782

Al-Sharman A, 2014, NAT SCI SLEEP, V6, P27, DOI 10.2147/NSS.S53789

Al-Sharman A, 2013, PHYS THER, V93, P1625, DOI 10.2522/ptj.20120502

Amer H, 2018, EUR J NEUROL, V25, P290

Appleman ER, 2016, BEHAV NEUROSCI, V130, P290, DOI 10.1037/bne0000131

Ashford JW, 2019, J ALZHEIMERS DIS, V67, P923, DOI 10.3233/JAD-181106

Backhaus J, 2002, J PSYCHOSOM RES, V53, P737, DOI 10.1016/S0022-3999(02)00330-6

Blischke K, 2017, FRONT HUM NEUROSCI, V11, DOI 10.3389/fnhum.2017.00374

Borich MR, 2011, EXP BRAIN RES, V214, P619, DOI 10.1007/s00221-011-2863-0

Brawn TP, 2008, LEARN MEMORY, V15, P815, DOI 10.1101/lm.1180908

BROOKS JO, 1993, SLEEP, V16, P151, DOI 10.1093/sleep/16.2.151

Browne P, 2014, NEUROLOGY, V83, P1022, DOI 10.1212/WNL.0000000000000768

Burks JS, 2009, ANN INDIAN ACAD NEUR, V12, P296, DOI 10.4103/0972-2327.58273

BUYSSE DJ, 1989, PSYCHIAT RES, V28, P193, DOI 10.1016/0165-1781(89)90047-4

Chiou SY, 2018, J PHYSIOL-LONDON, V596, P1295, DOI 10.1113/JP275312

COHEN J, 1992, PSYCHOL BULL, V112, P155, DOI 10.1037/0033-2909.112.1.155

Dias RA, 2012, SLEEP BREATH, V16, P1255, DOI 10.1007/s11325-011-0642-6

Dutta R, 2011, PROG NEUROBIOL, V93, P1, DOI 10.1016/j.pneurobio.2010.09.005

Ferris LT, 2005, J SPORT SCI MED, V4, P354

Fetveit A, 2002, INT J GERIATR PSYCH, V17, P604, DOI 10.1002/gps.639

Fischer S, 2002, P NATL ACAD SCI USA, V99, P11987, DOI 10.1073/pnas.182178199

Fleming WE, 2005, SEMIN NEUROL, V25, P64, DOI 10.1055/s-2005-867075

Gomez-Choco MJ, 2007, MULT SCLER, V13, P805, DOI 10.1177/1352458506074644

Hall J.E., 2010, GUYTON HALL TXB MED

Hall JE., 2015, GUYTON HALL TXB MEDI, V13th

Hornung JP, 2003, J CHEM NEUROANAT, V26, P331, DOI 10.1016/j.jchemneu.2003.10.002

Huang CY, 2017, COMPLEMENT THER MED, V34, P116, DOI 10.1016/j.ctim.2017.08.015

Huang ZL, 2014, INT REV NEUROBIOL, V119, P349, DOI 10.1016/B978-0-12-801022-8.00014-3

JACOBS BL, 1992, PHYSIOL REV, V72, P165, DOI 10.1152/physrev.1992.72.1.165

JOUVET M, 1969, SCIENCE, V163, P32, DOI 10.1126/science.163.3862.32

Kallweit U, 2013, MULT SCLER INT, V2013, DOI 10.1155/2013/286581

Kanekar N, 2015, J ELECTROMYOGR KINES, V25, P400, DOI 10.1016/j.jelekin.2014.11.002

Kawashima T, 2018, NEUROSCI RES, V129, P32, DOI 10.1016/j.neures.2017.07.005

Kawashima T, 2016, CELL, V167, P933, DOI 10.1016/j.cell.2016.09.055

Kaynak H, 2006, EUR J NEUROL, V13, P1333, DOI 10.1111/j.1468-1331.2006.01499.x

Khalil H, 2020, MULT SCLER RELAT DIS, V39, DOI 10.1016/j.msard.2019.101878

Khalil H, 2018, NEUROREHABILITATION, V43, P473, DOI 10.3233/NRE-182471

Khan F, 2017, J NEUROL, V264, P603, DOI 10.1007/s00415-016-8307-9

King BR, 2017, CEREB CORTEX, V27, P1588, DOI 10.1093/cercor/bhv347

Kloepfer C, 2009, J CLIN SLEEP MED, V5, P540

Krueger JM, 2008, NAT REV NEUROSCI, V9, P910, DOI 10.1038/nrn2521

KURTZKE JF, 1983, NEUROLOGY, V33, P1444, DOI 10.1212/WNL.33.11.1444

Lee GS, 2014, J BONE MINER RES, V29, P976, DOI 10.1002/jbmr.2086

Lunde HMB, 2012, PLOS ONE, V7, DOI 10.1371/journal.pone.0049996

Malangre A, 2014, J HUM KINET, V40, P7, DOI 10.2478/hukin-2014-0002

May A, 2011, TRENDS COGN SCI, V15, P475, DOI 10.1016/j.tics.2011.08.002

McKenna JT, 2001, BRAIN RES BULL, V54, P619, DOI 10.1016/S0361-9230(01)00465-8

McKenzie IA, 2014, SCIENCE, V346, P318, DOI 10.1126/science.1254960

Mellor A, 2019, TRIALS, V20, DOI 10.1186/s13063-019-3334-3

Monti JM, 2008, PROG BRAIN RES, V172, P625, DOI 10.1016/S0079-6123(08)00929-1

Muratori LM, 2013, J HAND THER, V26, P94, DOI 10.1016/j.jht.2012.12.007

Nociti V, 2017, J NEUROL SCI, V372, P387, DOI 10.1016/j.jns.2016.10.040

Petrovic ZK, 2019, PSYCHIAT DANUB, V31, P78, DOI 10.24869/psyd.2019.78

Plazzi Giuseppe, 2002, Sleep Med, V3, P437, DOI 10.1016/S1389-9457(02)00042-4

Porkka-Heiskanen T, 2002, SLEEP MED REV, V6, P321, DOI 10.1053/smrv.2001.0201

Portney LG, FDN CLIN RES APPL PR, V3rd

Rahman TTA, 2009, GERIATR GERONTOL INT, V9, P54, DOI 10.1111/j.1447-0594.2008.00509.x

Reed DL, 2016, J CLIN SLEEP MED, V12, P263, DOI 10.5664/jcsm.5498

Rossetti HC, 2011, NEUROLOGY, V77, P1272, DOI 10.1212/WNL.0b013e318230208a

Sekeres MJ, 2017, STUD NEUROSCI, P17, DOI 10.1007/978-3-319-45066-7_2

Shrivastava D, 2014, J COMMUNITY HOSP INT, V4, DOI 10.3402/jchimp.v4.24983

SHUELL TJ, 1990, REV EDUC RES, V60, P531, DOI 10.2307/1170505

Siengsukon Catherine F, 2016, Mult Scler J Exp Transl Clin, V2, p2055217316680639, DOI 10.1177/2055217316680639

Siengsukon CF, 2011, NAT SCI SLEEP, V3, P39, DOI 10.2147/NSS.S20063

Silva-Batista C, 2017, J STRENGTH COND RES, V31, P2270, DOI 10.1519/JSC.0000000000001685

Slater JA, 2015, SLEEP BIOL RHYTHMS, V13, P172, DOI 10.1111/sbr.12103

Stenberg D, 2007, CELL MOL LIFE SCI, V64, P1187, DOI 10.1007/s00018-007-6530-3

Tacchino A, 2014, BRAIN RES, V1585, P91, DOI 10.1016/j.brainres.2014.08.031

TACHIBANA N, 1994, EUR NEUROL, V34, P320, DOI 10.1159/000117070

Tippmann-Peikert M, 2006, NEUROLOGY, V66, P1277, DOI 10.1212/01.wnl.0000208518.72660.ff

Tomassini V, 2011, MULT SCLER J, V17, P103, DOI 10.1177/1352458510381257

Trapp BD, 2008, ANNU REV NEUROSCI, V31, P247, DOI 10.1146/annurev.neuro.30.051606.094313

Vitkova M, 2016, BRAIN BEHAV, V6, DOI 10.1002/brb3.553

Walker MP, 2006, ANNU REV PSYCHOL, V57, P139, DOI 10.1146/annurev.psych.56.091103.070307

Walker MP, 2002, NEURON, V35, P205, DOI 10.1016/S0896-6273(02)00746-8

Wang DV, 2015, NAT NEUROSCI, V18, P728, DOI 10.1038/nn.3998

Wulf G, 2002, PSYCHON B REV, V9, P185, DOI 10.3758/BF03196276

Xiao L, 2016, NAT NEUROSCI, V19, P1210, DOI 10.1038/nn.4351

Zajac A, 2015, J HUM KINET, V49, P159, DOI 10.1515/hukin-2015-0118

NR 81

TC 0

Z9 0

U1 1

U2 2

PU OXFORD UNIV PRESS INC

PI CARY

PA JOURNALS DEPT, 2001 EVANS RD, CARY, NC 27513 USA

SN 0031-9023

EI 1538-6724

J9 PHYS THER

JI Phys. Therapy

PD OCT

PY 2021

VL 101

IS 10

DI 10.1093/ptj/pzab175

EA JUL 2021

PG 9

WC Orthopedics; Rehabilitation

WE Science Citation Index Expanded (SCI-EXPANDED); Social Science Citation Index (SSCI)

SC Orthopedics; Rehabilitation

GA YB8RI

UT WOS:000739271800007

PM 34270772

DA 2022-06-21

ER

PT J

AU Al-Whaibi, RM

Al-Jadid, MS

ElSerougy, HR

Badawy, WM

AF Al-Whaibi, Reem M.

Al-Jadid, Maher S.

ElSerougy, Hager R.

Badawy, Wanees M.

TI Effectiveness of virtual reality-based rehabilitation versus

conventional therapy on upper limb motor function of chronic stroke

patients: a systematic review and meta-analysis of randomized controlled

trials

SO PHYSIOTHERAPY THEORY AND PRACTICE

LA English

DT Review; Early Access

DE Stroke; virtual reality; conventional therapy; upper limb

ID MOVEMENT THERAPY; RECOVERY; ARM; STIMULATION; INTERVENTION; BIAS

AB Objective: To systematically review the available randomized controlled trials in the literature concerning the application of virtual reality (VR) rehabilitation interventions compared to conventional physical therapy, in regaining the upper limb motor function among patients with chronic stroke. Methods: A systematic electronic database search was conducted for related studies published from inauguration and until June 25, 2020 in nine databases. Another new search was done on February 1, 2021 and no new studies were identified. Results: Six studies were included in the analysis. Significant improvement was seen following the VR therapy in patients with chronic stroke, compared to their scores prior to it (SMD = 0.28; 95% CI = 0.03-0.53; p = .03). There was neither heterogeneity (I-2 = 0% and P = .5) nor a risk of bias (P = .8) among the included studies. VR interventions produced a comparable effectiveness to that of the conventional rehabilitation, with no statistically significant difference (SMD = 0.15; 95% CI = -0.14-0.44; P = .3). There was neither heterogeneity (I-2 = 40% and P = .1) nor a risk of bias (P = .5) among the included studies. Conclusions: The upper limb motor function of patients with chronic stroke who underwent VR-based rehabilitative intervention showed significant improvement as compared to the pre-treatment state. Our analysis also revealed no superiority of VR interventions over conservative therapies; however, the difference observed did not accomplish statistical significance.

C1 [Al-Whaibi, Reem M.] Princess Nourah Bint Abdulrahman Univ, Hlth & Rehabil Sci Coll, Rehabil Sci Dept, Riyadh, Saudi Arabia.

[Al-Jadid, Maher S.] Prince Sultan Mil Med Sch, Rehabil Med Dept, Riyadh, Saudi Arabia.

[ElSerougy, Hager R.] Misr Univ Sci & Technol, Fac Phys Therapy, Dept Phys Therapy Neuromuscular Disorders & Its S, Giza, Egypt.

[Badawy, Wanees M.] Cairo Univ, Fac Phys Therapy, Dept Phys Therapy Neuromuscular Disorders & Its S, Giza, Egypt.

RP Badawy, WM (通讯作者)，Cairo Univ, Fac Phys Therapy, Phys Therapy Neuromuscular Disorders & Its Surg, Giza 12613, Egypt.

EM wanees.alamir@pt.cu.edu.eg

OI Badawy, Wanees/0000-0002-9814-4162

FU Deanship of Scientific Research at Princess Nourah bint Abdulrahman

University, Saudi Arabia [RGP1440-0012]

FX This work was supported by the Deanship of Scientific Research at

Princess Nourah bint Abdulrahman University, Saudi Arabia, through the

Research Groups Program [RGP1440-0012].

CR Ackerley SJ, 2016, NEUROREHAB NEURAL RE, V30, P339, DOI 10.1177/1545968315595285

Ahmad MA, 2019, INT J ENV RES PUB HE, V16, DOI 10.3390/ijerph16245144

Ahn S, 2019, J EXERC REHABIL, V15, P358, DOI 10.12965/jer.1938174.087

Arya KN, 2011, J BODYW MOV THER, V15, P528, DOI 10.1016/j.jbmt.2011.01.023

Bailey RR, 2015, NEUROREHAB NEURAL RE, V29, P969, DOI 10.1177/1545968315583720

Baumann M, 2011, TOP STROKE REHABIL, V18, P162, DOI 10.1310/tsr1802-162

Brewer L, 2013, QJM-INT J MED, V106, P11, DOI 10.1093/qjmed/hcs174

Cambier DC, 2003, CLIN REHABIL, V17, P14, DOI 10.1191/0269215503cr580oa

Chen JC, 2005, STROKE, V36, P2665, DOI 10.1161/01.STR.0000189992.06654.ab

Chen JC, 2014, WORLD J CLIN CASES, V2, P316, DOI 10.12998/wjcc.v2.i8.316

CHEN L, 2016, BIOMED RES INT, V2016, DOI DOI 10.1155/2016/7309272

Choo PL, 2015, BRAIN BEHAV, V5, DOI 10.1002/brb3.411

Crosbie JH, 2012, CLIN REHABIL, V26, P798, DOI 10.1177/0269215511434575

Cameirao MDS, 2011, RESTOR NEUROL NEUROS, V29, P287, DOI 10.3233/RNN-2011-0599

Ribeiro NMD, 2015, TOP STROKE REHABIL, V22, P299, DOI 10.1179/1074935714Z.0000000017

Dobkin BH, 2013, CURR ATHEROSCLER REP, V15, DOI 10.1007/s11883-013-0331-y

Doyle S, 2010, COCHRANE DB SYST REV, DOI 10.1002/14651858.CD006331.pub2

Egger M, 1997, BMJ-BRIT MED J, V315, P629, DOI 10.1136/bmj.315.7109.629

El-Qushayri AE, 2020, INT J INFECT DIS, V92, P218, DOI 10.1016/j.ijid.2019.12.030

French B, 2007, COCHRANE DB SYST REV, DOI 10.1002/14651858.CD006073.pub2

Gibbons EM, 2016, TOP STROKE REHABIL, V23, P440, DOI 10.1080/10749357.2016.1183349

Hedges LV, 2013, RES SYNTH METHODS, V4, P324, DOI 10.1002/jrsm.1086

Higgins JPT., 2022, COCHRANE HDB SYSTEMA

Hosp JA, 2011, NEURAL PLAST, V2011, DOI 10.1155/2011/871296

Hsu WY, 2012, STROKE, V43, P1849, DOI 10.1161/STROKEAHA.111.649756

Iruthayarajah J, 2017, TOP STROKE REHABIL, V24, P68, DOI 10.1080/10749357.2016.1192361

Jarvis K, 2014, BRIT J OCCUP THER, V77, P126, DOI 10.4276/030802214X13941036266469

Johansson BB, 2011, ACTA NEUROL SCAND, V123, P147, DOI 10.1111/j.1600-0404.2010.01417.x

Johnson CO, 2019, LANCET NEUROL, V18, P439, DOI 10.1016/S1474-4422(19)30034-1

Kim Ju-Hong, 2018, J Phys Ther Sci, V30, P1408, DOI 10.1589/jpts.30.1408

Kita K, 2013, J NEUROENG REHABIL, V10, DOI 10.1186/1743-0003-10-55

Kleim JA, 2008, J SPEECH LANG HEAR R, V51, pS225, DOI 10.1044/1092-4388(2008/018)

Kraemer WJ, 2002, J STRENGTH COND RES, V16, P373

Langhorne P, 2011, LANCET, V377, P1693, DOI 10.1016/S0140-6736(11)60325-5

Laver KE, 2011, COCHRANE DB SYST REV, DOI [10.1002/14651858.CD008349.pub2, 10.1002/14651858.CD008349.pub4, 10.1002/14651858.CD008349.pub3]

Lee HS, 2019, BIOMED RES INT, V2019, DOI 10.1155/2019/7595639

Levin MF, 2015, PHYS THER, V95, P415, DOI 10.2522/ptj.20130579

Lohr K, 1990, I MED US DIVISION HL, P179

Lohse KR, 2014, PLOS ONE, V9, DOI 10.1371/journal.pone.0093318

Mekbib DB, 2020, BRAIN INJURY, V34, P456, DOI 10.1080/02699052.2020.1725126

Merians AS, 2002, PHYS THER, V82, P898, DOI 10.1093/ptj/82.9.898

Molier BI, 2010, DISABIL REHABIL, V32, P1799, DOI 10.3109/09638281003734359

Nijland R, 2011, INT J STROKE, V6, P425, DOI 10.1111/j.1747-4949.2011.00646.x

Peters JL, 2006, JAMA-J AM MED ASSOC, V295, P676, DOI 10.1001/jama.295.6.676

Pollock A, 2007, CLIN REHABIL, V21, P395, DOI 10.1177/0269215507073438

Roger VL, 2012, CIRCULATION, V125, P188, DOI 10.1161/CIR.0b013e3182456d46

Saposnik G, 2016, LANCET NEUROL, V15, P1019, DOI 10.1016/S1474-4422(16)30121-1

Saposnik G, 2011, STROKE, V42, P1380, DOI 10.1161/STROKEAHA.110.605451

Schuster-Amft C, 2018, PLOS ONE, V13, DOI 10.1371/journal.pone.0204455

Simpkins AN, 2020, TRANSL STROKE RES, V11, P615, DOI 10.1007/s12975-019-00762-3

Sterne JAC, 2019, BMJ-BRIT MED J, V366, DOI 10.1136/bmj.l4898

Stockley RC, 2017, REHABIL RES PRACT, V2017, DOI 10.1155/2017/9569178

Subramanian SK, 2013, NEUROREHAB NEURAL RE, V27, P13, DOI 10.1177/1545968312449695

Takeshima N, 2014, BMC MED RES METHODOL, V14, DOI 10.1186/1471-2288-14-30

Takeuchi N, 2012, STROKE RES TREAT, V2012, DOI 10.1155/2012/584727

Takeuchi N, 2012, NEURAL PLAST, V2012, DOI 10.1155/2012/359728

Talelli P, 2006, CLIN NEUROPHYSIOL, V117, P1641, DOI 10.1016/j.clinph.2006.01.016

Tinga AM, 2016, NEUROPSYCHOL REV, V26, P73, DOI 10.1007/s11065-015-9301-1

Vassar M, 2016, J MED LIBR ASSOC, V104, P302, DOI 10.3163/1536-5050.104.4.009

Veerbeek JM, 2014, PLOS ONE, V9, DOI 10.1371/journal.pone.0087987

Wechsler LR, 2018, STROKE, V49, P1066, DOI 10.1161/STROKEAHA.117.018290

Wu HC, 2010, STROKE, V41, P2378, DOI 10.1161/STROKEAHA.110.593673

Zorowitz R, 2011, STROKE, V42, P294, DOI 10.1161/STROKEAHA.110.605063

NR 63

TC 1

Z9 1

U1 2

U2 3

PU TAYLOR & FRANCIS INC

PI PHILADELPHIA

PA 530 WALNUT STREET, STE 850, PHILADELPHIA, PA 19106 USA

SN 0959-3985

EI 1532-5040

J9 PHYSIOTHER THEOR PR

JI Physiother. Theory Pract.

DI 10.1080/09593985.2021.1941458

EA JUL 2021

PG 15

WC Rehabilitation

WE Science Citation Index Expanded (SCI-EXPANDED)

SC Rehabilitation

GA TP9XH

UT WOS:000677944500001

PM 34315320

DA 2022-06-21

ER

PT J

AU Alamri, A

Eid, M

Iglesias, R

Shirmohammadi, S

El Saddik, A

AF Alamri, Atif

Eid, Mohamad

Iglesias, Rosa

Shirmohammadi, Shervin

El Saddik, Abdulmotaleb

TI Haptic virtual rehabilitation exercises for poststroke diagnosis

SO IEEE TRANSACTIONS ON INSTRUMENTATION AND MEASUREMENT

LA English

DT Article

DE haptic applications; medical instrumentation and measurement;

occupational therapy; stroke rehabilitation; virtual reality (VR)

ID REALITY; STROKE; TELEREHABILITATION; ENVIRONMENTS; RECOVERY; SYSTEM

AB Nowadays, stroke is one of the most frequent causes of severe adult disability in the world. Virtual reality and haptic technologies have emerged as promising assistive tools for effective diagnosis and rehabilitation intervention. The objective of this paper is to develop and test a set of five virtual exercises on top of a framework, which is designed for the diagnosis and rehabilitation of patients with hand impairments. We have implemented task-oriented exercises based on well-established and common exercises, namely the Jebsen Test of Hand Function and the Box and Block Test. These include moving a cup, arranging blocks, navigating a maze, training with a dumbbell, and grasping a rubber ball. Furthermore, key performance measures (metrics) are proposed for each exercise to quantitatively evaluate and judge the performance of stroke patients. Our evaluation of these exercises shows promising potential to define "golden" reference metrics for healthy subjects, against which the performance of a patient is compared. This will facilitate the ability of occupational therapists to assess the patient's progress.

C1 [Alamri, Atif; Eid, Mohamad; Iglesias, Rosa; Shirmohammadi, Shervin; El Saddik, Abdulmotaleb] Univ Ottawa, Sch Informat Technol & Engn, Ottawa, ON K1N 6N5, Canada.

[Iglesias, Rosa] Ikerlan, Arrasate Mondragon 20500, Spain.

RP Alamri, A (通讯作者)，Univ Ottawa, Sch Informat Technol & Engn, Ottawa, ON K1N 6N5, Canada.

EM riglesias@ikerlan.es

RI El Saddik, Abdulmotaleb/D-4159-2009; Shirmohammadi, Shervin/E-6945-2012

OI El Saddik, Abdulmotaleb/0000-0002-7690-8547; Shirmohammadi,

Shervin/0000-0002-3973-4445

CR Bardorfer A, 2001, IEEE-ASME T MECH, V6, P253, DOI 10.1109/3516.951363

Boian RF, 2003, 11TH SYMPOSIUM ON HAPTIC INTERFACES FOR VIRTUAL ENVIRONMENT AND TELEOPERATOR SYSTEMS - HAPTICS 2003, PROCEEDINGS, P247, DOI 10.1109/HAPTIC.2003.1191289

Bouzit M, 2002, IEEE-ASME T MECH, V7, P256, DOI 10.1109/TMECH.2002.1011262

Brandt E, 1997, EN AM ASS ROL REH SC

Broeren J, 2002, CYBERPSYCHOL BEHAV, V5, P207, DOI 10.1089/109493102760147196

Burdea G.C., 2003, VIRTUAL REALITY TECH, V2nd

Burns RB, 1998, ASSIST TECHNOL, V10, P126, DOI 10.1080/10400435.1998.10131970

Feigin VL, 2005, LANCET, V365, P2160, DOI 10.1016/S0140-6736(05)66755-4

Holden M., 1999, NEUROLOGY REPORT, V23, P57

Holden MK, 2005, CYBERPSYCHOL BEHAV, V8, P187, DOI 10.1089/cpb.2005.8.187

Jack D, 2001, IEEE T NEUR SYS REH, V9, P308, DOI 10.1109/7333.948460

JEBSEN R H, 1969, Archives of Physical Medicine and Rehabilitation, V50, P311

Kizony R, 2003, J VISUAL COMP ANIMAT, V14, P261, DOI 10.1002/vis.323

Kline T, 2005, INT C REHAB ROBOT, P78

Koyanagi K., 2005, P 15 INT C ART REAL, P91

Langhorne P, 1996, Physiother Res Int, V1, P75

Loureiro R., 2001, P 1 EUR C HAPT EUR 2, P1

MATHIOWETZ V, 1985, AM J OCCUP THER, V39, P386, DOI 10.5014/ajot.39.6.386

MCLAUGHLIN M, 2005, P IPSI CAMBR MA

Merians AS, 2006, NEUROREHAB NEURAL RE, V20, P252, DOI 10.1177/1545968306286914

Murray CJL, 1997, LANCET, V349, P1498, DOI 10.1016/S0140-6736(96)07492-2

PARANJAPE RP, 2006, P IEEE EMBS ANN INT, P3958

Popescu VG, 2000, IEEE T INF TECHNOL B, V4, P45, DOI 10.1109/4233.826858

Riva G, 2005, CYBERPSYCHOL BEHAV, V8, P220, DOI 10.1089/cpb.2005.8.220

Rose FD, 2005, CYBERPSYCHOL BEHAV, V8, P241, DOI 10.1089/cpb.2005.8.241

Shakra I, 2006, IEEE IMTC P, P1178, DOI 10.1109/IMTC.2006.328445

Shakra I, 2006, IEEE INT WORK MED ME, P98, DOI 10.1109/MEMEA.2006.1644470

Srinivasan MA, 1997, COMPUT GRAPH-UK, V21, P393, DOI 10.1016/S0097-8493(97)00030-7

*VIRT TECHN INC, 2001, VIRT US PROGR GUID

You SH, 2005, STROKE, V36, P1166, DOI 10.1161/01.STR.0000162715.43417.91

[No title captured]

NR 31

TC 46

Z9 47

U1 1

U2 14

PU IEEE-INST ELECTRICAL ELECTRONICS ENGINEERS INC

PI PISCATAWAY

PA 445 HOES LANE, PISCATAWAY, NJ 08855-4141 USA

SN 0018-9456

EI 1557-9662

J9 IEEE T INSTRUM MEAS

JI IEEE Trans. Instrum. Meas.

PD SEP

PY 2008

VL 57

IS 9

BP 1876

EP 1884

DI 10.1109/TIM.2008.919878

PG 9

WC Engineering, Electrical & Electronic; Instruments & Instrumentation

WE Science Citation Index Expanded (SCI-EXPANDED)

SC Engineering; Instruments & Instrumentation

GA 340AG

UT WOS:000258617800008

DA 2022-06-21

ER

PT J

AU Alashram, AR

Giuseppe, A

Raju, M

Padua, E

AF Alashram, Anas R.

Giuseppe, Annino

Raju, Manikandan

Padua, Elvira

TI Effects of physical therapy interventions on balance ability in people

with traumatic brain injury: A systematic review

SO NEUROREHABILITATION

LA English

DT Review

DE Brain injuries; trauma; postural balance; rehabilitation; therapeutics;

systematic review; neurological rehabilitation

ID VIRTUAL-REALITY; EXERCISE PROGRAMS; POSTURAL SWAY; REHABILITATION;

MOTOR; PERFORMANCE; CONCUSSION; MOBILITY; DEFICITS; STROKE

AB BACKGROUND: Balance deficits are common impairments in individuals with post-traumatic brain injury (TBI). Balance deficits can restrict the activities of daily living and productive participation in social life. To date, no systematic reviews have examined the impact of physical therapy intervention on balance post-TBI.

OBJECTIVE: To examine the effects of physical therapy interventions on balance impairments in individuals with TBI.

METHODS: We systematically searched in PubMed, EMBASE, Scopus, PEDro, MEDLINE, REHABDATA, and Web of Science for randomized controlled trials (RCTs), clinical control trials, and pilot studies that examined the effects of physical therapy interventions on balance deficits in individuals post-TBI. The methodological quality was estimated using the Physiotherapy Evidence Database (PEDro) scale.

RESULTS: Eight studies published from 2003 to 2019 were included in this study. A total of 259 TBI participants post-TBI were included in this review, 71 (27.41%) of which were females. The methodological quality of the selected studies ranged from low to high. There were no significant differences between experimental interventions, virtual reality (VR), vestibular rehabilitation therapy (VRT), control group interventions, and other traditional physical therapy interventions.

CONCLUSIONS: The evidence about the effects of the physical therapy interventions in improving the balance ability post-TBI was limited. Further randomized controlled trials are strongly warranted to understand the role of physical therapy in patients with TBI who complain about balance deficits.

C1 [Alashram, Anas R.] Univ Roma Tor Vergata, Fac Med & Surg, PhD Sch Neurosci, Rome, Italy.

[Alashram, Anas R.; Giuseppe, Annino] Univ Roma Tor Vergata, Dept Med Syst, Rome, Italy.

[Raju, Manikandan] Univ Sapienza, Dept Neurosci Umane, Sch Clin Expt Neurosci & Psychol, Rome, Italy.

[Padua, Elvira] San Raffaele Roma Open Univ, Dept Human Sci & Promot Qual Life, Rome, Italy.

RP Alashram, AR (通讯作者)，Univ Roma Tor Vergata, Neurosci PhD Sch, Dept Med Syst, Fac Med & Surg, Montpellier St, I-00133 Rome, Italy.

EM anasalashram@gmail.com

RI Alashram, Anas/AAU-4481-2021

OI Alashram, Anas/0000-0002-3066-3943

CR Alashram A., 2020, J CLIN NEUROSCIENCE

Alashram AR, 2019, J CLIN NEUROSCI, V69, P287, DOI 10.1016/j.jocn.2019.08.080

Alashram AR, 2019, AM J PHYS MED REHAB, V98, P1084, DOI 10.1097/PHM.0000000000001252

Black K, 2000, BRAIN INJURY, V14, P141, DOI 10.1080/026990500120808

Booth M, 2019, INT J ATHL THER TRAI, V24, P100, DOI 10.1123/ijatt.2018-0057

Broderick P, 2018, GAIT POSTURE, V63, P208, DOI 10.1016/j.gaitpost.2018.05.017

Chen BL, 2015, PLOS ONE, V10, DOI 10.1371/journal.pone.0135932

Cohen J., 1988, Statistical Power Analysis for the Behavioral Sciences, V2nd

Cuff S, 2014, CLIN J SPORT MED, V24, P186

Cuthbert JP, 2014, BRAIN INJURY, V28, P181, DOI 10.3109/02699052.2013.860475

Damiano DL, 2016, EXP BRAIN RES, V234, P2245, DOI 10.1007/s00221-016-4630-8

Egger M, 1998, BMJ-BRIT MED J, V316, P61, DOI 10.1136/bmj.316.7124.61

Feld JA, 2001, NEUROREHAB NEURAL RE, V15, P239, DOI 10.1177/154596830101500312

Greenwald BD, 2001, J HEAD TRAUMA REHAB, V16, P238, DOI 10.1097/00001199-200106000-00003

Higgins J, 2008, COCHRANE HDB SYSTEMA

Hsieh CL, 2002, STROKE, V33, P2626, DOI 10.1161/01.STR.0000033930.05931.93

Ialongo C, 2016, BIOCHEM MEDICA, V26, P150, DOI 10.11613/BM.2016.015

Kleffelgaard I, 2019, CLIN REHABIL, V33, P74, DOI 10.1177/0269215518791274

Lauber B, 2014, EUR J SPORT SCI, V14, P36, DOI 10.1080/17461391.2012.725104

Liberati A, 2009, BMJ-BRIT MED J, V339, DOI [10.1371/journal.pmed.1000100, 10.1136/bmj.b2700, 10.7326/0003-4819-151-4-200908180-00136]

Maher CG, 2003, PHYS THER, V83, P713, DOI 10.1093/ptj/83.8.713

Mann GC, 1996, J VESTIBUL RES-EQUIL, V6, P343

Marsh NV, 2016, NEUROREHABILITATION, V38, P71, DOI 10.3233/NRE-151297

Murray DA, 2017, BRIT J SPORT MED, V51, P442, DOI 10.1136/bjsports-2016-096081

Peterka RJ, 2002, J NEUROPHYSIOL, V88, P1097, DOI 10.1152/jn.2002.88.3.1097

Peters DM, 2014, ARCH PHYS MED REHAB, V95, P1454, DOI 10.1016/j.apmr.2014.04.006

Peterson Michelle, 2015, Arch Phys Med Rehabil, V96, P379, DOI 10.1016/j.apmr.2013.06.012

Pietrzak E, 2014, GAMES HEALTH J, V3, P202, DOI 10.1089/g4h.2014.0013

Portney L., 2009, FDN CLIN RES, V(3rd ed.)

SACKETT DL, 1989, CHEST, V95, pS2, DOI 10.1378/chest.95.2_Supplement.2S

Schmid AA, 2012, STROKE, V43, P2402, DOI 10.1161/STROKEAHA.112.658211

Shaffer SW, 2007, PHYS THER, V87, P193, DOI 10.2522/ptj.20060083

SHUMWAYCOOK A, 1988, ARCH PHYS MED REHAB, V69, P395

Straudi S, 2017, BMC NEUROL, V17, DOI 10.1186/s12883-017-0871-9

Sveistrup H, 2003, CYBERPSYCHOL BEHAV, V6, P245, DOI 10.1089/109493103322011524

Thornton M, 2005, BRAIN INJURY, V19, P989, DOI 10.1080/02699050500109944

Ustinova KI, 2015, PHYSIOTHER THEOR PR, V31, P1, DOI 10.3109/09593985.2014.945674

Wade LD, 1997, ARCH PHYS MED REHAB, V78, P1107, DOI 10.1016/S0003-9993(97)90136-2

Walker WC, 2007, J REHABIL RES DEV, V44, P975, DOI 10.1682/JRRD.2006.12.0158

Wulf G, 2013, INT REV SPORT EXER P, V6, P77, DOI 10.1080/1750984X.2012.723728

Xu L, 2018, MEDICINE, V97, DOI 10.1097/MD.0000000000011681

NR 41

TC 2

Z9 2

U1 0

U2 10

PU IOS PRESS

PI AMSTERDAM

PA NIEUWE HEMWEG 6B, 1013 BG AMSTERDAM, NETHERLANDS

SN 1053-8135

EI 1878-6448

J9 NEUROREHABILITATION

JI Neurorehabilitation

PY 2020

VL 46

IS 4

BP 455

EP 466

DI 10.3233/NRE-203047

PG 12

WC Clinical Neurology; Rehabilitation

WE Science Citation Index Expanded (SCI-EXPANDED); Social Science Citation Index (SSCI)

SC Neurosciences & Neurology; Rehabilitation

GA MM3DS

UT WOS:000550036100002

PM 32508337

OA Bronze, Green Published

DA 2022-06-21

ER

PT J

AU Alashram, AR

Annino, G

Padua, E

Romagnoli, C

Mercuri, NB

AF Alashram, Anas R.

Annino, Giuseppe

Padua, Elvira

Romagnoli, Cristian

Mercuri, Nicola Biagio

TI Cognitive rehabilitation post traumatic brain injury: A systematic

review for emerging use of virtual reality technology

SO JOURNAL OF CLINICAL NEUROSCIENCE

LA English

DT Review

DE Virtual reality; Cognition; Therapy; Traumatic brain injury;

Rehabilitation

ID OF-THE-LITERATURE; MEMORY; DYSFUNCTION; EXERCISE; PEOPLE; PROOF; TBI

AB Background: Traumatic brain injury (TBI) can causes numerous cognitive impairments usually in the aspects of problem-solving, executive function, memory, and attention. Several studies has suggested that rehabilitation treatment interventions can be effective in treating cognitive symptoms of brain injury. Virtual reality (VR) technology potential as a useful tool for the assessment and rehabilitation of cognitive processes.

Objectives: The aims of present systematic review are to examine effects of VR training intervention on cognitive function, and to identify effective VR treatment protocol in patients with TBI.

Methods: PubMed, Scopus, PEDro, REHABDATA, EMBASE, web of science, and MEDLINE were searched for studies investigated effect of VR on cognitive functions post TBI. The methodological quality were evaluated using PEDro scale. The results of selected studies were summarized.

Results: Nine studies were included in present study. Four were randomized clinical trials, case studies (n = 3), prospective study (n = 1), and pilot study (n = 1). The scores on the PEDro ranged from 0 to 7 with a mean score of 3. The results showed improvement in various cognitive function aspects such as; memory, executive function, and attention in patients with TBI after VR training.

Conclusion: Using different VR tools with following treatment protocol; 10-12 sessions, 20-40 min in duration with 2-4 sessions per week may improves cognitive function in patients with TBI. There was weak evidence for effects of VR training on attention post TBI. (C) 2019 Elsevier Ltd. All rights reserved.

C1 [Alashram, Anas R.; Mercuri, Nicola Biagio] Univ Roma Tor Vergata, Fac Med & Surg, Dept Neurol, Rome, Italy.

[Annino, Giuseppe; Mercuri, Nicola Biagio] Univ Roma Tor Vergata, Dept Med Syst, Rome, Italy.

[Annino, Giuseppe; Padua, Elvira] San Raffaele Roma Open Univ, Dept Human Sci & Promot Qual Life, Rome, Italy.

[Romagnoli, Cristian] Alma Mater Univ, PhD Sch Sci & Culture Well Being & Lifestyle, Bologna, Italy.

RP Alashram, AR (通讯作者)，Via Cambridge 115, Rome, Italy.

EM anasradihassan.alashram@students.uniroma2.eu;

elvira.padua@unisanraffaele.gov.it; cristian-romagnoli@outlook.it;

mercurin@med.uniroma2.it

RI Padua, Elvira/AAO-3237-2020; Romagnoli, Cristian/AGQ-5230-2022; Mercuri,

Nicola Biagio/K-5348-2016; Alashram, Anas/AAU-4481-2021

OI Mercuri, Nicola Biagio/0000-0001-6700-7491; Alashram,

Anas/0000-0002-3066-3943; Padua, Elvira/0000-0001-5227-2567; ROMAGNOLI,

CRISTIAN/0000-0003-0904-634X

CR Allred RP, 2005, RESTOR NEUROL NEUROS, V23, P297

[Anonymous], 1978, EDUC RESEARCHER, DOI [10.3102/0013189x007002005, DOI 10.3102/0013189X007002005]

Caglio M, 2012, NEUROCASE, V18, P123, DOI 10.1080/13554794.2011.568499

Caglio M, 2009, COGN PROCESS, V10, pS195, DOI 10.1007/s10339-009-0295-6

Centre for Reviews and Dissemination, 2009, CRDS GUID UND REV HE

Chevignard M, 2000, CORTEX, V36, P649, DOI 10.1016/S0010-9452(08)70543-4

Cicerone KD, 2005, ARCH PHYS MED REHAB, V86, P1681, DOI 10.1016/j.apmr.2005.03.024

Cicerone KD, 2000, ARCH PHYS MED REHAB, V81, P1596, DOI 10.1053/apmr.2000.19240

Cicerone KD, 2011, ARCH PHYS MED REHAB, V92, P519, DOI 10.1016/j.apmr.2010.11.015

Dahdah MN, 2017, NEUROREHABILITATION, V41, P721, DOI 10.3233/NRE-172183

De Luca R, 2018, INT J NEUROSCI, V128, P791, DOI 10.1080/00207454.2017.1403915

Dvorkin AY, 2013, J NEUROENG REHABIL, V10, DOI 10.1186/1743-0003-10-92

Gamito P., 2011, INT J DISABIL HUM DE, V10, P309, DOI [10.1515/IJDHD.2011.049, DOI 10.1515/IJDHD.2011.049]

Ghai S, 2017, PHYS THER SPORT, V25, P65, DOI 10.1016/j.ptsp.2016.05.006

Giovannetti T, 2007, J CLIN EXP NEUROPSYC, V29, P690, DOI 10.1080/13803390600932286

Grealy MA, 1999, ARCH PHYS MED REHAB, V80, P661, DOI 10.1016/S0003-9993(99)90169-7

Groswasser Z, 1990, INTEGRATIVE ASPECTS, P143

Holden MK, 2005, CYBERPSYCHOL BEHAV, V8, P187, DOI 10.1089/cpb.2005.8.187

Hoofien D, 2001, BRAIN INJURY, V15, P189

Jacobsen D, 2002, VAD HUR VARFOR

Jacoby M, 2013, IEEE T NEUR SYS REH, V21, P182, DOI 10.1109/TNSRE.2012.2235184

Knight RG, 2009, BRAIN IMPAIR, V10, P3, DOI 10.1375/brim.10.1.3

Larson EB, 2014, NEUROREHABILITATION, V34, P759, DOI 10.3233/NRE-141078

Maggio MG, 2019, J CLIN NEUROSCI, V61, P1, DOI 10.1016/j.jocn.2018.12.020

Maher CG, 2003, PHYS THER, V83, P713, DOI 10.1093/ptj/83.8.713

Man DWK, 2013, BRAIN INJURY, V27, P1016, DOI 10.3109/02699052.2013.794969

Maye S, 2005, HEAD INJURY, P483

Moher D, 2009, ANN INTERN MED, V151, P264, DOI 10.7326/0003-4819-151-4-200908180-00135

Pietrzak E, 2014, GAMES HEALTH J, V3, P202, DOI 10.1089/g4h.2014.0013

Portney L.G., 2009, FDN CLIN RES APPL PR, VVolume 892

Riva G, 2003, EFFECTS MEASUREMENT

Rizzo A, 2005, PRESENCE-TELEOP VIRT, V14, P119, DOI 10.1162/1054746053967094

Rizzo AA, 2004, NEUROPSYCHOL REHABIL, V14, P207, DOI 10.1080/09602010343000183

Rizzo AA, 1997, J HEAD TRAUMA REHAB, V12, P1, DOI 10.1097/00001199-199712000-00002

Rizzolatti G, 2014, PHILOS T R SOC B, V369, DOI 10.1098/rstb.2013.0420

Robitaille N, 2017, DISABIL REHABIL-ASSI, V12, P758, DOI 10.1080/17483107.2016.1229048

Rose FD, 2005, CYBERPSYCHOL BEHAV, V8, P241, DOI 10.1089/cpb.2005.8.241

ROSE FD, 1994, MED SCI RES, V22, P82

Salisbury David B, 2016, Proc (Bayl Univ Med Cent), V29, P124

Saposnik G, 2010, STROKE, V41, P1477, DOI 10.1161/STROKEAHA.110.584979

Schneider M, 2008, PSYCHOL SPORT EXERC, V9, P1, DOI 10.1016/j.psychsport.2007.01.003

Schultheis MT, 2001, REHABIL PSYCHOL, V46, P296, DOI 10.1037/0090-5550.46.3.296

Schwartz MF, 1998, NEUROPSYCHOLOGY, V12, P13, DOI 10.1037/0894-4105.12.1.13

Soy S. K., 1997, CASE STUDY RES UNPUB

Thornton M, 2005, BRAIN INJURY, V19, P989, DOI 10.1080/02699050500109944

Vakil E, 2005, J CLIN EXP NEUROPSYC, V27, P977, DOI 10.1080/13803390490919245

Wang PJ, 2004, COMPUT METH PROG BIO, V74, P235, DOI 10.1016/j.cmpb.2003.08.001

Whyte J, 2006, NEUROPSYCHOLOGIA, V44, P2007, DOI 10.1016/j.neuropsychologia.2006.02.012

Yip BCB, 2009, BRAIN INJURY, V23, P1017, DOI 10.3109/02699050903379412

Yuen Y. P., 2007, Hong Kong Medical Journal, V13, P216

Zanier ER, 2018, FRONT NEUROL, V9, DOI 10.3389/fneur.2018.00345

NR 51

TC 19

Z9 20

U1 8

U2 36

PU ELSEVIER SCI LTD

PI OXFORD

PA THE BOULEVARD, LANGFORD LANE, KIDLINGTON, OXFORD OX5 1GB, OXON, ENGLAND

SN 0967-5868

EI 1532-2653

J9 J CLIN NEUROSCI

JI J. Clin. Neurosci.

PD AUG

PY 2019

VL 66

BP 209

EP 219

DI 10.1016/j.jocn.2019.04.026

PG 11

WC Clinical Neurology; Neurosciences

WE Science Citation Index Expanded (SCI-EXPANDED); Social Science Citation Index (SSCI)

SC Neurosciences & Neurology

GA IM0PA

UT WOS:000477689500036

PM 31085075

DA 2022-06-21

ER

PT J

AU Alashram, AR

Padua, E

Hammash, AK

Lombardo, M

Annino, G

AF Alashram, Anas R.

Padua, Elvira

Hammash, Ahmad K.

Lombardo, Mauro

Annino, Giuseppe

TI Effectiveness of virtual reality on balance ability in individuals with

incomplete spinal cord injury: A systematic review

SO JOURNAL OF CLINICAL NEUROSCIENCE

LA English

DT Review

DE Virtual reality; Spinal cord injury; Rehabilitation; Balance; Therapy

ID STROKE PATIENTS; REHABILITATION; THERAPY; GAIT; QUALITY; PEOPLE; WII

AB Background: Balance dysfunctions are one of the most prevalent impairments post incomplete spinal cord injury (SCI). The evidence has proposed that the rehabilitation can be efficacious in treating balance dysfunctions in patients with SCI. Virtual reality (VR) is a computer technology designate 3-D setting which provides immersed users to generate numerous feedbacks such as visual, audio, and haptic.

Objective: To investigate the effects of VR on balance ability in individuals with incomplete SCI and to identify efficient training protocol.

Methods: We searched in SCOPUS, PEDro, PUBMED, REHABDATA, EMBASE, and web of science for experimental trials studying impacts of VR training on balance in patients with incomplete SCI that published in English. Physiotherapy Evidence Database (PEDro) scale was used to evaluate the methodological quality for selected studies.

Results: Five pilot studies were met the inclusion criteria. The PEDro scores ranged from 2 to 3, with a median of 2. All selected studies enrolled less than 20 patients. The findings showed beneficial effects of VR in improving balance ability in patients with incomplete SCI.

Conclusions: The preliminary findings showed that the influence of VR training on the balance ability in patients with incomplete SCI is promising. Applying 12 to 20 sessions of 30 to 60 min of VR training may show meaningful effects. Further randomized controlled trials strongly needed. (C) 2020 Elsevier Ltd. All rights reserved.

C1 [Alashram, Anas R.] Univ Roma Tor Vergata, PhD Sch Neurosci, Fac Med & Surg, Rome, Italy.

[Alashram, Anas R.; Annino, Giuseppe] Univ Roma Tor Vergata, Dept Med Syst, Rome, Italy.

[Padua, Elvira; Lombardo, Mauro; Annino, Giuseppe] San Raffaele Roma Open Univ, Dept Human Sci & Promot Qual Life, Rome, Italy.

[Hammash, Ahmad K.] Hamad Med Corp, Ambulance Serv, Doha, Qatar.

RP Alashram, AR (通讯作者)，Via Cambridge 115, Rome, Italy.

EM anasradihassan.alashram@students.uniroma2.eu;

elvira.padua@unisanraffaele.gov.it; Ahammash@hamad.qa;

Mauro.lombardo@uniroma5.it; g_annino@hotmail.com

RI Alashram, Anas/AAU-4481-2021; Padua, Elvira/AAO-3237-2020; Lombardo,

Mauro/F-6133-2019

OI Alashram, Anas/0000-0002-3066-3943; Lombardo, Mauro/0000-0001-7509-5487

CR Adamovich SV, 2009, NEUROREHABILITATION, V25, P29, DOI 10.3233/NRE-2009-0497

Alashram A, 2019, AM J PHYS MED REHABI, V1, DOI [10.1097/ohm.0000000000001752., DOI 10.1097/PHM.0000000000001752]

Alashram AR, 2019, J CLIN NEUROSCI, V69, P287, DOI 10.1016/j.jocn.2019.08.080

Alashram AR, 2019, J CLIN NEUROSCI, V66, P209, DOI 10.1016/j.jocn.2019.04.026

An CM, 2018, J SPINAL CORD MED, V41, P223, DOI 10.1080/10790268.2017.1369217

Betker AL, 2007, PHYS THER, V87, P1389, DOI 10.2522/ptj.20060229

Brotherton SS, 2007, SPINAL CORD, V45, P37, DOI 10.1038/sj.sc.3101909

Chi B, 2019, ANN PHYS REHABIL MED, V62, P49, DOI 10.1016/j.rehab.2018.09.006

Deutsch JE, 2008, PHYS THER, V88, P1196, DOI 10.2522/ptj.20080062

Flynn Sheryl, 2007, J Neurol Phys Ther, V31, P180, DOI 10.1097/NPT.0b013e31815d00d5

Fulk George D, 2005, J Neurol Phys Ther, V29, P34

Ghai S, 2017, PHYS THER SPORT, V25, P65, DOI 10.1016/j.ptsp.2016.05.006

Holden MK, 2005, CYBERPSYCHOL BEHAV, V8, P187, DOI 10.1089/cpb.2005.8.187

Hsieh CL, 2002, STROKE, V33, P2626, DOI 10.1161/01.STR.0000033930.05931.93

Hu R, 2014, NEURAL REGEN RES, V9, P143, DOI 10.4103/1673-5374.125343

Casuso-Holgado MJ, 2018, CLIN REHABIL, V32, P1220, DOI 10.1177/0269215518768084

Krause JS, 2004, ARCH PHYS MED REHAB, V85, P1503, DOI 10.1016/j.apmr.2004.01.017

Maggio MG, 2019, J CLIN NEUROSCI, V61, P1, DOI 10.1016/j.jocn.2018.12.020

Maher CG, 2003, PHYS THER, V83, P713, DOI 10.1093/ptj/83.8.713

McDonald JW, 2002, LANCET, V359, P417, DOI 10.1016/S0140-6736(02)07603-1

Michielsen ME, 2011, NEUROREHAB NEURAL RE, V25, P223, DOI 10.1177/1545968310385127

Mohammadi R, 2019, J STROKE CEREBROVASC, V28, P1787, DOI 10.1016/j.jstrokecerebrovasdis.2019.03.054

Nijendijk JHB, 2014, SPINAL CORD, V52, P258, DOI 10.1038/sc.2013.180

Portney LG., 2009, FDN CLIN RES APPL PR

Rose FD, 2005, CYBERPSYCHOL BEHAV, V8, P241, DOI 10.1089/cpb.2005.8.241

Song YB, 2014, ANN REHABIL MED-ARM, V38, P160, DOI 10.5535/arm.2014.38.2.160

van Dijsseldonk RB, 2018, FRONT NEUROL, V9, DOI 10.3389/fneur.2018.00963

Villiger M, 2017, FRONT NEUROL, V8, DOI 10.3389/fneur.2017.00635

Villiger M, 2013, NEUROREHAB NEURAL RE, V27, P675, DOI 10.1177/1545968313490999

Wall T, 2015, J SPINAL CORD MED, V38, P777, DOI 10.1179/2045772314Y.0000000296

Wang B, 2019, CLIN REHABIL, V33, P1130, DOI 10.1177/0269215519843174

Zanier ER, 2018, FRONT NEUROL, V9, DOI 10.3389/fneur.2018.00345

NR 32

TC 5

Z9 7

U1 2

U2 15

PU ELSEVIER SCI LTD

PI OXFORD

PA THE BOULEVARD, LANGFORD LANE, KIDLINGTON, OXFORD OX5 1GB, OXON, ENGLAND

SN 0967-5868

EI 1532-2653

J9 J CLIN NEUROSCI

JI J. Clin. Neurosci.

PD FEB

PY 2020

VL 72

BP 322

EP 327

DI 10.1016/j.jocn.2020.01.037

PG 6

WC Clinical Neurology; Neurosciences

WE Science Citation Index Expanded (SCI-EXPANDED)

SC Neurosciences & Neurology

GA KR5NG

UT WOS:000517664500058

PM 31956089

DA 2022-06-21

ER

PT J

AU Albani, G

Pedroli, E

Cipresso, P

Bulla, D

Cimolin, V

Thomas, A

Mauro, A

Riva, G

AF Albani, Giovanni

Pedroli, Elisa

Cipresso, Pietro

Bulla, Daniel

Cimolin, Veronica

Thomas, Astrid

Mauro, Alessandro

Riva, Giuseppe

TI Visual Hallucinations as Incidental Negative Effects of Virtual Reality

on Parkinson's Disease Patients: A Link with Neurodegeneration?

SO PARKINSONS DISEASE

LA English

DT Article

ID EVOKED-POTENTIALS; DEMENTIA; RISK; PREVALENCE; MOVEMENT; BEHAVIOR;

ADULTS; SLEEP; GAIT; CUES

AB We followed up a series of 23 Parkinson's disease (PD) patients who had performed an immersive virtual reality (VR) protocol eight years before. On that occasion, six patients incidentally described visual hallucinations (VH) with occurrences of images not included in the virtual environment. Curiously, in the following years, only these patients reported the appearance of VH later in their clinical history, while the rest of the group did not. Even considering the limited sample size, we may argue that VR immersive systems can induce unpleasant effects in PD patients who are predisposed to a cognitive impairment.

C1 [Albani, Giovanni; Bulla, Daniel; Mauro, Alessandro] IRCCS Ist Auxol Italiano, Osped San Giuseppe, Div Neurol & Neurorehabil, I-28824 Oggebbio, Verbania, Italy.

[Pedroli, Elisa; Cipresso, Pietro; Riva, Giuseppe] IRCCS Ist Auxol Italiano, Appl Technol Neuropsychol Lab, I-20149 Milan, Italy.

[Cimolin, Veronica] Politecn Milan, Dept Elect Informat & Bioengn, I-20133 Milan, Italy.

[Thomas, Astrid] Univ G dAnnunzio, Dept Neurol Neuroimaging & Med Sci, I-66100 Chieti, Italy.

[Mauro, Alessandro] Univ Turin, Dept Neurosci Rita Levi Montalcini, I-10126 Turin, Italy.

[Riva, Giuseppe] Univ Cattolica Milano, Dept Psychol, I-20123 Milan, Italy.

RP Albani, G (通讯作者)，IRCCS Ist Auxol Italiano, Osped San Giuseppe, Div Neurol & Neurorehabil, Via Cadorna 90, I-28824 Oggebbio, Verbania, Italy.

EM g.albani@auxologico.it

RI Cipresso, Pietro/G-4676-2011; Pedroli, Elisa/AAC-5927-2022; Cimolin,

Veronica/AAA-3326-2021; Cimolin, Veronica/AAF-6414-2019; Riva,

Giuseppe/C-5917-2008; THOMAS, Astrid Maria/K-7727-2016; Pedroli,

Elisa/K-5751-2016

OI Cipresso, Pietro/0000-0002-0662-7678; Cimolin,

Veronica/0000-0001-6299-7254; Cimolin, Veronica/0000-0001-6299-7254;

Riva, Giuseppe/0000-0003-3657-106X; THOMAS, Astrid

Maria/0000-0003-3853-2996; Mauro, Alessandro/0000-0001-9072-7454;

Pedroli, Elisa/0000-0003-4012-262X

CR Aarsland D, 2003, ARCH NEUROL-CHICAGO, V60, P387, DOI 10.1001/archneur.60.3.387

Albani G, 2009, J NEUROL, V256, pS144

Albani G, 2009, STUD HEALTH TECHNOL, V144, P204, DOI 10.3233/978-1-60750-017-9-204

Appollonio I, 2005, NEUROL SCI, V26, P108, DOI 10.1007/s10072-005-0443-4

Arnulf I, 2000, NEUROLOGY, V55, P281, DOI 10.1212/WNL.55.2.281

Badarny S, 2014, TREMOR OTHER HYPERK, V4, DOI 10.7916/D8V69GM4

Barnes J, 2003, NEUROPSYCHOLOGIA, V41, P565, DOI 10.1016/S0028-3932(02)00182-3

Baumgartner T, 2008, FRONT HUM NEUROSCI, V2, DOI 10.3389/neuro.09.008.2008

BODISWOLLNER I, 1990, TRENDS NEUROSCI, V13, P296, DOI 10.1016/0166-2236(90)90113-O

Bronnick K, 2011, MOVEMENT DISORD, V26, P824, DOI 10.1002/mds.23525

CUMMINGS JL, 1994, NEUROLOGY, V44, P2308, DOI 10.1212/WNL.44.12.2308

Fenelon G, 2000, BRAIN, V123, P733, DOI 10.1093/brain/123.4.733

Ferrarin M, 2008, J NEUROENG REHABIL, V5, DOI 10.1186/1743-0003-5-3

Frazzitta G, 2009, MOVEMENT DISORD, V24, P1139, DOI 10.1002/mds.22491

GHILARDI MF, 1988, BRAIN, V111, P131, DOI 10.1093/brain/111.1.131

Goetz CG, 2003, MOVEMENT DISORD, V18, P738, DOI 10.1002/mds.10473

Graham G, 2011, INT J GERIATR PSYCH, V26, P263, DOI 10.1002/gps.2522

Magni E, 1996, EUR J NEUROL, V3, P198, DOI 10.1111/j.1468-1331.1996.tb00423.x

Mirelman A, 2013, BMC NEUROL, V13, DOI 10.1186/1471-2377-13-15

Mon-Williams M. A., 1993, OPHTHAL PHYSL OPT, V13, P435

Mongeon D, 2013, BRAIN COGNITION, V81, P271, DOI 10.1016/j.bandc.2012.12.001

Onofrj M, 2006, J NEUROL SCI, V248, P143, DOI 10.1016/j.jns.2006.05.025

ONOFRJ M, 1986, J NEUROL NEUROSUR PS, V49, P1150, DOI 10.1136/jnnp.49.10.1150

Park H. -S., 2011, PRACTICAL GUIDES PAN, V2011, DOI 10.1109/ICORR.2011.5975463

Postuma RB, 2012, MOVEMENT DISORD, V27, P720, DOI 10.1002/mds.24939

Ramirez-Ruiz B, 2008, MOVEMENT DISORD, V23, P2335, DOI 10.1002/mds.22258

Ravina B, 2007, MOVEMENT DISORD, V22, P1061, DOI 10.1002/mds.21382

Regan C., 1995, VIRTUAL REAL-LONDON, V1, P17, DOI [10.1007/BF02009710, DOI 10.1007/BF02009710]

Riva G, 2009, VIRTUAL REAL-LONDON, V13, P159, DOI 10.1007/s10055-009-0121-6

Rochester L, 2005, ARCH PHYS MED REHAB, V86, P999, DOI 10.1016/j.apmr.2004.10.040

Shine JM, 2011, MOVEMENT DISORD, V26, P2154, DOI 10.1002/mds.23896

Shoji Y, 2014, PLOS ONE, V9, DOI 10.1371/journal.pone.0110547

Su KJ, 2014, GAIT POSTURE, V39, P65, DOI 10.1016/j.gaitpost.2013.05.029

Zhu KD, 2014, PARKINSONISM RELAT D, V20, P980, DOI 10.1016/j.parkreldis.2014.06.006

NR 34

TC 5

Z9 6

U1 0

U2 10

PU HINDAWI LTD

PI LONDON

PA ADAM HOUSE, 3RD FLR, 1 FITZROY SQ, LONDON, W1T 5HF, ENGLAND

SN 2090-8083

EI 2042-0080

J9 PARKINSONS DIS-US

JI Parkinsons Dis.

PY 2015

VL 2015

AR 194629

DI 10.1155/2015/194629

PG 6

WC Clinical Neurology

WE Science Citation Index Expanded (SCI-EXPANDED); Social Science Citation Index (SSCI)

SC Neurosciences & Neurology

GA CJ0CZ

UT WOS:000355141800001

PM 26064775

OA Green Published, Green Submitted, gold

DA 2022-06-21

ER

PT J

AU Albani, G

Pignatti, R

Bertella, L

Priano, L

Semenza, C

Molinari, E

Riva, G

Mauro, A

AF Albani, G

Pignatti, R

Bertella, L

Priano, L

Semenza, C

Molinari, E

Riva, G

Mauro, A

TI Common daily activities in the virtual environment: a preliminary study

in parkinsonian patients

SO NEUROLOGICAL SCIENCES

LA English

DT Article; Proceedings Paper

CT 28th National Congress on Experimental Models and Clinical Correclations

in Extrapyramidal Diseases and Dementia

CY NOV 07-09, 2001

CL ALBA, ITALY

AB Patients with Parkinson's disease (PD) create behavioral motor strategies by using external cues to facilitate their movements. Virtual reality (VR) could work as an external stimulus in order to explore the motor plans by means of creation of mental images. We tested 2 women with PD aged 68 and 69 years, and 10 normal control subjects. Patients underwent a neuropsychological assessment to evaluate cognitive abilities involved in the tasks required by the VR session. VR environment reproduces common daily activities situations at home, such as eating or using the bathroom. VR describes the alterations of the motor plans in PD by a point of view different from the clinical one, by testing "pure" mental sequences of the execution of a movement, without the interference of motor disability.

C1 Ist Auxol Italiano, IRCCS, Neurol & Neurorehabil Unit, Piancavallo, VB, Italy.

Ist Auxol Italiano, IRCCS, Lab Psychol Res, Piancavallo, VB, Italy.

Univ Turin, Dept Neurosci, Turin, Italy.

Ist Auxol Italiano, IRCCS, Appl Technol Neuro Psychol Lab, Piancavallo, VB, Italy.

Univ Trieste, Dept Psychol, Trieste, Italy.

Univ Sacred Heart, Dept Psychol, I-20123 Milan, Italy.

RP Albani, G (通讯作者)，Ist Auxol Italiano, IRCCS, Neurol & Neurorehabil Unit, Piancavallo, VB, Italy.

RI Pignatti, Riccardo/A-9044-2010; Priano, Lorenzo/A-9251-2013; Riva,

Giuseppe/C-5917-2008

OI Pignatti, Riccardo/0000-0002-1200-600X; Riva,

Giuseppe/0000-0003-3657-106X; Molinari, Enrico/0000-0001-8132-694X;

Priano, Lorenzo/0000-0002-7012-6501; Mauro,

Alessandro/0000-0001-9072-7454

CR Baatile J, 2000, J REHABIL RES DEV, V37, P529

MOLINE J, 1997, VIRTUAL REALITY NEUR, P3

ROVETTA A, 1988, STUDIES HLTH TECHNOL, V50, P180

SHALLICE T, 1991, BRAIN, V114, P727, DOI 10.1093/brain/114.2.727

Stern G M, 1980, J Neural Transm Suppl, P137

WOLFSON L, 1995, J GERONTOL A-BIOL, V50, P64

NR 6

TC 24

Z9 26

U1 0

U2 11

PU SPRINGER-VERLAG

PI NEW YORK

PA 175 FIFTH AVE, NEW YORK, NY 10010 USA

SN 1590-1874

J9 NEUROL SCI

JI Neurol. Sci.

PD SEP

PY 2002

VL 23

SU 2

BP S49

EP S50

DI 10.1007/s100720200064

PG 2

WC Clinical Neurology; Neurosciences

WE Conference Proceedings Citation Index - Science (CPCI-S); Science Citation Index Expanded (SCI-EXPANDED)

SC Neurosciences & Neurology

GA 616RY

UT WOS:000179317800001

PM 12548338

DA 2022-06-21

ER

PT J

AU Albiol-Perez, S

Forcano-Garcia, M

Munoz-Tomas, MT

Manzano-Fernandez, P

Solsona-Hernandez, S

Mashat, MA

Gil-Gomez, JA

AF Albiol-Perez, S.

Forcano-Garcia, M.

Munoz-Tomas, M. T.

Manzano-Fernandez, P.

Solsona-Hernandez, S.

Mashat, M. A.

Gil-Gomez, J. A.

TI A Novel Virtual Motor Rehabilitation System for Guillain-Barre Syndrome

Two Single Case Studies

SO METHODS OF INFORMATION IN MEDICINE

LA English

DT Article

DE Guillain-Barre; postural control; balance disorders; virtual motor

rehabilitation; virtual reality; balance rehabilitation

ID AXONAL NEUROPATHY; TECHNOLOGIES; BALANCE; VARIANT

AB Introduction: This article is part of the Focus Theme of Methods of Information in Medicine on "New Methodologies for Patients Rehabilitation".

Objectives: For Guillain-Barre patients, motor rehabilitation programs are helpful at the onset to prevent the complications of paralysis and in cases of persistent motor impairment. Traditional motor rehabilitation programs may be tedious and monotonous, resulting in low adherence to the treatments. A Virtual Motor Rehabilitation system has been tested in Guillain-Barre patients to increase patient adherence and to improve clinical results.

Methods: Two people with Guillain-Barre performed 20 rehabilitation sessions. We tested a novel system based on Motor Virtual Rehabilitation in three periods of time (baseline evaluation, final evaluation, and follow-up. In the training program, the participants carried out a specific treatment using the Active Balance Rehabilitation system (ABAR). The system is composed of customizable virtual games to perform static and dynamic balance rehabilitation.

Results: Significant improvements in clinical results were obtained by both participants, with significant results in the static balance clinical test of the Anterior Reach test in the standing position and unipedal stance time. Other significant results were found in dynamic balance clinical tests in the Berg Balance Scale test and the 30-second Sit-to-Stand test. With regard to acceptance of the system, both patients enjoyed the experience, and both patients thought that this system was helpful for their rehabilitation.

Conclusions: The results show that Virtual Motor Rehabilitation for Guillain-Barre patients provides clinical improvements in an entertaining way.

C1 [Albiol-Perez, S.] Univ Zaragoza, Dept Informat & Ingn Sistemas, Teruel 44003, Spain.

[Albiol-Perez, S.] Univ Fuerzas Armadas ESPE, Dept Elect & Elect, Sangolqui, Ecuador.

[Albiol-Perez, S.] Prometeo Project Res SENESCYT, Quito, Ecuador.

[Forcano-Garcia, M.; Munoz-Tomas, M. T.; Manzano-Fernandez, P.; Solsona-Hernandez, S.] Hosp S Jose, Teruel, Spain.

[Mashat, M. A.] King Abdulaziz Univ, Fac Med, Med Educ Dept, Jeddah 21413, Saudi Arabia.

[Gil-Gomez, J. A.] Univ Politecn Valencia, Inst Univ Automat & Informat Ind, E-46022 Valencia, Spain.

RP Albiol-Perez, S (通讯作者)，Univ Zaragoza, Dept Informat & Ingn Sistemas, Ciudad Escolar S-N, Teruel 44003, Spain.

EM salbiol@unizar.es

RI Gil-Gómez, José-Antonio/H-9756-2015

OI Gil-Gómez, José-Antonio/0000-0001-9954-2480; Albiol Perez,

Sergio/0000-0002-6280-1474

FU Fundacion Antonio Gargallo ("Ayudas financiadas por la Obra Social de

Ibercaja de proyectos de investigacion") [2013/B001]

FX The authors would like to thank all of the clinical specialists and

patients of San Jose Hospital for participating in the present study. We

specially thank Carmen Aula-Valero for her time in the training program,

and Jose-Antonio Lozano-Quilis and Hermenegildo Gil-Gomez for their

suggestions in the design of the ABAR system. This contribution was

partially funded by the Fundacion Antonio Gargallo ("Ayudas financiadas

por la Obra Social de Ibercaja de proyectos de investigacion 2013",

proyecto 2013/B001).

CR Albiol-Perez S, 2013, IEEE J BIOMEDICAL HL, V99

Albiol-Perez S, 2013, INT CONF PER COMP, P370, DOI 10.4108/icst.pervasivehealth.2013.252190

ASBURY AK, 1990, ANN NEUROL, V27, pS21, DOI 10.1002/ana.410270707

Austin GP, 2002, PERCEPT MOTOR SKILL, V95, P733, DOI 10.2466/PMS.95.7.733-740

BERG KO, 1992, CAN J PUBLIC HEALTH, V83, pS7

BOHANNON RW, 1984, PHYS THER, V64, P1067, DOI 10.1093/ptj/64.7.1067

BRISCOE DM, 1987, ARCH DIS CHILD, V62, P733, DOI 10.1136/adc.62.7.733

Burns TM, 2008, SEMIN NEUROL, V28, P152, DOI 10.1055/s-2008-1062261

CHARLSON ME, 1987, J CHRON DIS, V40, P373, DOI 10.1016/0021-9681(87)90171-8

Chen An-Chih, 2013, Acta Neurol Taiwan, V22, P26

Cheng J, 2011, J NEUROSURG-SPINE, V15, P605, DOI 10.3171/2011.8.SPINE1159

Collin C, 1988, Int Disabil Stud, V10, P61

Dimachkie MM, 2013, NEUROL CLIN, V31, P491, DOI 10.1016/j.ncl.2013.01.005

Drenthen J, 2013, NEUROLOGY

Drenthen J, 2013, J PERIPHER NERV SYST, V18, P25, DOI 10.1111/jns5.12003

DUNCAN PW, 1990, J GERONTOL, V45, pM192, DOI 10.1093/geronj/45.6.M192

El Mhandi L, 2007, AM J PHYS MED REHAB, V86, P716, DOI 10.1097/PHM.0b013e31813e0b67

FOLSTEIN MF, 1975, J PSYCHIAT RES, V12, P189, DOI 10.1016/0022-3956(75)90026-6

Forcano Garcia M, 2013, EUROPEAN GERIATRIC M, V4, pS109, DOI DOI 10.1016/J.EURGER.2013.07.356

Fujii D, 2011, CASE REP NEUROL, V4, P212, DOI 10.1159/000345847

Garssen MPJ, 2004, NEUROLOGY, V63, P2393, DOI 10.1212/01.WNL.0000148589.87107.9C

Gietzelt M, 2014, METHOD INFORM MED, V53, P160, DOI 10.3414/ME13-02-0008

Gil-Gomez JA, 2011, J NEUROENG REHABIL, V8, DOI 10.1186/1743-0003-8-30

Holmes J. D., 2012, CLIN REHABIL

Hughes RAC, 2005, ARCH NEUROL-CHICAGO, V62, P1194, DOI 10.1001/archneur.62.8.1194

Islam Z, 2010, NEUROLOGY, V74, P581, DOI 10.1212/WNL.0b013e3181cff735

Jones C, 2002, INT J ACTIVE AGING, P25

Khan F, 2012, EUR J PHYS REHAB MED, V48, P507

Khan F, 2010, COCHRANE DB SYST REV, DOI 10.1002/14651858.CD008505.pub2

Koch S, 2009, METHOD INFORM MED, V48, P29, DOI 10.3414/ME9136

Kuwabara S, 1999, J NEUROL NEUROSUR PS, V67, P180, DOI 10.1136/jnnp.67.2.180

Kuwabara S, 2013, LANCET NEUROL, V12, P1180, DOI 10.1016/S1474-4422(13)70215-1

Lange B, 2012, DISABIL REHABIL, V34, P1863, DOI 10.3109/09638288.2012.670029

LAWTON MP, 1975, J GERONTOL, V30, P85, DOI 10.1093/geronj/30.1.85

McGrogan A, 2009, NEUROEPIDEMIOLOGY, V32, P150, DOI 10.1159/000184748

Misraa I, 2011, CASE REP NEUROL, V4, P137, DOI 10.1159/000342448

Munoz Tomas M, 2013, EUR GERIATR MED, V4, pS109, DOI [10.1016/j.eurger.2013.07.358, DOI 10.1016/J.EURGER.2013.07.358]

Odaka M, 2001, J NEUROL NEUROSUR PS, V70, P50, DOI 10.1136/jnnp.70.1.50

Oh SJ, 2003, NEUROLOGY, V61, P1507, DOI 10.1212/01.WNL.0000096166.28131.4C

Orlikowski D, 2006, INTENS CARE MED, V32, P1962, DOI 10.1007/s00134-006-0332-1

Orsini M, 2010, REV NEUROSCIENCIAS, V18, P572

Pfeiffer G, 1999, NERVENARZT, V70, P136, DOI 10.1007/s001150050414

PODSIADLO D, 1991, J AM GERIATR SOC, V39, P142, DOI 10.1111/j.1532-5415.1991.tb01616.x

RICHARDS CL, 1995, GAIT ANAL THEORY APP, P355

ROPPER AH, 1992, NEW ENGL J MED, V326, P1130, DOI 10.1056/NEJM199204233261706

Rostasy KM, 2005, NEUROPEDIATRICS, V36, P260, DOI 10.1055/s-2005-865774

Sankhyan N, 2013, J CLIN NEUROSCI

Sejvar JJ, 2011, NEUROEPIDEMIOLOGY, V36, P123, DOI 10.1159/000324710

Simonov M, 2014, METHODS INF MED, V53

Takazawa T, 2012, INTERNAL MED, V51, P2433, DOI 10.2169/internalmedicine.51.7737

TINETTI ME, 1986, AM J MED, V80, P429, DOI 10.1016/0002-9343(86)90717-5

Tuckey Jo, 2004, Physiother Res Int, V9, P96, DOI 10.1002/pri.306

Verma R., 2012, BMJ CASE REP

Verschuren O, 2008, DISABIL REHABIL, V30, P1358, DOI 10.1080/09638280701639873

NR 54

TC 12

Z9 12

U1 2

U2 11

PU GEORG THIEME VERLAG KG

PI STUTTGART

PA RUDIGERSTR 14, D-70469 STUTTGART, GERMANY

SN 0026-1270

EI 2511-705X

J9 METHOD INFORM MED

JI Methods Inf. Med.

PY 2015

VL 54

IS 2

BP 127

EP 134

DI 10.3414/ME14-02-0002

PG 8

WC Computer Science, Information Systems; Health Care Sciences & Services;

Medical Informatics

WE Science Citation Index Expanded (SCI-EXPANDED)

SC Computer Science; Health Care Sciences & Services; Medical Informatics

GA CE4MF

UT WOS:000351804000004

PM 25609504

DA 2022-06-21

ER

PT J

AU Albiol-Perez, S

Gil-Gomez, JA

Munoz-Tomas, MT

Gil-Gomez, H

Vial-Escolano, R

Lozano-Quilis, JA

AF Albiol-Perez, Sergio

Gil-Gomez, Jose-Antonio

Munoz-Tomas, Maria-Teresa

Gil-Gomez, Hermenegildo

Vial-Escolano, Raquel

Lozano-Quilis, Jose-Antonio

TI The Effect of Balance Training on Postural Control in Patients with

Parkinson's Disease Using a Virtual Rehabilitation System

SO METHODS OF INFORMATION IN MEDICINE

LA English

DT Article

DE Parkinson's disease; spatial postural control; motor symptoms; virtual

motor rehabilitation; virtual reality

ID MOVEMENT-DISORDER SOCIETY; MOTOR REHABILITATION; NONMOTOR SYMPTOMS;

TREMOR; PREVALENCE; REALITY; BOARD; PATHOPHYSIOLOGY; BRADYKINESIA;

EPIDEMIOLOGY

AB Objectives: Parkinson's disease (PD) is a progressive neurodegenerative disorder characterized by motor clinical alterations among others. Postural problems have serious consequences for patients, not only limiting their daily life but also increasing some risks, like the risk of fall. Inadequate postural control and postural instability is a major problem in PD patients. A Virtual Motor Rehabilitation System (VMR) has been tested in patients with PD in the intervention period. Our pur-pose was to analyze the evolution of the spatial postural control during the intervention period, to see if there are any changes caused precisely by this intervention.

Methods: Ten people with PD carried out 15 virtual rehabilitation sessions. We tested a groundbreaking system based on Virtual Motor Rehabilitation in two periods of time (baseline evaluation and final evaluation). In the training sessions, the participants performed a customizable treatment using a low-cost system, the Active Balance Rehabilitation system (ABAR). We stored the pressure performed by the participants every five hundredths of a second, and we analyzed the patients' pressure when they maintained their body on the left, on the right, and in the center in sitting position. Our system was able to measure postural control in every patient in each of the virtual rehabilitation sessions.

Results: There are no significant differences in the performance of postural control in any of the positions evaluated throughout the sessions. Moreover, the results show a trend to an improvement in all positions. This improvement is especially remarkable in the left/right positions, which are the most important positions in order to avoid problems such as the risk of fall. With regard to the suitability of the ABAR system, we have found outstanding results in enjoyment, success, clarity, and helpfulness.

Conclusions: Although PD is a progressive neurodegenerative disorder, the results demonstrate that patients with PD maintain or even improve their postural control in all positions. We think that the main factor influencing these results is that patients use more of their available cognitive processing to improve their postural control. The ABAR system allows us to make this assumption because the system requires the continuous attention of patients, promoting cognitive processing.

C1 [Albiol-Perez, Sergio; Munoz-Tomas, Maria-Teresa; Vial-Escolano, Raquel] Univ Zaragoza, Aragon Hlth Res Inst IIS Aragon, C-Atarazana 4, Teruel 44003, Spain.

[Gil-Gomez, Jose-Antonio; Gil-Gomez, Hermenegildo; Lozano-Quilis, Jose-Antonio] Univ Politecn Valencia, Inst Univ Automat & Informat Ind, Valencia, Spain.

[Munoz-Tomas, Maria-Teresa; Vial-Escolano, Raquel] Hosp S Jose, Teruel, Spain.

RP Albiol-Perez, S (通讯作者)，Univ Zaragoza, Aragon Hlth Res Inst IIS Aragon, C-Atarazana 4, Teruel 44003, Spain.

EM salbiol@unizar.es

RI Lozano-Quilis, José-Antonio/K-6630-2017; Gil-Gómez,

José-Antonio/H-9756-2015; Gil-Gómez, Hermenegildo/AAJ-8287-2020

OI Gil-Gómez, José-Antonio/0000-0001-9954-2480; Gil-Gomez,

hermenegildo/0000-0002-7985-2454; Munoz Tomas/0000-0002-3480-3878

FU Gobierno de Aragon, Departamento de Industrie e Innovacion, y Fondo

Social Europeo "Construyendo Europa desde Aragon"; Programa Ibercaja-CAI

de Estancias de Investigacion

FX This contribution was partially funded by the Gobierno de Aragon,

Departamento de Industrie e Innovacion, y Fondo Social Europeo

"Construyendo Europa desde Aragon" and by the Programa Ibercaja-CAI de

Estancias de Investigacion.

CR Albiol-Perez S, 2015, METHOD INFORM MED, V54, P127, DOI 10.3414/ME14-02-0002

Albiol-Perez S., 2012, 9 INT C DIS VIRT REA

Albiol-Perez S, 2014, IEEE J BIOMED HEALTH, V18, P391, DOI 10.1109/JBHI.2013.2272101

Badarny S, 2014, TREMOR OTHER HYPERK, V4, DOI 10.7916/D8V69GM4

Bachlin M, 2010, METHOD INFORM MED, V49, P88, DOI 10.3414/ME09-02-0003

Berardelli A, 2001, BRAIN, V124, P2131, DOI 10.1093/brain/124.11.2131

Bronnick K, 2006, J NEUROL NEUROSUR PS, V77, P1136, DOI 10.1136/jnnp.2006.093146

Budzianowska A, 2008, NEUROL NEUROCHIR POL, V42, P12

Camara C, 2015, BIOMED SIGNAL PROCES, V16, P88, DOI 10.1016/j.bspc.2014.09.006

CHARLSON ME, 1987, J CHRON DIS, V40, P373, DOI 10.1016/0021-9681(87)90171-8

Chaudhuri KR, 2011, PARKINSONISM RELAT D, V17, P717, DOI 10.1016/j.parkreldis.2011.02.018

Collin C, 1988, Int Disabil Stud, V10, P61

Crawford P, 2011, AM FAM PHYSICIAN, V83, P697

Dai HD, 2015, SENSORS-BASEL, V15, P25055, DOI 10.3390/s151025055

de Lau LML, 2006, LANCET NEUROL, V5, P525, DOI 10.1016/S1474-4422(06)70471-9

Deane K., 2001, COCHRANE DB SYST REV, V3, DOI DOI 10.1002/14651858

deRijk MC, 1997, J NEUROL NEUROSUR PS, V62, P10, DOI 10.1136/jnnp.62.1.10

Deuschl G, 1998, MOVEMENT DISORD, V13, P2, DOI 10.1002/mds.870131303

Dibble LE, 2006, MOVEMENT DISORD, V21, P1444, DOI 10.1002/mds.20997

Dibble LE, 2009, PARKINSONISM RELAT D, V15, P752, DOI 10.1016/j.parkreldis.2009.04.009

Dorsey ER, 2007, NEUROLOGY, V68, P384, DOI 10.1212/01.wnl.0000247740.47667.03

Mendes FAD, 2012, PHYSIOTHERAPY, V98, P217, DOI 10.1016/j.physio.2012.06.001

Duval C, 2006, BRAIN RES BULL, V70, P44, DOI 10.1016/j.brainresbull.2005.11.010

Fahn S, 2003, ANN NY ACAD SCI, V991, P1, DOI 10.1111/j.1749-6632.2003.tb07458.x

FINDLEY LJ, 1981, J NEUROL NEUROSUR PS, V44, P534, DOI 10.1136/jnnp.44.6.534

FOLSTEIN MF, 1975, J PSYCHIAT RES, V12, P189, DOI 10.1016/0022-3956(75)90026-6

Gil-Gomez JA, 2013, INT CONF PER COMP, P335, DOI 10.4108/icst.pervasivehealth.2013.252216

Gil-Gomez JA, 2011, J NEUROENG REHABIL, V8, DOI 10.1186/1743-0003-8-30

Goetz CG, 2003, MOVEMENT DISORD, V18, P738, DOI 10.1002/mds.10473

Goetz CG, 2008, MOVEMENT DISORD, V23, P2129, DOI 10.1002/mds.22340

Herz NB, 2013, PARKINSONISM RELAT D, V19, P1039, DOI 10.1016/j.parkreldis.2013.07.014

Holmes JD, 2013, CLIN REHABIL, V27, P361, DOI 10.1177/0269215512458684

Horak FB, 2006, AGE AGEING, V35, P7, DOI 10.1093/ageing/afl077

Jankovic J, 2008, J NEUROL NEUROSUR PS, V79, P368, DOI 10.1136/jnnp.2007.131045

Kashihara K, 2006, J NEUROL, V253, P38, DOI 10.1007/s00415-006-7009-0

Kim SD, 2013, CNS DRUGS, V27, P97, DOI 10.1007/s40263-012-0012-3

King LA, 2014, ARCH PHYS MED REHAB, V95, P2152, DOI 10.1016/j.apmr.2014.07.396

Kizony R, 2006, 11 ANN CYB THER C VI

LAWTON MP, 1975, J GERONTOL, V30, P85, DOI 10.1093/geronj/30.1.85

Lozano-Quilis JA, 2014, JMIR SERIOUS GAMES, V2, P43, DOI 10.2196/games.2933

Martens KAE, 2015, EXP BRAIN RES, V233, P787, DOI 10.1007/s00221-014-4154-z

Massano J, 2012, CSH PERSPECT MED, V2, DOI 10.1101/cshperspect.a008870

McIntosh GC, 1997, J NEUROL NEUROSUR PS, V62, P22, DOI 10.1136/jnnp.62.1.22

Mhatre PV, 2013, PM&R, V5, P769, DOI 10.1016/j.pmrj.2013.05.019

Micieli G, 2003, NEUROL SCI, V24, pS32, DOI 10.1007/s100720300035

Muangpaisan W, 2009, J EPIDEMIOL, V19, P281, DOI 10.2188/jea.JE20081034

Munoz Tomas M, 2013, EUR GERIATR MED, V4, pS109, DOI [10.1016/j.eurger.2013.07.358, DOI 10.1016/J.EURGER.2013.07.358]

Nolano M, 2008, BRAIN, V131, P1903, DOI 10.1093/brain/awn102

Nombela Cristina, 2011, Front Neurol, V2, P82, DOI 10.3389/fneur.2011.00082

Nussbaum RL, 2003, NEW ENGL J MED, V348, P1356, DOI 10.1056/NEJM2003ra020003

Ondo WG, 2001, NEUROLOGY, V57, P1392, DOI 10.1212/WNL.57.8.1392

Poletti M, 2012, J NEUROL SCI, V317, P97, DOI 10.1016/j.jns.2012.02.022

Pompeu JE, 2014, PHYSIOTHERAPY, V100, P162, DOI 10.1016/j.physio.2013.10.003

Pompeu JE, 2012, PHYSIOTHERAPY, V98, P196, DOI 10.1016/j.physio.2012.06.004

Pringsheim T, 2014, MOVEMENT DISORD, V29, P1583, DOI 10.1002/mds.25945

Rigas G, 2012, IEEE T INF TECHNOL B, V16, P478, DOI 10.1109/TITB.2011.2182616

Robbins TW, 2014, MOVEMENT DISORD, V29, P597, DOI 10.1002/mds.25853

Salarian A, 2007, IEEE T BIO-MED ENG, V54, P313, DOI 10.1109/TBME.2006.886670

Salat-Foix D, 2012, EXPERT REV NEUROTHER, V12, P239, DOI [10.1586/ern.11.192, 10.1586/ERN.11.192]

Saposnik G, 2011, STROKE, V42, P1380, DOI 10.1161/STROKEAHA.110.605451

Schwartze M, 2016, FRONT NEUROL, V6, DOI 10.3389/fneur.2015.00270

SHAPIRO SS, 1965, BIOMETRIKA, V52, P591, DOI 10.2307/2333709

Shulman LM, 2001, MOVEMENT DISORD, V16, P507, DOI 10.1002/mds.1099

Summa S., 2013, GAIT POSTURE S1, V37, P235, DOI [10.1016/j.gaitpost.2012.12.040, DOI 10.1016/J.GAITP0ST.2012.12.040]

von Campenhausen S, 2005, EUR NEUROPSYCHOPHARM, V15, P473, DOI 10.1016/j.euroneuro.2005.04.007

Weintraub D, 2004, J AM GERIATR SOC, V52, P784, DOI 10.1111/j.1532-5415.2004.52219.x

*WORLD MED ASS ETH, DECL HELS

NR 67

TC 20

Z9 23

U1 2

U2 17

PU GEORG THIEME VERLAG KG

PI STUTTGART

PA RUDIGERSTR 14, D-70469 STUTTGART, GERMANY

SN 0026-1270

EI 2511-705X

J9 METHOD INFORM MED

JI Methods Inf. Med.

PY 2017

VL 56

IS 2

BP 138

EP 144

DI 10.3414/ME16-02-0004

PG 7

WC Computer Science, Information Systems; Health Care Sciences & Services;

Medical Informatics

WE Science Citation Index Expanded (SCI-EXPANDED)

SC Computer Science; Health Care Sciences & Services; Medical Informatics

GA EP7IY

UT WOS:000397552700007

PM 28244545

OA Green Accepted, Green Published

DA 2022-06-21

ER

PT J

AU Albiol-Perez, S

Gil-Gomez, JA

Llorens, R

Alcaniz, M

Font, CC

AF Albiol-Perez, Sergio

Gil-Gomez, Jose-Antonio

Llorens, Roberto

Alcaniz, Mariano

Colomer Font, Carolina

TI The Role of Virtual Motor Rehabilitation: A Quantitative Analysis

Between Acute and Chronic Patients With Acquired Brain Injury

SO IEEE JOURNAL OF BIOMEDICAL AND HEALTH INFORMATICS

LA English

DT Article

DE Acute/chronic acquired brain injury; balance rehabilitation; virtual

motor rehabilitation; virtual reality

ID WII GAMING TECHNOLOGY; CHRONIC STROKE; DEVELOPMENTAL-DISABILITIES;

FUNCTIONAL RECOVERY; PHYSICAL-ACTIVITIES; ENABLING PEOPLE; BALANCE

BOARDS; REALITY; COMMUNITY; CHILDREN

AB Acquired brain injury (ABI) is one of the main problems of disability and death in the world. Its incidence and survival rate are increasing annually. Thus, the number of chronic ABI patients is gradually growing. Traditionally, rehabilitation programs are applied to postacute and acute patients, but recent publications determine that chronic patients may benefit from rehabilitation. Also, in the last few years, the potential of virtual rehabilitation (VR) systems has been demonstrated. However, until now, no previous studies have been carried out to compare the evolution of chronic patients with acute patients in a VR program. To perform this study, we developed a VR system for ABI patients. The system, vestibular virtual rehabilitation (V2R), was designed with clinical specialists. V2R has been tested with 21 people ranging in age from 18 to 80 years old that were classified in two groups: chronic patients and acute patients. The results demonstrate a similar recovery for chronic and acute patients during the intervention period. Also, the results showed that chronic patients stop their improvement when they finish their training. This conclusion encourages us to direct our developments toward VR systems that can be easily integrated at home, allowing chronic patients to have a permanent VR training program.

C1 [Albiol-Perez, Sergio] Univ Zaragoza, Dept Informat & Ingn Sistemas, E-50009 Zaragoza, Spain.

[Gil-Gomez, Jose-Antonio] Univ Politecn Valencia, Inst Univ Automat & Informat Ind, Valencia 46022, Spain.

[Llorens, Roberto; Alcaniz, Mariano] Univ Politecn Valencia, Inst Interuniv Invest Bioingn & Tecnol Orientada, Valencia 46022, Spain.

[Colomer Font, Carolina] Hosp NISA, Serv Neurorrehabil, Valencia 146011, Spain.

RP Albiol-Perez, S (通讯作者)，Univ Zaragoza, Dept Informat & Ingn Sistemas, E-50009 Zaragoza, Spain.

EM salbiol@unizar.es; jgil@upv.es; rllorens@labhuman.i3bh.es;

malcaniz@labhuman.i3bh.es; carol@neurorhb.com

RI Lloréns, Roberto/O-8978-2014; Gil-Gómez, José-Antonio/H-9756-2015;

Alcaniz, Mariano L./I-9659-2016; Llorens, Roberto/AAL-2604-2021;

Alcañiz, Mariano/CAG-6569-2022

OI Lloréns, Roberto/0000-0002-8677-8707; Gil-Gómez,

José-Antonio/0000-0001-9954-2480; Alcaniz, Mariano

L./0000-0001-9207-0636; Llorens, Roberto/0000-0002-8677-8707; Alcañiz,

Mariano/0000-0001-9207-0636; Albiol Perez, Sergio/0000-0002-6280-1474

FU Ministerio de Educacion y Ciencia Spain [SEJ2006-14301/PSIC]; Excellence

Research Program PROMETEO (Generalitat Valenciana. Conselleria de

Educacion) [2008-157]

FX This work was supported by the Ministerio de Educacion y Ciencia Spain:

Projects Consolider-C (SEJ2006-14301/PSIC), "CIBER of Physiopathology of

Obesity and Nutrition, an initiative of ISCIII," and the Excellence

Research Program PROMETEO (Generalitat Valenciana. Conselleria de

Educacion, 2008-157).

CR Babikian T, 2009, NEUROPSYCHOLOGY, V23, P283, DOI 10.1037/a0015268

BERG KO, 1992, CAN J PUBLIC HEALTH, V83, pS7

Brooke J., 1996, SUS A QUICK DIRTY US

Cameirao MS, 2012, STROKE, V43, P2720, DOI 10.1161/STROKEAHA.112.653196

Cameirao MS, 2010, J NEUROENG REHABIL, V7, DOI 10.1186/1743-0003-7-48

Cho KH, 2012, TOHOKU J EXP MED, V228, P69, DOI 10.1620/tjem.228.69

DUNCAN PW, 1990, J GERONTOL, V45, pM192, DOI 10.1093/geronj/45.6.M192

Eley KA, 2010, NEW ENGL J MED, V362, P473, DOI 10.1056/NEJMc0909544

Faul M., 2010, TRAUMATIC BRAIN INJU, P7

FOLSTEIN MF, 1975, J PSYCHIAT RES, V12, P189, DOI 10.1016/0022-3956(75)90026-6

Geurtsen GJ, 2012, ARCH PHYS MED REHAB, V93, P908, DOI 10.1016/j.apmr.2011.12.008

Gil-Gomez JA, 2011, J NEUROENG REHABIL, V8, DOI 10.1186/1743-0003-8-30

Gupta A, 2012, ANN INDIAN ACAD NEUR, V15, P120, DOI 10.4103/0972-2327.94995

Hill KD, 1996, PHYSIOTHER CAN, V48, P257, DOI DOI 10.3138/PTC.48.4.257

Hoofien D, 2001, BRAIN INJURY, V15, P189

Hyndman D, 2003, DISABIL REHABIL, V25, P817, DOI 10.1080/0963828031000122221

Ju YH, 2012, ARCH PHYS MED REHAB, V93, P471, DOI 10.1016/j.apmr.2011.10.004

Kalron A, 2011, EUR J PHYS REHAB MED, V47, P579

Kim JH, 2009, AM J PHYS MED REHAB, V88, P693, DOI 10.1097/PHM.0b013e3181b33350

Lange B, 2012, DISABIL REHABIL, V34, P1863, DOI 10.3109/09638288.2012.670029

Merians AS, 2006, NEUROREHAB NEURAL RE, V20, P252, DOI 10.1177/1545968306286914

Nakase-Thompson R, 2005, BRAIN INJURY, V19, P685, DOI 10.1080/02699050400025331

Nardone A, 2010, ARCH PHYS MED REHAB, V91, P1869, DOI 10.1016/j.apmr.2010.09.011

PODSIADLO D, 1991, J AM GERIATR SOC, V39, P142, DOI 10.1111/j.1532-5415.1991.tb01616.x

Robinson RJ, 2008, EMERG RADIOL, V15, P255, DOI 10.1007/s10140-008-0712-7

Saposnik G, 2011, STROKE, V42, P1380, DOI 10.1161/STROKEAHA.110.605451

Saposnik G, 2010, STROKE, V41, P1477, DOI 10.1161/STROKEAHA.110.584979

Shih CH, 2012, RES DEV DISABIL, V33, P983, DOI 10.1016/j.ridd.2011.12.018

Shih CH, 2012, RES DEV DISABIL, V33, P39, DOI 10.1016/j.ridd.2011.08.006

Shih CH, 2011, RES DEV DISABIL, V32, P2780, DOI 10.1016/j.ridd.2011.05.031

Shih CH, 2011, RES DEV DISABIL, V32, P699, DOI 10.1016/j.ridd.2010.11.011

Shih CH, 2010, RES DEV DISABIL, V31, P936, DOI 10.1016/j.ridd.2010.03.004

Shih CH, 2010, RES DEV DISABIL, V31, P281, DOI 10.1016/j.ridd.2009.09.013

Shiroma Eric J, 2010, J Correct Health Care, V16, P147, DOI 10.1177/1078345809356538

Tagliaferri F, 2006, ACTA NEUROCHIR, V148, P255, DOI 10.1007/s00701-005-0651-y

Teasell R, 2002, ARCH PHYS MED REHAB, V83, P329, DOI 10.1053/apmr.2002.29623

Ustinova KI, 2011, J NEUROENG REHABIL, V8, DOI 10.1186/1743-0003-8-61

Verschuren O., 2007, ARCH PHYS MED REHAB, V88, P32

World Health Organization, 2006, NEUR DIS PUBL HLTH C, P164

Wuang YP, 2011, RES DEV DISABIL, V32, P312, DOI 10.1016/j.ridd.2010.10.002

You SH, 2005, STROKE, V36, P1166, DOI 10.1161/01.STR.0000162715.43417.91

NR 41

TC 13

Z9 13

U1 0

U2 19

PU IEEE-INST ELECTRICAL ELECTRONICS ENGINEERS INC

PI PISCATAWAY

PA 445 HOES LANE, PISCATAWAY, NJ 08855-4141 USA

SN 2168-2194

J9 IEEE J BIOMED HEALTH

JI IEEE J. Biomed. Health Inform.

PD JAN

PY 2014

VL 18

IS 1

BP 391

EP 398

DI 10.1109/JBHI.2013.2272101

PG 8

WC Computer Science, Information Systems; Computer Science,

Interdisciplinary Applications; Mathematical & Computational Biology;

Medical Informatics

WE Science Citation Index Expanded (SCI-EXPANDED); Social Science Citation Index (SSCI)

SC Computer Science; Mathematical & Computational Biology; Medical

Informatics

GA 287CM

UT WOS:000329516400045

PM 24403439

OA Green Published

DA 2022-06-21

ER

PT J

AU Aldridge, A

Bethel, CL

AF Aldridge, Audrey

Bethel, Cindy L.

TI A Systematic Review of the Use of Art in Virtual Reality

SO ELECTRONICS

LA English

DT Review

DE virtual reality; art therapy; rehabilitation; neurorehabilitation;

neuroplasticity; brain injury

ID CHRONIC STROKE PATIENTS; TRAUMATIC BRAIN-INJURY; MENTAL PRACTICE; MOTOR

IMAGERY; UPPER-LIMB; REHABILITATION; THERAPY; TECHNOLOGY; POSTSTROKE;

SKILLS

AB Brain injuries can create life-altering challenges and have the potential to leave people with permanent disabilities. Art therapy is a popular method used for treating many of the disabilities that can accompany a brain injury. In a systematic review, an assessment of how art is being used in virtual reality (VR) was conducted, and the feasibility of brain injury patients to participate in virtual art therapy was investigated. Studies included in this review highlight the importance of artistic subject matter, sensory stimulation, and measurable performance outcomes for assessing the effect art therapy has on motor impairment in VR. Although there are limitations to using art therapy in a virtual environment, studies show that it can feasibly be used in virtual reality for neurorehabilitation purposes.

C1 [Aldridge, Audrey; Bethel, Cindy L.] Mississippi State Univ, Dept Comp Sci & Engn, Starkville, MS 39762 USA.

RP Aldridge, A (通讯作者)，Mississippi State Univ, Dept Comp Sci & Engn, Starkville, MS 39762 USA.

EM ala214@msstate.edu; cbethel@cse.msstate.edu

OI Aldridge, Audrey/0000-0003-3733-4736; Bethel, Cindy

L./0000-0001-9036-3275

CR Adamovich SV, 2004, P ANN INT IEEE EMBS, V26, P4936

Agnihotri S, 2014, DEV NEUROREHABIL, V17, P44, DOI 10.3109/17518423.2013.844739

Alex M, 2021, INT J HUM-COMPUT ST, V145, DOI 10.1016/j.ijhcs.2020.102481

[Anonymous], 2018, TILT BRUSH GOOGLE

[Anonymous], The Daily Californian

Bermdez i Badia S., 2016, NEUROREHABILITATION, P573, DOI [10.1007/978-3-319-28603-7_28, DOI 10.1007/978-3-319-28603-7_28, 10.1007/978-3- 319-28603-7 28, DOI 10.1007/978-3-319-28603-728]

Bolwerk A, 2014, PLOS ONE, V9, DOI 10.1371/journal.pone.0101035

Brain Injury Association of America, BRAIN INJ FACTS STAT

Broeren J, 2004, ARCH PHYS MED REHAB, V85, P1247, DOI 10.1016/j.apmr.2003.09.020

Chan EHW, 2005, PROCEEDINGS OF CRIOCM 2005 INTERNATIONAL RESEARCH SYMPOSIUM ON ADVANCEMENT OF CONSTRUCTION MANAGEMENT AND REAL ESTATE, P56

Chegg, DIAGRAM PREMOTOR COR

Crosbie JH, 2004, CLIN REHABIL, V18, P60, DOI 10.1191/0269215504cr702oa

Csikszentmihalyi M., 1992, OPTIMAL EXPERIENCE P, V1st ed., P1

Cucca A, 2018, COMPLEMENT THER MED, V40, P70, DOI 10.1016/j.ctim.2018.07.011

Dickstein R, 2004, PHYS THER, V84, P1167, DOI 10.1093/ptj/84.12.1167

Dijkerman HC, 2004, CLIN REHABIL, V18, P538, DOI 10.1191/0269215504cr769oa

FUGLMEYER AR, 1975, SCAND J REHABIL MED, V7, P13

Hacmun I, 2021, ART PSYCHOTHER, V72, DOI 10.1016/j.aip.2020.101745

Holden M.K., 2002, NEUROL REP, V26, P62, DOI DOI 10.1097/01253086-200226020-00003

Hoshi E, 2007, CURR OPIN NEUROBIOL, V17, P234, DOI 10.1016/j.conb.2007.02.003

Iosa M, 2021, FRONT PSYCHOL, V11, DOI 10.3389/fpsyg.2020.611956

Jackson PL, 2004, NEUROREHAB NEURAL RE, V18, P106, DOI 10.1177/0888439004265249

James SL, 2019, LANCET NEUROL, V18, P56, DOI 10.1016/S1474-4422(18)30415-0

Jones JP, 2019, ART PSYCHOTHER, V63, P18, DOI 10.1016/j.aip.2019.04.004

Jung RE, 2010, HUM BRAIN MAPP, V31, P398, DOI 10.1002/hbm.20874

Jung SH, 2005, ARCH PHYS MED REHAB, V86, P2218, DOI 10.1016/j.apmr.2005.04.015

Kaimal G, 2020, ART THER, V37, P16, DOI 10.1080/07421656.2019.1659662

Kline T, 2016, ART THER, V33, P67, DOI 10.1080/07421656.2016.1164002

Kolb B, 1999, CAN J EXP PSYCHOL, V53, P62, DOI 10.1037/h0087300

Lusebrink V.B., 2004, ART THERAPY J AM ART, V21, P125, DOI [10.1080/07421656.2004.10129496, DOI 10.1080/07421656.2004.10129496, https://doi.org/10.1080/07421656.2004.10129496]

Makuuchi M, 2003, COGNITIVE BRAIN RES, V16, P338, DOI 10.1016/S0926-6410(02)00302-6

McDonald B., 2020, J HUMANIT REHABIL

MCGRAW M, 1989, AM J ART THER, V28, P37

Merians AS, 2002, PHYS THER, V82, P898, DOI 10.1093/ptj/82.9.898

Osburn, 2017, BIOMED J SCI TECH RE, V1, P1, DOI [DOI 10.26717/BJSTR.2017.01.000366, https://doi.org/10.26717/BJSTR.2017.01.000366]

Paczynski A, 2017, HUM-COMPUT INT-SPRIN, P97, DOI 10.1007/978-3-319-60672-9_5

Page SJ, 2000, OCCUP THER J RES, V20, P200, DOI 10.1177/153944920002000304

Page SJ, 2001, PHYS THER, V81, P1455, DOI 10.1093/ptj/81.8.1455

Park DC, 2009, ANNU REV PSYCHOL, V60, P173, DOI 10.1146/annurev.psych.59.103006.093656

Perrin T., 2001, BRIT J OCCUP THER, V64, P129, DOI [https://doi.org/10.1177/030802260106400304, DOI 10.1177/030802260106400304]

Piron L, 2005, PRESENCE-TELEOP VIRT, V14, P732, DOI 10.1162/105474605775196580

Smith C, 2007, J SOC WORK PRACT, V21, P297, DOI 10.1080/02650530701553591

Smith D, 2017, ARTS HEALTH, V9, P251, DOI 10.1080/17533015.2017.1354899

Stepankova H, 2014, DEV PSYCHOL, V50, P1049, DOI 10.1037/a0034913

Stevens JA, 2003, ARCH PHYS MED REHAB, V84, P1090, DOI 10.1016/S0003-9993(03)00042-X

Symons J, 2011, BRIT J OCCUP THER, V74, P44, DOI 10.4276/030802211X12947686093729

Tawfik GM, 2019, TROP MED HEALTH, V47, DOI 10.1186/s41182-019-0165-6

Van Lith T., 2009, INT ENCY REHABILITAT

Vourvopoulos A, 2019, FRONT HUM NEUROSCI, V13, DOI 10.3389/fnhum.2019.00244

Worthen-Chaudhari L, 2013, NEUROREHABILITATION, V33, P481, DOI 10.3233/NRE-130981

Yoo E, 2001, ARCH PHYS MED REHAB, V82, P1213, DOI 10.1053/apmr.2001.25095

Zeki S, 2002, J CONSCIOUSNESS STUD, V9, P53

NR 52

TC 0

Z9 0

U1 7

U2 9

PU MDPI

PI BASEL

PA ST ALBAN-ANLAGE 66, CH-4052 BASEL, SWITZERLAND

EI 2079-9292

J9 ELECTRONICS-SWITZ

JI Electronics

PD SEP

PY 2021

VL 10

IS 18

AR 2314

DI 10.3390/electronics10182314

PG 17

WC Computer Science, Information Systems; Engineering, Electrical &

Electronic; Physics, Applied

WE Science Citation Index Expanded (SCI-EXPANDED)

SC Computer Science; Engineering; Physics

GA UV4PZ

UT WOS:000699463800001

OA gold, Green Published

DA 2022-06-21

ER

PT J

AU Alex, M

Wunsche, BC

Lottridge, D

AF Alex, Marylyn

Wunsche, Burkhard C.

Lottridge, Danielle

TI Virtual reality art-making for stroke rehabilitation: Field study and

technology probe

SO INTERNATIONAL JOURNAL OF HUMAN-COMPUTER STUDIES

LA English

DT Article

DE Accessibility; Virtual reality; Stroke; Art-making; Digital art; 3D

Painting

ID THERAPY; CREATIVITY

AB How can we better understand the process of therapeutic art-making for stroke rehabilitation, and what are design opportunities for virtual reality art-making for people with stroke-related impairments? We investigated this question in a two-part study with 14 amateur artists with disabilities resulting from stroke: a three-week field study and a technology probe consisting of experiential virtual reality interviews. We uncovered what participants made, the aesthetics of the materials and the process of making. The field study revealed inspirations around identity, situatedness of choices for tools in the social and physical environment, and a breadth of application techniques (e.g., dripping paint or use of tape) that varied in need for fine motor control. The experiential virtual reality interviews highlighted the need for control, the affordances of the medium, and the challenges in viewing and reflecting on work. Emergent art reflected qualities of the 3D paint and free-form gesture. Virtual reality and traditional art-making contrasted in the speed and finality of application, opportunities for iteration and reflection, and in the need for dexterity. We discuss strengths, weaknesses and implications for design of virtual reality art-making for those with stroke-related impairments.

C1 [Alex, Marylyn; Wunsche, Burkhard C.; Lottridge, Danielle] Univ Auckland, Sch Comp Sci, 38 Princes St, Auckland, New Zealand.

RP Alex, M (通讯作者)，Univ Auckland, Sch Comp Sci, 38 Princes St, Auckland, New Zealand.

EM marylyn.alex@auckland.ac.nz; burkhard@cs.auckland.ac.nz;

d.lottridge@auckland.ac.nz

OI Alex, Marylyn/0000-0002-8829-7931; Lottridge,

Danielle/0000-0002-5541-4425

CR Alankus Gazihan, 2012, P SIGCHI C HUM FACT, P2049, DOI [10.1145/2207676.2208354, DOI 10.1145/2207676.2208354]

Ali K, 2014, MED HUMANIT, V40, P56, DOI 10.1136/medhum-2013-010448

Aydn B, 2012, CURRENT APPROACHES P, V4, P69

Bassett S.F., 2003, NEW ZEAL J PHYSIOTHE, V31, P60, DOI [10.4172/2165-7025.1000e124, DOI 10.4172/2165-7025.1000E124]

Berge J., 1997, ACM SIGGRAPH 97 VIS, P131, DOI [10.1145/259081.259216, DOI 10.1145/259081.259216]

Boehner K, 2007, CONFERENCE ON HUMAN FACTORS IN COMPUTING SYSTEMS, VOLS 1 AND 2, P1077

Borrego A, 2019, FRONT NEUROL, V10, DOI 10.3389/fneur.2019.01061

Braun V., 2006, QUAL RES PSYCHOL, V3, P77, DOI [10.1191/1478088706qp063oa, DOI 10.1191/1478088706QP063OA]

Burke J W, 2010, 2010 2nd International Conference on Games and Virtual Worlds for Serious Applications (VS-GAMES 2010), P75, DOI 10.1109/VS-GAMES.2010.21

Cameirao MS, 2016, J NEUROENG REHABIL, V13, DOI 10.1186/s12984-016-0175-0

Vi CT, 2017, INT J HUM-COMPUT ST, V108, P1, DOI 10.1016/j.ijhcs.2017.06.004

Collie K., 2002, Journal of Technology in Human Services, V20, P155, DOI 10.1300/J017v20n01_11

Collie K., 1999, ART THER, V16, DOI [10.1080/07421656.1999.10129481, DOI 10.1080/07421656.1999.10129481]

Cornejo R, 2016, ACM CONFERENCE ON COMPUTER-SUPPORTED COOPERATIVE WORK AND SOCIAL COMPUTING (CSCW 2016), P1572, DOI 10.1145/2818048.2819960

Creed C., 2014, P 16 INT ACM SIGACCE, P253, DOI 10.1145/2661334.2661386.

Devendorf L., 2014, P 2014 COMP PUBL DES, P151, DOI [10.1145/2598784.2598787., DOI 10.1145/2598784.2598787]

Dourish Paul, 2004, ACTION IS FDN EMBODI

Eisner E. W., 2002, ARTS CREATION MIND

Eum Y, 2015, TOHOKU J EXP MED, V235, P17, DOI 10.1620/tjem.235.17

Faria AL, 2016, J NEUROENG REHABIL, V13, DOI 10.1186/s12984-016-0204-z

Farrow S., 2004, TECHNOL DISABIL, V16, P69, DOI [10.3233/tad-2004-16201, DOI 10.3233/TAD-2004-16201, 10.3233/TAD-2004-16201]

Hackett ML, 2005, STROKE, V36, P2296, DOI 10.1161/01.STR.0000183622.75135.a4

Hacmun I, 2018, FRONT PSYCHOL, V9, DOI 10.3389/fpsyg.2018.02082

Halverson ER, 2013, J LEARN SCI, V22, P121, DOI 10.1080/10508406.2011.639471

Hook K, 2018, INFORMATICS-BASEL, V5, DOI 10.3390/informatics5010008

Hutchinson H., 2003, P SIGCHI C HUMAN FAC, P17, DOI 10.1145/642611.642616

Jack D, 2001, IEEE T NEUR SYS REH, V9, P308, DOI 10.1109/7333.948460

Johansson S, 2015, ASSETS'15: PROCEEDINGS OF THE 17TH INTERNATIONAL ACM SIGACCESS CONFERENCE ON COMPUTERS & ACCESSIBILITY, P69, DOI 10.1145/2700648.2809849

Jones B., 2014, CHI 14 HUM FACT COMP, P1759, DOI [10.1145/2559206.2581302., DOI 10.1145/2559206.2581302]

Kara K.S., 1997, P C COMP HUM INT SPE, P235

Keefe D. F., 2001, P ACM S INT 3D GRAPH, P85

Keim D, 2008, LECT NOTES COMPUT SC, V4950, P154, DOI 10.1007/978-3-540-70956-5

Kim SH, 2008, ART THER, V25, P129, DOI 10.1080/07421656.2008.10129593

Kongkasuwan R, 2016, CLIN REHABIL, V30, P1016, DOI 10.1177/0269215515607072

LaViola Jr J.J., 2017, 3D USER INTERFACES T, Vsecond

Lazar A, 2018, PROCEEDINGS OF THE 2018 CHI CONFERENCE ON HUMAN FACTORS IN COMPUTING SYSTEMS (CHI 2018), DOI 10.1145/3173574.3173925

Lazar A, 2017, PROCEEDINGS OF THE 2017 ACM SIGCHI CONFERENCE ON HUMAN FACTORS IN COMPUTING SYSTEMS (CHI'17), P2175, DOI 10.1145/3025453.3025522

Lazar A, 2016, DIS 2016: PROCEEDINGS OF THE 2016 ACM CONFERENCE ON DESIGNING INTERACTIVE SYSTEMS, P1047, DOI 10.1145/2901790.2901854

Lohse KR, 2014, PLOS ONE, V9, DOI 10.1371/journal.pone.0093318

Maclean N, 2000, BMJ-BRIT MED J, V321, P1051, DOI 10.1136/bmj.321.7268.1051

Malchiodi, 2011, HDB ART THERAPY

Marks K., 2017, STEP MY VIRTUAL WORL, V12, P99

McLuhan Marshall, 1964, MEDIUM IS MESSAGE

Meijer-Degen F, 2006, ART PSYCHOTHER, V33, P167, DOI 10.1016/j.aip.2005.10.002

Nam S.H., 2015, SIGGRAPH ASIA 2015 A, P7, DOI 10.1145/2835641.2835648.

Paczynski A, 2017, HUM-COMPUT INT-SPRIN, P97, DOI 10.1007/978-3-319-60672-9_5

Patten SB, 2002, INT J PSYCHIAT MED, V32, P155, DOI 10.2190/2G2N-WE19-NM47-JNY8

Perera D., 2006, P 2006 IRMA INT C WA

Perera D, 2007, CC2007-CREATIVITY AND COGNITION 2007 SEEDING CREATIVITY: TOOLS, MEDIA, AND ENVIRONMENTS, P147

Pruen S., 2019, TILT BRUSH DRAWING H

RITTEL HWJ, 1973, POLICY SCI, V4, P155, DOI 10.1007/BF01405730

Rogerson MJ, 2016, 34TH ANNUAL CHI CONFERENCE ON HUMAN FACTORS IN COMPUTING SYSTEMS, CHI 2016, P3956, DOI 10.1145/2858036.2858433

Rose Dorian K, 2004, Top Stroke Rehabil, V11, P20, DOI 10.1310/XAUM-LPBM-0RXD-RLDK

Schultheis MT, 2001, REHABIL PSYCHOL, V46, P296, DOI 10.1037/0090-5550.46.3.296

Seo JH, 2018, CHI 2018: EXTENDED ABSTRACTS OF THE 2018 CHI CONFERENCE ON HUMAN FACTORS IN COMPUTING SYSTEMS, DOI 10.1145/3170427.3188476

Shneiderman B, 2007, COMMUN ACM, V50, P20, DOI 10.1145/1323688.1323689

Standen PJ, 2005, CYBERPSYCHOL BEHAV, V8, P272, DOI 10.1089/cpb.2005.8.272

Strong Rob, 1996, P CSCW, P29

Suchman L.A., 1987, 15 INT S ROBOT HUMAN

Teofilo M, 2016, P IEEE VIRT REAL ANN, P293, DOI 10.1109/VR.2016.7504769

Thomson K, 2014, INT J STROKE, V9, P479, DOI 10.1111/ijs.12263

Treadaway C, 2009, C & C 09: PROCEEDINGS OF THE 2009 ACM SIGCHI CONFERENCE ON CREATIVITY AND COGNITION, P185

Utas Akhan L., 2015, REHABILITATION NURSI, V42, P39

Veas E.E., 2010, P 9 INT C MOB UB MUL, P3

VONHIPPEL E, 1986, MANAGE SCI, V32, P791, DOI 10.1287/mnsc.32.7.791

Vourvopoulos A., 2014, P 11 C ADV COMP ENT, P26, DOI DOI 10.1145/2663-806.2663852

Wagner JL, 2009, J PEDIATR PSYCHOL, V34, P89, DOI 10.1093/jpepsy/jsn052

Wilson PN, 1997, DISABIL REHABIL, V19, P213, DOI 10.3109/09638289709166530

Worthen-Chaudhari L, 2013, NEUROREHABILITATION, V33, P481, DOI 10.3233/NRE-130981

NR 69

TC 2

Z9 2

U1 7

U2 35

PU ACADEMIC PRESS LTD- ELSEVIER SCIENCE LTD

PI LONDON

PA 24-28 OVAL RD, LONDON NW1 7DX, ENGLAND

SN 1071-5819

EI 1095-9300

J9 INT J HUM-COMPUT ST

JI Int. J. Hum.-Comput. Stud.

PD JAN

PY 2021

VL 145

AR 102481

DI 10.1016/j.ijhcs.2020.102481

PG 14

WC Computer Science, Cybernetics; Ergonomics; Psychology, Multidisciplinary

WE Science Citation Index Expanded (SCI-EXPANDED); Social Science Citation Index (SSCI)

SC Computer Science; Engineering; Psychology

GA OO3OH

UT WOS:000587291600016

DA 2022-06-21

ER

PT J

AU Ali, AS

Arumugam, A

Kumaran, DS

AF Ali, A. Sulfikar

Arumugam, Ashokan

Kumaran, Senthil D.

TI Effectiveness of an intensive, functional, gamified Rehabilitation

program in improving upper limb motor function in people with stroke: A

protocol of the EnteRtain randomized clinical trial

SO CONTEMPORARY CLINICAL TRIALS

LA English

DT Article

DE Commercial games; Game-based training; Hemiparesis; Hemiplegia; Upper

extremity; Virtual reality

ID VIDEO GAMES; RECOVERY; RELIABILITY; IMPAIRMENT; EXERCISES; VALIDITY

AB Introduction: Game-based rehabilitation is an emerging therapeutic intervention that allows intensive, repetitive, task-based training to improve upper limb (UL) function following stroke, based on the principles of neuroplasticity and motor (re)learning. Rehabilitation using commercial gaming system will be motivating, enjoyable, challenging and affordable. Therefore, the present study aims at assessing the effectiveness of an intensive, functional, gamified rehabilitation program using the ArmAble (TM) device in improving UL motor function in people with stroke.

Method: In this single-blinded, multi-centric, randomized clinical trial, 120 adults with acute/sub-acute unilateral stroke will be randomized to receive an intensive, functional, gamified training program using the ArmAble (TM) or task-based training along with a conventional therapy for 2 h/day, 6 days/week for 2 weeks, followed by a home-based, functional rehabilitation program for another 4 weeks (similar to 30 min/day, 6 days/week). Primary outcomes evaluated by a blinded assessor at the baseline, 2 weeks and 6 weeks' post-intervention will include the Fugl-Meyer assessment - upper extremity and the action research arm test. A linear mixed effect regression model or relevant non-parametric tests will be used to analyze the data for all outcomes. An intention-to-treat analysis will be used with missing data handled by multiple imputation.

Discussion: Rehabilitation provided with the ArmAble (TM) device, if found effective, can be used from the early stages post-stroke to provide intensive, repetitive, gamified training to improve UL motor function.

C1 [Ali, A. Sulfikar; Kumaran, Senthil D.] Manipal Acad Higher Educ, Manipal Coll Hlth Profess, Dept Physiotherapy, Manipal 576104, Karnataka, India.

[Arumugam, Ashokan] Univ Sharjah, Dept Physiotherapy, Coll Hlth Sci, POB 27272, Sharjah, U Arab Emirates.

[Arumugam, Ashokan] Univ Sharjah, RISE Res Inst Sci & Engn, Sustainable Engn Asset Management Res Grp, POB 27272, Sharjah, U Arab Emirates.

[Arumugam, Ashokan] Manipal Acad Higher Educ, Manipal Coll Hlth Profess, Manipal 576104, Karnataka, India.

RP Kumaran, DS (通讯作者)，Manipal Acad Higher Educ, Manipal Coll Hlth Profess, Dept Physiotherapy, Manipal 576104, Karnataka, India.

EM sulfikar.ali@learner.manipal.edu; aarumugam@sharjah.ac.ae;

senthil.kumaran@manipal.edu

RI D, Senthil Kumaran/AAJ-5399-2021; Arumugam, Ashokan/ABA-4953-2022

OI D, Senthil Kumaran/0000-0001-6491-2584; Arumugam,

Ashokan/0000-0001-5795-3812

FU BeAble Health Pvt Ltd. (IIT Hyderabad, Telangana, India)

FX This study is partially funded by an industry grant provided through the

BeAble Health Pvt Ltd. (IIT Hyderabad, Telangana, India) , a company

that manufactures and markets the ArmAbleTM device. However, the funders

do not have any role in the conception, design, execution, data analysis

and interpretation, and dissemination of the findings of this study.

CR Abdullahi A, 2018, NEUROL RES INT, V2018, DOI 10.1155/2018/5496408

[Anonymous], 1997, JAMA-J AM MED ASSOC, V277, P925

Askin A, 2018, SOMATOSENS MOT RES, V35, P25, DOI 10.1080/08990220.2018.1444599

Barrett N, 2016, J Rehabil Assist Technol Eng, V3, p2055668316643644, DOI 10.1177/2055668316643644

Bassolino M, 2015, DEV MED CHILD NEUROL, V57, P42, DOI 10.1111/dmcn.12686

Carod-Artal FJ, 2008, STROKE, V39, P2477, DOI 10.1161/STROKEAHA.107.513671

Chan AW, 2013, BMJ-BRIT MED J, V346, DOI 10.1136/bmj.e7586

COLLIN C, 1990, J NEUROL NEUROSUR PS, V53, P576, DOI 10.1136/jnnp.53.7.576

Edwards DF, 2012, ARCH PHYS MED REHAB, V93, P660, DOI 10.1016/j.apmr.2011.10.005

Fayazi Maryam, 2012, Med J Islam Repub Iran, V26, P27

Hayward K, 2010, DISABIL REHABIL, V32, P1973, DOI 10.3109/09638288.2010.481027

Hayward KS, 2015, CLIN REHABIL, V29, P1234, DOI 10.1177/0269215514565395

Hoffmann TC, 2014, BMJ-BRIT MED J, V348, DOI [10.1136/bmj.g1687, 10.1055/s-0041-111066]

Hsieh CL, 1998, AGE AGEING, V27, P107, DOI 10.1093/ageing/27.2.107

Hubbard IJ, 2015, NEUROREHAB NEURAL RE, V29, P703, DOI 10.1177/1545968314562647

Hussain N, 2018, FRONT NEUROL, V9, DOI 10.3389/fneur.2018.00300

Kilbride C, 2018, BMJ OPEN, V8, DOI 10.1136/bmjopen-2018-026620

Kleim JA, 1998, J NEUROPHYSIOL, V80, P3321, DOI 10.1152/jn.1998.80.6.3321

Kottink AIR, 2014, GAMES HEALTH J, V3, P184, DOI 10.1089/g4h.2014.0026

Kwakkel G, 2017, NEUROREHAB NEURAL RE, V31, P784, DOI 10.1177/1545968317732662

Laffont I, 2020, ANN PHYS REHABIL MED, V63, P173, DOI 10.1016/j.rehab.2019.10.009

Lang CE, 2008, ARCH PHYS MED REHAB, V89, P1693, DOI 10.1016/j.apmr.2008.02.022

Langhorne P., 2014, COCHRANE DB SYST REV, V11

Laver KE, 2011, COCHRANE DB SYST REV, DOI [10.1002/14651858.CD008349.pub2, 10.1002/14651858.CD008349.pub4, 10.1002/14651858.CD008349.pub3]

Lee NK, 2013, NEUROREHABILITATION, V33, P177, DOI 10.3233/NRE-130943

Lohse KR, 2014, PLOS ONE, V9, DOI 10.1371/journal.pone.0093318

MALOUIN F, 1994, ARCH PHYS MED REHAB, V75, P1206, DOI 10.1016/0003-9993(94)90006-X

Meng GL, 2018, FRONT NEUROL, V8, DOI 10.3389/fneur.2017.00726

Park Yong Keun, 2017, J Phys Ther Sci, V29, P1390, DOI 10.1589/jpts.29.1390

Phan MH, 2016, HUM FACTORS, V58, P1217, DOI 10.1177/0018720816669646

Pietrzah E, 2014, TOP STROKE REHABIL, V21, P152, DOI 10.1310/tsr2102-152

Prakash S.C., 2020, 2020 INT C SIGN PROC, P1

Richardson M, 2016, DISABIL REHABIL, V38, P1425, DOI 10.3109/09638288.2015.1102337

Rorden C, 2010, NEUROPSYCHOLOGIA, V48, P2758, DOI 10.1016/j.neuropsychologia.2010.04.018

Shelton FDAP, 2001, NEUROREHAB NEURAL RE, V15, P229, DOI 10.1177/154596830101500311

Song GB, 2015, J PHYS THER SCI, V27, P1353, DOI 10.1589/jpts.27.1353

Subramanian SK, 2022, DISABIL REHABIL-ASSI, V17, P107, DOI 10.1080/17483107.2020.1765422

Subramanian SK, 2013, NEUROREHAB NEURAL RE, V27, P13, DOI 10.1177/1545968312449695

Sullivan KJ, 2011, STROKE, V42, P427, DOI 10.1161/STROKEAHA.110.592766

Taub E, 2006, Eura Medicophys, V42, P241

Taut D, 2017, NEUROREHABILITATION, V41, P105, DOI 10.3233/NRE-171462

Tseng BY, 2010, STROKE RES TREAT, V2010, DOI 10.4061/2010/412964

Vanbellingen T, 2017, FRONT NEUROL, V8, DOI 10.3389/fneur.2017.00654

Veerbeek JM, 2014, PLOS ONE, V9, DOI 10.1371/journal.pone.0087987

W Bohannon R., 1999, J PHYS THER SCI, V11, P59, DOI [10.1589/jpts.11.59, DOI 10.1589/JPTS.11.59]

Zhang C, 2017, INT J REHABIL RES, V40, P19, DOI 10.1097/MRR.0000000000000204

NR 46

TC 1

Z9 1

U1 3

U2 9

PU ELSEVIER SCIENCE INC

PI NEW YORK

PA STE 800, 230 PARK AVE, NEW YORK, NY 10169 USA

SN 1551-7144

EI 1559-2030

J9 CONTEMP CLIN TRIALS

JI Contemp. Clin. Trials

PD JUN

PY 2021

VL 105

AR 106381

DI 10.1016/j.cct.2021.106381

EA APR 2021

PG 8

WC Medicine, Research & Experimental; Pharmacology & Pharmacy

WE Science Citation Index Expanded (SCI-EXPANDED); Social Science Citation Index (SSCI)

SC Research & Experimental Medicine; Pharmacology & Pharmacy

GA SL2YE

UT WOS:000656784700016

PM 33862286

DA 2022-06-21

ER

PT J

AU Allain, P

Foloppe, DA

Besnard, J

Yamaguchi, T

Etcharry-Bouyx, F

Le Gall, D

Nolin, P

Richard, P

AF Allain, Philippe

Foloppe, Deborah Alexandra

Besnard, Jeremy

Yamaguchi, Takehiko

Etcharry-Bouyx, Frederique

Le Gall, Didier

Nolin, Pierre

Richard, Paul

TI Detecting Everyday Action Deficits in Alzheimer's Disease Using a

Nonimmersive Virtual Reality Kitchen

SO JOURNAL OF THE INTERNATIONAL NEUROPSYCHOLOGICAL SOCIETY

LA English

DT Article

DE Alzheimer's disease; Virtual reality; Action; Activities of daily living

(ADL); Instrumental activities of daily living (IADL);

Neuropsychological assessment

ID MILD COGNITIVE IMPAIRMENT; MEMORY; REHABILITATION; MANAGEMENT;

PREDICTOR; DEMENTIA; COFFEE; APATHY

AB Alzheimer's disease (AD) causes impairments affecting instrumental activities of daily living (IADL). Transdisciplinary research in neuropsychology and virtual reality has fostered the development of ecologically valid virtual tools for the assessment of IADL, using simulations of real life activities. Few studies have examined the benefits of this approach in AD patients. Our aim was to examine the utility of a non-immersive virtual coffee task (NI-VCT) for assessment of IADL in these patients. We focus on the assessment results obtained from a group of 24 AD patients on a task designed to assess their ability to prepare a virtual cup of coffee, using a virtual coffee machine. We compared performance on the virtual task to an identical daily living task involving the actual preparation of a cup of coffee, as well as to global cognitive, executive, and caregiver-reported IADL functioning. Relative to 32 comparable, healthy elderly (HE) controls, AD patients performed worse than HE controls on all tasks. Correlation analyses revealed that NI-VCT measures were related to all other neuropsychological measures. Moreover, regression analyses demonstrated that performance on the NI-VCT predicted actual task performance and caregiver-reported IADL functioning. Our results provide initial support for the utility of our virtual kitchen for assessment of IADL in AD patients.

C1 [Allain, Philippe; Foloppe, Deborah Alexandra; Besnard, Jeremy; Etcharry-Bouyx, Frederique; Le Gall, Didier] Univ Angers, UPRES EA 4638, LUNAM Univ, Lab Psychol Pays Loire, Angers, France.

[Allain, Philippe; Etcharry-Bouyx, Frederique; Le Gall, Didier] CHU Angers, Dept Neurol, Unite Neuropsychol, F-49033 Angers 09, France.

[Foloppe, Deborah Alexandra; Richard, Paul] Univ Angers, UPRES EA 7315, LUNAM Univ, Lab Angevin Rech Ingn Syst, Angers, France.

[Yamaguchi, Takehiko] Tokyo Univ Sci, Dept Appl Elect, Fac Ind Sci & Technol, Tokyo 162, Japan.

[Nolin, Pierre] Univ Quebec Trois Rivieres, Lab Rech Interdisciplinaire Realite Virtuelle, Trois Rivieres, PQ, Canada.

RP Allain, P (通讯作者)，CHU Angers, Dept Neurol, Unite Neuropsychol, 4 Rue Larrey, F-49033 Angers 09, France.

EM phallain@chu-angers.fr

RI Yamaguchi, Takehiko/AAE-2907-2022

OI Foloppe, Deborah/0000-0001-8508-1572; ALLAIN,

Philiooe/0000-0003-0668-0986; Besnard, Jeremy/0000-0001-7127-7558

FU "Conseil Scientifique" of the University of Angers in France and from

France Alzheimer

FX This study was supported by a Grant from the "Conseil Scientifique" of

the University of Angers in France and from France Alzheimer. The

authors have no conflicts of interest to declare. We thank Dr. John L.

Woodard for his editing assistance, under the auspices of the Research

and Editing Consultant Program of the International Neuropsychological

Society International Liaison Committee. Doctors Allain and Foloppe have

equally contributed to the preparation of this manuscript.

CR Adam S, 2000, NEUROPSYCHOL REHABIL, V10, P485, DOI 10.1080/09602010050143568

Agniel A., 1992, PROTOCOLE MONTREAL T

Barberger-Gateau P, 1999, J GERONTOL B-PSYCHOL, V54, pP293, DOI 10.1093/geronb/54B.5.P293

BELAND R, 1992, PROTOCOLE MONTREAL T

Bialystok E, 2008, J INT NEUROPSYCH SOC, V14, P257, DOI 10.1017/S1355617708080296

Botvinick M, 2004, PSYCHOL REV, V111, P395, DOI 10.1037/0033-295X.111.2.395

Boyle PA, 2003, AM J GERIAT PSYCHIAT, V11, P214, DOI 10.1176/appi.ajgp.11.2.214

Cahn-Weiner DA, 2003, J GERIATR PSYCH NEUR, V16, P84, DOI 10.1177/0891988703016002004

Chaytor N, 2006, ARCH CLIN NEUROPSYCH, V21, P217, DOI 10.1016/j.acn.2005.12.002

Cooper R, 2000, COGN NEUROPSYCHOL, V17, P297, DOI 10.1080/026432900380427

Cushman LA, 2008, NEUROLOGY, V71, P888, DOI 10.1212/01.wnl.0000326262.67613.fe

Debettignies Barbara H., 1993, Clinical Gerontologist, V12, P31, DOI 10.1300/J018v12n04_03

Dickinson A, 2007, BEHAV INFORM TECHNOL, V26, P343, DOI 10.1080/01449290601176948

Dubois B, 2000, NEUROLOGY, V55, P1621, DOI 10.1212/WNL.55.11.1621

Festa EK, 2010, NEUROPSYCHOLOGIA, V48, P3252, DOI 10.1016/j.neuropsychologia.2010.07.003

FOLSTEIN MF, 1975, J PSYCHIAT RES, V12, P189, DOI 10.1016/0022-3956(75)90026-6

Giovannetti T, 2006, J INT NEUROPSYCH SOC, V12, P45, DOI 10.1017/S1355617706060012

Giovannetti T, 2008, DEMENT GERIATR COGN, V25, P359, DOI 10.1159/000121005

Giovannetti T, 2008, NEUROPSYCHOLOGY, V22, P235, DOI 10.1037/0894-4105.22.2.235

Giovannetti T, 2007, J CLIN EXP NEUROPSYC, V29, P690, DOI 10.1080/13803390600932286

Josman N, 2008, INT J DISABIL HUM DE, V7, P49

Kawano N, 2012, AGING CLIN EXP RES, V24, P285, DOI 10.1007/BF03325260

Kluger A, 1997, J GERONTOL B-PSYCHOL, V52, pP28, DOI 10.1093/geronb/52B.1.P28

LAWTON MP, 1969, GERONTOLOGIST, V9, P9, DOI 10.1093/geront/9.1.9

Lechowski L, 2003, INT J GERIATR PSYCH, V18, P977, DOI 10.1002/gps.999

Lee JH, 2003, CYBERPSYCHOL BEHAV, V6, P383, DOI 10.1089/109493103322278763

Mast BT, 2004, REHABIL PSYCHOL, V49, P219, DOI 10.1037/0090-5550.49.3.219

MCKHANN G, 1984, NEUROLOGY, V34, P939, DOI 10.1212/WNL.34.7.939

Millan-Calenti JC, 2012, ARCH GERONTOL GERIAT, V54, P197, DOI 10.1016/j.archger.2011.02.010

Mitchell MB, 2011, GERONTOLOGIST, V51, P179, DOI 10.1093/geront/gnq087

Noale M, 2003, DEMENT GERIATR COGN, V16, P7, DOI 10.1159/000069987

Nolin P, 2012, BRAIN INJURY, V26, P1564, DOI 10.3109/02699052.2012.698359

Plancher G, 2012, NEUROPSYCHOLOGIA, V50, P592, DOI 10.1016/j.neuropsychologia.2011.12.013

Ramsden CM, 2008, NEUROPSYCHOLOGY, V22, P17, DOI 10.1037/0894-4105.22.1.17

Rizzo AA, 2004, NEUROPSYCHOL REHABIL, V14, P207, DOI 10.1080/09602010343000183

Rusted J, 2002, NEUROCASE, V8, P111, DOI 10.1093/neucas/8.1.111

Schwartz MF, 1998, NEUROPSYCHOLOGY, V12, P13, DOI 10.1037/0894-4105.12.1.13

Schwartz MF, 2002, NEUROPSYCHOL REHABIL, V12, P311, DOI 10.1080/09602010244000084

Segal, 2003, NATURALISTIC ACTION

Senonarong V, 2005, INT PSYCHOGERIATR, V17, P81, DOI 10.1017/S1041610205000980

Starkstein SE, 2010, J NEUROPSYCH CLIN N, V22, P378, DOI 10.1176/appi.neuropsych.22.4.378

Staub B, 2013, AGEING RES REV, V12, P459, DOI 10.1016/j.arr.2012.12.001

Tekin S, 2001, AM J GERIAT PSYCHIAT, V9, P81, DOI 10.1176/appi.ajgp.9.1.81

Werner P, 2009, DEMENT GERIATR COGN, V27, P301, DOI 10.1159/000204915

Widmann CN, 2012, NEUROBIOL AGING, V33, P297, DOI 10.1016/j.neurobiolaging.2010.03.012

Wood E, 2005, J APPL GERONTOL, V24, P419, DOI 10.1177/0733464805278378

Yamaguchi T, 2012, PRESENCE-TELEOP VIRT, V21, P43, DOI 10.1162/PRES_a_00080

Yan JH, 2008, J PSYCHIATR RES, V42, P1203, DOI 10.1016/j.jpsychires.2008.01.006

Zhang L, 2003, ARCH PHYS MED REHAB, V84, P1118, DOI 10.1016/S0003-9993(03)00203-X

NR 49

TC 59

Z9 61

U1 2

U2 46

PU CAMBRIDGE UNIV PRESS

PI NEW YORK

PA 32 AVENUE OF THE AMERICAS, NEW YORK, NY 10013-2473 USA

SN 1355-6177

EI 1469-7661

J9 J INT NEUROPSYCH SOC

JI J. Int. Neuropsychol. Soc.

PD MAY

PY 2014

VL 20

IS 5

BP 468

EP 477

DI 10.1017/S1355617714000344

PG 10

WC Clinical Neurology; Neurosciences; Psychiatry; Psychology

WE Science Citation Index Expanded (SCI-EXPANDED); Social Science Citation Index (SSCI)

SC Neurosciences & Neurology; Psychiatry; Psychology

GA AI0SH

UT WOS:000336559700002

PM 24785240

DA 2022-06-21

ER

PT J

AU Allegue, DR

Kairy, D

Higgins, J

Archambault, PS

Michaud, F

Miller, WC

Sweet, SN

Tousignant, M

AF Allegue, Dorra Rakia

Kairy, Dahlia

Higgins, Johanne

Archambault, Philippe S.

Michaud, Francois

Miller, William C.

Sweet, Shane N.

Tousignant, Michel

TI A Personalized Home-Based Rehabilitation Program Using Exergames

Combined With a Telerehabilitation App in a Chronic Stroke Survivor:

Mixed Methods Case Study

SO JMIR SERIOUS GAMES

LA English

DT Article

DE stroke; rehabilitation; virtual reality; video games;

telerehabilitation; upper extremity; motivation

ID CLINICALLY IMPORTANT DIFFERENCES; UNIFIED THEORY; PHYSICAL-ACTIVITY;

MOVEMENT THERAPY; TECHNOLOGY; ACCEPTANCE; BEHAVIOR; RECOMMENDATIONS;

INTERVENTIONS; RECOVERY

AB Background: In Canada, only 11% of stroke survivors have access to outpatient and community-based rehabilitation after discharge from inpatient rehabilitation. Hence, innovative community-based strategies are needed to provide adequate postrehabilitation services. The VirTele program, which combines virtual reality exergames and a telerehabilitation app, was developed to provide stroke survivors with residual upper extremity deficits, the opportunity to participate in a personalized home rehabilitation program. Objective: This study aims to determine the feasibility of VirTele for remote upper extremity rehabilitation in a chronic stroke survivor; explore the preliminary efficacy of VirTele on upper extremity motor function, the amount and quality of upper extremity use, and impact on quality of life and motivation; and explore the determinants of behavioral intention and use behavior of VirTele along with indicators of empowerment. Methods: A 63-year-old male stroke survivor (3 years) with moderate upper extremity impairment participated in a 2-month VirTele intervention. He was instructed to use exergames (5 games for upper extremity) for 30 minutes, 5 times per week, and conduct videoconference sessions with a clinician at least once per week. Motivational interviewing was incorporated into VirTele to empower the participant to continue exercising and use his upper extremities in everyday activities. Upper extremity motor function (Fugl-Meyer Assessment-upper extremity), amount and quality of upper extremity use (Motor Activity Log-30), and impact on quality of life (Stroke Impact Scale-16) and motivation (Treatment Self-Regulation Questionnaire-15) were measured before (T1), after (T2) VirTele intervention, and during a 1-(T3) and 2-month (T4) follow-up period. Qualitative data were collected through logs and semistructured interviews. Feasibility data (eg, number and duration of videoconference sessions and adherence) were documented at the end of each week. Results: The participant completed 48 exergame sessions (33 hours) and 8 videoconference sessions. Results suggest that the VirTele intervention and the study protocol could be feasible for stroke survivors. The participant exhibited clinically meaningful improvements at T2 on the Fugl-Meyer and Stroke Impact Scale-16 and maintained these gains at T3 and T4. During the follow-up periods, the amount and quality of upper extremity use showed meaningful changes, suggesting more involvement of the affected upper extremity in daily activities. The participant demonstrated a high level of autonomous motivation, which may explain his adherence. Performance, effort, and social influence have meaningful weights in the behavioral intention of using VirTele. However, the lack of control of technical and organizational infrastructures may influence the long-term use of technology. At the end of the intervention, the participant demonstrated considerable empowerment at both the behavioral and capacity levels. Conclusions: VirTele was shown to be feasible for use in chronic stroke survivors for remote upper extremity rehabilitation. Meaningful determinants of behavioral intention and use behavior of VirTele were identified, and preliminary efficacy results are promising. International Registered Report Identifier (IRRID): RR2-10.2196/14629

C1 [Allegue, Dorra Rakia; Kairy, Dahlia; Higgins, Johanne] Univ Montreal, Sch Rehabil, Montreal, PQ, Canada.

[Allegue, Dorra Rakia; Kairy, Dahlia; Higgins, Johanne; Archambault, Philippe S.; Sweet, Shane N.] Inst Univ Readaptat Deficience Phys Montreal, Ctr Interdisciplinary Res Rehabil Greater Montrea, 6363 Chemin Hudson Fifth Floor, Montreal, PQ H3S 1M9, Canada.

[Allegue, Dorra Rakia] Mission Univ Tunisie, Montreal, PQ, Canada.

[Archambault, Philippe S.] McGill Univ, Sch Phys & Occupat Therapy, Montreal, PQ, Canada.

[Michaud, Francois] Univ Sherbrooke, Dept Elect Engn & Comp Engn, Sherbrooke, PQ, Canada.

[Miller, William C.] Univ British Columbia, Dept Occupat Sci & Occupat Therapy, Vancouver, BC, Canada.

[Sweet, Shane N.] McGill Univ, Dept Kinesiol & Phys Educ, Montreal, PQ, Canada.

[Tousignant, Michel] Univ Sherbrooke, Fac Med & Hlth Sci, Sch Rehabil, Sherbrooke, PQ, Canada.

[Tousignant, Michel] Ctr Res Aging, Sherbrooke, PQ, Canada.

RP Allegue, DR (通讯作者)，Inst Univ Readaptat Deficience Phys Montreal, Ctr Interdisciplinary Res Rehabil Greater Montrea, 6363 Chemin Hudson Fifth Floor, Montreal, PQ H3S 1M9, Canada.

EM dorra.rakia.allegue@umontreal.ca

RI Allegue, Dorra Rakia/ACE-8866-2022; Archambault, Philippe S./F-4675-2010

OI Michaud, Francois/0000-0002-3639-7770; Miller,

William/0000-0003-3060-0210; Sweet, Shane/0000-0002-6172-3769;

Archambault, Philippe S./0000-0002-8656-4477; Tousignant,

Michel/0000-0001-7561-1170; Higgins, Johanne/0000-0003-1513-6587

FU Canadian Institutes of Health Research [385297]; Mission Universitaire

de Tunisie

FX This work was supported by the Canadian Institutes of Health Research

(385297, 2017) and a scholarship from the Mission Universitaire de

Tunisie. The funding source had no involvement in the research or

preparation of the paper.

CR Adie K, 2017, CLIN REHABIL, V31, P173, DOI 10.1177/0269215516637893

Allegue DR, 2020, JMIR RES PROTOC, V9, DOI 10.2196/14629

Amano S, 2018, TOP STROKE REHABIL, V25, P432, DOI 10.1080/10749357.2018.1481569

[Anonymous], 2009, 2009 TRACK HEART DIS

[Anonymous], 2014, LIFE STROKES

Bravo P, 2015, BMC HEALTH SERV RES, V15, DOI 10.1186/s12913-015-0907-z

Calabro RS, 2017, J NEUROENG REHABIL, V14, DOI 10.1186/s12984-017-0268-4

Carraro N, 2013, PSYCHOL SPORT EXERC, V14, P228, DOI 10.1016/j.psychsport.2012.10.004

Caughlin S, 2020, TELEMED E-HEALTH, V26, P710, DOI 10.1089/tmj.2019.0097

Chan DK, 2009, ARCH PHYS MED REHAB, V90, P1977, DOI 10.1016/j.apmr.2009.05.024

Chen K, 2014, ERGONOMICS, V57, P635, DOI 10.1080/00140139.2014.895855

Chou CY, 2015, CLIN REHABIL, V29, P816, DOI 10.1177/0269215514555137

Cikajlo I, 2012, DISABIL REHABIL, V34, P13, DOI 10.3109/09638288.2011.583308

Conner M, 2017, PSYCHOL HEALTH, V32, P895, DOI 10.1080/08870446.2017.1336240

Deci EL, 2000, PSYCHOL INQ, V11, P227, DOI 10.1207/S15327965PLI1104_01

DUNCAN PW, 1992, STROKE, V23, P1084, DOI 10.1161/01.STR.23.8.1084

Dwivedi YK, 2020, CURR OPIN PSYCHOL, V36, P13, DOI 10.1016/j.copsyc.2020.03.008

Edwards B, 2003, QUAL LIFE RES, V12, P1127, DOI 10.1023/A:1026109920478

Faric N, 2019, J MED INTERNET RES, V21, DOI 10.2196/13833

Fritz SL, 2007, PHYS THER, V87, P170, DOI 10.2522/ptj.20060101

Fulk GD, 2010, TOP STROKE REHABIL, V17, P477, DOI 10.1310/tsr1706-477

Gauthier LV, 2017, BMC NEUROL, V17, DOI 10.1186/s12883-017-0888-0

Hardcastle SJ, 2017, HEALTH PSYCHOL REV, V11, P1, DOI 10.1080/17437199.2016.1190659

Harrison, 1985, PHYSIOTHER CAN, V37, P65, DOI DOI 10.3138/PTC.37.2.065

Hebert D, 2016, INT J STROKE, V11, P459, DOI 10.1177/1747493016643553

Kwakkel G, 2004, STROKE, V35, P2529, DOI 10.1161/01.STR.0000143153.76460.7d

Lang CE, 2008, ARCH PHYS MED REHAB, V89, P1693, DOI 10.1016/j.apmr.2008.02.022

Laver KE, 2011, COCHRANE DB SYST REV, DOI [10.1002/14651858.CD008349.pub2, 10.1002/14651858.CD008349.pub4, 10.1002/14651858.CD008349.pub3]

Levesque CS, 2007, HEALTH EDUC RES, V22, P691, DOI 10.1093/her/cyl148

Lincoln Y.S., 1985, NATURALISTIC INQUIRY

Linder SM, 2015, AM J OCCUP THER, V69, DOI 10.5014/ajot.2015.014498

Michie S, 2013, ANN BEHAV MED, V46, P81, DOI 10.1007/s12160-013-9486-6

Michie S, 2011, IMPLEMENT SCI, V6, DOI 10.1186/1748-5908-6-42

Michie S, 2010, HEALTH PSYCHOL, V29, P1, DOI 10.1037/a0016939

Michie S, 2009, HEALTH PSYCHOL, V28, P690, DOI 10.1037/a0016136

Silva ESM, 2019, PHYSIOTHER THEOR PR, V35, P964, DOI 10.1080/09593985.2018.1460430

Mouawad MR, 2011, J REHABIL MED, V43, P527, DOI 10.2340/16501977-0816

Page SJ, 2015, PHYS THER, V95, P103, DOI 10.2522/ptj.20130235

Page SJ, 2012, PHYS THER, V92, P791, DOI 10.2522/ptj.20110009

Painter JE, 2008, ANN BEHAV MED, V35, P358, DOI 10.1007/s12160-008-9042-y

Piron L, 2002, STUD HEALTH TECHNOL, V85, P355

Prestwich A, 2014, HEALTH PSYCHOL, V33, P465, DOI 10.1037/a0032853

Ryan RM, 2017, SELF-DETERMINATION THEORY: BASIC PSYCHOLOGICAL NEEDS IN MOTIVATION, DEVELOPMENT, AND WELLNESS, P1, DOI 10.1521/978.14625/28806

Singer Barbara, 2017, J Physiother, V63, P53, DOI 10.1016/j.jphys.2016.08.010

Slater M, 1997, PRESENCE-TELEOP VIRT, V6, P603, DOI 10.1162/pres.1997.6.6.603

Standen PJ, 2017, CLIN REHABIL, V31, P340, DOI 10.1177/0269215516640320

Sullivan KJ, 2011, STROKE, V42, P427, DOI 10.1161/STROKEAHA.110.592766

van der Lee JH, 2004, STROKE, V35, P1410, DOI 10.1161/01.STR.0000126900.24964.7e

Venkatesh V, 2016, J ASSOC INF SYST, V17, P328, DOI 10.17705/1jais.00428

Weiss PLT, 2014, VIRTUAL REALITY PHYS

Williams MD, 2015, J ENTERP INF MANAG, V28, P443, DOI 10.1108/JEIM-09-2014-0088

NR 51

TC 1

Z9 1

U1 2

U2 6

PU JMIR PUBLICATIONS, INC

PI TORONTO

PA 130 QUEENS QUAY E, STE 1102, TORONTO, ON M5A 0P6, CANADA

SN 2291-9279

J9 JMIR SERIOUS GAMES

JI JMIR Serious Games

PD JUL-SEP

PY 2021

VL 9

IS 3

AR e26153

DI 10.2196/26153

PG 16

WC Health Care Sciences & Services; Public, Environmental & Occupational

Health; Medical Informatics

WE Science Citation Index Expanded (SCI-EXPANDED); Social Science Citation Index (SSCI)

SC Health Care Sciences & Services; Public, Environmental & Occupational

Health; Medical Informatics

GA WD2OP

UT WOS:000704787100006

PM 34132649

OA gold, Green Published

DA 2022-06-21

ER

PT J

AU Allison, SL

Fagan, AM

Morris, JC

Head, D

AF Allison, Samantha L.

Fagan, Anne M.

Morris, John C.

Head, Denise

TI Spatial Navigation in Preclinical Alzheimer's Disease

SO JOURNAL OF ALZHEIMERS DISEASE

LA English

DT Article

DE Aging; allocentric; amyloid; caudate nucleus; egocentric; hippocampus

ID MILD COGNITIVE IMPAIRMENT; A-BETA-DEPOSITION; MORRIS WATER MAZE; CSF

BIOMARKERS; DEMENTIA; DECLINE; MEMORY; BRAIN; OUTCOMES; CORTEX

AB Although several previous studies have demonstrated navigational deficits in early-stage symptomatic Alzheimer's disease (AD), navigational abilities in preclinical AD have not been examined. The present investigation examined the effects of preclinical AD and early-stage symptomatic AD on spatial navigation performance. Performance on tasks of wayfinding and route learning in a virtual reality environment were examined. Comparisons were made across the following three groups: Clinically normal without preclinical AD (n = 42), clinically normal with preclinical AD (n = 13), and early-stage symptomatic AD (n = 16) groups. Preclinical AD was defined based on cerebrospinal fluid A beta(42) levels below 500 pg/ml. Preclinical AD was associated with deficits in the use of a wayfinding strategy, but not a route learning strategy. Moreover, post-hoc analyses indicated that wayfinding performance had moderate sensitivity and specificity. Results also confirmed early-stage symptomatic AD-related deficits in the use of both wayfinding and route learning strategies. The results of this study suggest that aspects of spatial navigation may be particularly sensitive at detecting the earliest cognitive deficits of AD.

C1 [Allison, Samantha L.; Head, Denise] Washington Univ, Dept Psychol, St Louis, MO 63130 USA.

[Fagan, Anne M.; Morris, John C.; Head, Denise] Washington Univ, Knight Alzheimers Dis Res Ctr, St Louis, MO 63130 USA.

[Fagan, Anne M.] Washington Univ, Hope Ctr Neurol Disorders, St Louis, MO 63130 USA.

[Fagan, Anne M.; Morris, John C.] Washington Univ, Dept Neurol, St Louis, MO 63130 USA.

[Head, Denise] Washington Univ, Dept Radiol, St Louis, MO 63130 USA.

RP Head, D (通讯作者)，Washington Univ, One Brookings Dr,Box 1125, St Louis, MO 63130 USA.

EM dhead@wustl.edu

FU NIH [P50 AG05861, P01 AG03991, P01 AG026276]; National Institute on

Aging [5T32AG00030]; NATIONAL INSTITUTE ON AGING [F32AG005861,

P50AG005681, P01AG003991, T32AG000030, P01AG026276] Funding Source: NIH

RePORTER

FX This work was supported by NIH grants P50 AG05861, P01 AG03991, and P01

AG026276. S. Allison was supported by National Institute on Aging

5T32AG00030. We thank our lumbar puncture physicians for obtaining our

CSF samples, M. Amos and A. Shah for processing and analyzing the CSF

samples, S. Sathyan for scheduling the lumbar punctures, and the

Clinical Core of the Knight Alzheimer's Disease Research Center for

participant assessments. We thank Chauncey Scott and Tyler Blazey for

assistance with the development of the maze environments and programming

the maze tasks.

CR Barnes J, 2009, NEUROBIOL AGING, V30, P1711, DOI 10.1016/j.neurobiolaging.2008.01.010

Becker JA, 2011, ANN NEUROL, V69, P1032, DOI 10.1002/ana.22333

Bellassen V, 2012, J NEUROSCI, V32, P1942, DOI 10.1523/JNEUROSCI.4556-11.2012

Berg L, 1998, ARCH NEUROL-CHICAGO, V55, P326, DOI 10.1001/archneur.55.3.326

Cherrier MM, 2001, NEUROPSY NEUROPSY BE, V14, P159

Chetelat G, 2012, NEURODEGENER DIS, V10, P141

Cohen J., 2003, APPL MULTIPLE REGRES, V3rd

Cushman LA, 2008, NEUROLOGY, V71, P888, DOI 10.1212/01.wnl.0000326262.67613.fe

deBruin JPC, 1997, BEHAV BRAIN RES, V85, P47, DOI 10.1016/S0166-4328(96)00163-5

Deipolyi AR, 2007, NEUROLOGY, V69, P986, DOI 10.1212/01.wnl.0000271376.19515.c6

DELONG ER, 1988, BIOMETRICS, V44, P837, DOI 10.2307/2531595

Doraiswamy PM, 2012, NEUROLOGY, V79, P1636, DOI 10.1212/WNL.0b013e3182661f74

Drzezga A, 2011, BRAIN, V134, P1635, DOI 10.1093/brain/awr066

Dubinsky RM, 2000, NEUROLOGY, V54, P2205, DOI 10.1212/WNL.54.12.2205

Fagan AM, 2006, ANN NEUROL, V59, P512, DOI 10.1002/ana.20730

Fjell AM, 2010, J NEUROSCI, V30, P2088, DOI 10.1523/JNEUROSCI.3785-09.2010

GROBER E, 1988, NEUROLOGY, V38, P900, DOI 10.1212/WNL.38.6.900

Gustafson DR, 2007, J NEUROL NEUROSUR PS, V78, P461, DOI 10.1136/jnnp.2006.100529

Hartley T, 2003, NEURON, V37, P877, DOI 10.1016/S0896-6273(03)00095-3

Head D, 2010, BEHAV BRAIN RES, V209, P49, DOI 10.1016/j.bbr.2010.01.012

Hedden T, 2013, NEUROLOGY, V80, P1341, DOI 10.1212/WNL.0b013e31828ab35d

HENDERSON VW, 1989, ARCH NEUROL-CHICAGO, V46, P391, DOI 10.1001/archneur.1989.00520400045018

HOLM S, 1979, SCAND J STAT, V6, P65

Hort J, 2007, P NATL ACAD SCI USA, V104, P4042, DOI 10.1073/pnas.0611314104

Hunt LA, 2010, AM J OCCUP THER, V64, P225, DOI 10.5014/ajot.64.2.225

Iaria G, 2003, J NEUROSCI, V23, P5945

Iaria G, 2007, EUR J NEUROSCI, V25, P890, DOI 10.1111/j.1460-9568.2007.05371.x

Iaria G, 2009, BEHAV BRAIN RES, V196, P187, DOI 10.1016/j.bbr.2008.08.040

Jack CR, 2013, LANCET NEUROL, V12, P207, DOI 10.1016/S1474-4422(12)70291-0

Kalova E, 2005, BEHAV BRAIN RES, V159, P175, DOI 10.1016/j.bbr.2004.10.016

Knopman DS, 2012, NEUROLOGY, V78, P1576, DOI 10.1212/WNL.0b013e3182563bbe

Laczo J, 2010, NEURODEGENER DIS, V7, P148, DOI 10.1159/000289226

Laczo J, 2011, NEURODEGENER DIS, V8, P169, DOI 10.1159/000321581

Lewis J, 2001, SCIENCE, V293, P1487, DOI 10.1126/science.1058189

Lithfous S, 2013, AGEING RES REV, V12, P201, DOI 10.1016/j.arr.2012.04.007

Ludbrook J, 1998, CLIN EXP PHARMACOL P, V25, P1032, DOI 10.1111/j.1440-1681.1998.tb02179.x

Madsen SK, 2010, NEUROBIOL AGING, V31, P1312, DOI 10.1016/j.neurobiolaging.2010.05.002

Maguire EA, 1998, SCIENCE, V280, P921, DOI 10.1126/science.280.5365.921

MCKHANN G, 1984, NEUROLOGY, V34, P939, DOI 10.1212/WNL.34.7.939

Minoshima S, 1997, ANN NEUROL, V42, P85, DOI 10.1002/ana.410420114

Moffat SD, 2009, NEUROPSYCHOL REV, V19, P478, DOI 10.1007/s11065-009-9120-3

Mormino EC, 2009, BRAIN, V132, P1310, DOI 10.1093/brain/awn320

Morris JC, 2001, ARCH NEUROL-CHICAGO, V58, P397, DOI 10.1001/archneur.58.3.397

MORRIS JC, 1993, NEUROLOGY, V43, P2412, DOI 10.1212/WNL.43.11.2412-a

Nedelska Z, 2012, P NATL ACAD SCI USA, V109, P2590, DOI 10.1073/pnas.1121588109

O'Keefe J., 1978, HIPPOCAMPUS COGNITIV

Ohnishi T, 2006, NEUROSCI RES, V55, P361, DOI 10.1016/j.neures.2006.04.009

Pai MC, 2004, INT J GERIATR PSYCH, V19, P250, DOI 10.1002/gps.1081

Pengas G, 2010, J ALZHEIMERS DIS, V21, P1347, DOI 10.3233/JAD-2010-100654

Pennanen C, 2004, NEUROBIOL AGING, V25, P303, DOI 10.1016/S0197-4580(03)00084-8

Petersen RC, 1999, ARCH NEUROL-CHICAGO, V56, P303, DOI 10.1001/archneur.56.3.303

Pike KE, 2007, BRAIN, V130, P2837, DOI 10.1093/brain/awm238

Price JL, 1999, ANN NEUROL, V45, P358, DOI 10.1002/1531-8249(199903)45:3<358::AID-ANA12>3.0.CO;2-X

Price JL, 2009, NEUROBIOL AGING, V30, P1026, DOI 10.1016/j.neurobiolaging.2009.04.002

Richardson AE, 1999, MEM COGNITION, V27, P741, DOI 10.3758/BF03211566

Roe CM, 2013, NEUROLOGY, V80, P1784, DOI 10.1212/WNL.0b013e3182918ca6

Selkoe DJ, 2000, JAMA-J AM MED ASSOC, V283, P1615, DOI 10.1001/jama.283.12.1615

Sheline YI, 2010, BIOL PSYCHIAT, V67, P584, DOI 10.1016/j.biopsych.2009.08.024

Shen L, 2010, BRAIN IMAGING BEHAV, V4, P86, DOI 10.1007/s11682-010-9088-x

Skoog I, 2003, DEMENT GERIATR COGN, V15, P169, DOI 10.1159/000068478

Sperling RA, 2011, ALZHEIMERS DEMENT, V7, P280, DOI 10.1016/j.jalz.2011.03.003

Stomrud E, 2007, DEMENT GERIATR COGN, V24, P118, DOI 10.1159/000105017

Storandt M, 2006, NEUROLOGY, V67, P467, DOI 10.1212/01.wnl.0000228231.26111.6e

Storandt M, 2009, ARCH NEUROL-CHICAGO, V66, P1476, DOI 10.1001/archneurol.2009.272

Strozyk D, 2003, NEUROLOGY, V60, P652, DOI 10.1212/01.WNL.0000046581.81650.D0

Thal DR, 2002, NEUROLOGY, V58, P1791, DOI 10.1212/WNL.58.12.1791

Tu MC, 2006, INT PSYCHOGERIATR, V18, P567, DOI 10.1017/S1041610206224025

Vos SJB, 2013, LANCET NEUROL, V12, P957, DOI 10.1016/S1474-4422(13)70194-7

Walhovd KB, 2010, AM J NEURORADIOL, V31, P347, DOI 10.3174/ajnr.A1809

Weniger G, 2011, NEUROPSYCHOLOGIA, V49, P518, DOI 10.1016/j.neuropsychologia.2010.12.031

Wolbers T, 2008, NAT NEUROSCI, V11, P1223, DOI 10.1038/nn.2189

ZWEIG MH, 1993, CLIN CHEM, V39, P561

NR 72

TC 76

Z9 78

U1 1

U2 44

PU IOS PRESS

PI AMSTERDAM

PA NIEUWE HEMWEG 6B, 1013 BG AMSTERDAM, NETHERLANDS

SN 1387-2877

EI 1875-8908

J9 J ALZHEIMERS DIS

JI J. Alzheimers Dis.

PY 2016

VL 52

IS 1

BP 77

EP 90

DI 10.3233/JAD-150855

PG 14

WC Neurosciences

WE Science Citation Index Expanded (SCI-EXPANDED); Social Science Citation Index (SSCI)

SC Neurosciences & Neurology

GA DK6CK

UT WOS:000375008500008

PM 26967209

OA Green Accepted

DA 2022-06-21

ER

PT J

AU AlMousa, M

Al-Khalifa, HS

AlSobayel, H

AF AlMousa, Maram

Al-Khalifa, Hend S.

AlSobayel, Hana

TI Requirements Elicitation and Prototyping of a Fully Immersive Virtual

Reality Gaming System for Upper Limb Stroke Rehabilitation in Saudi

Arabia

SO MOBILE INFORMATION SYSTEMS

LA English

DT Article

AB Stroke rehabilitation plays an important role in recovering the lifestyle of stroke survivors. Although existing research proved the effectiveness and engagement of nonimmersive virtual reality- (VR-) based rehabilitation systems, limited research is available on the applicability of fully immersive VR-based rehabilitation systems. In this paper, we present the elicited requirements of a fully immersive VR-based rehabilitation system that will be designed for domestic upper limb stroke patients; we will also provide an initial conceptual prototype of the proposed system.

C1 [AlMousa, Maram; Al-Khalifa, Hend S.] King Saud Univ, Informat Technol Dept, Coll Comp & Informat Sci, Riyadh, Saudi Arabia.

[AlSobayel, Hana] King Saud Univ, Dept Rehabil Sci, Coll Appl Med Sci, Riyadh, Saudi Arabia.

RP Al-Khalifa, HS (通讯作者)，King Saud Univ, Informat Technol Dept, Coll Comp & Informat Sci, Riyadh, Saudi Arabia.

EM hendk@ksu.edu.sa

RI Alsobayel, Hana I./AAE-8926-2022; Al-Khalifa, Hend S./F-3945-2011;

Al-Khalifa, Hend/AAY-5219-2020; Alsobayel, Hana/AGU-6834-2022

OI Alsobayel, Hana I./0000-0002-8366-5555; Al-Khalifa, Hend

S./0000-0002-7328-4935; Al-Khalifa, Hend/0000-0002-7328-4935;

FU "Research Center of the Female Scientific and Medical Colleges",

Deanship of Scientific Research, King Saud University

FX This research project was supported by a grant from the "Research Center

of the Female Scientific and Medical Colleges", Deanship of Scientific

Research, King Saud University.

CR Alhazani, 100 STROKE CASES OCC

Alsinani F, 2017, 6000 STROKE CASES AC

[Anonymous], WORLD HLTH REP 2002

Bower KJ, 2015, J NEUROENG REHABIL, V12, DOI 10.1186/s12984-015-0057-x

Burke JW, 2009, PROCEEDINGS OF THE IEEE VIRTUAL WORLDS FOR SERIOUS APPLICATIONS, P103, DOI 10.1109/VS-GAMES.2009.17

Burke JW, 2009, VISUAL COMPUT, V25, P1085, DOI 10.1007/s00371-009-0387-4

Grimm F., 2016, FRONTIERS NEUROSCIEN, V10

Hung YX, 2016, MEDICINE, V95, DOI 10.1097/MD.0000000000003032

Kaminer Conor, 2014, P 16 INT ACM SIGACCE, P299

Khujah K, STROKE REHABILITATIO

Laver KE, 2011, COCHRANE DB SYST REV, DOI [10.1002/14651858.CD008349.pub2, 10.1002/14651858.CD008349.pub4, 10.1002/14651858.CD008349.pub3]

Loureiro B., 2014, P ACHI, P41

Ma M, 2008, IEEE SYS MAN CYBERN, P1871

Oujamaa L., 2009, Annals of Physical and Rehabilitation Medicine, V52, P269, DOI 10.1016/j.rehab.2008.10.003

Rand D., P 5 INT C DIS VIRT E, P87

Rego P, 2010, SISTEMAS Y TECNOLOGIAS DE INFORMACION, P349

Saposnik G, 2010, STROKE, V41, P1477, DOI 10.1161/STROKEAHA.110.584979

Sen S. L., 2015, ASIA CONTROL CONF AS

Shin JH, 2014, J NEUROENG REHABIL, V11, DOI 10.1186/1743-0003-11-32

Standen P, 2015, PHYS THER, V95, P350, DOI 10.2522/ptj.20130564

Tsoupikova D, 2015, ANN BIOMED ENG, V43, P467, DOI 10.1007/s10439-014-1218-y

NR 21

TC 8

Z9 8

U1 8

U2 27

PU HINDAWI LTD

PI LONDON

PA ADAM HOUSE, 3RD FLR, 1 FITZROY SQ, LONDON, W1T 5HF, ENGLAND

SN 1574-017X

EI 1875-905X

J9 MOB INF SYST

JI Mob. Inf. Syst.

PY 2017

VL 2017

AR 7507940

DI 10.1155/2017/7507940

PG 12

WC Computer Science, Information Systems; Telecommunications

WE Science Citation Index Expanded (SCI-EXPANDED)

SC Computer Science; Telecommunications

GA EX4ZA

UT WOS:000403243600001

OA gold, Green Submitted

DA 2022-06-21

ER

PT J

AU Alneyadi, M

Drissi, N

Almeqbaali, M

Ouhbi, S

AF Alneyadi, Mahra

Drissi, Nidal

Almeqbaali, Mariam

Ouhbi, Sofia

TI Biofeedback-Based Connected Mental Health Interventions for Anxiety:

Systematic Literature Review

SO JMIR MHEALTH AND UHEALTH

LA English

DT Review

DE anxiety; biofeedback; systematic literature review; mental health;

eHealth; mHealth; connected health; digital health

ID VIRTUAL-REALITY EXPOSURE; EMOTION-REGULATION; GAMES; RELAXATION;

DISORDERS; STRESS; PAIN; INFORMATION; CHILDREN; THERAPY

AB Background: Connected mental health, which refers to the use of technology for mental health care and technology-based therapeutic solutions, has become an established field of research. Biofeedback is one of the approaches used in connected mental health solutions, which is mainly based on the analysis of physiological indicators for the assessment and management of the psychological state. Biofeedback is recommended by many therapists and has been used for conditions including depression, insomnia, and anxiety. Anxiety is associated with several physiological symptoms, including muscle tension and breathing issues, which makes the inclusion of biofeedback useful for anxiety detection and management.

Objective: The aim of this study was to identify interventions using biofeedback as a part of their process for anxiety management and investigate their perceived effectiveness.

Methods: A systematic literature review of publications presenting empirically evaluated biofeedback-based interventions for anxiety was conducted. The systematic literature review was based on publications retrieved from IEEE Digital Library, PubMed, ScienceDirect, and Scopus. A preliminary selection of papers was identified, examined, and filtered to include only relevant publications. Studies in the final selection were classified and analyzed to extract the modalities of use of biofeedback in the identified interventions, the types of physiological data that were collected and analyzed and the sensors used to collect them. Processes and outcomes of the empirical evaluations were also extracted.

Results: After final selection, 13 publications presenting different interventions were investigated. The interventions addressed either primarily anxiety disorders or anxiety associated with health issues such as migraine, Parkinson disease, and rheumatology. Solutions combined biofeedback with other techniques including virtual reality, music therapy, games, and relaxation practices and used different sensors including cardiovascular belts, wrist sensors, or stretch sensors to collect physiological data such as heart rate, respiration indicators, and movement information. The interventions targeted different cohorts including children, students, and patients. Overall, outcomes from the empirical evaluations yielded positive results and emphasized the effectiveness of connected mental health solutions using biofeedback for anxiety; however, certain unfavorable outcomes, such as interventions not having an effect on anxiety and patients' preferring traditional therapy, were reported in studies addressing patients with specific physical health issues.

Conclusions: The use of biofeedback in connected mental health interventions for the treatment and management of anxiety allows better screening and understanding of both psychological and physiological patient information, as well as of the association between the two. The inclusion of biofeedback could improve the outcome of interventions and boost their effectiveness; however, when used with patients suffering from certain physical health issues, suitability investigations are needed.

C1 [Alneyadi, Mahra; Drissi, Nidal; Almeqbaali, Mariam; Ouhbi, Sofia] Coll Informat Technol, Dept Comp Sci & Software Engn, Abu Dhabi, U Arab Emirates.

RP Ouhbi, S (通讯作者)，United Arab Emirates Univ, Dept Comp Sci & Software Engn, Coll Informat Technol, Abu Dhabi 15551, U Arab Emirates.

EM sofia.ouhbi@uaeu.ac.ae

OI Ouhbi, Sofia/0000-0001-7614-9731; Drissi, Nidal/0000-0001-9667-5931

FU Abu Dhabi Research and Development Authority [AYIA19-001]; United Arab

Emirates University [31T131]

FX This work is part of the Abu Dhabi Young Investigator Award 2019

(AYIA19-001) awarded by the Abu Dhabi Research and Development Authority

and the Startup project (31T131) funded by the United Arab Emirates

University.

CR Andrews Anya, 2011, Virtual and Mixed Reality - Systems and Applications. Proceedings International Conference, Virtual and Mixed Reality 2011. Held as Part of HCI International 2011, P3, DOI 10.1007/978-3-642-22024-1_1

[Anonymous], 2018, GEN ANXIETY DISORDER

[Anonymous], 2018, ANXIETY DISORDERS

Barnes C, 2009, AUST NZ J PSYCHIAT, V43, P934, DOI 10.1080/00048670903179137

Botella C, 2007, CLIN PSYCHOL PSYCHOT, V14, P164, DOI 10.1002/cpp.524

Burdea, 2003, VIRTUAL REALITY TECH, DOI [10.1162/105474603322955950, DOI 10.1162/105474603322955950]

Burt J, 2020, NEUROREHAB NEURAL RE, V34, P82, DOI 10.1177/1545968319893303

Carroll D, 1998, J ADV NURS, V27, P476, DOI 10.1046/j.1365-2648.1998.00551.x

Connolly TM, 2012, COMPUT EDUC, V59, P661, DOI 10.1016/j.compedu.2012.03.004

Crouch R., 2014, OCCUPATIONAL THERAPY, P978

Dondlinger MJ, 2007, J APPL ED TECHNOLOGY, V4, P21

Drissi N, 16 ACS IEEE INT C CO, DOI [10.1109/aiccsa47632.2019.9035325, DOI 10.1109/AICCSA47632.2019.9035325]

Drissi N, 2020, PROCEEDINGS OF THE 13TH INTERNATIONAL JOINT CONFERENCE ON BIOMEDICAL ENGINEERING SYSTEMS AND TECHNOLOGIES, VOL 5: HEALTHINF, P593, DOI 10.5220/0009100805930600

Drissi N, 2020, J MED INTERNET RES, V22, DOI 10.2196/19950

Drissi N, 2020, INT J MED INFORM, V141, DOI 10.1016/j.ijmedinf.2020.104243

Ebert DD, 2015, PLOS ONE, V10, DOI 10.1371/journal.pone.0119895

Enzenhofer M, 2004, J MED INTERNET RES, V6, DOI 10.2196/jmir.6.2.e16

EPPLEY KR, 1989, J CLIN PSYCHOL, V45, P957, DOI 10.1002/1097-4679(198911)45:6<957::AID-JCLP2270450622>3.0.CO;2-Q

Fleming TM, 2017, FRONT PSYCHIATRY, V7, DOI 10.3389/fpsyt.2016.00215

Frank Dana L, 2010, Ment Health Fam Med, V7, P85

Freeman D, 2017, PSYCHOL MED, V47, P2393, DOI 10.1017/S003329171700040X

Gaggioli A, 2014, J MED INTERNET RES, V16, P54, DOI 10.2196/jmir.3235

Gorini Alessandra, 2008, Expert Rev Neurother, V8, P215, DOI 10.1586/14737175.8.2.215

Gough C, 2017, DISTRIBUTION VIDEO G

Gough C, 2020, SHARE ACTIVE MOBILE

Harris O, 2016, P 2016 CHI C EXTENDE, P1989, DOI [10.1145/2851581.2892452, DOI 10.1145/2851581.2892452]

Hung JW, 2016, PM&R, V8, P962, DOI 10.1016/j.pmrj.2016.02.009

Ihmig FR, 2020, PLOS ONE, V15, DOI 10.1371/journal.pone.0231517

Jercic P, 2019, ENTERTAIN COMPUT, V29, P75, DOI 10.1016/j.entcom.2019.01.001

Knox M, 2011, Ment Health Fam Med, V8, P195

Kotwas I, 2018, INT J PSYCHOPHYSIOL, V123, P103, DOI 10.1016/j.ijpsycho.2017.10.005

Krusemark EA, 2012, CHEMOSENS PERCEPT, V5, P37, DOI 10.1007/s12078-011-9111-7

Kuriakose S, 2017, IEEE T NEUR SYS REH, V25, P1180, DOI 10.1109/TNSRE.2016.2613879

Laver KE, 2017, COCHRANE DB SYST REV, DOI 10.1002/14651858.CD008349.pub4

Leahy A, 1998, J ROY COLL PHYS LOND, V32, P552

Li F, 2016, 2016 8TH INTERNATIONAL CONFERENCE ON INFORMATION TECHNOLOGY IN MEDICINE AND EDUCATION (ITME), P90, DOI [10.1109/ITME.2016.0029, 10.1109/ITME.2016.95]

Liberati Alessandro, 2009, J Clin Epidemiol, V62, pe1, DOI 10.1016/j.jclinepi.2009.06.006

Maples-Keller JL, 2017, HARVARD REV PSYCHIAT, V25, P103, DOI 10.1097/HRP.0000000000000138

Martin EI, 2009, PSYCHIAT CLIN N AM, V32, P549, DOI 10.1016/j.psc.2009.05.004

McKenna K, 2015, PSYCHOSOMATICS, V56, P381, DOI 10.1016/j.psym.2014.06.003

McLay RN, 2009, APPL PSYCHOPHYS BIOF, V34, P319, DOI 10.1007/s10484-009-9104-3

Michael D., 2006, SERIOUS GAMES GAMES

Nutt DJ, 2005, CNS SPECTRUMS, V10, P49, DOI 10.1017/S1092852900009901

Pallavicini F., 2009, J CYBER THER REHABIL, V2, P315, DOI DOI 10.3233/978-1-60750-561-7-39

Payne R., 2010, PAYNES HDB RELAXATIO

Powers MB, 2008, J ANXIETY DISORD, V22, P561, DOI 10.1016/j.janxdis.2007.04.006

Przeworski A, 2006, CLIN PSYCHOL-UK, V10, P43, DOI [10.1080/13284200500378779, DOI 10.1080/13284200500378779]

Pyati S, 2007, CNS DRUGS, V21, P185, DOI 10.2165/00023210-200721030-00002

QUIRK ME, 1989, RADIOLOGY, V170, P463, DOI 10.1148/radiology.170.2.2911670

Ratanasiripong P, 2015, NURS RES PRACT, V2015, DOI 10.1155/2015/160746

Ratanasiripong Paul, 2012, ISRN Nurs, V2012, P827972, DOI 10.5402/2012/827972

Ratanasiripong P, 2012, J COLL STUDENT DEV, V53, P742, DOI 10.1353/csd.2012.0070

Read JL, 2011, JAMA-J AM MED ASSOC, V305, P1704, DOI 10.1001/jama.2011.408

Reiner R, 2008, APPL PSYCHOPHYS BIOF, V33, P55, DOI 10.1007/s10484-007-9046-6

Robb SL, 2000, J MUSIC THER, V37, P2, DOI 10.1093/jmt/37.1.2

Rodriguez N, 2018, ADJUNCT PROCEEDINGS OF THE 2018 IEEE INTERNATIONAL SYMPOSIUM ON MIXED AND AUGMENTED REALITY (ISMAR), P370, DOI 10.1109/ISMAR-Adjunct.2018.00107

Rothbaum BO, 2001, J CLIN PSYCHIAT, V62, P617, DOI 10.4088/JCP.v62n0808

Rusy LM, 2000, PEDIATR CLIN N AM, V47, P589, DOI 10.1016/S0031-3955(05)70227-3

Scharff L, 2002, J PEDIATR PSYCHOL, V27, P109, DOI 10.1093/jpepsy/27.2.109

Schild J, 2018, 25TH 2018 IEEE CONFERENCE ON VIRTUAL REALITY AND 3D USER INTERFACES (VR), P775, DOI 10.1109/VR.2018.8446160

Schuurmans AAT, 2018, J PSYCHOPATHOL BEHAV, V40, P344, DOI 10.1007/s10862-017-9638-2

Slater M, 2016, FRONT ROBOT AI, V3, DOI 10.3389/frobt.2016.00074

Smith CA, 2018, COCHRANE DB SYST REV, DOI 10.1002/14651858.CD009514.pub2

Stapleton A., 2004, P AUSTR GAM DEV C ME, P2

Stark DPH, 2000, BRIT J CANCER, V83, P1261, DOI 10.1054/bjoc.2000.1405

Tozzi F, 2018, JMIR PEDIATR PARENT, V1, DOI 10.2196/pediatrics.7248

Vavra KL, 2011, ALBERTA SCI ED J, V41, P22

Venuturupalli R Swamy, 2019, ACR Open Rheumatol, V1, P667, DOI 10.1002/acr2.11092

Vitasari P, IEEE STUD C RES DEV, P551, DOI [10.1109/scored.2009.5442939, DOI 10.1109/SCORED.2009.5442939]

Yohannes AM, 2014, EUR RESPIR REV, V23, P345, DOI 10.1183/09059180.00007813

Zermatten A, 2010, DEPRESS ANXIETY, V27, P852, DOI 10.1002/da.20665

Zyda M, 2005, COMPUTER, V38, P25, DOI 10.1109/MC.2005.297

NR 72

TC 2

Z9 2

U1 9

U2 11

PU JMIR PUBLICATIONS, INC

PI TORONTO

PA 130 QUEENS QUAY E, STE 1102, TORONTO, ON M5A 0P6, CANADA

SN 2291-5222

J9 JMIR MHEALTH UHEALTH

JI JMIR mHealth uHealth

PD APR 22

PY 2021

VL 9

IS 4

AR e26038

DI 10.2196/26038

PG 13

WC Health Care Sciences & Services; Medical Informatics

WE Science Citation Index Expanded (SCI-EXPANDED); Social Science Citation Index (SSCI)

SC Health Care Sciences & Services; Medical Informatics

GA WI8DX

UT WOS:000708587300001

PM 33792548

OA gold, Green Published

DA 2022-06-21

ER

PT J

AU Aloyuni, S

Alharbi, R

Kashoo, F

Alqahtani, M

Alanazi, A

Alzhrani, M

Ahmad, M

AF Aloyuni, Saleh

Alharbi, Raed

Kashoo, Faizan

Alqahtani, Mazen

Alanazi, Ahmad

Alzhrani, Msaad

Ahmad, Mehrunnisha

TI Knowledge, Attitude, and Barriers to Telerehabilitation-Based Physical

Therapy Practice in Saudi Arabia

SO HEALTHCARE

LA English

DT Article

DE telerehabilitation; physical therapy; cross-sectional survey

ID CARE; REHABILITATION; STROKE; TELEMEDICINE; SYSTEMS

AB (1) Telerehabilitation (TR) is a part of telemedicine involved in providing rehabilitation services to people in remote locations. TR in physical therapy in the kingdom of Saudi Arabia is still in its infancy and its implementation may pose different challenges in the physical therapy settings. The purpose of this nation-wide survey is to explore physiotherapists (PTs) knowledge, attitudes, and barriers towards implementation of TR in physical therapy settings; (2) Methods: A 14 item questionnaire was developed and mailed to PTs working in hospitals and rehabilitation centers across 13 provinces in Saudi Arabia; (3) Results: 347 PTs responded. Results are as follows: 58.8% (n = 204) of PTs reported that they had sufficient knowledge about TR. About31.7% (n = 110) of PTs reported that their hospital and rehabilitation center had installed TR, yet only 19.9% (n = 69) utilized the TR facility. Image-based TR was more frequently used (n = 33) as compared to sensor-based TR (n = 29) and virtual reality TR (n = 10).The main barriers were technical issues and cost related to implement TR in physical therapy settings; and (4) Conclusions: There is a relatively high number of PTs with self-reported knowledge about TR, however facilities and usage were limited. The main barriers were technical issues, staff skills, and the high cost involved in the introduction of TR in the PT-based health care settings.

C1 [Aloyuni, Saleh; Alharbi, Raed] Majmaah Univ, Coll Appl Med Sci, Dept Publ Hlth, Al Majmaah 11952, Saudi Arabia.

[Kashoo, Faizan; Alqahtani, Mazen; Alanazi, Ahmad; Alzhrani, Msaad] Majmaah Univ, Coll Appl Med Sci, Dept Phys Therapy & Hlth Rehabil, Al Majmaah 11952, Saudi Arabia.

[Ahmad, Mehrunnisha] Majmaah Univ, Coll Appl Med Sci, Dept Nursing, Al Majmaah 11952, Saudi Arabia.

RP Kashoo, F (通讯作者)，Majmaah Univ, Coll Appl Med Sci, Dept Phys Therapy & Hlth Rehabil, Al Majmaah 11952, Saudi Arabia.

EM s.aloyuni@mu.edu.sa; r.abdullah@mu.edu.sa; f.kashoo@mu.edu.sa;

mm.alqahtani@mu.edu.sa; aalanazi@mu.edu.sa; m.alzhrani@mu.edu.sa;

m.ahmer@mu.edu.sa

RI Alanazi, Ahmad Dhahawi/AAM-8336-2020; Kashoo, Faizan/AAD-1748-2020

OI Alanazi, Ahmad Dhahawi/0000-0002-5604-1835; Kashoo,

Faizan/0000-0002-8272-674X

CR Al-Yousuf M, 2002, East Mediterr Health J, V8, P645

Alajmi D, 2013, STUD HEALTH TECHNOL, V190, P118, DOI 10.3233/978-1-61499-276-9-118

[Anonymous], REP WCPT INPTRA DIG

Burdea GC, 2003, METHOD INFORM MED, V42, P519

Burns RB, 1998, ASSIST TECHNOL, V10, P126, DOI 10.1080/10400435.1998.10131970

Dipietro L, 2008, IEEE T SYST MAN CY C, V38, P461, DOI 10.1109/TSMCC.2008.923862

Heuser A, 2007, IEEE T NEUR SYS REH, V15, P43, DOI 10.1109/TNSRE.2007.891393

Holden MK, 2005, PRESENCE-VIRTUAL AUG, V14, P214, DOI 10.1162/1054746053967058

Huijgen BCH, 2008, J TELEMED TELECARE, V14, P249, DOI 10.1258/jtt.2008.080104

Jha AK, 2010, JAMA-J AM MED ASSOC, V304, P1709, DOI 10.1001/jama.2010.1497

Johansson T, 2011, J TELEMED TELECARE, V17, P1, DOI 10.1258/jtt.2010.100105

Lange B, 2009, EUR J PHYS REHAB MED, V45, P143

Lange B, 2011, IEEE ENG MED BIO, P1831, DOI 10.1109/IEMBS.2011.6090521

Lathan C.E., 2001, P 2001 EC NSF WORKSH, P80

Martin M, 2005, BRIT J EDUC TECHNOL, V36, P397, DOI 10.1111/j.1467-8535.2005.00471.x

Mathie MJ, 2004, PHYSIOL MEAS, V25, pR1, DOI 10.1088/0967-3334/25/2/R01

McCue Michael, 2010, Phys Med Rehabil Clin N Am, V21, P195, DOI 10.1016/j.pmr.2009.07.005

Mufti MH, 2000, HEALTHCARE DEV STRAT

Omachonu V., 2010, INNOV J, V15, P1

Rosen MJ, 1999, NEUROREHABILITATION, V12, P11

Russell TG, 2007, J TELEMED TELECARE, V13, P217, DOI 10.1258/135763307781458886

Russell TG, 2011, J BONE JOINT SURG AM, V93A, P113, DOI 10.2106/JBJS.I.01375

Saleem JJ, 2009, INT J MED INFORM, V78, P618, DOI 10.1016/j.ijmedinf.2009.04.001

Schwamm LH, 2009, STROKE, V40, P2616, DOI 10.1161/STROKEAHA.109.192360

Tam Sing-Fai, 2003, Occup Ther Int, V10, P20, DOI 10.1002/oti.175

Waite MC, 2010, TELEMED J E-HEALTH, V16, P564, DOI 10.1089/tmj.2009.0161

Walston S, 2008, ANN SAUDI MED, V28, P243

WHO, 2020, COVID 19 OP GUID MAI

NR 28

TC 5

Z9 5

U1 0

U2 2

PU MDPI

PI BASEL

PA ST ALBAN-ANLAGE 66, CH-4052 BASEL, SWITZERLAND

EI 2227-9032

J9 HEALTHCARE-BASEL

JI Healthcare

PD DEC

PY 2020

VL 8

IS 4

AR 460

DI 10.3390/healthcare8040460

PG 10

WC Health Care Sciences & Services; Health Policy & Services

WE Science Citation Index Expanded (SCI-EXPANDED); Social Science Citation Index (SSCI)

SC Health Care Sciences & Services

GA PJ1BL

UT WOS:000601512500001

PM 33158298

OA Green Published, gold

DA 2022-06-21

ER

PT J

AU Alsofy, SZ

Stroop, R

Fusek, I

Sakellaropoulou, I

Lewitz, M

Nakamura, M

Ewelt, C

Fortmann, T

AF Alsofy, Samer Zawy

Stroop, Ralf

Fusek, Ivo

Sakellaropoulou, Ioanna

Lewitz, Marc

Nakamura, Makoto

Ewelt, Christian

Fortmann, Thomas

TI Early autologous cranioplasty: complications and identification of risk

factors using virtual reality visualisation technique

SO BRITISH JOURNAL OF NEUROSURGERY

LA English

DT Article

DE 3D reconstruction; acute brain swelling; bone flap resorption;

decompressive craniectomy; early autologous cranioplasty; virtual

reality visualisation

ID DECOMPRESSIVE CRANIECTOMY; BONE; EXPERIENCE; BRAIN; OUTCOMES; SIMULATION

AB Background: Cranioplasty (CP) of autologous bone flap after decompressive craniectomy (DC) is known to be associated with a high complication rate, particularly bone flap resorption (BFR). In a retrospective study, we used a novel virtual reality (VR) visualisation technique to identify and evaluate risk factors associated with CP. Method: Twenty-five patients underwent early autologous CP. All complications were recorded. Cranial computed tomography scans were visualised via the VR software to access the fitting accuracy of the bone flap (bone flap size, gap width at trepanation cutting edge, extent of osteoclastic extension). Results: An overall complication rate of 44% was seen, and BFR was the most common (36%). Only 'osteoclastic extension of trepanation' (p = .04) was a significant risk factor for BFR. The factors 'indication for DC' (p = .09) and 'size of bone flap' (p = .09) had a tendency towards influencing the rate of BFR, while 'age' (p = .68), 'time interval between DC and CP' (p = 1.00), and 'gap width' (p = .50) were not considered to influence the BFR rate. Conclusions: DC and subsequent CP is a complication-prone procedure. Therefore, it is relevant to identify and quantify probable risk factors for the most common complications, such as BFR. Here, we found that the extent of osteoclastic extension may impair the patient's healing process. Our investigation was made considerably easier by using the novel VR visualisation technique, which allows parallax free measurements of distances in 3D space.

C1 [Alsofy, Samer Zawy; Fusek, Ivo; Sakellaropoulou, Ioanna; Lewitz, Marc; Ewelt, Christian; Fortmann, Thomas] Westfalische Wilhelms Univ Munster, St Barbara Hosp, Dept Neurosurg, Acad Hosp, Hamm, Germany.

[Stroop, Ralf] Westfalische Wilhelms Univ Munster, St Barbara Hosp, Dept Stereotact Neurosurg, Acad Hosp, Hamm, Germany.

[Nakamura, Makoto] Witten Herdecke Univ, Acad Hosp Cologne Merheim, Dept Neurosurg, Witten, Germany.

[Alsofy, Samer Zawy; Stroop, Ralf] Witten Herdecke Univ, Fac Hlth, Dept Med, Witten, Germany.

RP Stroop, R (通讯作者)，St Barbara Hosp, Dept Stereotact Neurosurg, D-59073 Hamm, Germany.

EM ralf@stroop.de

OI Stroop, Ralf/0000-0001-8795-6790

CR Agner C, 2002, ACTA NEUROCHIR, V144, P1033, DOI 10.1007/s00701-002-0996-4

Andrabi Syed M, 2017, Surg Neurol Int, V8, P91, DOI 10.4103/sni.sni_45_17

Basheer N, 2010, INDIAN J NEUROTRAUM, V7, P139, DOI 10.1016/S0973-0508(10)80029-2

Beauchamp KM, 2010, J TRAUMA, V69, P270, DOI 10.1097/TA.0b013e3181e491c2

Bobinski L, 2013, CLIN NEUROL NEUROSUR, V115, P1788, DOI 10.1016/j.clineuro.2013.04.013

Brommeland T, 2015, SCAND J TRAUMA RESUS, V23, DOI 10.1186/s13049-015-0155-6

Chang V, 2010, J NEUROSURG, V112, P1120, DOI 10.3171/2009.6.JNS09133

Chun HJ, 2011, J CRANIOFAC SURG, V22, P203, DOI 10.1097/SCS.0b013e3181f753bd

CITARDI MJ, 1994, OTOLARYNG CLIN N AM, V27, P891

Coulter IC, 2014, ACTA NEUROCHIR, V156, P1361, DOI 10.1007/s00701-014-2081-1

De Bonis P, 2012, J NEUROTRAUM, V29, P1071, DOI 10.1089/neu.2011.2116

Diedler Jennifer, 2009, J Intensive Care Med, V24, P168, DOI 10.1177/0885066609332808

Ernst G, 2018, J NEUROSURG, V129, P1604, DOI 10.3171/2017.6.JNS17943

Fiorot JA, 2008, ARQ NEURO-PSIQUIAT, V66, P204, DOI 10.1590/S0004-282X2008000200012

Fisher RA, 1922, J R STAT SOC, V85, P87, DOI 10.2307/2340521

FODSTAD H, 1984, ACTA NEUROCHIR, V70, P21, DOI 10.1007/BF01406039

Grant GA, 2004, J NEUROSURG, V100, P163, DOI 10.3171/ped.2004.100.2.0163

Iwama T, 2003, NEUROSURGERY, V52, P591, DOI 10.1227/01.NEU.0000047891.86938.46

Kim Ji Sang, 2015, Korean J Neurotrauma, V11, P1, DOI 10.13004/kjnt.2015.11.1.1

Kim Sung Hoon, 2017, Korean J Neurotrauma, V13, P15, DOI 10.13004/kjnt.2017.13.1.15

Kin T, 2017, NEUROL MED-CHIR, V57, P513, DOI 10.2176/nmc.ra.2016-0320

Konakondla S, 2017, ADV MED EDUC PRACT, V8, P465, DOI 10.2147/AMEP.S113565

Lawton MT, 2005, NEUROSURGERY, V57, P9, DOI 10.1227/01.NEU.0000163082.20941.EF

Lee L, 2013, BRIT J NEUROSURG, V27, P629, DOI 10.3109/02688697.2013.815313

Lee S. H., 2014, J NEUROTRAUM, V10, P10, DOI [1410.13004/kjnt.2014.10.1.1027169026, DOI 10.13004/kjnt.2014.10.1.10]

LEVIN DN, 1989, RADIOLOGY, V171, P277, DOI 10.1148/radiology.171.1.2928539

Moreira-Gonzalez A, 2003, J CRANIOFAC SURG, V14, P144, DOI 10.1097/00001665-200303000-00003

Murthy JMK, 2005, NEUROCRIT CARE, V2, P258, DOI 10.1385/NCC:2:3:258

Ong Chin Siang, 2018, J Vasc Interv Neurol, V10, P17

Otani N, 2008, CEREBROVASC DIS, V26, P612, DOI 10.1159/000165115

Piedra Mark P, 2014, Surg Neurol Int, V5, P25, DOI 10.4103/2152-7806.127762

Qiu WS, 2009, CRIT CARE, V13, DOI 10.1186/cc8178

Schuss P, 2013, J NEUROTRAUM, V30, P91, DOI 10.1089/neu.2012.2542

Sundseth J, 2014, ACTA NEUROCHIR, V156, P805, DOI 10.1007/s00701-013-1992-6

Tory M, 2006, IEEE T VIS COMPUT GR, V12, P2, DOI 10.1109/TVCG.2006.17

Vahedi K, 2007, LANCET NEUROL, V6, P215, DOI 10.1016/S1474-4422(07)70036-4

Walcott BP, 2013, J NEUROSURG, V118, P757, DOI 10.3171/2013.1.JNS121626

Ward G. W, 2008, GROVE ENCY MAT TECHN

Zhao Z, 2007, NEUROCRIT CARE, V7, P263, DOI 10.1007/s12028-007-0074-0

NR 39

TC 2

Z9 2

U1 0

U2 0

PU TAYLOR & FRANCIS LTD

PI ABINGDON

PA 2-4 PARK SQUARE, MILTON PARK, ABINGDON OR14 4RN, OXON, ENGLAND

SN 0268-8697

EI 1360-046X

J9 BRIT J NEUROSURG

JI Br. J. Neurosurg.

PD NOV 2

PY 2019

VL 33

IS 6

BP 664

EP 670

DI 10.1080/02688697.2019.1661962

EA SEP 2019

PG 7

WC Clinical Neurology; Surgery

WE Science Citation Index Expanded (SCI-EXPANDED)

SC Neurosciences & Neurology; Surgery

GA KC6GT

UT WOS:000486642700001

PM 31514550

DA 2022-06-21

ER

PT J

AU Alves, J

Vourvopoulos, A

Bernardino, A

Badia, SBI

AF Alves, J.

Vourvopoulos, A.

Bernardino, A.

Bermudez i Badia, S.

TI Eye Gaze Correlates of Motor Impairment in VR Observation of Motor

Actions

SO METHODS OF INFORMATION IN MEDICINE

LA English

DT Article

DE Action observation; eye movements; stroke; virtual reality

ID MIRROR NEURON SYSTEM; RECOVERY; IMAGERY

AB Introduction: This article is part of the Focus Theme of Methods of Information in Medicine on "Methodologies, Models and Algorithms for Patients Rehabilitation".

Objective: Identify eye gaze correlates of motor impairment in a virtual reality motor observation task in a study with healthy participants and stroke patients.

Methods: Participants consisted of a group of healthy subjects (N = 20) and a group of stroke survivors (N = 10). Both groups were required to observe a simple reach-and-grab and place-and-release task in a virtual environment. Additionally, healthy subjects were required to observe the task in a normal condition and a constrained movement condition. Eye movements were recorded during the observation task for later analysis.

Results: For healthy participants, results showed differences in gaze metrics when comparing the normal and arm-constrained conditions. Differences in gaze metrics were also found when comparing dominant and non-dominant arm for saccades and smooth pursuit events. For stroke patients, results showed longer smooth pursuit segments in action observation when observing the paretic arm, thus providing evidence that the affected circuitry may be activated for eye gaze control during observation of the simulated motor action.

Conclusions: This study suggests that neural motor circuits are involved, at multiple levels, in observation of motor actions displayed in a virtual reality environment. Thus, eye tracking combined with action observation tasks in a virtual reality display can be used to monitor motor deficits derived from stroke, and consequently can also be used for re habilitation of stroke patients.

C1 [Alves, J.; Vourvopoulos, A.; Bermudez i Badia, S.] Univ Madeira, Madeira Interact Technol Inst, Funchal, Portugal.

[Bernardino, A.] Inst Super Tecn, Inst Sistemas & Robot, Lisbon, Portugal.

RP Alves, J (通讯作者)，Polo Cient & Tecnol Madeira, Madeira ITI, Floor 2,Caminho Penteada, P-9020105 Funchal, Portugal.

EM juliomalves@gmail.com

RI Vourvopoulos, Athanasios/F-3872-2017; Badia, Sergi Bermúdez

i/C-8681-2018; Bernardino, Alexandre/G-1316-2010

OI Vourvopoulos, Athanasios/0000-0001-9676-8599; Badia, Sergi Bermúdez

i/0000-0003-4452-0414; Bernardino, Alexandre/0000-0003-3991-1269

FU European Commission through the RehabNet project - Neuroscience Based

Interactive Systems for Motor Rehabilitation - EC [303891 RehabNet

FP7-PEOPLE-2011-CIG]; Fundacao para Ciencia e Tecnologia (Portuguese

Foundation for Science and Technology) through the project AHA-Augmented

Human Assistance [CMUP-ERI/HCI/0046/2013, SFRH/BD/97117/2013]; Projeto

Estrategico [LA 9-2013-2014]

FX This work is supported by the European Commission through the RehabNet

project - Neuroscience Based Interactive Systems for Motor

Rehabilitation - EC (303891 RehabNet FP7-PEOPLE-2011-CIG), and by the

Fundacao para Ciencia e Tecnologia (Portuguese Foundation for Science

and Technology) through the project AHA-Augmented Human Assistance,

CMUP-ERI/HCI/0046/2013, doctoral grant SFRH/BD/97117/2013, and Projeto

Estrategico - LA 9-2013-2014.

CR Alves J, 2014, P 2 PAT REH RES TECH

Amantis R, 2011, ASSIST TECHNOL RES S, V29, P489, DOI 10.3233/978-1-60750-814-4-489

Bonaiuto J, 2007, BIOL CYBERN, V96, P9, DOI 10.1007/s00422-006-0110-8

Brouwer AM, 2009, J VISION, V9, DOI 10.1167/9.1.18

Buccino G, 2004, BRAIN LANG, V89, P370, DOI 10.1016/S0093-934X(03)00356-0

Causer J, 2013, FRONT HUM NEUROSCI, V7, DOI 10.3389/fnhum.2013.00604

Ertelt D, 2007, NEUROIMAGE, V36, pT164, DOI 10.1016/j.neuroimage.2007.03.043

Grezes J, 2001, HUM BRAIN MAPP, V12, P1, DOI 10.1002/1097-0193(200101)12:1<1::AID-HBM10>3.0.CO;2-V

Hermann DM, 2012, LANCET NEUROL, V11, P369, DOI 10.1016/S1474-4422(12)70039-X

Holmes P. S, 2010, NEUROPHYSIOLOGICAL F, P245

Liversedge SP, 2000, TRENDS COGN SCI, V4, P6, DOI 10.1016/S1364-6613(99)01418-7

Loconsole C., 2011, 2011 IEEE World Haptics Conference (WHC 2011), P185, DOI 10.1109/WHC.2011.5945483

Mukherjee D, 2011, WORLD NEUROSURG, V76, pS85, DOI 10.1016/j.wneu.2011.07.023

Mulder T, 2007, J NEURAL TRANSM, V114, P1265, DOI 10.1007/s00702-007-0763-z

Oztop E, 2002, BIOL CYBERN, V87, P116, DOI 10.1007/s00422-002-0318-1

Oztop E, 2013, NEUROSCI LETT, V540, P43, DOI 10.1016/j.neulet.2012.10.005

Rizzolatti G, 2004, ANNU REV NEUROSCI, V27, P169, DOI 10.1146/annurev.neuro.27.070203.144230

Rizzolatti G, 2010, NAT REV NEUROSCI, V11, P264, DOI 10.1038/nrn2805

Seitz RJ, 2010, J MAGN RESON IMAGING, V32, P756, DOI 10.1002/jmri.22315

Thill S, 2013, NEUROSCI BIOBEHAV R, V37, P491, DOI 10.1016/j.neubiorev.2013.01.012

Vourvopoulos Athanasios, 2013, 2013 IEEE 15th International Conference on e-Health Networking, Applications and Services (Healthcom 2013), P454, DOI 10.1109/HealthCom.2013.6720719

NR 21

TC 5

Z9 5

U1 0

U2 20

PU GEORG THIEME VERLAG KG

PI STUTTGART

PA RUDIGERSTR 14, D-70469 STUTTGART, GERMANY

SN 0026-1270

EI 2511-705X

J9 METHOD INFORM MED

JI Methods Inf. Med.

PY 2016

VL 55

IS 1

BP 79

EP 83

DI 10.3414/ME14-01-0125

PG 5

WC Computer Science, Information Systems; Health Care Sciences & Services;

Medical Informatics

WE Science Citation Index Expanded (SCI-EXPANDED)

SC Computer Science; Health Care Sciences & Services; Medical Informatics

GA DA5FC

UT WOS:000367827600011

PM 26640834

OA Green Submitted

DA 2022-06-21

ER

PT J

AU Alves, T

Goncalves, RS

Carbone, G

AF Alves, Thiago

Goncalves, Rogerio Sales

Carbone, Giuseppe

TI Quantitative Progress Evaluation of Post-stroke Patients Using a Novel

Bimanual Cable-driven Robot

SO JOURNAL OF BIONIC ENGINEERING

LA English

DT Article

DE Cable-driven robots; Low cost; Stroke rehabilitation; Bimanual; Progress

tracking; Serious games

ID UPPER-LIMB; REHABILITATION; DESIGN; DEVICE

AB Rehabilitation is the most effective way to reduce motor impairments in post-stroke patients. This process demands several hours with a specialized therapist. Given resources and personnel shortages, the literature reports a high interest in robotic assisted rehabilitation solutions. Recently, cable-driven robotic architectures are attracting significant research interest for post-stroke rehabilitation. However, the existing cable-driven robots are mostly unilateral devices allowing the rehabilitation only of the most affected limb. This leaves unaddressed the rehabilitation of bimanual activities, which are predominant within the common Activities of Daily Living (ADL). Thus, this paper presents a specific novel design to achieve bimanual rehabilitation tasks that has been named as BiCAR robot. Specifically, this paper provides a full insight on the BiCAR system as well as on its dedicated developed software BiEval. In particular, BiEval software has been developed as based on a serious game strategy and a virtual reality environment to track the patient exercising duration, motion ranges, speeds, and forces over time for achieving a quantitative assessment of the rehabilitation progress. Finally, the paper presents the BiCAR/BiEval capabilities by referring to a pilot Randomized Controlled Trial (RCT). The clinical trials have been used to validate the BiCAR/BiEval in terms of engineering feasibility and user acceptance to achieve an innovative cost-oriented integrated hardware/software device for the bimanual assistive rehabilitation of post-stroke patients.

C1 [Alves, Thiago; Goncalves, Rogerio Sales] Univ Fed Uberlandia, Sch Mech Engn, Lab Automat & Robot, BR-38400902 Uberlandia, MG, Brazil.

[Carbone, Giuseppe] Univ Calabria, Dept Mech Energy & Management Engn, I-87036 Arcavacata Di Rende, Italy.

RP Alves, T (通讯作者)，Univ Fed Uberlandia, Sch Mech Engn, Lab Automat & Robot, BR-38400902 Uberlandia, MG, Brazil.

EM thiago.alves1@ufu.br

RI Carbone, Giuseppe/J-5846-2012

OI Carbone, Giuseppe/0000-0003-0831-8358

FU UFU; FAPEMIG; CNPQ; CAPES [001]

FX This project was partially funded by UFU, FAPEMIG, CNPQ, and CAPES

(Finance Code 001).

CR Amine LM, 2019, J BIONIC ENG, V16, P503, DOI 10.1007/s42235-019-0041-4

Blanding DL., 1992, PRINCIPLES EXACT CON

Boschetti G, 2019, ROBOTICS, V8, DOI 10.3390/robotics8010017

Cafolla D, 2019, J BIONIC ENG, V16, P492, DOI 10.1007/s42235-019-0040-5

Ceccarelli M, 2021, SENSORS-BASEL, V21, DOI 10.3390/s21155149

Ceccarelli M, 2010, J ZHEJIANG UNIV-SC A, V11, P231, DOI 10.1631/jzus.A1000027

Ceccarellli M, 2019, 25 ABCM INT C MECH E

Ching S. L, 2013, 2013 STUD C ENG SYST, P1

Costa M. V. O, 2018, FORCE CAPABILITY PLA

Garofalo G, 2019, IEEE INT CONF ROBOT, P6117, DOI 10.1109/ICRA.2019.8793529

Goncalves R.S., 2020, ADV COMPUT INTELLIGE, P52, DOI [10.4018/978-1-7998-1382-8.ch003, DOI 10.4018/978-1-7998-1382-8.CH003]

Goncalves R. S, 2011, REH ROB ICORR 2011 I

Hatem SM, 2016, FRONT HUM NEUROSCI, V10, DOI 10.3389/fnhum.2016.00442

Ibarra J. C. P, 2014, THESIS SAO PAULO U

IMI, 2021, INTRINSIC MOTIVATION

Johnson MJ, 2005, IEEE T NEUR SYS REH, V13, P335, DOI 10.1109/TNSRE.2005.850428

Lim WB, 2011, MECH MACH THEORY, V46, P1265, DOI 10.1016/j.mechmachtheory.2011.04.006

Mao Y, 2012, IEEE T ROBOT, V28, P922, DOI 10.1109/TRO.2012.2189496

Masiero S, 2007, ARCH PHYS MED REHAB, V88, P142, DOI 10.1016/j.apmr.2006.10.032

Masiero S, 2014, NEUROREHAB NEURAL RE, V28, P377, DOI 10.1177/1545968313513073

Masiero S, 2014, EXPERT REV MED DEVIC, V11, P187, DOI 10.1586/17434440.2014.882766

Masiero S, 2011, J REHABIL RES DEV, V48, P355, DOI 10.1682/JRRD.2010.04.0063

Mehrholz J, 2015, COCHRANE DB SYST REV, DOI 10.1002/14651858.CD006876.pub4

MING A, 1994, INT J JPN S PREC ENG, V28, P131

Nijenhuis SM, 2015, J NEUROENG REHABIL, V12, DOI 10.1186/s12984-015-0080-y

Oyman EL, 2022, ROBOTICA, V40, P1, DOI 10.1017/S0263574721000357

Pedro L. M, 2017, 24 ABCM INT C MECH E

Pedro L. M, 2017, 24 ABCM INT C MECH E

Prange GB, 2013, INT C REHAB ROBOT

Prange GB, 2015, NEUROREHAB NEURAL RE, V29, P174, DOI 10.1177/1545968314535985

Quaglia G, 2017, MECH MACHINE SCI ADV

Rosati G, 2017, MECH MACH SCI, V47, P551, DOI 10.1007/978-3-319-48375-7_59

Roy A, 2011, J REHABIL RES DEV, V48, P417, DOI 10.1682/JRRD.2010.04.0078

Russo M, 2020, MACHINES, V8, DOI 10.3390/machines8030048

Tappeiner L, 2018, MECH MACH SCI, V50, P174, DOI 10.1007/978-3-319-60867-9_20

Trlep M, 2011, ADV ROBOTICS, V25, P1949, DOI 10.1163/016918611X588853

Wang RB, 2021, ROBOTICA, V39, P2193, DOI 10.1017/S0263574721000266

Zhang SY, 2020, INT J ENV RES PUB HE, V17, DOI 10.3390/ijerph17041156

NR 38

TC 0

Z9 0

U1 7

U2 11

PU SPRINGER SINGAPORE PTE LTD

PI SINGAPORE

PA #04-01 CENCON I, 1 TANNERY RD, SINGAPORE 347719, SINGAPORE

SN 1672-6529

EI 2543-2141

J9 J BIONIC ENG

JI J. Bionic Eng.

PD NOV

PY 2021

VL 18

IS 6

BP 1331

EP 1343

DI 10.1007/s42235-021-00102-y

EA DEC 2021

PG 13

WC Engineering, Multidisciplinary; Materials Science, Biomaterials;

Robotics

WE Science Citation Index Expanded (SCI-EXPANDED)

SC Engineering; Materials Science; Robotics

GA XN9VK

UT WOS:000724611900001

DA 2022-06-21

ER

PT J

AU Amaefule, CO

Ludtke, S

Kirste, T

Teiper, SJ

AF Amaefule, Chimezie O.

Luedtke, Stefan

Kirste, Thomas

Teiper, Stefan J.

TI Effect of Spatial Disorientation in a Virtual Environment on Gait and

Vital Features in Patients with Dementia: Pilot Single-Blind Randomized

Control Trial

SO JMIR SERIOUS GAMES

LA English

DT Article

DE spatial disorientation; activity recognition; wayfinding; wearable

sensors; dementia; virtual reality; older adults

ID COGNITIVE IMPAIRMENT; ALZHEIMERS-DISEASE; ATTENTION; REALITY; MEMORY;

MILD; NAVIGATION; RESPONSES; TREADMILL; DEFICITS

AB Background: Orientation deficits are among the most devastating consequences of early dementia. Digital navigation devices could overcome these deficits if adaptable to the user's needs (ie, provide situation-aware, proactive navigation assistance). To fulfill this task, systems need to automatically detect spatial disorientation from sensors in real time. Ideally, this would require field studies consisting of real-world navigation. However, such field studies can be challenging and are not guaranteed to cover sufficient instances of disorientation due to the large variability of real-world settings and a lack of control over the environment.

Objective: Extending a foregoing field study, we aim to evaluate the feasibility of using a sophisticated virtual reality (VR) setup, which allows a more controlled observation of disorientation states and accompanying behavioral and physiological parameters in cognitively healthy older people and people with dementia.

Methods: In this feasibility study, we described the experimental design and pilot outcomes of an ongoing study aimed at investigating the effect of disorientation on gait and selected physiological features in a virtual laboratory. We transferred a real-world navigation task to a treadmill-based virtual system for gait analysis. Disorientation was induced by deliberately manipulating landmarks in the VR projection. Associated responses in motion behavior and physiological parameters were recorded by sensors. Primary outcomes were variations in motion and physiological parameters, frequency of disorientation, and questionnaire-derived usability estimates (immersion and perceived control of the gait system) for our population of interest. At this time, the included participants were 9 cognitively healthy older participants [5/9 women, 4/9 men; mean age 70 years, SD 4.40; Mini-Mental State Examination (MMSE) mean 29, SD 0.70) and 4 participants with dementia (2/4 women, 2/4 men; mean age 78 years, SD 2.30 years; MMSE mean 20.50, SD 7.54). Recruitment is ongoing, with the aim of including 30 cognitively healthy older participants and 20 participants with dementia.

Results: All 13 participants completed the experiment. Patients' route was adapted by shortening it relative to the original route. Average instances of disorientation were 21.40, 36.50, and 37.50 for the cognitively healthy older control, cognitively healthy older experimental participants, and participants with dementia, respectively. Questionnaire outcomes indicated that participants experienced adequate usability and immersion; 4.30 for presence, 3.73 for involvement, and 3.85 for realism of 7 possible points, indicating a good overall ability to cope with the experiment. Variations were also observed in motion and physiological parameters during instances of disorientation.

Conclusions: This study presents the first feasibility outcomes of a study investigating the viability of using a sophisticated VR setup, based on an earlier real-world navigation study, to study spatial disorientation among cognitively healthy older people and people with dementia. Preliminary outcomes give confidence to the notion that our setup can be used to assess motion and physiological markers of disorientation, even in people with cognitive decline.

Trial Registration: ClinicalTrials.gov; https://clinicaltrials.gov/ct2/show/NCT04134806

C1 [Amaefule, Chimezie O.; Teiper, Stefan J.] German Ctr Neurodegenerat Dis DZNE, Gehlsheimer Str 20, D-18147 Rostock, Germany.

[Luedtke, Stefan; Kirste, Thomas] Univ Rostock, Inst Visual & Analyt Comp, Rostock, Germany.

[Teiper, Stefan J.] Univ Med Rostock, Dept Psychosomat & Psychotherapeut Med, Rostock, Germany.

RP Amaefule, CO (通讯作者)，German Ctr Neurodegenerat Dis DZNE, Gehlsheimer Str 20, D-18147 Rostock, Germany.

EM chimezie.amaefule@dzne.de

OI Amaefule, Chimezie/0000-0001-9822-3648; Ludtke,

Stefan/0000-0002-1488-4236

FU DFG (German Research Foundation) [INST 264/137-1 FUGG]; European

Regional Development Fund (EFRE) [TBI-V-1-100-VBW-035]

FX The GRAIL was funded by the DFG (German Research Foundation), grant

number INST 264/137-1 FUGG. The study is also partly funded through the

European Regional Development Fund (EFRE), reference number

TBI-V-1-100-VBW-035. The funders had no role in study design, data

collection and analysis, decision to publish, or preparation of the

manuscript. We would like to thank the following people: Johann Bauer,

Cindy Rikert, and Marie-Luise Ewald for developing the VR environment

application; Anne Klostermann and Charlotte Hinz for conducting the

experiments with the pilot subjects; Jonas Topfer for annotating the

videos; and Deborah Sonnenberg for patient recruitment.

CR Allali G, 2016, EUR J NEUROL, V23, P527, DOI 10.1111/ene.12882

[Anonymous], 2006, P LREC

Bachis S, VALIDITY ECGMOVE R P

Behrens M, 2018, J GERONTOL A-BIOL, V73, P792, DOI 10.1093/gerona/glx210

Biffi E, 2018, FRONT PSYCHOL, V9, DOI 10.3389/fpsyg.2018.02530

Buracchio T, 2010, ARCH NEUROL-CHICAGO, V67, P980, DOI 10.1001/archneurol.2010.159

Burgess N, 2006, REV NEUROSCIENCE, V17, P239

Callisaya ML, 2011, AGE AGEING, V40, P481, DOI 10.1093/ageing/afr055

COHEN J, 1960, EDUC PSYCHOL MEAS, V20, P37, DOI 10.1177/001316446002000104

Cohen JA, 2019, HAND CLINIC, V167, P419, DOI 10.1016/B978-0-12-804766-8.00022-4

Craig M, 2016, NEUROBIOL AGING, V48, P143, DOI 10.1016/j.neurobiolaging.2016.08.007

Cushman LA, 2008, NEUROLOGY, V71, P888, DOI 10.1212/01.wnl.0000326262.67613.fe

Costa Raquel Quimas Molina da, 2018, Dement. neuropsychol., V12, P196, DOI 10.1590/1980-57642018dn12-020013

de Kruijff LGM, 2018, J ROY ARMY MED CORPS, V164, P322, DOI 10.1136/jramc-2017-000870

de Rooij IJM, 2019, TRIALS, V20, DOI 10.1186/s13063-018-3165-7

De Urturi Breton Z, 2012, IEEE 14 INT C E HLTH, DOI [10.1109/HealthCom.2012.6379430, DOI 10.1109/HEALTHCOM.2012.6379430]

Delahaye Marcel, 2015, BMC Psychol, V3, P22, DOI 10.1186/s40359-015-0080-5

Glass TA, 1999, BRIT MED J, V319, P478, DOI 10.1136/bmj.319.7208.478

Hartel S, 2011, EUR REV AGING PHYS A, V8, P109, DOI 10.1007/s11556-010-0074-5

Hughes TF, 2013, INT PSYCHOGERIATR, V25, P587, DOI 10.1017/S1041610212002086

Ijaz K, 2019, JMIR MENT HEALTH, V6, DOI 10.2196/13887

Iwata H, 1999, IEEE COMPUT GRAPH, V19, P30, DOI 10.1109/38.799737

Jayakody O, 2019, J ALZHEIMERS DIS, V71, pS5, DOI 10.3233/JAD-181157

Kapp D, ISOLATING EFFECTS EM

Kaye J, 2012, 2012 JUL 01 ALZH ASS, pP483, DOI [10.1016/j.jalz, DOI 10.1016/J.JALZ.2012.05.1308]

Kimoto A, 2017, PSYCHIAT RES, V257, P456, DOI 10.1016/j.psychres.2017.08.038

Kimura K, 2017, SCI REP-UK, V7, DOI 10.1038/s41598-017-18289-8

Kizony R, 2017, J VESTIBUL RES-EQUIL, V27, P39, DOI 10.3233/VES-170605

Kober SE, 2013, J NEUROENG REHABIL, V10, DOI 10.1186/1743-0003-10-17

McGuinness B, 2010, J NEUROL NEUROSUR PS, V81, P157, DOI 10.1136/jnnp.2008.164483

MENDEZ MF, 1990, J CLIN NEURO-OPHTHAL, V10, P62

Mirahadi SS, 2018, MIDDLE E J REHABIL H, V5, DOI [10.5812/mejrh.64738, DOI 10.5812/MEJRH.64738]

Moreno A, 2019, ALZH DEMENT-TRCI, V5, P834, DOI 10.1016/j.trci.2019.09.016

NEBES RD, 1989, CORTEX, V25, P305, DOI 10.1016/S0010-9452(89)80045-0

Nichols S, 2002, APPL ERGON, V33, P251, DOI 10.1016/S0003-6870(02)00020-0

Schaat S, 2020, GERONTOLOGY, V66, P85, DOI 10.1159/000500971

Schubert T, 2001, PRESENCE-VIRTUAL AUG, V10, P266, DOI 10.1162/105474601300343603

Silveri MC, 2007, J GERIATR PSYCH NEUR, V20, P67, DOI 10.1177/0891988706297469

Sloot LH, 2014, GAIT POSTURE, V39, P478, DOI 10.1016/j.gaitpost.2013.08.022

Souman JL, 2011, ACM T APPL PERCEPT, V8, DOI 10.1145/2043603.2043607

Tamura A, 2018, FRONT NEUROL, V9, DOI 10.3389/fneur.2018.01056

Teipel S, 2016, ALZHEIMERS DEMENT, V12, P695, DOI 10.1016/j.jalz.2015.11.003

Tichon J, 2006, CYBERPSYCHOL BEHAV, V9, P480, DOI 10.1089/cpb.2006.9.480

Tregillus S, 2017, PROCEEDINGS OF THE 2017 ACM SIGCHI CONFERENCE ON HUMAN FACTORS IN COMPUTING SYSTEMS (CHI'17), P4063, DOI 10.1145/3025453.3025521

Urbaniak GC., 2013, RES RANDOMIZER VERSI

van den Bogert AJ, 2013, MED BIOL ENG COMPUT, V51, P1069, DOI 10.1007/s11517-013-1076-z

Westmoreland D, 2007, AVIAT SPACE ENVIR MD, V78, P985, DOI 10.3357/ASEM.2010.2007

Wettstein M, 2015, J APPL GERONTOL, V34, P3, DOI 10.1177/0733464812459373

Yordanova K, 2017, J ALZHEIMERS DIS, V60, P1461, DOI 10.3233/JAD-170105

NR 49

TC 2

Z9 2

U1 4

U2 9

PU JMIR PUBLICATIONS, INC

PI TORONTO

PA 130 QUEENS QUAY E, STE 1102, TORONTO, ON M5A 0P6, CANADA

SN 2291-9279

J9 JMIR SERIOUS GAMES

JI JMIR Serious Games

PD JUL-SEP

PY 2020

VL 8

IS 3

AR e18455

DI 10.2196/18455

PG 14

WC Health Care Sciences & Services; Public, Environmental & Occupational

Health; Medical Informatics

WE Science Citation Index Expanded (SCI-EXPANDED); Social Science Citation Index (SSCI)

SC Health Care Sciences & Services; Public, Environmental & Occupational

Health; Medical Informatics

GA OH2JX

UT WOS:000582396700004

PM 33030436

OA gold, Green Published

DA 2022-06-21

ER

PT J

AU Amaya, A

Woolf, C

Devane, N

Galliers, J

Talbot, R

Wilson, S

Marshall, J

AF Amaya, Ana

Woolf, Celia

Devane, Niamh

Galliers, Julia

Talbot, Richard

Wilson, Stephanie

Marshall, Jane

TI Receiving aphasia intervention in a virtual environment: the

participants' perspective

SO APHASIOLOGY

LA English

DT Article

DE Aphasia; therapy; participant perspectives; virtual reality

ID PUBLIC-SPEAKING ANXIETY; QUALITATIVE RESEARCH; HEALTH-CARE; THERAPY;

DEPRESSION; PEOPLE; STROKE; RELIABILITY; VALIDITY; REALITY

AB Background: Digital technology is making an increasing contribution to aphasia therapy. However, applications of virtual reality are rare. EVA Park is a virtual island developed with and for people with aphasia. It is a multi-user environment, which enables people with aphasia to interact with support workers, therapists and each other. The first study to use EVA Park in aphasia rehabilitation demonstrated significant gains in functional communication. This article augments the findings of that study, by reporting results from qualitative interviews conducted with the 20 study participants.Aims: This study aimed to determine the views of participants about the intervention that they received in EVA Park, and the impacts of that intervention. Long-term retrospective views were also explored.Methods & Procedures: Participants took part in 1:1, semi-structured interviews two weeks before (entry) and two weeks after (exit) the intervention. Questions focussed on activities undertaken by participants, communication, changes since the stroke and uses of technology. Exit interviews additionally explored participants' views and experiences of EVA Park and any perceived impacts of the intervention. A subset of five participants was interviewed at least one year later, to explore long-term recollections of the EVA Park intervention and any perceived long-term impacts. Interview data were transcribed and subject to framework analysis.Outcomes & Results: The thematic framework comprised 10 parent themes and 33 sub-themes. Following affect, the largest single theme related to EVA Park, with 636 coded references. Comments were overwhelmingly positive. EVA Park intervention was strongly associated with fun and enjoyment. Participants particularly valued their relationship with the support workers who delivered the intervention. The virtual locations and activities in EVA Park were also appreciated, together with the contact with other participants. Perceived impacts related to communication, activity, computer use and confidence. Most (4) participants in the long-term interviews described maintained impacts.Conclusions: These interview results indicate that the first intervention delivered in EVA Park was highly acceptable to participants and perceived as beneficial. They augment the findings of our experimental study and suggest that EVA Park could be a valuable addition to the resources available to practising clinicians.

C1 [Amaya, Ana; Woolf, Celia; Devane, Niamh; Talbot, Richard; Marshall, Jane] City Univ London, Div Language & Commun Sci, Northampton Sq, London EC1V 0HB, England.

[Galliers, Julia; Wilson, Stephanie] City Univ London, Ctr Human Comp Interact Design, London, England.

RP Marshall, J (通讯作者)，City Univ London, Div Language & Commun Sci, Northampton Sq, London EC1V 0HB, England.

EM J.Marshall@city.ac.uk

RI Wilson, Stephanie/AAX-1579-2020

OI Wilson, Stephanie/0000-0001-6445-654X; Devane, Niamh/0000-0001-8448-1478

FU Stroke Association [TSA 2011/10]; Stroke Association [TSA2011/10]

Funding Source: researchfish

FX This work was supported by The Stroke Association [TSA 2011/10].

CR Anderson PL, 2005, DEPRESS ANXIETY, V22, P156, DOI 10.1002/da.20090

Babbitt EM, 2011, APHASIOLOGY, V25, P727, DOI 10.1080/02687038.2010.537347

Brady MC, 2016, COCHRANE DB SYST REV, DOI 10.1002/14651858.CD000425.pub4

Cherney LR, 2011, J COMMUN DISORD, V44, P493, DOI 10.1016/j.jcomdis.2011.04.002

Cruice M, 2006, APHASIOLOGY, V20, P1210, DOI 10.1080/02687030600790136

Department for Business Innovation & Skills, 2012, 2011 SKILLS LIF SURV

Fourie RJ, 2009, INT J LANG COMM DIS, V44, P979, DOI [10.3109/13682820802535285, 10.1080/13682820802535285]

Galliers J, 2017, ACM T ACCESS COMPUT, V10, DOI 10.1145/3134227

Gilbert RL, 2013, INT J DISABIL DEV ED, V60, P208, DOI 10.1080/1034912X.2013.812189

Hackett ML, 2014, INT J STROKE, V9, P1017, DOI 10.1111/ijs.12357

Hawthorne G, 2006, SOC INDIC RES, V77, P521, DOI 10.1007/s11205-005-7746-y

Holland A., 1999, CADL 2 COMMUNICATION

Ingles JL, 1999, ARCH PHYS MED REHAB, V80, P173, DOI 10.1016/S0003-9993(99)90116-8

Kauhanen ML, 2000, CEREBROVASC DIS, V10, P455, DOI 10.1159/000016107

Kelly H, 2016, APHASIOLOGY, V30, P133, DOI 10.1080/02687038.2015.1077926

Lawton M, 2016, ARCH PHYS MED REHAB, V97, P1979, DOI 10.1016/j.apmr.2016.03.031

Lee RM, 2001, J COUNS PSYCHOL, V48, P310

Lee S, 2011, BMJ OPEN, V1, DOI 10.1136/bmjopen-2011-000269

Lerdal A, 2009, J PAIN SYMPTOM MANAG, V38, P928, DOI 10.1016/j.jpainsymman.2009.04.028

Luck AM, 2007, APHASIOLOGY, V21, P208, DOI 10.1080/02687030601065470

Lynch EB, 2008, J REHABIL MED, V40, P518, DOI 10.2340/16501977-0203

Marshall J, 2016, PLOS ONE, V11, DOI 10.1371/journal.pone.0160381

Martin N., 2017, APHASIA RELATED NEUR

Menger F, 2016, APHASIOLOGY, V30, P112, DOI 10.1080/02687038.2015.1109050

Palmer R, 2013, INT J LANG COMM DIS, V48, P508, DOI 10.1111/1460-6984.12024

Parr S, 2007, APHASIOLOGY, V21, P98, DOI 10.1080/02687030600798337

Pope C, 2000, BMJ-BRIT MED J, V320, P114, DOI 10.1136/bmj.320.7227.114

Riley EA, 2017, COMMUN DISORD Q, V38, P143, DOI 10.1177/1525740116656330

Rose TA, 2011, INT J SPEECH-LANG PA, V13, P335, DOI 10.3109/17549507.2011.560396

Russell DW, 1996, J PERS ASSESS, V66, P20, DOI 10.1207/s15327752jpa6601_2

Simmons-Mackie N, 2013, APHASIOLOGY, V27, P1281, DOI 10.1080/02687038.2013.818098

Spencer L., 2014, QUALITATIVE RES PRAC, V2nd, P269

Swinburn K, 2004, COMPREHENSIVE APHASI

Tomkins B, 2013, APHASIOLOGY, V27, P972, DOI 10.1080/02687038.2013.811211

True G, 2010, APHASIOLOGY, V24, P1032, DOI 10.1080/02687030903249350

van Dijk MJ, 2016, CLIN REHABIL, V30, P795, DOI 10.1177/0269215515599665

Wade J, 2003, APHASIOLOGY, V17, P1031, DOI 10.1080/02687030344000373

Wallach HS, 2009, BEHAV MODIF, V33, P314, DOI 10.1177/0145445509331926

Wilson S, 2015, CODESIGN, V11, P21, DOI 10.1080/15710882.2014.997744

NR 39

TC 15

Z9 15

U1 6

U2 31

PU ROUTLEDGE JOURNALS, TAYLOR & FRANCIS LTD

PI ABINGDON

PA 2-4 PARK SQUARE, MILTON PARK, ABINGDON OX14 4RN, OXON, ENGLAND

SN 0268-7038

EI 1464-5041

J9 APHASIOLOGY

JI Aphasiology

PY 2018

VL 32

IS 5

BP 538

EP 558

DI 10.1080/02687038.2018.1431831

PG 21

WC Audiology & Speech-Language Pathology; Linguistics; Clinical Neurology;

Rehabilitation

WE Science Citation Index Expanded (SCI-EXPANDED); Social Science Citation Index (SSCI)

SC Audiology & Speech-Language Pathology; Linguistics; Neurosciences &

Neurology; Rehabilitation

GA FW5KS

UT WOS:000425356200003

OA Green Accepted

DA 2022-06-21

ER

PT J

AU Amini, A

Banitsas, K

AF Amini, Amin

Banitsas, Konstantinos

TI Using Kinect v2 to Control a Laser Visual Cue System to Improve the

Mobility during Freezing of Gait in Parkinson's Disease

SO JOURNAL OF HEALTHCARE ENGINEERING

LA English

DT Article

ID VIRTUAL-REALITY; STRIDE LENGTH; PEOPLE

AB Different auditory and visual cues have been proven to be very effective in improving the mobility of people with Parkinson's (PwP). Nonetheless, many of the available methods require user intervention and so on to activate the cues. Moreover, once activated, these systems would provide cues continuously regardless of the patient's needs. This research proposes a new indoor method for casting dynamic/automatic visual cues for PwP based on their head direction and location in a room. The proposed system controls the behavior of a set of pan/tilt servo motors and laser pointers, based on the real-time skeletal information acquired from a Kinect v2 sensor. This produces an automatically adjusting set of laser lines that can always be in front of the patient as a guideline for where the next footstep would be placed. A user interface was also created that enables users to control and adjust the settings based on the preferences. The aim of this research was to provide PwP with an unobtrusive/automatic indoor system for improving their mobility during a Freezing of gait (FOG) incident. The results showed the possibility of employing such system, which does not rely on the subject's input nor does it introduce any additional complexities to operate.

C1 [Amini, Amin; Banitsas, Konstantinos] Brunel Univ London, Dept Elect & Comp Engn, Coll Engn Design & Phys Sci, London, England.

RP Amini, A (通讯作者)，Brunel Univ London, Dept Elect & Comp Engn, Coll Engn Design & Phys Sci, London, England.

EM amin.amini@brunel.ac.uk; konstantinos.banitsas@brunel.ac.uk

OI Amini, Dr. Amin/0000-0001-7081-2440

CR Amini A, 2017, 2017 IEEE EMBS INTERNATIONAL CONFERENCE ON BIOMEDICAL & HEALTH INFORMATICS (BHI), P153, DOI 10.1109/BHI.2017.7897228

Amini A., 2013, P INT C EMB SOFTW, P1, DOI DOI 10.1109/MEMEA.2016.7533763

Amini A., 2018, THESIS

Amini A, 2019, DISABIL REHABIL-ASSI, V14, P566, DOI 10.1080/17483107.2018.1467975

Bigy AAM, 2015, I IEEE EMBS C NEUR E, P731, DOI 10.1109/NER.2015.7146727

Bloem BR, 2004, MOVEMENT DISORD, V19, P871, DOI 10.1002/mds.20115

Bunting-Perry L, 2013, J REHABIL RES DEV, V50, P223, DOI 10.1682/JRRD.2011.12.0255

Choi SM, 2019, NEUROL SCI, V40, P293, DOI 10.1007/s10072-018-3625-6

Corti A, 2016, ROBOT AUTON SYST, V75, P584, DOI 10.1016/j.robot.2015.09.024

Donovan S, 2011, PARKINSONISM RELAT D, V17, P240, DOI 10.1016/j.parkreldis.2010.08.010

Evans B., 2011, BEGINNING ARDUINO PR

Foroughi F., 2015, INT J COMPUTER APPL, V116, P1, DOI [10.5120/20433-2764, DOI 10.5120/20433-2764]

Gilat M, 2013, PLOS ONE, V8, DOI 10.1371/journal.pone.0066718

Ginis P, 2018, ANN PHYS REHABIL MED, V61, P407, DOI 10.1016/j.rehab.2017.08.002

Griffin HJ, 2011, J NEUROL, V258, P991, DOI 10.1007/s00415-010-5866-z

Kalia LV, 2015, LANCET, V386, P896, DOI 10.1016/S0140-6736(14)61393-3

Kaminsky TA, 2007, J REHABIL RES DEV, V44, P437, DOI 10.1682/JRRD.2006.09.0109

Lewis GN, 2000, BRAIN, V123, P2077, DOI 10.1093/brain/123.10.2077

Lo AC, 2010, J NEUROENG REHABIL, V7, DOI 10.1186/1743-0003-7-51

McAuley JH, 2009, CLIN REHABIL, V23, P687, DOI 10.1177/0269215509104170

Mirelman A, 2013, MOVEMENT DISORD, V28, P1597, DOI 10.1002/mds.25670

Nieuwboer A, 2008, MOVEMENT DISORD, V23, pS475, DOI 10.1002/mds.21978

Okuma Y, 2006, J NEUROL, V253, P27, DOI 10.1007/s00415-006-7007-2

Robles-Garcia V, 2016, PARKINSONISM RELAT D, V26, P17, DOI 10.1016/j.parkreldis.2016.02.022

Rocha PA, 2014, CLIN NEUROL NEUROSUR, V124, P127, DOI 10.1016/j.clineuro.2014.06.026

Severiano MIR, 2018, ARQ NEURO-PSIQUIAT, V76, P78, DOI [10.1590/0004-282X20170195, 10.1590/0004-282x20170195]

Swim K., 2010, EFFECT LASER GUIDED

Velik R, 2012, IEEE ENG MED BIO, P4656, DOI 10.1109/EMBC.2012.6347005

Yelshyna D, 2016, BEHAV BRAIN RES, V296, P384, DOI 10.1016/j.bbr.2015.08.017

Zhao Y, 2013, IEEE ENG MED BIO, P5895, DOI 10.1109/EMBC.2013.6610893

NR 30

TC 2

Z9 3

U1 0

U2 6

PU HINDAWI LTD

PI LONDON

PA ADAM HOUSE, 3RD FLR, 1 FITZROY SQ, LONDON, W1T 5HF, ENGLAND

SN 2040-2295

EI 2040-2309

J9 J HEALTHC ENG

JI J. Healthc. Eng.

PY 2019

VL 2019

AR 3845462

DI 10.1155/2019/3845462

PG 8

WC Health Care Sciences & Services

WE Science Citation Index Expanded (SCI-EXPANDED)

SC Health Care Sciences & Services

GA HN5TW

UT WOS:000460248900001

PM 30915207

OA Green Published, gold, Green Submitted

DA 2022-06-21

ER

PT J

AU Aminov, A

Rogers, JM

Middleton, S

Caeyenberghs, K

Wilson, PH

AF Aminov, Anna

Rogers, Jeffrey M.

Middleton, Sandy

Caeyenberghs, Karen

Wilson, Peter H.

TI What do randomized controlled trials say about virtual rehabilitation in

stroke? A systematic literature review and meta-analysis of upper-limb

and cognitive outcomes

SO JOURNAL OF NEUROENGINEERING AND REHABILITATION

LA English

DT Review

DE Cognition; Meta-analysis; Motor performance; Rehabilitation; Stroke;

Virtual reality

ID REALITY-BASED REHABILITATION; UPPER EXTREMITY FUNCTION; FILE-DRAWER

PROBLEM; ARM MOTOR RECOVERY; QUALITY-OF-LIFE; VIDEO-GAMES; INTERNATIONAL

CLASSIFICATION; FUNCTIONAL RECOVERY; VISUAL-PERCEPTION; SUBACUTE STROKE

AB Background: Virtual-reality based rehabilitation (VR) shows potential as an engaging and effective way to improve upper-limb function and cognitive abilities following a stroke. However, an updated synthesis of the literature is needed to capture growth in recent research and address gaps in our understanding of factors that may optimize training parameters and treatment effects.

Methods: Published randomized controlled trials comparing VR to conventional therapy were retrieved from seven electronic databases. Treatment effects (Hedge's g) were estimated using a random effects model, with motor and functional outcomes between different protocols compared at the Body Structure/Function, Activity, and Participation levels of the International Classification of Functioning.

Results: Thirty-three studies were identified, including 971 participants (492 VR participants). VR produced small to medium overall effects (g = 0.46; 95% CI: 0.33-0.59, p < 0.01), above and beyond conventional therapies. Small to medium effects were observed on Body Structure/Function (g = 0.41; 95% CI: 0.28-0.55; p < 0.01) and Activity outcomes (g = 0.47; 95% CI: 0.34-0.60, p < 0.01), while Participation outcomes failed to reach significance (g = 0.38; 95% CI: -0.29-1.04, p = 0.27). Superior benefits for Body Structure/Function (g = 0.56) and Activity outcomes (g = 0.62) were observed when examining outcomes only from purpose-designed VR systems. Preliminary results (k = 4) suggested small to medium effects for cognitive outcomes (g = 0.41; 95% CI: 0.28-0.55; p < 0.01). Moderator analysis found no advantage for higher doses of VR, massed practice training schedules, or greater time since injury.

Conclusion: VR can effect significant gains on Body Structure/Function and Activity level outcomes, including improvements in cognitive function, for individuals who have sustained a stroke. The evidence supports the use of VR as an adjunct for stroke rehabilitation, with effectiveness evident for a variety of platforms, training parameters, and stages of recovery.

C1 [Aminov, Anna; Middleton, Sandy; Wilson, Peter H.] Australian Catholic Univ, Sch Psychol, Fac Hlth Sci, Sydney, NSW, Australia.

[Rogers, Jeffrey M.] South Eastern Sydney Local Hlth Dist, Sydney, NSW, Australia.

[Caeyenberghs, Karen; Wilson, Peter H.] Australian Catholic Univ, Sch Psychol, Melbourne, Vic, Australia.

[Caeyenberghs, Karen; Wilson, Peter H.] Australian Catholic Univ, Ctr Disabil & Dev Res CeDDR, Melbourne, Vic, Australia.

RP Wilson, PH (通讯作者)，Australian Catholic Univ, Sch Psychol, Fac Hlth Sci, Sydney, NSW, Australia.; Wilson, PH (通讯作者)，Australian Catholic Univ, Sch Psychol, Melbourne, Vic, Australia.; Wilson, PH (通讯作者)，Australian Catholic Univ, Ctr Disabil & Dev Res CeDDR, Melbourne, Vic, Australia.

EM peterh.wilson@acu.edu.au

RI Middleton, Sandy/J-5526-2015; Wilson, Peter H./E-2881-2018

OI Middleton, Sandy/0000-0002-7201-4394; Wilson, Peter

H./0000-0003-3747-0287; Aminov, Anna/0000-0003-4805-5476; Caeyenberghs,

Karen/0000-0001-7009-6843; Rogers, Jeffrey M/0000-0002-0320-969X

FU Prince of Wales Hospital Foundation

FX This research is supported by a Prince of Wales Hospital Foundation

grant awarded to JR, and an Research Training Program award awarded to

AA.

CR Adams HP, 2007, STROKE, V38, P1655, DOI 10.1161/STROKEAHA.107.181486

Al-Khindi T, 2010, STROKE, V41, pE519, DOI 10.1161/STROKEAHA.110.581975

Alt Murphy M, 2015, BMC NEUROL, V15, DOI 10.1186/s12883-015-0292-6

Aminov A, 2017, INT J STROKE, V12, P18

Andersen KK, 2009, STROKE, V40, P2068, DOI 10.1161/STROKEAHA.108.540112

Bhalla A, 2013, STROKE, V44, P2174, DOI 10.1161/STROKEAHA.113.001263

Broeren J, 2008, CEREBROVASC DIS, V26, P289, DOI 10.1159/000149576

Chen C, 2013, NEUROLOGY, V80, pS27, DOI 10.1212/WNL.0b013e3182762569

Chen MH, 2015, CLIN REHABIL, V29, P674, DOI 10.1177/0269215514554115

Choi JH, 2014, ANN REHABIL MED-ARM, V38, P485, DOI 10.5535/arm.2014.38.4.485

Cicerone KD, 2011, ARCH PHYS MED REHAB, V92, P519, DOI 10.1016/j.apmr.2010.11.015

Claessen MHG, 2016, NEUROPSYCHOL REHABIL, V26, P822, DOI 10.1080/09602011.2015.1045910

Clare L, 2003, COCHRANE DB SYST REV, DOI [10.1002/14651858.CD003260., DOI 10.1002/14651858.CD003260]

Cohen J., 1988, Statistical Power Analysis for the Behavioral Sciences, V2nd

Crichton SL, 2016, J NEUROL NEUROSUR PS, V87, P1091, DOI 10.1136/jnnp-2016-313361

Crosbie JH, 2007, DISABIL REHABIL, V29, P1139, DOI 10.1080/09638280600960909

Crosbie JH, 2012, CLIN REHABIL, V26, P798, DOI 10.1177/0269215511434575

Cumming TB, 2013, ACTA NEUROL SCAND, V128, P122, DOI 10.1111/ane.12084

Cumming TB, 2013, INT J STROKE, V8, P38, DOI 10.1111/j.1747-4949.2012.00972.x

Cumming TB, 2011, STROKE, V42, P2642, DOI 10.1161/STROKEAHA.111.619486

Cameirao MDS, 2011, RESTOR NEUROL NEUROS, V29, P287, DOI 10.3233/RNN-2011-0599

Ribeiro NMD, 2015, TOP STROKE REHABIL, V22, P299, DOI 10.1179/1074935714Z.0000000017

de Assis GA, 2016, DISABIL REHABIL-ASSI, V11, P521, DOI 10.3109/17483107.2014.979330

Palma GCD, 2017, TOP STROKE REHABIL, V24, P269, DOI 10.1080/10749357.2016.1250373

Douiri A, 2013, STROKE, V44, P138, DOI 10.1161/STROKEAHA.112.670844

Duckworth Jonathan, 2013, Design, User Experience, and Usability. Health, Learning, Playing, Cultural, and Cross-Cultural User Experience.Second International Conference, DUXU 2013 Held as Part of HCI International 2013. Proceedings. LNCS 8013, P391, DOI 10.1007/978-3-642-39241-2_43

Duckworth J, 2015, LECT NOTES COMPUT SC, V9177, P420, DOI 10.1007/978-3-319-20684-4_41

Duff M, 2010, IEEE T NEUR SYS REH, V18, P531, DOI 10.1109/TNSRE.2010.2055061

Duncan F, 2012, INT J STROKE, V7, P157, DOI 10.1111/j.1747-4949.2011.00741.x

Edmans J, 2009, CLIN REHABIL, V23, P106, DOI 10.1177/0269215508095875

Edmans JA, 2006, STROKE, V37, P2770, DOI 10.1161/01.STR.0000245133.50935.65

Egger M, 1997, BMJ-BRIT MED J, V315, P629, DOI 10.1136/bmj.315.7109.629

Feigin VL, 2014, LANCET, V383, P245, DOI 10.1016/S0140-6736(13)61953-4

Fritz SL, 2013, TOP STROKE REHABIL, V20, P218, DOI 10.1310/tsr2003-218

Gamito P, 2017, DISABIL REHABIL, V39, P385, DOI 10.3109/09638288.2014.934925

Givon N, 2016, CLIN REHABIL, V30, P383, DOI 10.1177/0269215515584382

Green D, 2012, DISABIL REHABIL, V34, P593, DOI 10.3109/09638288.2011.613520

Hakkennes SJ, 2011, ARCH PHYS MED REHAB, V92, P2057, DOI 10.1016/j.apmr.2011.07.189

Henderson A, 2007, TOP STROKE REHABIL, V14, P52, DOI 10.1310/tsr1402-52

Hocine N, 2015, USER MODEL USER-ADAP, V25, P65, DOI 10.1007/s11257-015-9154-6

Housman SJ, 2009, NEUROREHAB NEURAL RE, V23, P505, DOI 10.1177/1545968308331148

Howick J, 2011, EXPLANATION 2011 OXF

Imms C, 2017, DEV MED CHILD NEUROL, V59, P16, DOI 10.1111/dmcn.13237

In TS, 2012, J PHYS THER SCI, V24, P339, DOI 10.1589/jpts.24.339

Jo K, 2012, J PHYS THER SCI, V24, P1205

Kalaria RN, 2016, BBA-MOL BASIS DIS, V1862, P915, DOI 10.1016/j.bbadis.2016.01.015

Kamper SJ, 2015, 15 YEARS TRACKING PH

Kang SH, 2009, CLIN REHABIL, V23, P434, DOI 10.1177/0269215508101732

Kim BR, 2011, ANN REHABIL MED-ARM, V35, P450, DOI 10.5535/arm.2011.35.4.450

Kim EK, 2012, J PHYS THER SCI, V24, P901, DOI 10.1589/jpts.24.901

Kiper P, 2014, BIOMED RES INT, V2014, DOI 10.1155/2014/752128

Kiper P, 2011, NEUROL NEUROCHIR POL, V45, P436

Kizony R, 2003, J VISUAL COMP ANIMAT, V14, P261, DOI 10.1002/vis.323

Kizony R, 2004, P 5 INT C DIS VIRT R

Kong KH, 2016, TOP STROKE REHABIL, V23, P333, DOI 10.1080/10749357.2016.1139796

Kottink AIR, 2014, GAMES HEALTH J, V3, P184, DOI 10.1089/g4h.2014.0026

Kwon JS, 2012, NEUROREHABILITATION, V31, P379, DOI 10.3233/NRE-2012-00807

Lampit A, 2014, PLOS MED, V11, DOI 10.1371/journal.pmed.1001756

Langhorne P, 2009, LANCET NEUROL, V8, P741, DOI 10.1016/S1474-4422(09)70150-4

Laver KE, 2011, COCHRANE DB SYST REV, DOI [10.1002/14651858.CD008349.pub2, 10.1002/14651858.CD008349.pub4, 10.1002/14651858.CD008349.pub3]

Lawrence ES, 2001, STROKE, V32, P1279, DOI 10.1161/01.STR.32.6.1279

Lee G, 2013, J PHYS THER SCI, V25, P595, DOI 10.1589/jpts.25.595

Lee SJ, 2014, ARCH PHYS MED REHAB, V95, P431, DOI 10.1016/j.apmr.2013.10.027

Lesniak M, 2008, DEMENT GERIATR COGN, V26, P356, DOI 10.1159/000162262

Levin MF, 2012, NEUROL THER, V1, DOI 10.1007/s40120-012-0003-9

Levin MF, 2011, EXPERT REV NEUROTHER, V11, P153, DOI [10.1586/ern.10.201, 10.1586/ERN.10.201]

Liberati A, 2009, BMJ-BRIT MED J, V339, DOI [10.1371/journal.pmed.1000100, 10.1136/bmj.b2700, 10.7326/0003-4819-151-4-200908180-00136]

Lincoln NB, 2000, COCHRANE DB SYST REV, DOI [10.1002/14651858,CD002842, DOI 10.1002/14651858.CD002842]

Lofgren B, 1999, CEREBROVASC DIS, V9, P163, DOI 10.1159/000015948

Lohse KR, 2014, PLOS ONE, V9, DOI 10.1371/journal.pone.0093318

Maher CG, 2003, PHYS THER, V83, P713, DOI 10.1093/ptj/83.8.713

Mercier C, 2004, CLIN REHABIL, V18, P215, DOI 10.1191/0269215504cr724oa

Meyer S, 2015, STROKE, V46, P1613, DOI 10.1161/STROKEAHA.115.009421

Modig S, 2011, BMC GERIATR, V11, DOI 10.1186/1471-2318-11-55

Moreira MC, 2013, DISABIL REHABIL-ASSI, V8, P357, DOI 10.3109/17483107.2012.749428

Moseley AM, 2002, AUST J PHYSIOTHER, V48, P43, DOI 10.1016/S0004-9514(14)60281-6

Mullick AA, 2015, RESTOR NEUROL NEUROS, V33, P389, DOI 10.3233/RNN-150510

Mumford N, 2012, BRAIN INJURY, V26, P166, DOI 10.3109/02699052.2011.648706

Mumford N, 2009, BRAIN INJURY, V23, P179, DOI 10.1080/02699050802695566

Mundy L, 2010, AUSTR NZ HORIZON SCA, P27

Muratori LM, 2013, J HAND THER, V26, P94, DOI 10.1016/j.jht.2012.12.007

Musicco M, 2003, ARCH PHYS MED REHAB, V84, P551, DOI 10.1053/apmr.2003.50084

Norouzi-Gheidari N, 2012, J REHABIL RES DEV, V49, P479, DOI 10.1682/JRRD.2010.10.0210

Nys GMS, 2007, CEREBROVASC DIS, V23, P408, DOI 10.1159/000101464

Patel M, 2003, CLIN REHABIL, V17, P158, DOI 10.1191/0269215503cr596oa

Patel MD, 2006, AGE AGEING, V35, P273, DOI 10.1093/ageing/afj074

Peek K, 2016, PHYSIOTHERAPY, V102, P127, DOI 10.1016/j.physio.2015.10.003

Piron L, 2010, NEUROREHAB NEURAL RE, V24, P501, DOI 10.1177/1545968310362672

Piron L, 2009, J REHABIL MED, V41, P1016, DOI 10.2340/16501977-0459

Pollock A, 2014, STROKE, V45, pE202, DOI 10.1161/STROKEAHA.114.006275

Rizzo A, 2005, PRESENCE-TELEOP VIRT, V14, P119, DOI 10.1162/1054746053967094

Rizzo AA, 2004, NEUROPSYCHOL REHABIL, V14, P207, DOI 10.1080/09602010343000183

Rose FD, 2005, CYBERPSYCHOL BEHAV, V8, P241, DOI 10.1089/cpb.2005.8.241

Rosenberg MS, 2005, EVOLUTION, V59, P464, DOI 10.1111/j.0014-3820.2005.tb01004.x

ROSENTHAL R, 1979, PSYCHOL BULL, V86, P638, DOI 10.1037/0033-2909.86.3.638

ROSENTHAL R, 1995, PSYCHOL BULL, V118, P183, DOI 10.1037/0033-2909.118.2.183

Rothstein HR, 2005, PUBLICATION BIAS IN META-ANALYSIS: PREVENTION, ASSESSMENT AND ADJUSTMENTS, P1, DOI 10.1002/0470870168

Santisteban L, 2016, PLOS ONE, V11, DOI 10.1371/journal.pone.0154792

Saposnik G, 2016, LANCET NEUROL, V15, P1019, DOI 10.1016/S1474-4422(16)30121-1

Saposnik G, 2011, STROKE, V42, P1380, DOI 10.1161/STROKEAHA.110.605451

Schepers VP, 2006, ARCH PHYS MED REHAB, V87, P184, DOI 10.1016/j.apmr.2005.10.005

Shin JH, 2015, COMPUT BIOL MED, V63, P92, DOI 10.1016/j.compbiomed.2015.03.011

Shin JH, 2014, J NEUROENG REHABIL, V11, DOI 10.1186/1743-0003-11-32

Sin H, 2013, AM J PHYS MED REHAB, V92, P871, DOI 10.1097/PHM.0b013e3182a38e40

Standen PJ, 2017, CLIN REHABIL, V31, P340, DOI 10.1177/0269215516640320

Stucki G, 2002, DISABIL REHABIL, V25, P628

Subramanian S, 2007, J NEUROENG REHABIL, V4, DOI 10.1186/1743-0003-4-20

Subramanian SK, 2015, 2015 INTERNATIONAL CONFERENCE ON VIRTUAL REHABILITATION PROCEEDINGS (ICVR), P38, DOI 10.1109/ICVR.2015.7358582

Subramanian SK, 2013, NEUROREHAB NEURAL RE, V27, P13, DOI 10.1177/1545968312449695

Tabachnick B.G., 2001, USING MULTIVARIATE A

Taylor MJD, 2011, J REHABIL RES DEV, V48, P1171, DOI 10.1682/JRRD.2010.09.0171

Teasell R, 2009, TOP STROKE REHABIL, V16, P44, DOI 10.1310/tsr1601-44

Vargus-Adams JN, 2014, J CHILD NEUROL, V29, P1030, DOI 10.1177/0883073814533595

Vinas-Diz S, 2016, NEUROLOGIA, V31, P255, DOI 10.1016/j.nrl.2015.06.012

Wagle J, 2011, DEMENT GERIATR COGN, V31, P379, DOI 10.1159/000328970

Wahl AS, 2014, FRONT HUM NEUROSCI, V8, DOI 10.3389/fnhum.2014.00381

Weiss P L, 2006, TXB NEURAL REPAIR RE, P182, DOI DOI 10.1017/CBO9780511545078.015

Weiss PLT, 2014, VIRTUAL REALITY PHYS

WHO, 2014, GLOBAL STATUS REPORT ON VIOLENCE PREVENTION 2014, P1

WHO, 2017, INT CLASS FUNCT DIS

Wilson P., 2016, CURR DEV DISORD REP, V3, P138, DOI [10.1007/s40474-016-0083-9, DOI 10.1007/S40474-016-0083-9]

Winstein CJ, 2016, JAMA-J AM MED ASSOC, V315, P571, DOI 10.1001/jama.2016.0276

Yavuzer G, 2008, EUR J PHYS REHAB MED, V44, P237

Yin CW, 2014, CLIN REHABIL, V28, P1107, DOI 10.1177/0269215514532851

Zinn S, 2004, ARCH PHYS MED REHAB, V85, P1084, DOI 10.1016/j.apmr.2003.10.022

NR 125

TC 64

Z9 68

U1 3

U2 37

PU BMC

PI LONDON

PA CAMPUS, 4 CRINAN ST, LONDON N1 9XW, ENGLAND

EI 1743-0003

J9 J NEUROENG REHABIL

JI J. NeuroEng. Rehabil.

PD MAR 27

PY 2018

VL 15

AR 29

DI 10.1186/s12984-018-0370-2

PG 24

WC Engineering, Biomedical; Neurosciences; Rehabilitation

WE Science Citation Index Expanded (SCI-EXPANDED); Social Science Citation Index (SSCI)

SC Engineering; Neurosciences & Neurology; Rehabilitation

GA GB2MT

UT WOS:000428887900002

PM 29587853

OA Green Published, gold

DA 2022-06-21

ER

PT J

AU An, CM

Park, YH

AF An, Chang-Man

Park, Young-Hyun

TI The effects of semi-immersive virtual reality therapy on standing

balance and upright mobility function in individuals with chronic

incomplete spinal cord injury: A preliminary study

SO JOURNAL OF SPINAL CORD MEDICINE

LA English

DT Article

DE Semi-immersive; Virtual reality therapy; Incomplete spinal cord injury;

Standing balance; Upright mobility

ID RANDOMIZED CONTROLLED-TRIAL; CONFIDENCE ABC SCALE; CHRONIC STROKE;

WALKING INDEX; REHABILITATION; AMBULATION; VALIDATION; STABILITY;

COMMUNITY; MEDICINE

AB Background: Individuals with chronic incomplete spinal cord injury (iSCI) commonly face persistent balance or mobility impairments. Virtual reality (VR) therapy is a useful rehabilitation approach; however, little is known about its effects in individuals with chronic iSCI.

Objective: To investigate the effects of semi-immersive VR therapy on standing balance and upright mobility function in individuals with chronic iSCI.

Methods: Ten subjects with chronic iSCI underwent VR therapy 30 minutes a day, 3 days a week, for 6 weeks. Limit of stability (LOS) and the Berg Balance Scale (BBS) were used to evaluate standing balance function. The Timed Up & Go (TUG) test, Activities-specific Balance Confidence (ABS) Scale, and Walking Index for Spinal Cord Injury-II (WISCI-II) were used to measure the subject's upright mobility function. Outcomes were assessed and recorded pre- and post-intervention.

Results: After semi-immersive VR therapy, LOS and BBS scores were significantly increased. In addition, the TUG test results increased significantly over time, while ABC scale scores and WSCI-II levels improved significantly.

Conclusion: This study is the first to assess the effects of semi-immersive VR therapy for patients with chronic iSCI and limited functional abilities. These results indicated that semi-immersive VR therapy has a positive effect and is a useful intervention for standing balance and upright mobility function in patients with chronic iSCI.

C1 [An, Chang-Man; Park, Young-Hyun] Han Seo Univ, Chonbuk Natl Univ Hosp, Grad Sch, Dept Phys Therapy,Dept Med Sci, Seosan, South Korea.

RP An, CM (通讯作者)，Chonbuk Natl Univ Hosp, Dept Phys Therapy, Jeonju Si 54907, Jeollabuk Do, South Korea.

EM dks3597@hanmail.net

OI An, Changman/0000-0003-0995-2983

CR Adamovich SV, 2009, NEUROREHABILITATION, V25, P29, DOI 10.3233/NRE-2009-0497

Biodex Balance System SD, 2010, OP SERV MAN

Bishop L, 2012, J NEUROL PHYS THER, V36, P138, DOI 10.1097/NPT.0b013e3182624c87

Bisson E, 2007, CYBERPSYCHOL BEHAV, V10, P16, DOI 10.1089/cpb.2006.9997

Ditunno PL, 2006, SPINAL CORD, V44, P567, DOI 10.1038/sj.sc.3101876

Ditunno PL, 2001, SPINAL CORD, V39, P654, DOI 10.1038/sj.sc.3101223

Forrest GF, 2012, ARCH PHYS MED REHAB, V93, P1553, DOI 10.1016/j.apmr.2011.08.051

Foster H, 2016, PHYSIOTHER THEOR PR, V32, P536, DOI 10.1080/09593985.2016.1206155

Fox EJ, 2010, PHYS THER, V90, P793, DOI 10.2522/ptj.20090171

Fritz SL, 2013, TOP STROKE REHABIL, V20, P218, DOI 10.1310/tsr2003-218

Gittler MS, 2002, ARCH PHYS MED REHAB, V83, pS90, DOI 10.1016/S0003-9993(02)80019-3

Gittler MS, 2002, ARCH PHYS MED REHAB, V83, pS65, DOI 10.1053/apmr.2002.32160

Holden MK, 2005, CYBERPSYCHOL BEHAV, V8, P187, DOI 10.1089/cpb.2005.8.187

Jackson AB, 2004, ARCH PHYS MED REHAB, V85, P1740, DOI 10.1016/j.apmr.2004.04.035

Jeon HS, 2013, KNEE, V20, P600, DOI 10.1016/j.knee.2012.09.001

Keshner Emily A, 2004, J Neuroeng Rehabil, V1, P8, DOI 10.1186/1743-0003-1-8

Kim JH, 2009, AM J PHYS MED REHAB, V88, P693, DOI 10.1097/PHM.0b013e3181b33350

Lajoie Y, 2004, ARCH GERONTOL GERIAT, V38, P11, DOI 10.1016/S0167-4943(03)00082-7

Lam T, 2008, SPINAL CORD, V46, P246, DOI 10.1038/sj.sc.3102134

Laver K, 2011, AUST OCCUP THER J, V58, P215, DOI 10.1111/j.1440-1630.2010.00897.x

Luque-Moreno C, 2015, BIOMED RES INT, V2015, DOI 10.1155/2015/342529

Mak MK, 2007, ARCH PHYS MED REHAB, V88, P496, DOI 10.1016/j.apmr.2007.01.018

Maurer C, 2006, EXP BRAIN RES, V171, P231, DOI 10.1007/s00221-005-0256-y

McDonald JW, 2002, LANCET, V359, P417, DOI 10.1016/S0140-6736(02)07603-1

McEwen D, 2014, STROKE, V45, P1853, DOI 10.1161/STROKEAHA.114.005362

McKinley WO, 1999, ARCH PHYS MED REHAB, V80, P619, DOI 10.1016/S0003-9993(99)90162-4

Morganti B, 2005, SPINAL CORD, V43, P27, DOI 10.1038/sj.sc.3101658

Pickerill ML, 2011, J ATHL TRAINING, V46, P600

POWELL LE, 1995, J GERONTOL A-BIOL, V50, pM28, DOI 10.1093/gerona/50A.1.M28

Scivoletto G, 2008, SPINE, V33, P259, DOI 10.1097/BRS.0b013e3181626ab0

Shumway-Cook A, 2012, MOTOR CONTROL TRANSL

Song YB, 2014, ANN REHABIL MED-ARM, V38, P160, DOI 10.5535/arm.2014.38.2.160

Thornton M, 2005, BRAIN INJURY, V19, P989, DOI 10.1080/02699050500109944

Wall T, 2015, J SPINAL CORD MED, V38, P777, DOI 10.1179/2045772314Y.0000000296

Wirz M, 2010, NEUROREHAB NEURAL RE, V24, P70, DOI 10.1177/1545968309341059

Yang S, 2014, ANN REHABIL MED-ARM, V38, P726, DOI 10.5535/arm.2014.38.6.726

Yang YR, 2008, GAIT POSTURE, V28, P201, DOI 10.1016/j.gaitpost.2007.11.007

NR 37

TC 16

Z9 17

U1 1

U2 12

PU TAYLOR & FRANCIS LTD

PI ABINGDON

PA 2-4 PARK SQUARE, MILTON PARK, ABINGDON OR14 4RN, OXON, ENGLAND

SN 1079-0268

EI 2045-7723

J9 J SPINAL CORD MED

JI J. Spinal Cord. Med.

PY 2018

VL 41

IS 2

BP 223

EP 229

DI 10.1080/10790268.2017.1369217

PG 7

WC Clinical Neurology

WE Science Citation Index Expanded (SCI-EXPANDED)

SC Neurosciences & Neurology

GA GJ8LA

UT WOS:000435640000013

PM 28880130

OA Green Published

DA 2022-06-21

ER

PT J

AU Andaloro, A

Russo, M

Pastura, C

Sessa, E

Calatozzo, P

Maggio, MG

Bramanti, P

AF Andaloro, Adriana

Russo, Margherita

Pastura, Concetta

Sessa, Edoardo

Calatozzo, Patrizia

Maggio, Maria Grazia

Bramanti, Placido

TI Is there a correlation between dyslipidemia and cognitive impairment in

patients with multiple sclerosis?

SO INTERNATIONAL JOURNAL OF NEUROSCIENCE

LA English

DT Article

DE Serum lipid profile; multiple sclerosis; neurodegenerative disease;

rehabilitation; cognition; neuropsychological dysfunctions

ID BODY-MASS INDEX; CHOLESTEROL; SENSITIVITY; PERFORMANCE; PROGRESSION;

DISABILITY; DEMENTIA; PROTEIN; MIDLIFE

AB Background. Multiple sclerosis (MS) is an autoimmune and demyelination disease of the central nervous system that causes progressive accumulation of disability over time. Recent studies have highlighted the correlation between metabolic disorders and cognitive dysfunctions. The present study aims to evaluate the correlation between components of the lipid profile and cognitive dysfunctions in patients affected by MS. Methods. 90 MS inpatients were included in this study. We divided the sample into three subgroups to evaluate the influence of the presence of dyslipidemia: G1 (patients with dyslipidemia), G2 (patients without dyslipidemia), G3 (patients with a higher than normal lipid value). Patients underwent rehabilitation treatment which included conventional physiotherapy, speech therapy, psychological support, cognitive rehabilitation, nutritional therapy, robotic rehabilitation, cognitive rehabilitation, and virtual reality. Results. The results showed that the three subgroups had a significant improvement in global cognitive functioning (MOCAp < 0.00), working memory (BRB-NV SRT-LTSp < 0.00) and in attention process (BRB-NV SDMTp < 0.00). Only in the G2, we observed a significant improvement in visuospatial abilities (RAO SPARTp < 0.00). Moreover, we found that the cholesterol was negatively correlated with the cognitive functioning score of the patients after rehabilitation and the EDSS score. While the triglyceride scores were negatively correlated with the working memory score before and after rehabilitation. BMI scores were negatively correlated with the visuospatial ability score. Conclusion. Investigating these aspects could help in managing patients, preventing alterations that compromise the patient's quality of life.

C1 [Andaloro, Adriana; Russo, Margherita; Pastura, Concetta; Sessa, Edoardo; Calatozzo, Patrizia; Maggio, Maria Grazia; Bramanti, Placido] IRCCS Ctr Neurol Bonino Pulejo, SS 113, I-98124 Messina, Italy.

RP Maggio, MG (通讯作者)，IRCCS Ctr Neurol Bonino Pulejo, SS 113, I-98124 Messina, Italy.

EM mariagraziamay@gmail.com

RI Maggio, Maria Grazia/ABA-1852-2020

OI /0000-0003-0757-2389

CR American Psychiatric Association, 2013, DIAGNOSTIC STAT MANU, V5th, DOI 10.1176/appi.books.9780890425596

Anstey KJ, 2011, OBES REV, V12, pe426, DOI 10.1111/j.1467-789X.2010.00825.x

Bassuk SS, 2004, CURR PROB CARDIOLOGY, V29, P439, DOI 10.1016/j.cpcardiol.2004.03.004

Craft S, 2009, ARCH NEUROL-CHICAGO, V66, P300, DOI 10.1001/archneurol.2009.27

Cunningham C, 2009, BIOL PSYCHIAT, V65, P304, DOI 10.1016/j.biopsych.2008.07.024

Eskandarieh S, 2018, MULT SCLER RELAT DIS, V25, P143, DOI 10.1016/j.msard.2018.07.023

Eskandarieh S, 2018, BMC NEUROL, V18, DOI 10.1186/s12883-018-1019-2

Eskandarieh S, 2016, NEUROEPIDEMIOLOGY, V46, P209, DOI 10.1159/000444019

Filippi M, 2018, NAT REV DIS PRIMERS, V4, DOI [10.1038/s41572-018-0046-z, 10.1038/s41572-018-0041-4]

Gafson AR, 2018, SCI REP-UK, V8, DOI 10.1038/s41598-018-35232-7

Ghasemi N, 2017, CELL J, V19, P1

Giubilei F, 2002, ACTA NEUROL SCAND, V106, P109, DOI 10.1034/j.1600-0404.2002.01334.x

He Q, 2016, LIPIDS HEALTH DIS, V15, DOI 10.1186/s12944-016-0320-6

Hoane MR, 2011, BEHAV BRAIN RES, V223, P119, DOI 10.1016/j.bbr.2011.04.028

Karastergiou K, 2010, MOL CELL ENDOCRINOL, V318, P69, DOI 10.1016/j.mce.2009.11.011

Loef M, 2013, OBESITY, V21, pE51, DOI 10.1002/oby.20037

Islas MAM, 2019, BIOMEDICINES, V7, DOI 10.3390/biomedicines7010022

Marrie RA, 2010, NEUROLOGY, V74, P1041, DOI 10.1212/WNL.0b013e3181d6b125

McNeilly AD, 2011, BEHAV BRAIN RES, V217, P134, DOI 10.1016/j.bbr.2010.10.017

Meyer-Moock S, 2014, BMC NEUROL, V14, DOI 10.1186/1471-2377-14-58

Nameni G, 2017, INT J DEV NEUROSCI, V59, P15, DOI 10.1016/j.ijdevneu.2017.02.008

Ng TP, 2015, DEMENT GERIATR COGN, V39, P176, DOI 10.1159/000368827

Noori H, 2019, MULT SCLER RELAT DIS, V36, DOI 10.1016/j.msard.2019.101415

O'Brien PD, 2017, LANCET NEUROL, V16, P465, DOI 10.1016/S1474-4422(17)30084-4

Owji M, 2019, MULT SCLER RELAT DIS, V32, P37, DOI 10.1016/j.msard.2019.04.024

Reiner Z, 2011, EUR HEART J, V32, P1769, DOI [10.1093/eurheartj/ehr158, 10.1016/j.atherosclerosis.2011.06.012]

Sabia S, 2009, AM J CLIN NUTR, V89, P601, DOI 10.3945/ajcn.2008.26482

Solomon A, 2007, NEUROLOGY, V68, P751, DOI 10.1212/01.wnl.0000256368.57375.b7

Stepien M, 2014, LIPIDS HEALTH DIS, V13, DOI 10.1186/1476-511X-13-29

Strachan MWJ, 2011, NAT REV ENDOCRINOL, V7, P108, DOI 10.1038/nrendo.2010.228

Tettey P, 2014, MULT SCLER J, V20, P1737, DOI 10.1177/1352458514533162

Thirumangalakudi L, 2008, J NEUROCHEM, V106, P475, DOI 10.1111/j.1471-4159.2008.05415.x

Thompson AJ, 2018, LANCET NEUROL, V17, P162, DOI 10.1016/S1474-4422(17)30470-2

Valladolid-Acebes I, 2011, NEUROBIOL LEARN MEM, V95, P80, DOI 10.1016/j.nlm.2010.11.007

van Vliet P, 2012, J ALZHEIMERS DIS, V30, pS147, DOI 10.3233/JAD-2011-111028

Weinstock-Guttman B, 2011, J NEUROINFLAMM, V8, DOI 10.1186/1742-2094-8-127

Willette AA, 2015, AGEING RES REV, V20, P86, DOI 10.1016/j.arr.2014.03.007

NR 37

TC 3

Z9 3

U1 0

U2 3

PU TAYLOR & FRANCIS LTD

PI ABINGDON

PA 2-4 PARK SQUARE, MILTON PARK, ABINGDON OR14 4RN, OXON, ENGLAND

SN 0020-7454

EI 1563-5279

J9 INT J NEUROSCI

JI Int. J. Neurosci.

PD DEC 30

PY 2021

VL 132

IS 2

BP 201

EP 206

DI 10.1080/00207454.2020.1807980

EA AUG 2020

PG 6

WC Neurosciences

WE Science Citation Index Expanded (SCI-EXPANDED)

SC Neurosciences & Neurology

GA 0K5MD

UT WOS:000560842600001

PM 32767908

DA 2022-06-21

ER

PT J

AU Anderson-Hanley, C

Arciero, PJ

Brickman, AM

Nimon, JP

Okuma, N

Westen, SC

Merz, ME

Pence, BD

Woods, JA

Kramer, AF

Zimmerman, EA

AF Anderson-Hanley, Cay

Arciero, Paul J.

Brickman, Adam M.

Nimon, Joseph P.

Okuma, Naoko

Westen, Sarah C.

Merz, Molly E.

Pence, Brandt D.

Woods, Jeffrey A.

Kramer, Arthur F.

Zimmerman, Earl A.

TI Exergaming and Older Adult Cognition A Cluster Randomized Clinical Trial

SO AMERICAN JOURNAL OF PREVENTIVE MEDICINE

LA English

DT Article

ID PHYSICAL-ACTIVITY; VIRTUAL-REALITY; ALZHEIMERS-DISEASE; ENVIRONMENTAL

ENRICHMENT; AEROBIC EXERCISE; BRAIN; IMPAIRMENT; FITNESS; INTERVENTIONS;

NEUROGENESIS

AB Background: Dementia cases may reach 100 million by 2050. Interventions are sought to curb or prevent cognitive decline. Exercise yields cognitive benefits, but few older adults exercise. Virtual reality-enhanced exercise or "exergames" may elicit greater participation.

Purpose: To test the following hypotheses: (1) stationary cycling with virtual reality tours ("cybercycle") will enhance executive function and clinical status more than traditional exercise; (2) exercise effort will explain improvement; and (3) brain-derived neurotrophic growth factor (BDNF) will increase.

Design: Multi-site cluster randomized clinical trial (RCT) of the impact of 3 months of cybercycling versus traditional exercise, on cognitive function in older adults. Data were collected in 2008-2010; analyses were conducted in 2010-2011.

Setting/participants: 102 older adults from eight retirement communities enrolled; 79 were randomized and 63 completed.

Interventions: A recumbent stationary ergometer was utilized; virtual reality tours and competitors were enabled on the cybercycle.

Main outcome measures: Executive function (Color Trails Difference, Stroop C, Digits Backward); clinical status (mild cognitive impairment; MCI); exercise effort/fitness; and plasma BDNF.

Results: Intent-to-treat analyses, controlling for age, education, and cluster randomization, revealed a significant group X time interaction for composite executive function (p = 0.002). Cybercycling yielded a medium effect over traditional exercise (d = 0.50). Cybercyclists had a 23% relative risk reduction in clinical progression to MCI. Exercise effort and fitness were comparable, suggesting another underlying mechanism. A significant group X time interaction for BDNF(p = 0.05) indicated enhanced neuroplasticity among cybercyclists.

Conclusions: Cybercycling older adults achieved better cognitive function than traditional exercisers, for the same effort, suggesting that simultaneous cognitive and physical exercise has greater potential for preventing cognitive decline.

C1 [Anderson-Hanley, Cay; Arciero, Paul J.; Nimon, Joseph P.; Westen, Sarah C.; Merz, Molly E.] Union Coll, Dept Psychol, Healthy Aging & Neuropsychol Lab, Schenectady, NY 12308 USA.

[Anderson-Hanley, Cay; Arciero, Paul J.; Okuma, Naoko] Skidmore Coll, Hlth & Exercise Sci Dept, Saratoga Springs, NY 12866 USA.

[Brickman, Adam M.] Columbia Univ, Coll Phys & Surg, Taub Inst Res Alzheimers Dis & Aging Brain, Dept Neurol, New York, NY USA.

[Zimmerman, Earl A.] Albany Med Ctr, Dept Neurol, Albany, NY USA.

[Pence, Brandt D.; Woods, Jeffrey A.] Univ Illinois, Dept Kinesiol, Urbana, IL 61801 USA.

[Kramer, Arthur F.] Univ Illinois, Beckman Inst Adv Sci & Technol, Urbana, IL 61801 USA.

RP Anderson-Hanley, C (通讯作者)，Union Coll, Dept Psychol, Healthy Aging & Neuropsychol Lab, 807 Union St, Schenectady, NY 12308 USA.

EM andersoc@union.edu

RI Arfanakis, Konstantinos/AAN-3148-2021; Kramer, Arthur/AAB-2937-2019

OI Arfanakis, Konstantinos/0000-0001-9705-597X; Kramer,

Arthur/0000-0001-5870-2724; Pence, Brandt/0000-0002-4059-9092

FU Pioneer Portfolio of the Robert Wood Johnson Foundation [64449]; Union

College; Skidmore College

FX This study was funded by a grant from the Pioneer Portfolio of the

Robert Wood Johnson Foundation, through the Health Games Research

national program (# 64449); and by faculty and student grants from Union

and Skidmore Colleges. The Robert Wood Johnson Foundation had no role in

the design and conduct of the study, analysis and interpretation of the

data, or preparation or approval of the manuscript.

CR Angevaren M, 2008, COCHRANE DATABASE SY

Annesi JJ, 1997, PERCEPT MOTOR SKILL, V85, P835, DOI 10.2466/pms.1997.85.3.835

Baker LD, 2010, ARCH NEUROL-CHICAGO, V67, P71, DOI 10.1001/archneurol.2009.307

Baranowski T, 2008, AM J PREV MED, V34, P74, DOI 10.1016/j.amepre.2007.09.027

Booth FW, 2006, MED SCI SPORT EXER, V38, P405, DOI 10.1249/01.mss.0000205117.11882.65

Chang ML, 2010, J GERONTOL A-BIOL, V65, P1369, DOI 10.1093/gerona/glq152

Chodzko-Zajko WJ, 2009, MED SCI SPORT EXER, V41, P1510, DOI 10.1249/MSS.0b013e3181a0c95c

Chuang TY, 2006, PHYS THER, V86, P1369, DOI 10.2522/ptj.20050335

Colcombe S, 2003, PSYCHOL SCI, V14, P125, DOI 10.1111/1467-9280.t01-1-01430

Colcombe SJ, 2006, J GERONTOL A-BIOL, V61, P1166, DOI 10.1093/gerona/61.11.1166

D'Elia LG, 1996, COLOR TRAILS TEST OD

Erickson KI, 2011, P NATL ACAD SCI USA, V108, P3017, DOI 10.1073/pnas.1015950108

Etnier JL, 2006, BRAIN RES REV, V52, P119, DOI 10.1016/j.brainresrev.2006.01.002

Etnier JL, 2009, J SPORT EXERCISE PSY, V31, P469, DOI 10.1123/jsep.31.4.469

Fabel K, 2009, FRONT NEUROSCI-SWITZ, V3, DOI 10.3389/neuro.22.002.2009

Fabre C, 2002, INT J SPORTS MED, V23, P415, DOI 10.1055/s-2002-33735

FOSTER VL, 1989, J GERONTOL, V44, pM184, DOI 10.1093/geronj/44.6.M184

Geda YE, 2010, ARCH NEUROL-CHICAGO, V67, P80, DOI 10.1001/archneurol.2009.297

Grandes G, 2009, ARCH INTERN MED, V169, P694, DOI 10.1001/archinternmed.2009.23

Grealy MA, 1999, ARCH PHYS MED REHAB, V80, P661, DOI 10.1016/S0003-9993(99)90169-7

Heyn P, 2004, ARCH PHYS MED REHAB, V85, P1694, DOI 10.1016/j.apmr.2004.03.019

HILL RD, 1993, J GERONTOL, V48, pP12, DOI 10.1093/geronj/48.1.P12

Hillman CH, 2008, NAT REV NEUROSCI, V9, P58, DOI 10.1038/nrn2298

Hogan M, 2005, INT J AGING HUM DEV, V60, P95, DOI 10.2190/PTG9-XDVM-YETA-MKXA

Jak AJ, 2009, AM J GERIAT PSYCHIAT, V17, P368, DOI 10.1097/JGP.0b013e31819431d5

Knaepen K, 2010, SPORTS MED, V40, P765, DOI 10.2165/11534530-000000000-00000

KOHL HW, 1988, AM J EPIDEMIOL, V127, P1228, DOI 10.1093/oxfordjournals.aje.a114915

Kramer AF, 2007, TRENDS COGN SCI, V11, P342, DOI 10.1016/j.tics.2007.06.009

Kwon DS, 2002, 2002 IEEE/RSJ INTERNATIONAL CONFERENCE ON INTELLIGENT ROBOTS AND SYSTEMS, VOLS 1-3, PROCEEDINGS, P2961, DOI 10.1109/IRDS.2002.1041722

Lange BS, 2010, PHYS MED REH CLIN N, V21, P339, DOI 10.1016/j.pmr.2009.12.007

Larson EB, 2008, JAMA-J AM MED ASSOC, V300, P1077, DOI 10.1001/jama.300.9.1077

Larson EB, 2010, INT PSYCHOGERIATR, V22, P1196, DOI 10.1017/S1041610210001080

Lautenschlager NT, 2008, JAMA-J AM MED ASSOC, V300, P1027, DOI 10.1001/jama.300.9.1027

Liang KY, 2010, ANN NEUROL, V68, P311, DOI 10.1002/ana.22096

Lieberman D. A., 2009, SERIOUS GAMES MECH E, P117

Morrison-Bogorad M, 2007, ALZHEIMERS DEMENT, V3, pS80, DOI 10.1016/j.jalz.2007.01.015

Nation DA, 2011, MED HYPOTHESES, V76, P847, DOI 10.1016/j.mehy.2011.02.034

Olson AK, 2006, HIPPOCAMPUS, V16, P250, DOI 10.1002/hipo.20157

Oswald Wolf D, 2006, Eur J Ageing, V3, P179, DOI 10.1007/s10433-006-0035-z

Owen AM, 2010, NATURE, V465, P775, DOI 10.1038/nature09042

Pajonk FG, 2010, ARCH GEN PSYCHIAT, V67, P133, DOI 10.1001/archgenpsychiatry.2009.193

Papp KV, 2009, ALZHEIMERS DEMENT, V5, P50, DOI 10.1016/j.jalz.2008.10.008

Petersen RC, 2005, ARCH NEUROL-CHICAGO, V62, P1160, DOI 10.1001/archneur.62.7.1160

Plante TG, 2003, J HUM MOVEMENT STUD, V45, P485

Plassman BL, 2007, NEUROEPIDEMIOLOGY, V29, P125, DOI 10.1159/000109998

RAND, 2003, EX PROGR OLD AD SYST

Read JL, 2011, JAMA-J AM MED ASSOC, V305, P1704, DOI 10.1001/jama.2011.408

REED BD, 1991, AM J PREV MED, V7, P410, DOI 10.1016/S0749-3797(18)30880-8

Sallis JF, 2003, AM J PREV MED, V25, P110, DOI 10.1016/S0749-3797(03)00186-7

Saxton J, 2009, J NEUROL NEUROSUR PS, V80, P737, DOI 10.1136/jnnp.2008.160705

Scarmeas N, 2009, JAMA-J AM MED ASSOC, V302, P627, DOI 10.1001/jama.2009.1144

Sevick MA, 2000, AM J PREV MED, V19, P1, DOI 10.1016/S0749-3797(00)00154-9

Smiley-Oyen AL, 2008, ANN BEHAV MED, V36, P280, DOI 10.1007/s12160-008-9064-5

Strauss E., 2006, COMPENDIUM NEUROPSYC, V3rd

Studenski S., 2006, SCI AGING KNOWLEDGE, V2006, P21, DOI DOI 10.1126/SAGEKE.2006.10.PE21

Taylor-Piliae RE, 2010, J AGING PHYS ACTIV, V18, P261, DOI 10.1123/japa.18.3.261

Unverzagt FW, 2009, CURR ALZHEIMER RES, V6, P375, DOI 10.2174/156720509788929345

Valenzuela M, 2009, AM J GERIAT PSYCHIAT, V17, P179, DOI 10.1097/JGP.0b013e3181953b57

Van der Elst W, 2006, ASSESSMENT, V13, P62, DOI 10.1177/1073191105283427

van Praag H, 2008, NEUROMOL MED, V10, P128, DOI 10.1007/s12017-008-8028-z

Van Schaik P, 2008, CYBERPSYCHOL BEHAV, V11, P103, DOI 10.1089/cpb.2007.9925

Verghese J, 2006, J AM GERIATR SOC, V54, P1241, DOI 10.1111/j.1532-5415.2006.00808.x

Vinogradov S, 2009, BIOL PSYCHIAT, V66, P549, DOI 10.1016/j.biopsych.2009.02.017

Voss MW, 2010, NEUROPSYCHOLOGIA, V48, P1394, DOI 10.1016/j.neuropsychologia.2010.01.005

Yaffe K, 2010, ANN NEUROL, V68, P275, DOI 10.1002/ana.22143

Yang LX, 2009, J GERONTOL B-PSYCHOL, V64, P470, DOI 10.1093/geronb/gbp040

NR 66

TC 237

Z9 242

U1 9

U2 183

PU ELSEVIER SCIENCE INC

PI NEW YORK

PA STE 800, 230 PARK AVE, NEW YORK, NY 10169 USA

SN 0749-3797

EI 1873-2607

J9 AM J PREV MED

JI Am. J. Prev. Med.

PD FEB

PY 2012

VL 42

IS 2

BP 109

EP 119

DI 10.1016/j.amepre.2011.10.016

PG 11

WC Public, Environmental & Occupational Health; Medicine, General &

Internal

WE Science Citation Index Expanded (SCI-EXPANDED); Social Science Citation Index (SSCI)

SC Public, Environmental & Occupational Health; General & Internal Medicine

GA 879DQ

UT WOS:000299310300001

PM 22261206

HC Y

HP N

DA 2022-06-21

ER

PT J

AU Anderson-Hanley, C

Arciero, PJ

Barcelos, N

Nimon, J

Rocha, T

Thurin, M

Maloney, M

AF Anderson-Hanley, Cay

Arciero, Paul J.

Barcelos, Nicole

Nimon, Joseph

Rocha, Tracey

Thurin, Marisa

Maloney, Molly

TI Executive function and self-regulated exergaming adherence among older

adults

SO FRONTIERS IN HUMAN NEUROSCIENCE

LA English

DT Article

DE exergame; aerobic exercise; cognition; older adults; self-regulation;

executive function

ID MILD COGNITIVE IMPAIRMENT; PHYSICAL-ACTIVITY; AEROBIC EXERCISE; FITNESS;

BRAIN; BEHAVIOR; PLASTICITY; MOTIVATION; BENEFITS; TRIAL

AB The rise in dementia and the evidence of cognitive benefits of exercise for the older adult population together make salient the research into variables affecting cognitive benefit and exercise behavior. One promising avenue for increasing exercise participation has been the introduction of exergaming, a type of exercise that works in combination with virtual reality to enhance both the exercise experience and health outcomes. Past research has revealed that executive function (EF) was related to greater use of self regulatory strategies, which in turn was related to greater adherence to exercise following an intervention (McAuley et al., 2011). Best et al. (20141 found improvement in EF related to adherence to exercise post- intervention. Anderson-Hanley et al. (20121 found that for older adults aerobic exergaming yielded greater cognitive benefit than traditional exercise alone; however, questions remain as to the possible impact of greater cognitive benefit and other factors on participants' involvement in exercise following the end of an intervention. The current study presents follow-up data exploring the relationship between EF, self-regulation, and exercise behavior in the post-intervention (naturalistic) period. Herein, it was predicted that higher EF at the start of the naturalistic window, would predict subsequent exercise with an exergame. Contrary to expectations, results suggest that those with poorer EF are likely to exergame more frequently. The results of this study contradict previous literature, but suggest an interesting relationship between EF, self-regulation, and exercise behaviors when exergaming is employed, particularly with older adults with some cognitive decline. We hypothesize that other factors may be at work, perhaps expectation of cognitive benefit might act as a unique motivator.

C1 [Anderson-Hanley, Cay; Barcelos, Nicole; Nimon, Joseph; Thurin, Marisa; Maloney, Molly] Union Coll, Dept Psychol, Hlth Aging & Neuropsychol Lab, Schenectady, NY 12308 USA.

[Arciero, Paul J.] Skidmore Coll, Hlth & Exercise Sci Dept, Human Nutr & Metab Lab, Saratoga Springs, NY 12866 USA.

[Rocha, Tracey] SUNY Albany, Sch Educ, Dept Educ & Counseling Psychol, Div Counseling Psychol, Albany, NY 12222 USA.

RP Anderson-Hanley, C (通讯作者)，Union Coll, Dept Psychol, Hlth Aging & Neuropsychol Lab, 807 Union St, Schenectady, NY 12308 USA.

EM andersoc@union.edu

RI Maloney, Molly/L-1298-2019

CR Aichberger MC, 2010, GEROPSYCH, V23, P7, DOI 10.1024/1662-9647/a000003

Ajzen I., 1985, ACTION CONTROL COGNI, P11, DOI [10.1007/978-3-642-69746-3_2, DOI 10.1007/978-3-642-69746-3_2]

Anderson-Hanley C, 2012, AM J PREV MED, V42, P109, DOI 10.1016/j.amepre.2011.10.016

Best JR, 2014, FRONT HUM NEUROSCI, V8, DOI 10.3389/fnhum.2014.00353
[truncated: 11,188,700 more chars]
